# Antennal transcriptome analysis of the chemosensory gene families in *Carposina sasakii* (Lepidoptera: Carposinidae)

**DOI:** 10.1186/s12864-018-4900-x

**Published:** 2018-07-20

**Authors:** Zhiqiang Tian, Lina Sun, Yanyan Li, Linfa Quan, Huaijiang Zhang, Wentao Yan, Qiang Yue, Guisheng Qiu

**Affiliations:** Research Institute of Pomology, Chinese Academy of Agricultural Sciences, 98 South Street, Xinghai, Xingcheng, 125100 Liaoning China

**Keywords:** Peach fruit moth, Antennal transcriptome, Olfactory gene, Expression profile

## Abstract

**Background:**

The peach fruit moth, *Carposina sasakii* Matsumura (Lepidoptera: Carposinidae), poses a serious threat to a variety of fruits and causes significant economic loss owing to difficulties in its prevention and control. The olfactory sense is generally acknowledged to be a novel target for pest control. However, a systematic study of the olfactory genes expressed in *C. sasakii* has not been reported yet. Here, we reported the antennal transcriptome of *C. sasakii* using high-throughput sequencing and annotated the main chemosensory multi-gene families.

**Results:**

In the chemosensory gene families, 29 odorant-binding proteins, 13 chemosensory proteins, 1 sensory neuron membrane protein, 52 odorant receptors, 8 ionotropic receptors and 11 gustatory receptors were annotated in the *C. sasakii* antennal transcriptome. The number of olfactory genes obtained in our transcriptome was consistent with that identified in other lepidopteran insects, confirming that we basically accomplished the annotation of the chemosensory genes of *C. sasakii* in the adult antennal transcriptome. All sequences were annotated and analyzed by BLAST (basic local alignment search tool), and some chemosensory genes with specific functions were named according to the BLAST results and phylogenetic trees. Based on the expression profile in the transcriptome and phylogenetic analysis, differentially expressed genes (DEGs) were analyzed in both male and female adults. Finally, fluorescence quantitative real-time PCR was used to identify the male-specific or female-specific chemosensory genes that were putatively related to odor detection and recognition. Moreover, expression levels of OR33 and PBP2 were significantly higher in males than in females, indicating that these genes may interact with sex pheromones. We found some conserved antennal IRs and GRs involved in detecting sugar compounds (GR2, GR5, GR6, GR8) and carbon dioxide (GR1), which were also identified based on phylogenetic analysis.

**Conclusions:**

There are 114 putative chemosensory proteins expressed in *C. sasakii* identified in this study. The identification of these proteins will make the molecular mechanism of odor recognition accessible.

**Electronic supplementary material:**

The online version of this article (10.1186/s12864-018-4900-x) contains supplementary material, which is available to authorized users.

## Background

The peach fruit moth, *Carposina sasakii* Matsumura (Lepidoptera: Carposinidae), is one of the most damaging borers of pome and stone fruits [1, 2], such as apple, hawthorn, pear, jujube, peach, et al. Once the larvae of this insect bore into the young fruits, the pests deteriorate the quality of the fruits, then eat the pulp and release excreta into the fruits, ultimately resulting in significant economic losses. To avoid severe damage in production areas, the peach fruit moth is even listed as one of the important quarantine pests in some ports and markets and needs to be manually checked carefully whether or not the fruits are infested with it before shipment [3]. Due to difficulties in the prediction and control of peach fruit moth infestations, pesticides must be periodically employed based on previous ecological studies of the moth [4]. The peach fruit moth is highly elusive and has a long lifecycle with overlapping generations. The peach fruit moth has gradually been researched in different fields, such as physiology, pathology and toxicology [5]. However, long-term reliance on broad-spectrum chemical insecticides would easily caused pesticide resistance as a result of abuse or unreasonable pesticide application [6–8]. Furthermore, pesticide residues in fruits are harmful to human health and the environment. For these reasons, new methods to effectively prevent and control infestations of this pest must be explored. Some research has recognized that the chemoreception system of insects plays a crucial role in receiving signals, and this is not only suggest chemoreception system are responsible for identifying the signal source from host volatiles or pheromones but also regulate many aspects of insect biological behaviors, which is well established at locating food as well as oviposition, mating, and escaping predators [9, 10].

Therefore, an understanding of the olfaction mechanism may provide a good start to a better understanding of pest control strategies. At present, sex pheromones or other attractants are widely used for emergence forecasts, mass trapping and mating disruption of this pest in the fruit tree economy according to their ecological nature. However, the sex pheromone traps used for the peach fruit moth have much lower attractiveness than those used for other lepidopteran insect pests and, to some extent, do not even trap the peach fruit moth. In 1977, two sex pheromone compounds of *C. sasakii* were identified as the main component of Z-7-eicosen-11-one and the minor component of Z-7-nonadeoen-11-one (20:1) [11]. On the basis of the distinctive chemical properties of sex pheromones, lepidopteran sex pheromones are classified into two groups (type I and type II) according to the length of the chains and features of compound [12, 13]. The sex pheromones of *C. sasakii* belong to type II accounting for ~ 15% of all reported moth pheromones with typical characters of long-chain polyunsaturated hydrocarbons and the corresponding epoxides composed of C17-C25 [14]. However, activity of the second component, Z-7-nonadeoen-11-one, was not detected, and this species was not attracted by neither Z-7-eicosen-11-one nor Z-7-nonadeoen-11-one alone [15]. Therefore, much work is still needed to identify a novel attractant or technique as an alternative to sex pheromones. Additionally, insects are a vast group that interacts with varying levels of specificity, so the study on the molecular basis of olfaction will provide some new insight into key areas of olfaction research, and a combination of behavioral and molecular experiments described in prior studies can be used to screen countless volatile compounds and elucidate the recognition mechanism, and some of compounds may be also important or helpful to humans [16]. At the present stage, although the physiological [2], biochemical and morphological characteristics of *C. sasakii* antenna sensilla have been widely researched [17], the molecular biology of its antenna has not been well studied as of yet. The existing research on the sensilla located on the larval mouthparts and the adult antennae of *C. sasakii* shows that most of them are similar to the sensilla of other lepidopteran insects, but the sensilla of *C. sasakii* differ somewhat in number, external appearance and distribution compared to those of other lepidopterans [18]. Notably, there are 4 types of sensilla including sensilla gemmiformium, malformed sensilla chaetica, and sensilla auricillica II and III that exist only in female antennae [17]. Other research has shown that there are a number of sensilla trichodea and sensilla chaetica at the end of the abdomen [19]. Electroantennograms (EAGs) were recorded from adult *C. sasakii*. The results revealed that adults are able to respond to the 5 ester compounds from apple varieties, and female adults show a strong reaction to hexyl acetate [20]. Consequently, the identification of these functionally olfactory genes, in combination with other experiments, will provide insight into the fundamental molecular mechanisms of the processes involved in the olfactory system.

Odorant-binding protein (OBP) along with chemosensory proteins (CSPs) are regarded as the first step for the transportation of hydrophobic odorants in olfactory recognition [21]. It is supposed that the odorant molecules (ligands) are carried to the olfactory neurons by the OBPs and activate the olfactory receptors [22]. But compared with OBPs, studies have shown that CSPs may be involved in other physiological activities acting as carriers [23, 24]. For example, some CSPs promote the identification of sex pheromones and odorant signaling molecules [25–28], regenerate legs in the cockroach *Periplaneta americana* and affect the transformation of the *Locusta migratoria manilensis* from gregarious to solitary behavior [29, 30]. What’s more, sensory neuron membrane proteins (SNMPs) located in the dendritic membranes of pheromone-sensitive neurons are also essential for detecting pheromone [31, 32]. And ORs are responsible for detecting signals that are transmitted to the brain for further processing, however, later results showed that ORs are not evolutionarily conserved from insects to vertebrates [33–36]. Another receptors family encoding GRs are mainly used for taste or contact stimuli, which play an important role in host seeking behaviors in many insects [37–39]. Besides OR-based detection of odorants, IRs as a new insect chemosensory family have been proved to be involved in odor detection as well in recent discoveries [40]. IRs evolved from ionotropic glutamate receptors (iGluRs) are not related to insect ORs, while both IR- and OR-expressing olfactory sensory neurons (OSN) populations expressing the same receptor innervate the same spherical structures [41].

With the increasing maturation of next-generation sequencing (NGS), an increasing number of olfactory genes in insects have been widely verified by genomic and transcriptomic data. NGS is an effective and novel way to increase our understanding of the molecular mechanism of olfactory recognition in insects, especially in the *Carposinadae* family. However, hardly any olfactory-related genes have been studied in *C. sasakii*. Thus, the identification of olfactory-related genes will be helpful to further study the molecular mechanism of olfactory recognition.

In this study, we sequenced the antennal transcriptome of *C. sasakii* using Illumina HiSeq4000, assembled and analyzed the transcriptome data, and reported sets of putative OBPs, CSPs, SNMPs, ORs, GRs, and IRs. We identified the expression patterns of the olfactory genes via FPKM. Then, quantitative reverse transcriptase (qRT)-PCR experiments were conducted to investigate the adult tissue expression pattern of these DEGs in both sexes. These results may help reveal olfactory receptive mechanisms and lay the foundation for further studies of the olfactory system of *C. sasakii*.

## Methods

### Insect rearing and antenna collection

The larvae of *C. sasakii* used in the experiments were collected from the apple orchard of the Institute of Pomology, Chinese Academy of Agricultural Sciences (CAAS), Liaoning province (Latitude 40.6 + 1 °N, Longitude 120.73 °E), China. The insects were fed immature apples (*Golden Delicious*) picked in July, and newly emerged adults were reared on a 10% honey solution in climatic chambers (25 ± 1 °C, 70 ± 5% RH, 16:8 L:D photoperiod) [2]. We speculated that *C. sasakii* reached sexual maturity when the pupa emerged because most of the insects began mating after 9 o’clock at night on the day of emergence, and a few mated 2–3 days later [42]. Therefore, the male and female moths were kept separately, apart from each other. Antennae were dissected under low light intensity when the male and female moths entered the dark period. Then, the antennae were frozen in liquid nitrogen and stored at − 80 °C. The total number of antennae excised from males and females was 250 each.

### RNA extraction

Total RNA was extracted from male and female antennae with TRIZOL reagent using the manufacturer’s instructions (Invitrogen, Carlsbad, CA, USA). A nanodrop (IMPLEN, CA, USA), Qubit® RNA Assay Kit in Qubit® 2.0 Flurometer (Life Technologies, CA, USA) and Agilent Bioanalyzer 2100 system (Agilent Technologies, CA, USA) were used to detect the purity, concentration and integrity of RNA samples, and RNA degradation and contamination were monitored on 1% agarose gels to ensure the quality of the samples used for transcriptome sequencing.

### cDNA library construction and Illumina sequencing

First, a total amount of 3 μg RNA per sample was used as input material for the RNA sample preparations. Sequencing libraries were generated using the NEBNext® Ultra™ RNA Library Prep Kit for Illumina® (NEB, USA) following the manufacturer’s recommendations. For the preferential selection of cDNA fragments 150~ 200 bp in length, the library fragments were purified with the AMPure XP system (Beckman Coulter, Beverly, USA). Then, 3 μl USER Enzyme (NEB, USA) was used with size-selected, adaptor-ligated cDNA at 37 °C for 15 min followed by 5 min at 95 °C before PCR. Then, PCR was performed with Phusion High-Fidelity DNA polymerase, Universal PCR primers and Index (X) Primer. At last, PCR products were purified (AMPure XP system), and library quality was assessed on the Agilent Bioanalyzer 2100 system.

### Sequence assembly and functional annotation

To ensure the accuracy of sequence assembly and that clean reads were obtained, raw reads of the fastq format were first processed through in-house Perl scripts. In this step, clean data (clean reads) were obtained by removing reads containing adapters, reads containing poly-N and low-quality reads from raw data. At the same time, the Q20, Q30, GC content and sequence duplication level of the clean data were calculated. All downstream analyses were based on clean data with high quality reads. The left files (read1 files) from all libraries/samples were pooled into one big left.fq file, and right files (read2 files) were pooled into one big right.fq file. Transcriptome assembly was accomplished based on the left.fq and right.fq using Trinity (v2.3.0) with min_kmer_cov set to 2 by default and all other parameters set to default values [43]. The annotation of the assembled sequences was conducted by BLASTn and BLASTx searches (E-value <1e-5) against the non-redundant protein database. After the unigenes were obtained using KOBAS (Version 2.0) in KEGG Orthology, the amino acid sequence predicted by HMMER (E-value <1e-10) was blasted against the Pfam database to obtain unigene annotation information [44, 45]. Then, the blast results were imported into the Blast2GO pipeline for GO annotations [46]. FPKM (fragments per kilobase per million reads) values calculated by RSEM (RNA-Seq by Expectation-Maximization) (Version: v1.3.0) with default parameters directly represented gene expression differences between different antennae. Prior to differential gene expression analysis, for each sequenced library, the read counts were adjusted by the edgeR program package through one scaling normalized factor. Differential expression analysis of two samples was performed using the DEGseq (2010) R package. The *P* value was adjusted using the q value. Q value< 0.005 and |log2(fold change) | > 1 was set as the threshold for significantly differential expression [47].

### Verification of olfactory genes and phylogenetic analyses

All of the candidate chemosensory genes (OBPs, CSPs, SNMPs, ORs, GRs and IRs) and their open reading frames (ORFs) were manually verified by BLASTx and ORF Finder in the National Center of Biotechnology Information (NCBI). Moreover, the sequences of contigs with serious errors (mainly insertions/gap/deletions in homopolymer regions) were removed. Transmembrane domains of ORs, IRs, and GRs were predicted using the default parameter of TMHMM2.0 and TMPred, and the N-terminal signal peptide of the candidates OBPs and CSPs were predicted by SignalP4.0 [48]. The amino acid sequences of chemosensory genes identified in the *C. sasakii* antennal transcriptome are listed in Additional file 1.

For verification of the annotation of the candidate chemosensory genes and identification of orthologs, phylogenetic analyses were conducted among *C. sasakii* and other Lepidoptera species with close genetic relationships. For the selected insects, their transcriptomes and olfactory gene functions have been well studied, or their genomes have been published. In addition, since IRs are relatively conserved among different insects, IR sequences from non-lepidopteran species were also selected for phylogenetic analysis in the data set. The available amino acid sequences of chemosensory genes identified in different species were downloaded from the NCBI database to construct the phylogenetic tree. Amino acid sequences were aligned using the Clustalw method by Mega v7.0 [49]. The Maximum Likelihood Tree Method with the JTT model, uniform rates, partial deletion, Nearest-Neighbor-Interchange heuristic method and default automatic NJ/BioNJ was conducted by MEGA v7.0 and subsequently viewed and graphically edited by FigTree (version 1.4.3). To ensure the accuracy of the tree structure, the tree was created with 1000 replicates. The protein sequences of chemosensory proteins used for building phylogenetic trees are listed in Additional file 2.

### DEG analysis based on the FPKM value

To mine the data of the differential expression of chemosensory genes in the transcriptomes, we analyzed the expression of all chemosensory genes in the male and female antennae using FPKM (reads per kilobase of exon model per million mapped reads) values. First, the chemosensory genes in the female antennae were chosen as the reference for expression profiling analysis between male and female antennae. Then, the unigene expression levels were calculated based on the FPKM method. In addition, the corrected *P*-values were used to identify differentially expressed genes using the Benjamini-Hochberg method [50]. The FDR (False Discovery Rate) is considered to be a key indicator in multiple hypothesis testing for screening different genes. To normalize antennal expression levels of candidate chemosensory genes based on the FDR we used log2 to express the fold change. The parameter for filtering the significant differential expression was set to FDR ≤ 0.01 and FC (Fold Change) ≥ 2.

Finally, a total of 27 genes including 14 ORs (CsasOR3, 4, 8, 17, 21, 24, 30, 31, 33, 34, 41, 46, 48 and 49), 3 CSPs (CsasCSP1, 5 and 12), 1 GRs (CsasGR8), 8 OBPs (CsasOBP7, 9, 12, 15, 19, 21, CsasGOBP1 and CsasPBP2) and 1 SNMP (CsasSNMP2) were selected for investigation. Most of them (20) were selected due to their significantly different expression in male and female antenna based on the DEG analysis, and a few of them (7) were selected as genes of interest due to their extremely high or low expression based on their FPKM value.

### qRT-PCR verification for DEGs

Fluorescence quantitative real-time PCR was performed to verify the expression of candidate differential expression chemosensory genes. Different tissues including the head (exclude antennae, 50), thorax (30), abdomen (30), foot (30), wing (30), and antennae (250) were collected from both male and female adults. The extraction of total RNA followed the methods described above. The cDNA was synthesized from total RNA using the Prime ScriptRT Reagent Kit with gDNA Eraser to remove gDNA (No. RR047A; TaKaRa, Shiga, Japan). Gene-specific primers were designed using Primer3 (http://bioinfo.ut.ee/primer3-0.4.0/) (Additional file 3). Then, all of the differential genes and four reference genes including actin, 18S rRNA, elongation factor 1-alpha (EF1α), and ribosomal protein L40 were identified and selected from the antennal transcriptome; Then, the efficiency of amplification was analyzed to verify the different tissues of males and females (Additional file 3). According to the result, we selected actin and EF1α as reference genes for qPCR (Additional file 3). The Bio-Rad CFX96 PCR System (Hercules, CA, USA) and SYBR Premix ExTaq™ II (No. RR820A; TaKaRa) were used for the PCR reaction under a three-step amplification process of 95 °C for 30 s, followed by 40 cycles of 95 °C for 5 s, 60 °C for 30 s, and 65 to 95 °C in increments of 0.5 °C for 5 s to generate the melting curves. Furthermore, the qPCR amplification products were run on a gel to confirm that the size of the qPCR products were consistent with the predicted size.

In the analysis of the relative fold change of these all DEGs in different tissues, the female head (without antennae) sample was used as the calibrator. Two reference genes were used for calculating and normalizing the target gene expression and correcting for sample to sample variation, then means and standard errors were obtained based on three technical replicates and biological replicates. The relative expression levels were calculated according to the comparative 2^–△△Ct^ method.

Data (mean ± SE) from various samples were subjected to one way nested analysis of variance followed by a least significant difference test for mean comparison using SPSS 17.0 (IBM, Chicago, IL, USA).

## Results

### Transcriptome sequencing

To identify olfactory genes, we separately completed the transcriptome sequencing of male and female antennae. Approximately 32.2 and 32.9 million clean reads were generated in male and female antennae, respectively. The mapped ratios (mapped reads as a percent of clean reads) were 75.48 and 74.41% when the clean data was aligned with the Transcript or Unigene, respectively. In addition, the Q30 base percentage exceeded 92.70%. After the adapters and low-quality raw sequences were filtered out and the reads from both the male and female antennae were assembled into a single transcriptome, 66,290 unigenes with an N50 of 1449 bp were generated. The number of unigenes longer than 1 Kb was 11,997, which was listed at Additional file 4: Figure S1 and Table 1. The raw reads were deposited at the National Center for Biotechnology Information (NCBI) - Sequence Read Archive (SRA) database with the submission number SRR5431770 and SRR5431771. In addition, all contigs have been submitted to the Transcriptome Shotgun Assembly (TSA) sequence database at NCBI with the accession numbers GFQL00000000.

**Table 1:** Summary of assembled transcript and unigenes

Length Range(bp)	Transcript	Unigene
200–300	36,802(27.88%)	29,614(44.67%)
300–500	25,046(18.98%)	15,225(22.97%)
500–1000	24,612(18.65%)	9454(14.26%)
1000–2000	23,159(17.55%)	6433(9.70%)
2000+	22,366(16.95%)	5564(8.39%)
Total Number	131,985	66,290
Total Length	146,824,982	47,364,163
N50 Length	2135	1449
Mean Length	1112.44	714.5

### Functional annotation of the unigenes in *C. sasakii*

We used the unigenes assembled in the transcriptome as queries in BLASTx searches of the NCBI non-redundant protein (NR), Swiss-Prot, COG (Clusters of Orthologous Groups), KOG (euKaryotic Orthologous Groups), eggNOG, Pfam (Protein family), GO (Gene Ontology) and KEGG (Kyoto Encyclopedia of Genes and Genomes) databases (Table 2). When the BLASTx parameter E-value was less than 1e-5, 20,006 unigenes (30.18%) were annotated in the above databases. The highest two percentages of unigenes annotated were found in the NR and eggNOG databases and were almost equivalent, with 28.55 and 27.82%, respectively. Moreover, the COG database had the lowest number of annotated unigenes with 5673 unigenes (8.56%). GO analysis showed that most of the unigenes (approximately 84.58%) were not annotated in a GO category.

**Table 2:** Functional annotation of the unigenes in different databases

Annotated Database	unigene	300-1000 bp	≥1000 bp
COG_Annotation	5673	1556	3271
GO_Annotation	10,219	3073	5505
KEGG_Annotation	7699	2306	4225
KOG_Annotation	11,762	3317	6791
Pfam_Annotation	13,081	3736	7781
Swissprot_Annotation	9647	2674	5893
eggNOG_Annotation	18,441	6037	9224
nr_Annotation	18,925	6370	9425
All_Annotated	20,006	6779	9481

GO was used to divide the differentially expressed unigenes, and all of the unigenes were divided into three categories (molecular function, cellular component, or biological process) according to the biological processes and functional annotations (Additional file 4: Figure S2). In the biological process terms, cellular, single-organism and metabolic occupied the majority of both differentially expressed unigenes and all unigenes. In the cellular component terms, cell, cell part and organelle were the most abundant for all unigenes. However, membrane, cell and cell part were the most abundant for the differential unigenes. In the molecular function category, binding, catalytic activity and transporter activity had a huge preponderance of both of them.

### Candidate genes related to transport odorant molecules

#### Odorant-binding protein

A total of 29 OBPs including PBPs and GOBPs were identified using the BLASTx program (Additional file 5: Table S1). The sequence identities of the OBPs with other Lepidopteran insects ranged from 40 to 96% in the NCBI database, with an average of 67%. The further alignment of the amino acid sequences showed that 19 OBPs belonged to the classical OBP subgroup with the motif “C1-X15–39-C2-X3-C3-X21–44-C4-X7–12-C5-X8-C6” (where X represents any amino acid) [51–53] (Additional file 4: Figure S3). The remaining 9 OBPs (OBP4, OBP5, OBP8, OBP9, OBP13, OBP17, OBP19, OBP22, OBP23) were not only outside the range of the plus-C owing to the lack of complete 6 conserved cysteines but also the minus-C subgroups because of noncompliance with their motif. In addition, the classical OBPs that fit the motif encoded complete open reading frames (ORFs) with a sequence length >400 bp, while 5 OBPs (OBP1, OBP4, OBP9, OBP20, OBP22) had no signal peptides.

**Fig. 1:**
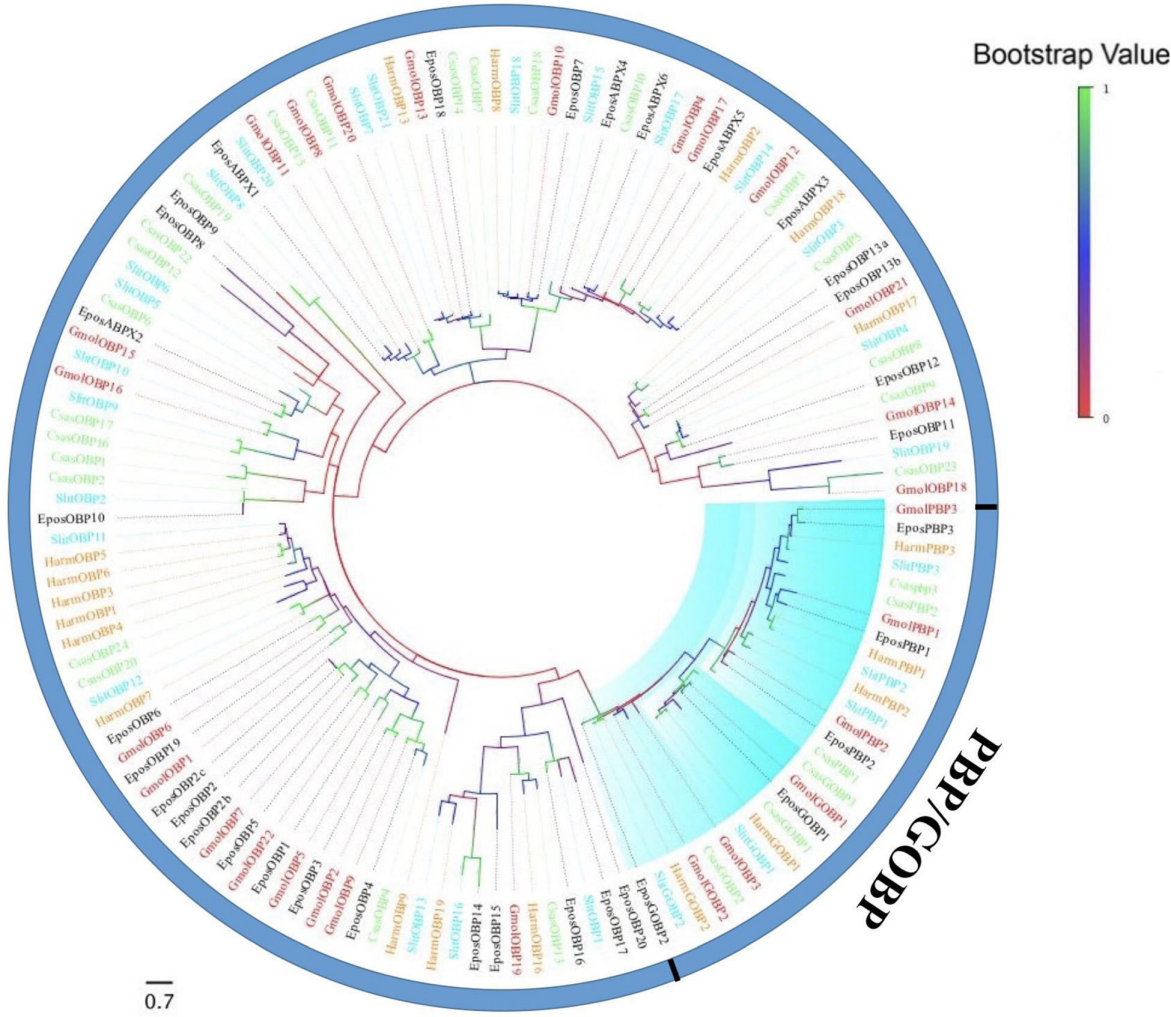
A maximum likelihood phylogenetic tree of putative *C. sasakii* OBPs. A maximum likelihood tree of putative *C. sasakii* OBPs based on the alignment of protein sequences including those from *Epiphyas postvittana* (Epos, black), *Spodoptera litura* (Slit, light blue), *Grapholita molesta* (Gmol, red), and *Helicoverpa armigera* (Harm, tangerine). The orthologous and paralogous groups involved in this paper are highlighted in turquoise (PBP/GOBP)

A phylogenetic tree was constructed using the sequences from four lepidopteran species (Fig. 1). The phylogenetic analysis demonstrated that the lepidopteran PBP and GOBP sequences were highly conserved and clustered into three lineage-specific clades according to their different functions. However, other OBPs showed an extremely divergent trend. Finally, three GOBPs and PBPs were identified, while GOBP3 did not cluster so closely with other GOBP1s/GOBP2s. Further homology matrix analysis indicated that the sequence identity of GOBP3 among each GOBP1 was much higher, ranging from 56.6 to 72.5% and sharing an average of 65.2% identity.

#### Chemosensory proteins

**Fig. 2:**
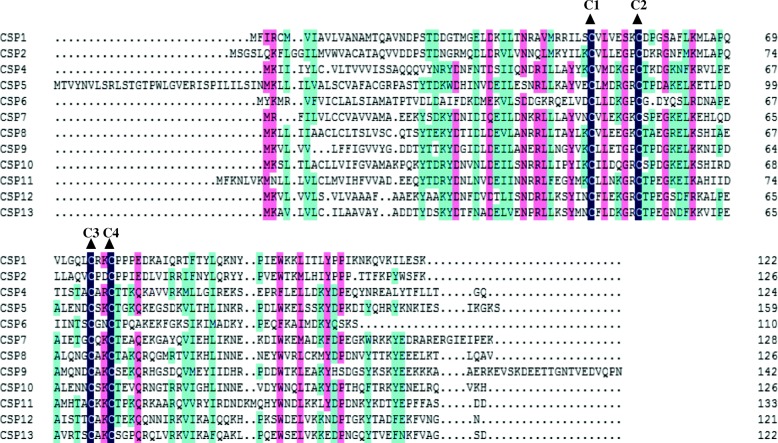
Alignment of candidate *C. sasakii* CSPs. The highly conserved cysteine residues are marked by a dark triangle above

In our study, 13 CSPs were identified in *C. sasakii*. All 13 CSPs have four highly conserved cysteine residues and fit the “CSP sequence motif”, C1-X6–8-C2-X16–21-C3-X2-C4, where X represents any amino acid [54]. Furthermore, all CSPs presented a complete ORF (https://www.ncbi.nlm.nih.gov/orffinder/) and signal peptide (http://www.cbs.dtu.dk/services/SignalP/) (Additional file 5: Table S2). At last, the multiple alignment (except CSP3 due to its longer sequence than that of other CSPs) was generated based on the characteristics of 4 cysteines and approximately 100–200 residues [55] (Fig. 2).

#### Sensory neuron membrane proteins (SNMPs)

**Fig. 3:**
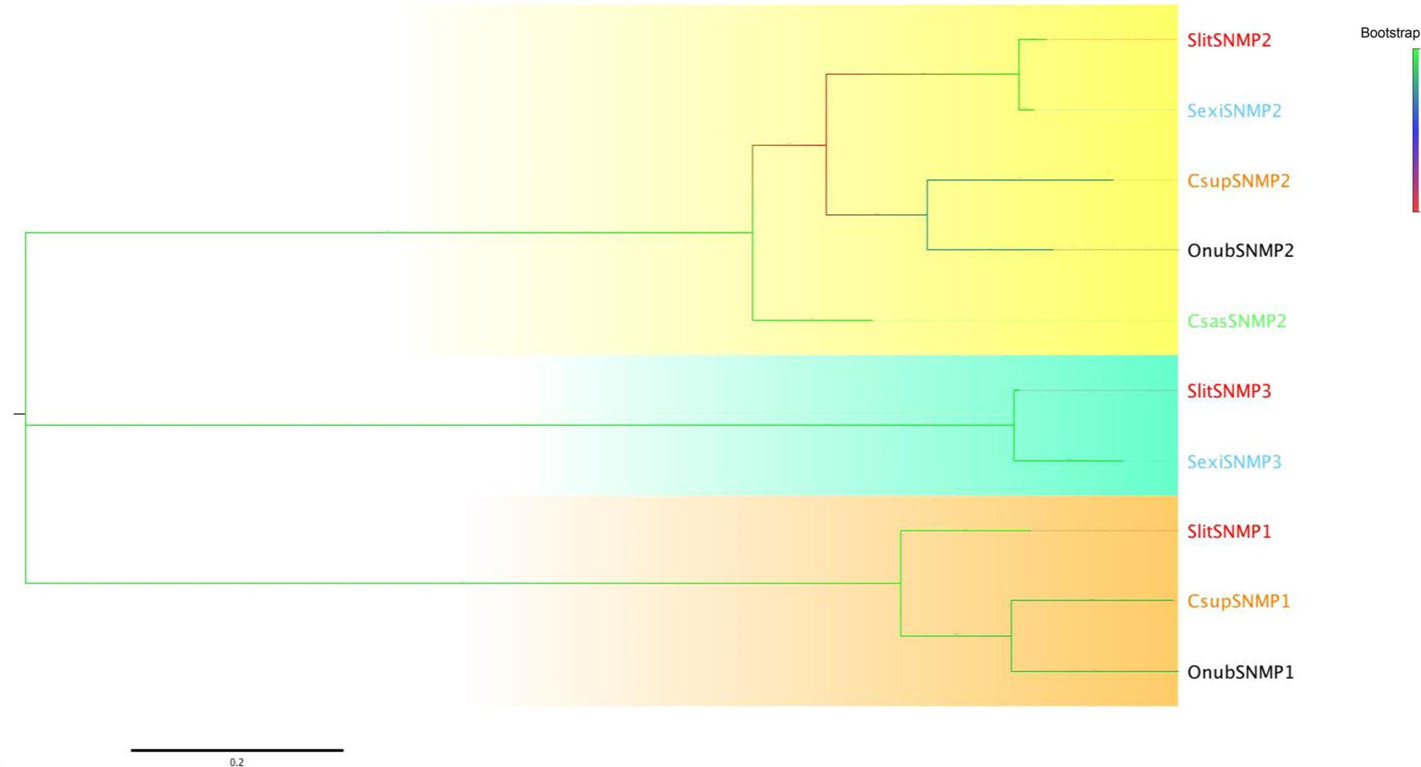
A maximum likelihood phylogenetic tree of putative *C. sasakii* SNMPs. A maximum likelihood phylogenetic tree of putative *C. sasakii* SNMPs based on the alignment of protein sequences including those from *Spodoptera litura* (Slit, yellow), *Ostrinia nubilalis* (Onub, black), *Spodoptera exigua* (Sexi, turquoise), and *Chilo suppressalis* (Csup, tangerine). ML analysis was conducted using MEGA (v7.0)

Only one sensory neuron membrane protein (SNMP) with a complete ORF was identified in our transcripts, and was termed SNMP2 based on the BLASTx and cluster analysis results. What’s more, the length of ORF (SNMP2) was approximately 1500 bp, indicating it was nearly full-length genes (Additional file 5: Table S3). In addition, CsasSNMP2 was more conserved across other SNMP2 variants, with 67 and 72% amino acid identity, respectively. As expected, CsasSNMP2 grouped together with other SNMP2 orthologues (Fig. 3).

### Identification of receptor-encoding genes

#### Odorant receptors

In this study, 52 ORs were identified in the male and female transcriptome, which was more than the number of ORs identified in *Heortia vitessoides* (35 ORs) [56]. In addition, 33 of the ORs were likely full-length OR genes because the length of the portion encoding the proteins was more than 389 amino acids. Of these ORs, the identity of the best BLAST match in the NR database ranged from 35 to 91%. Notably, Orco identified in the *C. sasakii* transcriptomes shared the highest identity, similar to Orco in *Conogethes punctiferalis* [57]. In addition, the transmembrane domains were predicted in view of the sequence characteristics of the ORs. The results indicated that all of them contained 2–10 transmembrane domains (Additional file 5: Table S4).

**Fig. 4:**
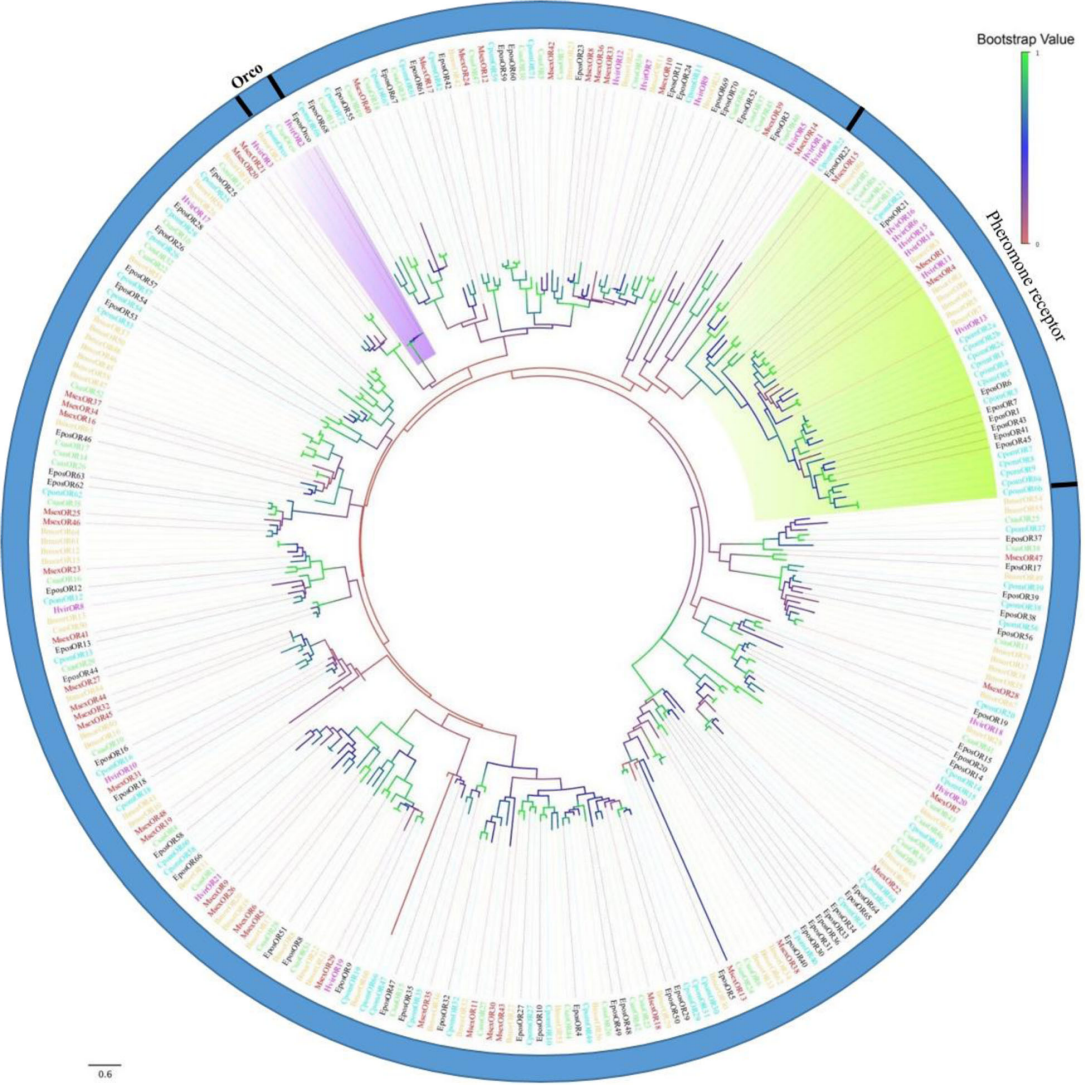
A maximum likelihood phylogenetic tree of putative *C. sasakii* ORs. A maximum likelihood phylogenetic tree of putative *C. sasakii* ORs based on the alignment of protein sequences including those from *Bombyx mori* (Bmor, tangerine), *Manduca sexta* (Msex, red), *Epiphyas postvittana* (Epos, black), *Cydia pomonella* (Cpom, turquoise), and *Heliothis virescens* (Hvir, pink). ML analysis was conducted using MEGA (v7.0) In addition, the gene names of orthologous sequences are emphasized in pink (ORco) and green (PRs)

The maximum likelihood tree was subsequently created by Mega 7.0. To guarantee the reliability and validity of the phylogenetic tree, all of the ORs that encoded proteins were used to build the ML tree based on the multiple protein sequence alignments (Fig. 4). The highly conserved co-receptor (Orco) formed a group indicated by a light purple background, and the identity ranged from 83 to 88%. In the interest of exploring the sex pheromone-binding receptors in *C. sasakii*, some sex pheromone receptors, such as *M. sex* [58], *C. pom* [59], *B. mor* [60], *E. pos* [61], and *H. vir* [62], whose sequences were derived from what has been reported in the NCBI database and has been widely studied, were used to construct the phylogenetic tree. The phylogenetic tree of the pheromone receptors (PRs) with the light green background, indicated that CsasOR3, CsasOR8, CsasOR21, and CsasOR33 were clustered with PRs from other moths. Comparison of the sequences of all identified olfactory receptors in the ML tree revealed a very high degree of diversity, with PR protein identities of 26–98%. These results confirmed that PRs are highly divergent in lepidopteran insects.

#### Ionotropic receptors

**Fig. 5:**
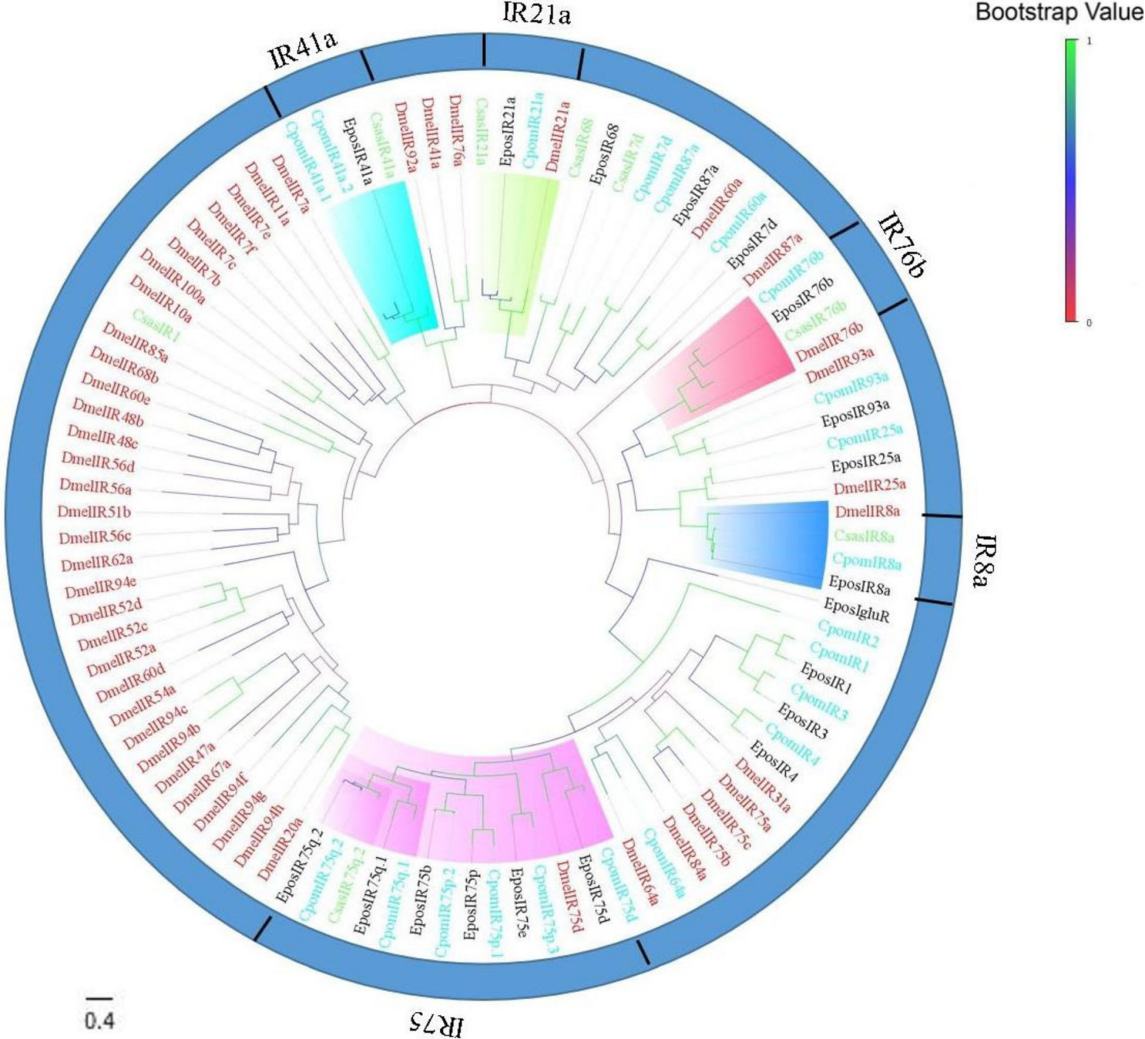
A maximum likelihood phylogenetic tree of putative *C. sasakii* IRs. A maximum likelihood phylogenetic tree of putative *C. sasakii* IRs based on the alignment of protein sequences including those from *Drosophila melanogaster* (Dmel, red), *Epiphyas postvittana* (Epos, black) and *Cydia pomonella* (Cpom, turquoise) IRs. ML analysis was conducted using MEGA (v7.0). In addition, the gene names of orthologous sequences are emphasized in pink (IR75), turquoise (IR41a), blue (IR8a), green (IR21a) and red (IR76b)

Eight candidate IRs were identified in the *C. sasakii* antennal transcriptomes. Some of the conserved IR genes including IR8a, IR21a, IR41a, IR76b and IR75 were present in our transcriptome data. These genes have also been identified in other lepidopteran species and some of them (IR8a and IR76b) are classified as co-receptors, as well as necessary for olfactory responses [63]. Most of the IRs encoded longer ORFs (exceeding 1600 bp except IR7d) than ORs with an average of 1834 bp. The transmembrane domains of IRs ranged from 0 to 8 (Additional file 5: Table S5). To further distinguish putative IRs from the transcriptome of *C. sasakii*, all of the IRs in our transcriptomes were aligned with IRs from *Drosophila melanogaster*, *Cydia pomonella* and *Epiphyas postvittana* by Mega software (v 7.0) for phylogenetic analysis. In the phylogenetic analyses, the IRs identified in our transcriptomes were clustered into the different clades of the conserved IRs (Fig. 5). For example, the IR41a group contained CpomIR41a.1, CpomIR41a.2 and EposIR41a. CsasIR21a was located in the clade of the IR21a group, IR8a group, IR75 group, and IR76b group, which are labelled with blue, pink and red circles, respectively. Finally, we named the CsasIRs based on the results of the phylogenetic tree. Compared to other conserved IRs including IR84a, IR100a, IR20a and 47a widely found in other species, fewer IRs were identified in *C. sasakii*.

#### Gustatory receptors

**Fig. 6:**
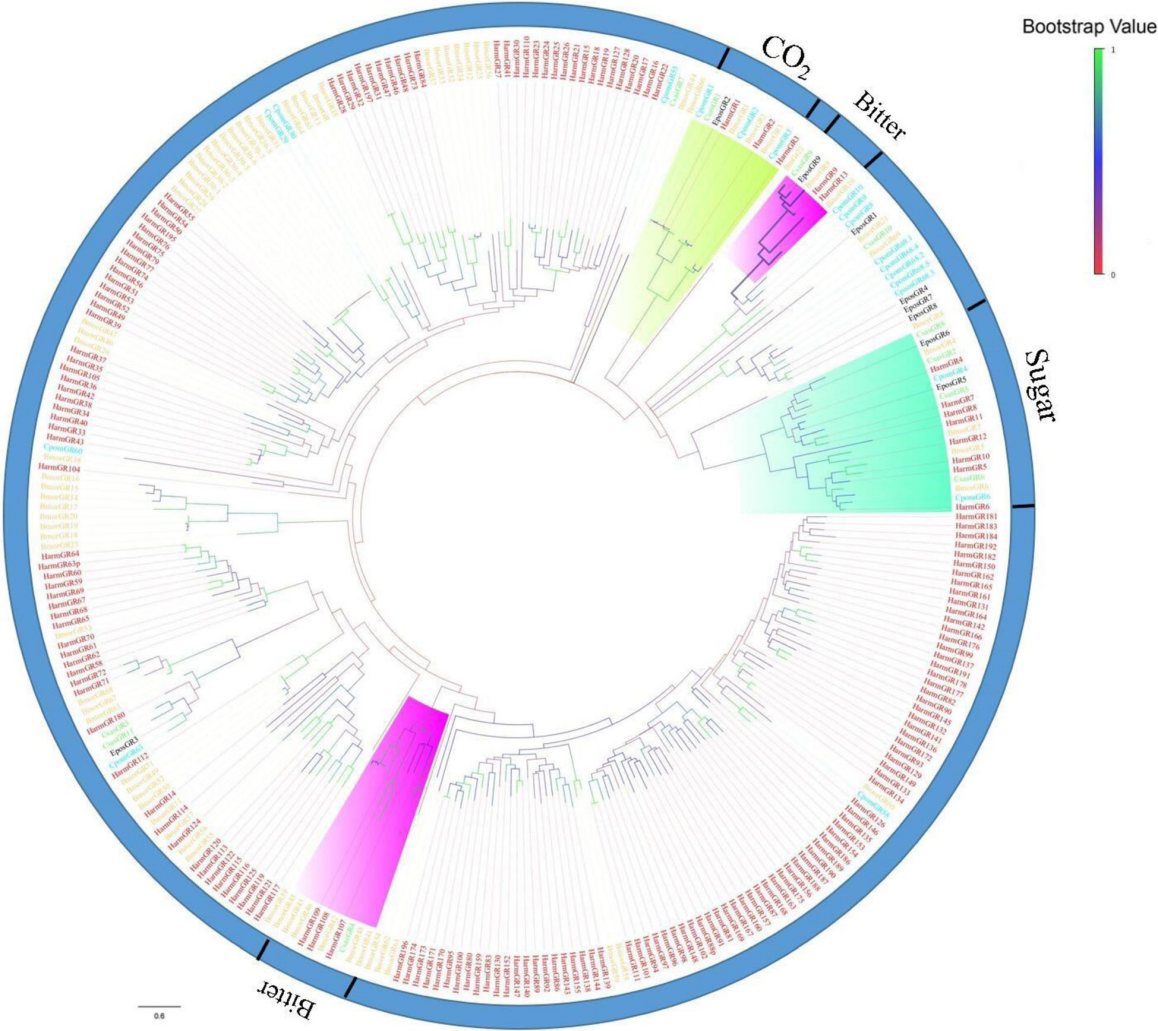
A maximum likelihood phylogenetic tree of putative *C. sasakii* GRs. A maximum likelihood phylogenetic tree of putative *C. sasakii* GRs based on the alignment of protein sequences including those from *Helicoverpa armigera* (Harm, red), *Bombyx mori* (Bmor, tangerine) and *Cydia pomonella* (Cpom, turquoise). ML analysis was conducted using MEGA (v7.0). Sugar receptors, CO_2_ receptors and bitter receptors are highlighted in sea foam, green and magenta, respectively

A total of 11 candidate GR transcripts were identified in both male and female *C. sasakii* transcriptomes with 2–9 transmembrane domains (Additional file 5: Table S6). This result was a little less than that in other Lepidoptera insects, such as *Athetis dissimilis* [64]. However, only four of them (GR2, GR3, GR9 and GR6) encoded an ORF longer than 1000 bp. The phylogenetic tree was used to classify the functions of GRs in *C. sasakii* transcriptomes using the GR genes identified in other insects (Fig. 6). GR2, GR5, GR6 and GR8 were clustered with the members of the candidate sugar detection GR subfamily. GR1 formed a clade with CO_2_ receptors from *Helicoverpa armigera*, *Epiphyas postvittana* and *Bombyx mori Linnaeus*. The bitter receptors included GR9 and GR4.

### Differentially expressed gene (DEG) analysis

Gene expression levels of all chemosensory genes based on the FPKM value in *C. sasakii* were represented in Additional file 6: Figures S2–S7. The expression levels of OBPs showed that 5 OBPs (OBP9, 15, 21, GOBP1, PBP2) were mainly expressed in male antennae and that 2 OBPs (OBP7, OBP19) were highly expressed in female antennae. Only one of the CSPs, SNMPs and GRs was highly differentially expressed between male and female antennae. Meanwhile, the analysis of ORs showed that Orco had the highest expression level of ORs, but there was no difference in its expression between the male and female transcriptome. In addition, 3 ORs (OR31, OR33, OR41) were expressed at significantly higher levels in male antenna than in female antenna, whereas 8 ORs (OR4, OR17, OR24, OR30, OR34, OR46, OR48, OR49) showed the opposite result. In addition, none of the IRs showed a drastic difference in expression between females and males. The expression levels of all candidate GR transcripts were extremely low (Maximum FPKM value < 4).

### Tissue-specific and sex-specific expression of candidate genes

**Fig. 7:**
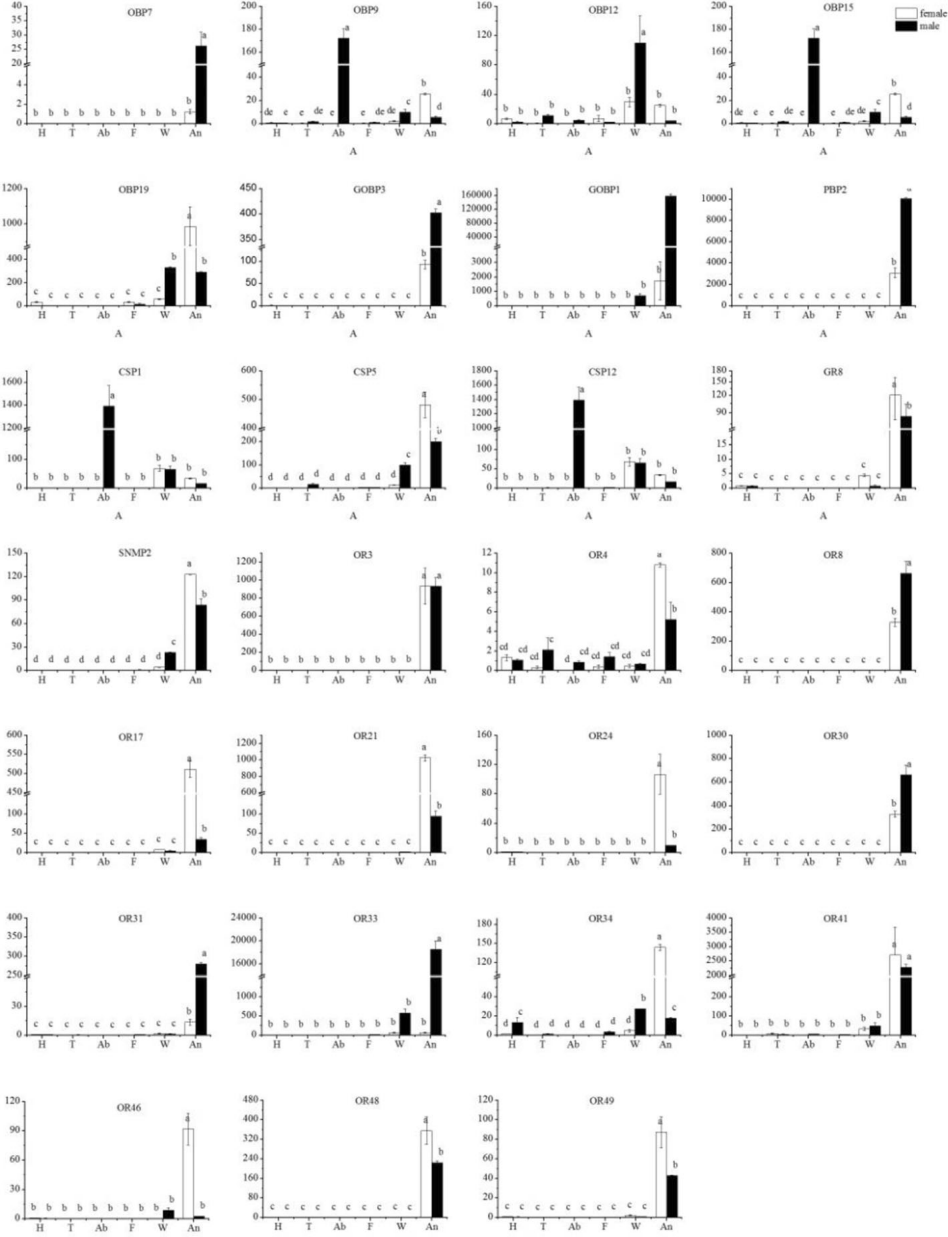
Expression profiles of the candidate genes in different *C. sasakii* tissues. H: head; T: thorax; Ab: abdomen; F: foot; W: wing; A: antenna. Actin and EF-1α were used as the reference gene to normalize target gene expression. Black and white represents males and females, respectively. The standard errors are represented by the error bars; different lowercase letters (a, b, c) above the bars denote significant differences at *p* < 0.05

To better understand and validate the functional role of candidate DEGs in the different tissues from male and female adults, we investigated the expression patterns of these genes via fluorescence quantitative real-time PCR (Fig. 7).

OBP7 was specifically expressed in both female and male antennae but was expressed at significantly higher levels in males. OBP9, OBP15, CSP1 and CSP12 were expressed in other organs besides the antennae in both sexes, but these genes were most abundantly expressed in the abdomen, suggesting a ubiquitous role in *C. sasakii*. GOBP1, GOBP3 and PBP2 were expressed at extremely significantly higher levels in male antenna than in female antenna, and there was little expression of these genes in other tissues. In addition, OBP12 was more highly expressed in the wings than in the antenna. The results for the 14 ORs showed that some of them were expressed not only in the olfactory organs but also in the non-olfactory organs; For instance, OR4 was widely distributed in the tissues examined in our study, and, to a certain extent, 5 ORs (OR17, OR33, OR34, OR41 and OR46) were detected in the wings, particularly in the wings of male adults. However, the expression levels above ORs were significantly higher in the antenna than those in the external genitalia. The expression of OR3 and OR41 was not significantly different between male and female antenna. The expression levels of 9 ORs (OR4, OR17, OR21, OR24, OR34, OR46, OR48, OR49 and GR8) were significantly higher in female antennae than those in male antennae. In addition, 4 ORs (OR8, OR30, OR31 and OR33) were significantly overexpressed in the male antenna compared to those in the female antenna.

## Discussion

Prior to our study, the majority of research on *C. sasakii* was concentrated in the fields of biology and ecology [3, 17, 42]. In recent years, studies on olfactory proteins have increased gradually due to the vital role of olfactory proteins in insects. Our studies will provide novel ideas for population control methods as well as facilitate further study on olfaction in *C. sasakii*.

Based on the BLASTx and GO terms analyses, gene transcripts derived from 114 olfactory genes encoding putative olfactory proteins including OBPs, CSPs, SNMP, ORs, GRs and IRs were identified in the transcripts of male and female *C. sasakii*. These results were similar to the results found in other insects, with 118 and 124 olfactory genes in *Grapholita molesta* and *Hyphantria cunea*, respectively [65, 66]. In comparison to the transcriptome data from *Eogystia hippophaecolus*, the transcriptome data from *C. sasakii* contained fewer olfactory genes [67].

OBPs and CSPs have been considered to be the first step in the recognition of hydrophobic odors in the olfactory process. The number of OBPs identified in our antennal transcriptome was smaller than that in *Epiphyas postvittana*, *Ostrinia furnacalis*, and *Bombyx mori* [61, 68, 69] but larger than that in *Conogethes punctiferalis* [70]. The qRT-PCR was used to explore the 12 of 29 OBPs of the expression files in different tissues based on the differential expression value analysis (FPKM), and the results revealed that most of them were primarily detected in antenna but not attributable exclusively to male and female antenna in *C. sasakii*, which confirmed the expression profile in other lepidopteran insects, such as *H. assulta* and *Agrotis ipsilon* [71, 72]. In addition, OBP7, GOBP1 and PBP2 exhibited highly abundant or biased obvious expression in male or female antennae in our observation, suggesting that these genes may play a vital role in antennal recognition processes. In comparison, CSPs showed high expression but no obvious expression bias in male and female antennae [73]. Moreover, the length of CSP3 was roughly twice that of the other CSPs, which was contrary to the general conclusion that chemosensory proteins were a class of binding proteins that are somewhat smaller than OBPs [31, 74]. Moreover, the amino acid sequence identity of CsasCSP3 with the other CSPs genes from *Spodoptera exigua* and *Spodoptera litura* was still as high as 60%. Furthermore, CSP3 also exhibited the highly conserved four cysteines. In most moths, SNMP1 and SNMP2 are widely expressed in both the antennae and other body parts [75]. A previous study showed SNMP1 may be especially indispensable for the identification of volatiles or pheromones [76, 77]. While only SNMP2 was annotated, this indicated a further study was needed to identify the SNMP1. However, the high expression of CsasSNMP2 in males may be conducive to distinguishing its function in the antenna of male *C. sasakii*.

ORs located and expressed in olfactory sensory neurons play a crucial role in completing the process of odor signal reception and transduction [78–82]. Moth canonical chemosensory receptors (CRs) are comprised of three families of receptors: olfactory receptors (ORs), ionotropic receptors (IRs) or gustatory receptors (GRs) [83–85]. In our study, 52 ORs were detected. The results of the phylogenetic tree analysis showed that the candidate pheromone receptors (PRs) from *C .sasakii* and all of the PRs from different lepidopteran insects were clustered into the same clade in the tree, and the expression profiles of the candidate PRs showed that the expression of OR21 was extremely biased in female antennae when compared to the expression in male antennae, and the OR3 remained almost the same expression both male and female. This observation was contrary to the results that PRs were restricted to male antenna in *Bombyx mori* and other lepidopteran insects [86, 87]. In addition, some recent studies revealed that two PRs identified in *S. littoralis* were expressed in both sexes [88]. This observation may be consistent with theories that PBPs with female antenna-biased expression are responsible for detecting self-released sex pheromones [89–91]. Most of the ORs identified in other insects appear in pairs on the dendrogram [57, 92]. Specifically, among the five male-biased ORs, only the OR33 was clustered into the PR clade. However, beyond that, the qRT-PCR results indicated that the expression of OR33 in the male was nearly 30 times higher than that in the female. Accordingly, OR33 is most likely a PR.

Gustatory receptors (GRs) play critical roles in detecting taste chemicals, mating and finding oviposition sites [37, 93, 94]. Among all of the identified candidate GR transcripts, none appeared to be enriched in the male and female data set. Conversely, the FPKM of GRs indicated that the GRs had the lowest expression levels of all of the identified genes. In the phylogenetic analysis, the function of the CsasGRs was investigated by grouping these genes with other presumed GRs whose functions in the detection of a wide range of molecules including CO_2_, bitters, sugar compounds, including fructose have been studied explicitly. GR2, GR6, and GR8 were clustered into the sugar receptors lineages, suggesting that they likely play a role in tasting sugar. In addition, GR1 formed a clade with CO_2_ receptors, which meant the presence of GR1 in *C. sasakii* was likely to detect carbon dioxide, similar to in *P. xylostella* and *B. mori* [95, 96]. In addition, this phenomenon that more of the CsasGRs found in the antennae transcriptome were clustered to sugar receptors may indicate that that they may perform an important function in sugar-detecting. In contrast, a large number of GRs have been identified in *Helicoverpa armigera* [97], which may be closely related to the host plant defense compounds but also the environment. The majority of GRs in insects showed high diversity, indicating that they were conducive to the specificity or expansion of taste detection [57].

Eight candidate IR genes were identified based on their similarities with other IRs in lepidopterans and physiologic analysis. Earlier studies have shown that IRs have a significant impact on the detection of amines and acids emitted during biological decomposition [81, 98, 99]. At present, IR8a and IR25a are commonly believed to be the co-receptor genes expressed in most of the coeloconic sensilla [81] and are assumed to have a similar function as co-receptor [63, 81]. Notably, co-receptors putatively encoding IR8a were found using BLASTx with reference to these transcripts and an ML tree. In addition, the lower expression level indicated by the FPKM analysis may account for the missing IRs. Other IRs belonged to the divergent IRs. Moreover, when compared to the IRs identified in *Grapholita molesta*, *Cydia pomonella* and *Helicoverpa armigera*, several conserved IRs of the same kind were identified [59, 67, 100–102]. Therefore, the number of IR genes expressed in antennal varied widely across different insects. Meanwhile, a variety of new IR groups have been proven to exist in different insects, such as Diptera [67, 103, 104].

Finally, the tissue- and sex-specific expression analysis showed that the expression levels of 4 OBPs (OBP7, OBP 9, OBP12 and OBP15), 2 CSPs (CSP1 and CSP5), SNMP2 and 3 ORs (OR8, OR30 and OR41) were not consistent with the DEG analysis of their transcript abundances using FPKM values. In addition, OBP12 and CSP1 genes were detected at low levels by transcriptome sequencing projects, but the results performed by qPCR showed that they were abundantly expressed in other tissues of the adult male and female. These differences in the qPCR and RNA-Seq results may be the consequence of the greater sensitivity of qPCR compared to that of RNA-Seq in the male and female antennae, given the depth at which these genes were sequenced. In addition, this sensitivity difference likely also accounts for why fewer IR gene transcripts were detected in the male and female antenna using RNA-Seq.

## Conclusion

The peach fruit moth is regarded as a major invasive fruit-boring pest affecting various fruit trees. However, the olfactory system of the peach fruit moth has not been deciphered as of yet. In our study, the six main olfactory gene families encoding proteins with vital roles in chemoreception were annotated. Although many olfactory genes remain to be identified in this transcriptome compared with the identified genes in other transcriptomes, this study fills our gap in knowledge of the olfactory system in *C. sasakii*. Then, we classified the olfactory genes based on their conservation, predicted transmembrane domains and phylogenetic analysis. Then, sex-biased expression levels of the differentially expressed genes were observed in the transcriptomic data and validated by RT-qPCR. The expression profile analysis revealed that 7 OBPs, 3 CSPs, 1 SNMP, 14 ORs and 2 GRs were uniquely or primarily expressed in the different tissues examined of males and females. Our research offers context and basic foundations for the future identification of the concrete molecular mechanisms of olfaction in *C. sasakii*. Further studies of olfactory function will provide comprehensive methods and original strategies for integrated pest management (IPM).

## Additional files

Additional file 1: Amino acid sequences of all candidate chemosensory proteins identified in the *C. sasakii* transcriptome. (DOCX 37 kb)

Additional file 2: Amino acid sequences used in phylogenetic trees. (DOCX 196 kb)

Additional file 3: Primers designed for fluorescence quantitative real-time PCR. (XLSX 12 kb)

Additional file 4: Figures S1–S3. (DOCX 1489 kb)

Additional file 5: BLASTx annotation against the NCBI non-redundant protein database for putative olfactory genes. (XLSX 35 kb)

Additional file 6: Expression levels of candidate olfactory receptors measured in the RNA-Seq analysis. (XLSX 58 kb)

## Abbreviations

CSPs: Chemosensory proteins
DEG: Differentially expressed genes
FPKM: Fragments per kilobase per million reads
GO: Gene ontology
GPCRs: G protein-coupled receptors
GRs: Gustatory receptors
IPM: Integrated pest management
IRs: Ionotropic receptors
NGS: Next-generation sequencing
OBPs: Odorant-binding proteins
Orco: Odorant receptor co-receptor
ORF: Open reading frame
ORN: Olfactory receptor neuron
ORs: Odorant receptors
PR: Pheromone receptor
SNMPs: Sensory neuron membrane proteins
