# Supplementary material for: Antennal transcriptome analysis of the chemosensory gene families in Carposina sasakii (Lepidoptera: Carposinidae)
Source: BMC Genomics. 2018 Jul 20;19:544. doi: 10.1186/s12864-018-4900-x (PMC6053724; doi:10.1186/s12864-018-4900-x)
Supplement: Supplementary file 1 — Amino acid sequences of all candidate chemosensory proteins identified in the C. sasakii transcriptome. (DOCX 37 kb) [file 12864_2018_4900_MOESM1_ESM.docx]

>CsasOBP1

MYAHEKLSDMVAEQCLNEMYPRKHVEFQESDEACIIFCVYHKFGIMTSNGAINLEIYRKRVQSAHQLDRQVTANDNGNTCAESAEATQHKQDVCKKAKVFNDCTHLY

>CsasOBP2

MVRKISALLCCLCVFGISSSDSAISAENEKRCRNPPTAPQKIERVITLCQDEIKLSILREALDVIKEEHTMPTQRRRNKREVPFTHDEKRIAGCLLQCVYRKVKAVDGYGFPTLEGLVGLYSDGVNERGYFMAVLEASRECLMRNHDLFSRTVPMDNGRNCDVSFDIFECISDRIGEYCGNTGL

>CsasOBP3

MEQRMLSILVITLLCNGIHAMTRAQLKNSAKMFKKSCLGKVDVNEDLIVDIEKGQFVEDRSVMCYIACIYQMSQIVKNNKLNYEASLKQVDIMYPAEMKDAVKATITACKDVSKKYKDLCEASFYTAKCIYTADPKNFVFA

>CsasOBP4

MEMQALKLPKSTNGKCLLACAYKKDGIMTEDGTYNLEHGYKMAELCKNGEEKRLVNGKKLADICSKVNDAKVSDGKKGCERAALIFTCVISNAPKLGFKV

>CsasOBP5

MTPYIFLCIVMAAAGLEAQHLTKEQKDKTLQYTAKCIKQTGVKPELVVEAKKGNFENDEALKKFTLCFFQKAGIIGSDGKLNVDAALAKLPQGVDKKAVGKVLEDCRKKTGKDAADTAFEVYKCYYKATPVHVV

>CsasOBP6

MRLPILTVLSLVAFITFSIGANSPAKSVVTTITTRMPETKEADSQDDSFNLMMLMAECNETFRTETSFIESLNETGSFPDETDRTPKCFLRCILERLEVASLDGVYDASKAAQVATGMGNTTPAADIEEIAGKCADRKEECKCEKAYQFMKCIIETQIKTAQKS

>CsasOBP7

MSSVNRIVAIFSMLIVLGNAGRDKPVFSDDVKEVIEHVHNKCVGKTGVAEEDITNCEKGIFKNDQRLKCYMFCLFDEASLVDKNGCVDFDLMVKMMPDVYSARFESGVNICKKEETMDKEACQRAFDMHTCMYKSDPEFYFLL

>CsasOBP8

MKTFIVFAICLVAAQALTDEQKEKLKKHRSECLLDTKADEQLVNKLKTGDFKTDNEQLKKYTHCLLVKSELMTKDGKFKKDVALAKVPNPADKPAVEKLIDACLANKGNTPQQTAWNYVKCYHEKDPKHSVVA

>CsasOBP9

MGCKNCVILGKEEKAMFRAHSDACLAESGVEPRLVDAMLSGELVDKPALRKHVYCVMLKCKIVAKDGKLQKTALLGKLNNRPDAKNATKVLENCAQQTGDTPVDIAWNLFRCGYDKKALLFNYMPTAPRDDHLENNSN

>CsasOBP10

MKKVLLVFCCVLFISDFSLGMTRQQMKNSLKLMKKTCMPKNDVTEEQVGSIEQGKFLEERNVMCYIACVYSMTQVVKNNKLNYEAVIKQIDMMFPPEMKDPVKASVDSCKDVGKKYKDICEASYYTAKCMYDFDPKIFVFA

>CsasOBP11_

MERLHFVLVTIAMLSVAIAEFPTAEFLEALKPTVEKCEAETGVNKDLVDQFSKGTMVDDPQLKCYMKCIFVEHDLLNEETGVIKYEKMLSLLPQEMKAIAYDMGKNCVHFKGEGGSDLCQVSYDLHKCWQKADPEHYFLM

>CsasOBP12

MLMLKILVCVLPYVVEGNRPLASSDTLVDFADPKVQGHLDAMVRMAQSCVIKVRATPKDVRAYFTNSPPVSRSGQCFAACMLEQSEVISHGKVNRDLLIHLASLVNGKESRVVKKLSSISRMCLDSIEGMSDKCQLASTYNDCLNENMIEFAFPLDIAEEAVRKMPFHLISPNLPPEGRQ

>CsasOBP13

MYKIYCFLIFCATVISADLISSSKGASLKPISACCDIPELADPKPLAECSVPKLPGPCSDVQCIFQHSGFLIDDRTLNKDAYREHLYKWSQNNIAWTAAVKRAISDCVDTDLRQYLDYPCRAYDVFTCTGIAMLKKCPKEFWKC

>CsasOBP14

MPSATLPVLFVLLVFGQAAKEKPVFSAEIEEIIQHVHNQCVDKTGVAEADIANCESGIFKDDNKLKCYMFCLLEEASLVDDEGVVDYDMMVSLIPEQYYDRVTKMIFGCKHLDTPDKDKCQRAFDVHKCSYDKDPDFYFLF

>CsasOBP15

MATRLALVMCTLAALARAKTEMDPEMAELAAMLRENCGDETGVDLALLDQVNAGAKLMDDAKLKCYMKCVMETAGMFGEGAVDVEAVVAMLPEDFRSRNEAMLRACGSRPGADDCDVAWQTQRCWQDGNPEDYFII

>CsasOBP16

MTKLLIITLLALVMSAFTFDQHGNGKRFFSPSLESEPSLSILYARDKKSDKVTNECLTEMYPRNLYKYPLRIDRNDIPCIIHCVLRKFGIMSNDGVINIKNYYKRVQSIHRYDPRILISDVGETCAHNINGMNLDHDVCKKAKVFNDCTQLYAISYSEPDEWTFVK

>CsasOBP17

MCPTNYCLLLFMLFILDITALNCRSGGGHKEEELKNIYQTCLNGNDKKNSSDNKRYSNDNDSNQSKGYSNQRSQWDRRDGIGSRDDRMNDRDDMEDSMGRKDYRNRNRDDSSNGMSSRDERTTYRDRINGRDYRIEGDTGDRSNSRNDMTGKRDDSYGRNFQFTGREDFPQNEDFSSDGRQFNNYRTSTQSPRRYKREKKNQMNSGQRSQYNPNPPQRGSGKDDTKSYRNDGKNSSDDNSMKASDHKACALHCFLENLEMTSEDGMPDRYLVTHVITKDVKNEDLRDFLQESIEECFQILDNEHTDDKCEFAQNLMICLSEKGKANCDDWKEDLHF

>CsasOBP18

MNLVTVVKFLTVLGLCGAMTMKQIRNTGKMMRKTCQPKNNVADEKIDPINEGVFIEEKEVMCYMACIMKMANAIKNNKLHLDSALKQADLLLPEEIKEPAKQAIIACRKYAESSKDICEASFQITKCIYNENPEIFYFP

>CsasOBP19

MIINGFFVSLIVAVAVRGCNGAVDIGKYLKICDRHSPDVNDCLVDAIQEGLDVLAEGIKELGVPAVDPYRQKELKVEYKNNQILVKMILKDILVHGLKASKVQDARLKADEDRFHLEVDMKTPRVQVTGRYHGTGRYNSLKLNATGEFSNNMTDLDYTWKLDGTPEKRDNETYIKINSFYMRPDVTDMKSFLSNDNPESRDLTNLGNTVVNENWRTLYRELLPFAQDNWNRIGVRVANKIFLKVPYDQLFPSKS

>CsasOBP20

MYLENYLGICAGVLVLSFVNVHSLSEEQKNSIELKLLPILTECSQRNGITILDIAVARQTKNIDNIPPCVFACVFKKIGIINNEGLFDIEKAQRKAKKLLQNDEETSSFLTVAETCSSVNKEPRSNDRNGCLLSRELFRCFYRNKEVVGL

>CsasGOBP3

MLSLWLAVILVPGILGNSDVVKDVTLGFGEALKFCREESQLTEDKMEEFYHFWHDDFKFEHRELGCAIKCMSSHFNLLTDSNRMHHENADKFIKSFPNGEVIAETMITAIHNCEKQFDSEPDHCWRILRIAECFKNACKEKGLAPTVEMIMAEFIMVSEP

>CsasOBP22

MHIVTYNLNSFINTCNIARHVVVIRKYAFLYISNLSFCDSMLWHALIFHNGQCFLKLHSCHLKIFKMQVMLWHQIAFVSVIIIIREHSTLGLCTSDLTLRHRNVQILGYVSVIGHTGCGHETGVTGPFL

>CsasOBP23

MDGRVVKVLVFISFISWVHTAQNTATVSPKKVIGIETSYPDGVKIDKDTIITKNMKLMQKTRIVNAAHNKGGDPNLAADDIESIPSELNSEIQAFRRNMTECLKEVGRNDKRKVKSLSPYKDSPIHGECLIACVLKRNGVLVN

>CsasOBP24

MALITFLTVSLAVVSSVYSITPEEKMKFKEALKPALDECGAKYGVTLEDIKKAHESGNDDAIDPCMAGCVYKKIGVINDDGFYVADKSQEDIKNMLSNQDDIDKLNLVVAECSKINEESVTDGKEGCQRAKLLNNCFKNHKELFLRSI

>CsasGOBP1

MAMEKQKQVSLVLGLVLSLLVTNIAGNADIMKDIALGFGEALKHCRDESELTPEKMQAFFHFWDDDFKFEQRELGCAIECMSRHFNLLTEEGKMHHDNADKFIRSFPKGEQIAQQLLDIVHACETKNEAEEDHCWRVLHTAECFIHSAKEQNIAPSVDMLMAEFVVAES

>CsasGOBP2

MVSWLPWLLLVVAASVKGDAVQSESMSHVTAHFGKALAKCREESGLTPEILDEFQNFWREDFEVVHRELGCALICMSNNFELYNDDVRIHHDNMHDYIKSFPKGEELSPKMVELLHNCEKQFDDIVDDCDRVVKMAACFKRDAKAAGIAPEVAMIEAVMEKY

>CsasPBP1

MEVKRCLLVGLITVSCCVFVVDSSQETLKKITSSFMKVLEVCKEELGISENLLAGLYHFWKEEYELVSKEVGCAILCMSHKLNLLDESGKLHHGNAQEFAEQHGAADSTAKQLVSMIHSCEENQEALEDQCLRALEVAKCFRTRIHELDWTPKMDVLVTEVLTDI

>CsasPBP2

MNHVKFLVSVVVALAIDRIDGSADVMREMTLRVGKVIEACKTELDIPDNKMEDFVNFWKEGYELQHRETGCVVMCMSSKLNLLDPDGKLHHGNAHEFAVSHGADDGMASQLVNLVHGCENSIPNNEDACLKVLDLARCFKDGVHKLNWAPNMDLVIGEILAEV

>CsasPBP3

MPSFVKWPTLVLCSIFLIIYVDKAISSQDIMMNLTKGFAAALDKCKKELNIQDHIMQDFFNFWREEYQLVNKEFGCVVMCMASHFDLINEDSKMHHEKAHAFAKSHGADDVLAKQIVTMIHECEKVHEGMSDDCSRVLEIAKCFRGKIHELKWAPNMETLIEEIMTEV

>CSP1

MFIRCMVIAVLVANAMTQAVNDPSTDDGTMGELDKILTNRAVMRRILSCVLVESKCDPGSAFLKMLAPQVLGQLCRKCPPPEDKAIQRTFTYLQKNYPIEWKKLITLYPPIKNKQVKILESK

>CSP2

MSGSLQKFLGGILMVWVACATAQVVDDPSTDNGRMQDLDRVLVNNQLMKYILKCVLLEGPCDKRGNFMKMLAPQLLAQVCPDCPPIEDLVIRRIFNYLQRYYPVEWTKMLHIYPPPTTFKPYWSFK*

>CSP4

MKIIIYLCVLTVVVISSAQQQVYNRYDNFNTDSIIQNDRILLAYYKCVMDKGPCTKDGKNFKRVLPETISTACARCTTKQKAVVRKMLLGIREKSEPRFLELLDKYDPEQYNREALYTFLLTGQ*

>CSP5

MTVYNVLSRLSTGTPWLGVERISPILILSINMKLLILVALSCVAFACGRPASTYTDKWDHINVDEILESNRLLKAYVECLMDRGRCTPDAKELKETLPDALENDCSKCTGKQKEGSDKVLTHLINKRPDLWKELSSKYDPKDIYQHRYKNKIESIKGKS*

>CSP6

MYKMRVFVICLALSIAMATPTVDLDAIFDKDMEKVLSDDGKRQELVDCLLDKGPCGDYQSLRDNAPEIINTSCGNCTPQAKEKFGKSIKIMADKYPEQFKAIMDKYQSKS*

>CSP7

MRFILVLCCVAVVAMAEEKYSDKYDNIDIQEILDNKRLLLAYVNCVLEKGKCSPEGKELKEHLQDAIETGCQKCTEAQEKGAYQVIEHLIKNEKDIWKEMADKFDPEGKWRKKYEDRARERGIEIPEK*

>CSP8

MKLLIIAACLCLTSLVSCQTSYTEKYDTIDLDEVLANRRLLTAYLKCVLEEGKCTAEGRELKSHIAEALQNGCAKCTAKQRQGMRTVIKHLINNENEYWVRLCKMYDPDNVYTTKYEEELKTLQAV*

>CSP9

MKVLVVLFFIGVVYGDDTYTTKYDGIDLDEILANERLLNGYVKCLLETGPCTPDGKELKKNIPDAMQNDCAKCSEKQRHGSDQVMEYIIDHRPDDWTKLEAKYHSDGSYKSKYEEKKKAAERKEVSKDEETTGNTVEDVQPNV*

>CSP10

MKSLTLACLLVIFGVAMAKPQKYTDRYDNVNLDEILSNRRLLIPYIKCILDQGRCSPDGKELKSHIRDALENNCSKCTEVQRNGTRRVIGHLINNEVDYWNQLTAKYDPTHQFTRKYENELRQVKH*

>CSP11

MFKNLVKMNLLLVLCLMVIHFVVADEEQYTDRYDNLNVDEIINNRRLFEGYMKCLLNKGRCTPEGKEIKAHIIDAMHTACKKCTPKQRKAARQVVRYIRDNDKMQHYWNDLKKLYDPDNKYKDTYEPFFASDD*

>CSP12

MKVLVVLSVLVAAAFAAEKYAAKYDNFDVDTLISNDRLLKSYINCFLEKGRCTPEGSDFRKALPEAISTTCAKCTEKQQNNIRKVIKAIQQKHPKSWDELVKKNDPTGKYTADFEKFVNGN*

>CSP13

MKAVLVLCILAAVAYADDTYDSKYDTFNADELVENPRLLKSYMNCFLDKGRCTPEGNDFKKVIPEAVRTSCAKCSGPQRQLVRKVIKAFQAKLPQEWSELVKKEDPNGQYTVEFNKFVAGSD*

>CsasSNMP2

MFGRHTKSLFAISLAFLVAAIVLASWGLPKIISNQIQKNVQIENSSVMFPKWVKLPIPLDFKVRVFNVTNPDEINSGEKPRLAEIGPYVYKEYREKTIISYGDNDTVKYTLKKSFVFDQEASGGLTEEDEVTVINFSYMAAVLAVEDMMPSLVPLVNQALEQFFPDLRDPFLRVKVKDLFFDGIYLNCNGNHSALGLVCGKIKSDPPPTMRPSEDGNGFYFSMFSHLNLTESGPYEMVRGRQNVYDLGHIISYKGKRKMTNWGDEYCGMLNGSDSSIFPPIQEGKVPLRLYTFEPDICRSIYVSLQGKRNIFNMSAYYYELDESTLASKSANPGNRCFCKSNWSNNHDGCLIMGILNLMPCQGAPAIASLPHFYLASEELLEYFSEGVQPDREKHNSYVYIDPTTGVVLEGVKRLQFNIELRKMSVPQLENVPTGLFPLLWIEEGAAISESLQQELKQSHTLLDYAEIFRWVLLALAIVAVIISTVAFARSNAIPIWPLNNNNTVSFVLSAPTVVNKVH

>CsasOR1

MMICLSYQYKNLQSYFYSLEKIQKNCSQREKEILYEQKVKIGIKMHSDTLWCTRYCQLTCSAVITGQIVVNITVLVLLMMQMVNTDRTLTNTLPIVTMGMTLLVTTGVFMCNAGDITIEAHKLGTAIYFSGWENCQEESSGRIKELLLVAMMLSQKPTEIKSHNIIELSYQSYLKIVKSSYSIFSVLY*

>CsasOrco

MMNKVKAQGLVSDLMPNIKLMQAAGHFLFNYHSDNAGMSVLLRKIYASAHAFFITLHFLCLVANMAKYSDEVNELTANTITVLFFAHTVIKQLFFAVNAKSFYRTLAVWNQSNSHPLFTESDARYHQLALSKMRRLLYFVLTVTGMTVVSWVTITFFGESVRMITDKETNETLTEVVPRLPLKAWYPFDAMSGTMYIIAFVFQIYWLLFSMGLANLMDVMFCSWLIFACEQLQHLKAIMKPLMELSAALDTYRPNTAELFKASSTEKSEKVPEPVDLDIRGIYSTQQDFGMTLRGAGGRLQTFGQQDMNPNGLTQKQEMLARSAIKYWVERHKHVVRLVTSIGDTYGTALLFHMLVSTITLTLLAYQATKIDGMNVYAFSTVGYLSYTLGQVFHFCIFGNRLIEESSSVMEAAYSCQWYDGSEEAKTFVQIVCQQCQKAMSISGAKFFTVSLDLFASVLGAVVTYFMVLVQLK

>CsasOR3

MLTNEEILNMKYVKIVRLMLIPIAGWPGEQFGENMLTRKKYLKYYLVVYNTIMTVGVFNYISKFLMTQSFFTAGHSVICASLGILTHIKLCSFHLENYGRTIKTFFTDFHLVNFKDKSKLHMTVYKQIEKISYYTSVFVAISTVMAMISFNMTPMYNNIKNGVFRKERPPNVTIDYIVSYLFFNYNTDDHFYTSTFLICYFNTVMVSLSVAIPDLLFILIVFQIIGHIKVLDKTFQNIPQPKTTRIRCQYKNGNIDVDYAVYGTEENKNIQNFLKECIFHHRTIMSFTNDAASLFGPILGSNYMYQLCNCCILLLELSQGGTDALTQYGTLTFVICYQLIVVSILYEEVHTASSRLKDAVYGMPWESMDMKNQRTVYIILYMIQTPIQVTAFHMVTVGVVTMSKILKSTFSYYAFLRTKKH

>CsasOR4

MAAKNTDLFLERPRKIMNFFGVWFPPENYIILHTIYMLVVMFTQYAFVLCEFIYIAGVLGDMDEVSEASYLLFTQASVCYKSTVFLLNKNNLTQLLEFMERETFSPQTKHHEKLLFLQARTIKRLCTFFLTSAITTCTLWAMIPLFDDAGSRSYPFKIWMPVDPQHSPYYELGYVYQMISIYISAFLFIGVDSVTLSMIMFGCAQLEIIMDKLKTVTICHEHGVSL

>CsasOR5

MNRKFKLSGGKKKVEDLNLQYDISTYDKTYEMPLHVFKYVAIRLTRKDGPIAKILYDAFYWFEFLNLITASTVEFLSMLKTATGGSFQDAVAIFLMLPCIGYVILGKVKSFNIVYHRDVYENIVWELRDMWPTRTLTREEHNIISAALKQHRTVLRIYYWCNNVLLLTFYVPPMIGSLRKYAGIDIQYHLPFLYWLPYDAFQPGFYEVTCIFQMWHGFLTVWLMMTGDLMFCTFITHITMQFDLLSIRIHKFIYVPIDQQLPSSFPLAVYSDEIRKDFETSNKALEKELREIMDRQRALIRLSADLEEMCSFALFVNFFNSSIILCFCGFCAVIVEKWNEVIYKSFLVTALLQTWMICWYGQRLLESSTDVSHALYKSGWYRASSKIKRSILIMIHRAQKEVHVTTYGFSIVSLSSYSAIIRTSWSYFTLLLNVYKK

>CsasOR6

MDLKFKNLFKMSVLSMTLNGAHPQLPKDWKWLIRFAMLHSLSTITFVLLIYNVIVYDTGDIMKACSDCVLVVVYSVVTFKYFIMVYYKKNLSMLIEWMDHEYECAKSLPVEDQEIVKQYAKKGQGAMWLWVVIAIGTGSLFSLKAFVLTSYYAITGEYILVPVYDIVYPFLGDKQYTVGAYWFFVVWFVLFLFYNALMYIANEPLGPIFMLHACGQLDIIRNRVEKLYVNVSTSEQLQTKIKEVVIRLDNVYSFIELINTSFSTTYEFILRATCFLLPITVYLVVEGFKHGLVSLEFFNIIAGTIAICSCPCYYSELIMNRSECLRQAVYSCRWESHWTLNGRGVRSSIIVLLTRLSRPVAINSVFLTLSLDTLANTFHDSYAIFNLMEAAW

>CsasOR7

MAVMKLQLPFLVNYIVFDPFKASVWPFVYVHQVWSTCIACANVYGTDSLLYALCAYIHINFCIISHRFEHMMSWSYNTRDEVRRRLIENVRRHQDLITIVGQVQTLYSESTLFNFVISSFLICLSAFNITFLDDIGAVVAFLTFLTMNISQISLMCFFGDMLMRSSSKVLDAVSRCPWYDADEETKKSILMVIIRQSTEAMQADSCQLWRSHVDIVHNGDESFLVILRSPEDHDGK

>CsasOR8

MANSLQSDIILNLRYIRIIRFFLLLIGAWPGERFGENIHSRKNYFRFVLISYKIAMSFGIFNYLHTYLKTETLITAGHIIIILSLSIVILIRVIFVHLNGYGTIVEKFITDFHLLNFKTVSKNHTTECLRVEKACNYLTKVMATVTLSALCVFNMSPMIYNWRNGVFKKNRPDNITIDYVVNYEWFGFNSDDHFYISTFFLGYFNSYLVSTTVVMLDLFMTIIVLHIIGHINILRNSLKIPPPQTVRVLHINDISSVNYAVFDADENKSIQQMLIKCVNHHRIIKRFATESSSGFGPILALNYLYQVLNICVLLFECSHLSSTNIGKYGPLTFMIVAQLVSISVLFEFVKTASSKLMEDVYAIPWESMDVKNQRTVYMLQFIVQTPLQVTALKMVPVGVTTMQQIIKSSLSYYAFLRTKSTF

>CsasOR9

MSSLRNNIVKFLSKIDYDVTRPNLDLHDYHPQIRILLLSNGVFFNNTDSKLRFVWPFLSCFIAVVAVAFELMFVIHGIMVEDYAFSSECFCYLIMVVCIPIVYLSVLFRRNTVIAVVNAMAADFEYICNHLGPSYKITFLEGQLLIWKLCFAWLAFTIFIGSSFALSPLGFLLYQSLFATQHEYMVRPLMFPMWLPEDDPYRTPNYEIFTLLDILLITFFIQTFCVYMYMQFHILIHYIYIMEMIIMDFKVIFEGLDPSVTKLNKHDPRRMKTQGILNSRMKRIVEWHNSVYKAVESVSTLYGPPLAYQIMISAILICVIAYQVAETLDEGKINVLFSMLLVASIVQLFIPCYLGTKIQNKNYEVGEACWDCGWHESSLGILLRSDIVTVIMRTQKPLTIKFTGLPKVQLETFSSVMSSAYSYFNMLRQYSANIK

>CsasOR10

MSKIPLAYLSLLPHLKNLRYGGVFLIGSENPKYLRALHTIFRRVVMALALLYDLQQVLKVYHERSDTNKAMATMFLLLTHMNSFYKQFIFTKREDKIVVLLDMLKEPIFNQDTPRHIEIQKDHIRRAHIVVKGFYRVAFCTTVIWALYPIVMYQRGTYIEFSIWLPYDASLTRNFYITMMYTWIQTSWLAVLNTTMDSFMAHIMAQAKVQFTILRYDIENVVNNSLEEAEATSTPFNDIFERRFRHILKHYDEILRLCKKFQDIFDGNLLVQFLSSGWIICTGAYRTLDLKPGSVDFMSMLMYLWCMLVEIFLLCYYGNEVNLESENLVQSTFSMNWMDLPMKQQRRVVIFMERIKRPFQPLAGSFIPLSNATFISVLRSSYSFYTFLKRT

>CsasOR11

MSTIRLLETISSALETLRGAWRCDPEAQLRVLGVPLRLLALDIHGEARGFDWLAGLATLIMSSCYYWVFLISWIPYLGRSGALAEHERIQTIVVFSLGVASEIGPVKLICMYINRSRVRQIVSGTIASSLASGSASNEEMRTRLKTVWRRATIFWCSVMMNGVIYMMRPLFQSGRHMMADNLPVLGLQPMLESPRYEVAYVVEILAVLFTVIPAASSAAFLVIVAGHTEAQLHCLAKRLRQIPLQRMLYQTPGEFEAYITNQLHDIIQKHNKALDNVRQLEIAYQISVAIEFILLAAGLIAELLGGLENTYLQVPFALTLVSMDCLSGQRLMDAALEYEMAIYDSGWERWDKRSQRTALNMLLVAQRRARLSAGGIATLNFECLMAVLKSVYSAYTALRSVVDK

>CsasOR12

MSSNVIPTDFKSTMAYAQKLFDISGLPLVDANKLISRNVRFVSISCILLPIIIIQFLYFFMRLNSDTNFLHFANNVACFSMYFQDIIKILVLFTQRKKIRLLVSEIKEMWPSDFDDQLKRCIFLRSIRKMMIFNKIYYIYAITGYSAFIMAPLIFILYKNITLDPIHEYLLPLDLSYPFEIDSISIYLMVYLQQLGTSAMLYCIYIACDIFIVSLAGQLSTLLRLLQLDLGSCVDVNKEDGDYGRIRTAVQNHQKLLRLVDSLNDIFGVLFLLQVTIASISICFFGFQSVYLTGGEALQSYTAAVGMMYNIYFTSWPGQILYDTSSGVAEAAYNSRWYERDVKYRKYMIIIICRAQKPCRLSALGYTDMTLDTFSTILKTSWSYLSLLKSIYER

>CsasOR13

MAVIDFTRFVNPDSELFDFNLKYLFYVGLWPHENWPKYKATLYTCYEISLHILTFAFIVLSTIGTYQNKDDILIVLANLDKSIVVYFFSLRAICFTYKRKDIAILMSEMINSGDTVSIEKKTLMVKTILLTTGLTTTVVTLFSMVAFYNGELTLEIWLPFKANEDLMHLITAVQVVALLFMPGLLRSNGIQGVVCSLIIYLCDQLEELQDRIRSLHFNAENEVAMRENFKDIVQKHVRLMRYSNTTSLLFKEYFLLQNLSLTMELCLNAVMASVTGSDDKNLLVSFIVFLFVALLNAYLYCYLGNELIIQSGGIALAAYETSWTSWPVDLQKRLLVMIRVAQRPLSLSAGGIAVMSVQTYSQVSYSHNKTYDLKTQRRCSIISSTRTS

>CsasOR14

MKYIQNEFKVMKICLNYMIAIGSWEPYPIEGFSNVLFFCYGIFSYLLQTGFFTIGQVIDLCLIWGDVNLMTSTMFLLLTNIALSAKYFNYLLKKTAIRKVINESNDSIKDEVSIEGREFIQRNNFQSICIWYLYIALSYGSIAGWGLTAEKNSLPLRAWFPYDTKKSPAYQITYAYQLIALFFSAFSNMSMDMVMINLMTQCRTRLQLLSLSLKTLGDDLQLTYNKKATPAQEAIMESRLRKCVRQHQSVLDTVTQLRDFIQVPTGIQFAVSMIIICVTAYQLAFESKNITRTVVMGVYLVDMMLQVFLYCNEGNELLGESSEVATAAYHCPWYTCSAGFRKKMVILMVRSTRVTKIIAGGFFALSLPTFMAIIKASYTFFTMLQQVVEDKM

>CsasOR15

MSTVEARPRRYFRIQFLLFRYLGLGWWHHPDEGNTSNFPGLYIYYTIITELLWVAGFVGLETIDPFIGEKDVDRFMFSLSFVVTHDLTIIKLYIFFFKNKEIQEIVHTLEIGLYNFYQNNKRNRATFKLSRIMTGSFIFFGWITIMNTNIYGIVQDYRWKKEIATLNKSSPMPARTLPQPIYIPWPYQSEGSYVFTFALETIGILWTGHIVMGIDTFIGSIILHMGNQFVNLQEAIRTGYDRAMLQLYERIDTSDRIDDTKTIERIVRAHYPENDIDACVEDVVRKCILQYQVLLDCIQKFNDTYTYGFMTQLLSSVAAICSVMVQLSQDASSFKSIRIVTSLAFFFAMIIQLAIQCFTGNELTYQAAQVSDVLMQCKWERMPVRVRRLLMLTMLRAQRPVRLTAAGFANMDNACFLSIMKAAYSYYAVLSQRQA

>CsasOR16

MYGHMMYNVNIDTFIAGLIIIAVTQIKVLNYKFNHLKSPEIENQATDLEYNYMTKLNRYLRNYDIILKYYAMIQDVISVTMFVQFGIASLVICVILCGLIQPSSTETIVFLTLYLFTMTFQLFVPSWLGTQLDFESKELMFAAYNSDWIPRSAAFKRSIWLFIERAKTPIVLTGLKMFPLSLVSFTSIIKSAYSFFTLVRNVQEGQNM

>CsasOR17

MLKIESKLNITMLQKIRVDNPTKSVSLSLTMLRLVGFWRPENLTMKERMVYNCYAFLMFMILLGTYIIIQLVDLHMVWGNIPLMTGTAFLLFTNLSQAAKFLNLVLRRDRIQTILAGSSQILMSQESLEGKQIVKNCNRETSLHQLLYFMLTTITVTGWVSSAEKNQLPMRAWYPYDTSKSPAYELTYVHQIGALYIAASLNVSKDTLVTALLAQCRCRLQILGLELRTLCRGLGGDKVSI

>CsasOR18

MADSEYLSKEFLLPFSFSLDLLGKCHIYYMKTDVQFPKLRLCLNLTIFLLHSLSLYYVLYLLFEGLMDYSDASMTVNIDFIMSQTFIEACLIVQHKNAIKDIIYEINSMWRTTGLSEEQVLLKDKLMGRFILRIKLIYWFCRLTAYLNLFVPLVKSFIRKAILQDEKAELTFPFLGRYPFDATSNVAIYLAVFMFQVYNTMLVVNTYIGHQLLLFSIVSYLTTEFVLLKEDMLILVKENDSDPLRKMEKIKGFIYRHQRLISLVNKTNDIFNAMVFSQLMFGTVTVCVFGFAARILKDTIVAALNALSTIGLLMILINLCDHAERMNGATSEISDLTYNNIIWYKGDVTYQKTILFIILRAQRSCSLTSLKYAPVTLNTFTMVLKTAWSFFSLMSTAYGD

>CsasOR19

MAKVTSEDLFLNRARFIMTCLGVWVPSTTDTILRKSYRHFMISLQYLFLIFQVIYIGQVWGDLAAVSQASYVLFTQACLCFKVTVFHVNMDMLIEILKQMNSDIFKPQSIKHERTLAWQAARIKRLLLAFMVGSQVTCALWALKPLFDDAGNRQFPFDMWMPVKPNRSPHYYFGYAFQLVTISLSADMYFGVDSVALSSVIFGCAQIEIVKDKILKVKSLQANNKTTAEQSKIQEQNYKILIECIKQHQALITFTKLIEDAYHTYMLFQLSGSVGLICMSALRILVIDWFSMQFMSIALYLTVMISQLFLCCWCGHELTASSEELHTVLYECIWYEQDVKFKRTLCFVMMRLGKPIVIRAGHYITLSRQTFVAILRMSYSYFAVLNQTTQRD

>CsasOR20

MFTTVVIVTVQPIGKYFSSSTYRENVKNGTETYLQVVSSWVPFDKNTIPGYLAASLIQIYAAVYGGGWITSFDTNSMVIMVFLRVELELLRRDCAKVFGSELNPVSNDVAMKRLKECHRRHVELVKHAKIFDACLSPIMLLYMLVCSIMLCVTAYQITIEKNPMQRFLMAEYLVFGVAQLFMYCWHSNDVMYMSKDLTLGLYESTWWTRNVMIRKDLHILTGQFKKTIVFSAGPFANLTVPTFISILKGAYSYYTLLNQSQIEKES

>CsasOR21

MAKEARTIVSDNEENRTYIDPLSFKYIKIIRTIYNPIGLFPVEKFGLTSRWRIQRFIHPFLAAIFWIFEINFVVQNFHELDFFLLGHCYLANVTTGMVAIRTYIYTREKYDNLMYEFLAKFHIVHARFNSPYYEKMFRLEQKISANLTNGIMISTFLGSFLYSSLPIYNNIRNGVFGREKGSNVTVDLSIYFLAPNFDPRDHVISATIFNIYASLCCSFMTMISDLVVYLMVIQIIAHIYGLINDLENIPKPKRFNDRRTFRHEDMIIPLVDMYDAEENAEVLSILGRCIEHHKMIVTFTHEISSFYGPMLAYNYFYQVTSLCVQLIEISRGDMNTFFRYIFFACLVFSELILMSVIFEAVNHTSEKVIQSVYDIPWEYMNTSNRRIVSVLLHRVQSPIKVTAIGMVPVSLGTMTAILKTTFSYFAFLRTLG

>CsasOR22

MGKQFAFNIKSSKVDHIVSVVDGPIFAPQNQYHVDVMKRNAREMSVLLTVSHVGVLSCGILWTIFPAVNKLLGDDVQFTAYFPFDTSQSMIFSIIQIYMSILIAVQAYGNVTMDCTIVAFYAQVRTQLQMLRFNLEHLADFDETKIDTIRTQSNGSIVYKDVVNRERMQTRLVRCVKHYQLIVWFCNEIEIIFGEAMVVMVLVTAWVICMTMYKIVKLSILSVEFLSMVVYLLCMLSQFFIYCFYGTQVIVESEHINESLYSCDWLALTPRFRKQLLIMMQYCTRPLAPRTAYIIPMSLETYISVLRFSYTLFTFLNRK

>CsasOR23

MKTKLLDYFENPNYPLLGPNIWSLDKVWLFLPKTLINKILCLSLHGSAMLFVLSQYIELYVIKTDLDEVLNNLKISMLSTICVLKAGTFLMFNDRWRKIIDYITEADKYERDNRDKIKGQIIDGYTKYCRRISFSYWFLVFCTFVTVTQTPTLKYGTSAALRYNFRNGTQAFQHIFSSYMPFDKNSFPGLPATILWHFCICVYGAWVVAAFDANVVVMMVFFGGQLEIVRERCMHMLDDYDYTDTTEVQAYASICKLHEMHVELIKHSKLFNSVLSPVMGIYMMMCSLMICASLYQFTGDISAAQKIIVAEYVVFGVAQLFVFCWHSNDVLVRVSS

>CsasOR24

MKKNDLVFEDIFKITTIAMHVTRSHPDIKRNIVWIVQVIPYMSLSLWAFINLTRSVIYTDIPNKDYENACKTGIMGIMSVTITYKYYNLVLCQKSIVGLISIMNQDYEISKNLPIEDQIIVRKYCAYGVNVSKFWLVCAFVASSVFPVKAFTLMAYYYWRGEPRFVSMFEFTYFSCIEYYKYLTWVNWLLFGICFAFGLLAMSMYIAFDPVAPIFILHACGQIELLVKKLNNVFLKKTDIKIVETEFKSIVVKLQQIYGFINEVDQNFTVFYEYNMKATTIMIPLSAFQIIKSYHQNEINFEFISFFFGCILQFFVPCYYSDLLMDKGEKLRQAIYSCGWEEQRSRKLRKLVLLILTRASRPLILRSIFNTICLDTFAEMCRQSYTIFNLMNALE

>CsasOR25

MFSLNTVKKYCSEFKMKLFDNSFDSLLWIVSFLPNVMGFAIYYEKVRISFWIIHFSLLIYVYGLGCSTYIINNVQGIDDYIKNIVNLGLLAIIINSGYWYLMQRDLLRSFIAETKISIELLGKSPEIQKEYYKMLSLVRKMLFVFMGTNTTNFLTSYLPNRINLNNKFSMNPCVGMEPLTSSPNKEICRCVLIVQEITIHSTAVYFQGLLIFLIAHTAVIFHMLSIEIMTLNHIDEQLDLGERDKFIKKKLISMIRRHVLILSIVDKLKSLFSIPIGINFGTNFLCISLFFFISLADWVRYVPILIYCFLVFFLYCFLCQAVILGAEKFEVAIYSCGWENFNLEGKKIIFAMLVQSQKTVEILAADIVPVNISTFATTCQAIYKFVTIIKF

>CsasOR26

MLFLGAFWRWLTNTAALNQLNGDYERMFFEEVYRVVYLTGFSSYDKGIVYLLYSSTVKLLIALFICSEIWYLYSELSSLDTIVENISVTLINLLSVYRYIDLIRNKDIFTKLATAMESRYFDVSTQTRKELVNFWVLRSLSYLRLILYLGTCTLAAWYVYPLVDDLEYNLCVAVRLPFDYRTPLRYTIVYIVVVVAFNYAAFFVMLNDIIMQSYLMHLLCQFTVLADCFENIVTDCETECGGKINRSHLQSNKEFTDTYLKRLRNLSEQHKIILNNTMRLRAVLSTPMLGQLGASSVLICFAGYQVSTTVSISITKCMMSLFYLCYNMFELFIFCRWCDEIKTQSENICQSIYFSGWELGLVTVPGVKRRLMLIVERAQRPMVLTAGGLYELSLVSYTAIVKVSYSALTVLLRLR

>CsasOR27

MEDGIVSIIEDRLNKTGLISTKRIVKTIKWIGIICFVATTATQVAALIISKDDPEKFFECCSILSFCGMGILKLCSLCRDNDSWHLLIEQVSVIEKEQLHTEDNLVDYDSDGDNKEKENAFMRNIRTYTSSHHFTAKVLSRFYYFTLVIFIVSPFTEYVIRVSQGYEYKGYPHILPCWSPLDDVSFSGYLLSIFAEIIASIYCVLVHIAFDLSATGLMIFSSGQFVLLREYSETIAGRGLYLFLSKKRDDRAHYRIKTSYKYHVELVW

>CsasOR28

MMAAIDCLAFCALVFVEYKFRVLQLYFGELKAKYVINVGKKSSAQLIFEFKKCLIVGMKFHENALWCASSVQYSLGNLYSVQIFQSIALVVLCLIKLVVTDHYLIYIVANMVYLACVLILTAIYMKAGGDITYEASMISDSIFYSGWEVMAADRELRCLCVVAIQRSNVPVIMSAFRVIPLSYSNFISVLRYSYSFFAVMY

>CsasOR29

MKVTEYIREKLSIIAPVLPYGVLESWDDLNPRLYHAVHIYWLKFYGMWYNSFEPTQLLFWVQMTYMLVVFWLVCFLPGIGEAVYLLRRKDNIADVADGLYLFLSEMYTYFKIAVFWLNKDKIIGLLDYLHCREFKPQEQEHRVIIRRSIQTARSVMTYYSTMCVGAVSVGIVMPLTEGLNVLPTNVEYPYVDVLSSPTYEALYIHHIYYKPATCIIDGVMDTILAAFVASAIEQIDILAHTLRNFEEIADRRRARAQCDYSKEYYVKTVLKDSIKHHNSIIQYVAMIERAFSLASALQFMLSVMVLCLIGIQFLSIENPSSHPMQIVWMAIYLTCMLIEVFILCWFGDELIWKSMDLQQAAFEGPWRSVDPKTAKLIIVFLERCKRPMKVTAGKIFTLSLDTYTILINWAYKAFAVMSNMKK

>CsasOR30

MPENELEIAQIQIFETLKWSEKCTRQIGIEVTDVSTNTFCGKTGKKCLILLGQTMLFLLVLVQLCHIIEGMVNGDLVYAVLGIHITGYGMLGLGKWVTLQKKQHVIKELVYQLAEIWPVHLEDPEAVEIKRSTLLKLRIVQACYIMFNISGILAYTVFPFIQFLVMMYILGKPADLGYAFFATYPFDKTKPVYHEISFVFESVGGILSVFSMLGSDVMFISMASHISMLLRLLQIRIGRITSAVESGDGVHELFDCSQEAINVIKVHQRLIKYSEDLEDAFTVSNFINILMSSVNICCVTFNIVLIEPVTEYSNKLFLAATLNQVFLICWYADEMYRSSLGVSDAVYKCDWFKCNNRCRRIFLLMIVRSQKALCFTALKFSSITIATFTSILTTSYSYFTLLYTMYSE

>CsasOR31

MYIYLHILMHMYYLTQMIILDIEVIFDGLDESVATLYKHDQRRKEVQGILNSRMKRIVEWHDKVIKYLKLVSNIYGPILVYQITCSSFAISIMVYQIAEKFEHGFFDLLMVALCISCTAQIFLPCFLGTLIQNKSYEIGDAFFYSGWEGNRLAPLIRNDIVMVMCRTQQPLSIKFTGVPIVSIQTFSSIMSTAYTYFNLLRQYSHAKER

>CsasOR32

MNSARVEKINNLMRGHDTLFAARTKKDEEIIMKNNKAMHRLVKAFMYTICMSNGSWIASHFMSRLRDDAAIVPVYMPFKVETWTQFTWSVMIESVFPLLWIGYGHLTLDMTIATYYANSRTQLKIIKYNLEHLFDRNVPLNMSASHSYVDEVDTTVRERFIHYVQRYDMVVWYTRQVDRVFKGAMIFQILSSCVVYCITVIKISQSDARKDSAALIFLLLFFVVMLSQVFVYCYYGGLVEYESKLLNDSLYLSDWTSASPAFRRMLLTAMCRWLRPLTPRAAHSIPLSLTTFFMIMRSAYSLYTVLVSTAQKG

>CsasOR33

MEPETQIKEVDVEIAEDTKVNPLSFKYIKVVRLFYDPLGVFPVERFGEKSRLRPLRFSYPAIVILFWSAEVVFVIRNYKTMDFFLIGHCYLTNVTTGMMVIRVAMYMMKTYDDIMEEYLKSFHIVHSRALSPYYEKLFNYEQKFSSMFAMAMMIFTCTGMLLYSSLPIYNNFHTGVFGREKGSNLTIDLSIYYYFPFYNPVEHVVSACILNFYLSYLCSFMTMVSDLVVYLMIFQIITHIYCLLDDLANIPKPKNFGKNSTFQHYDMIIPLAEMYDAQENAIIQGILGRCVHHHRIIVTFADKISSFFGPMLAYNYFYQVTSLCMQLIEISRGDFNTFVRYVFFTLLVFSELIFMSVIFEKVGSTSEQLIEAVYDVPWEYMSISNKRIVSVFLHRVQSPISITAIGMVPVGLGTMTGILKTTFSYFAFLRTLNS

>CsasOR34

MTFLESVKEDFLKELNFISLMGSKIFLYPFIGRSKLKLMGYYFIYFLLLFTSTQLLLTFCLNGFSDWMEIINMAPNIGVCVMSTIKFTTIHKNRELYREIFYHFQNDLWDLIPESVNNPKIMAKYKRIAMFINRFMVYYSFPLIVIVDSIPWLIMNYESRVLGKEIEHRYPFDAWYPFDKVDYYYAAYFWESFMTAIVICIYAYSNILHISFIAFTCMELKLLGNCISTLFSHEDIKDIHNGINADTIHNKINRKLRRIIIRYEFLTMITSKLDAVLGDIMTLSYIFGATFICLTAFSFTVVDDLFKSVRYFSFFVSLLCEMFDQCIIGQVLSDHSEELTDAIYTSDWIYADKKTKTLLLMLMLRTQRPLKLTANKYIVMNLNTFSGICSTSYQFFNLLRTIYHP

>CsasOR35

MVLRDIISGPMLGQLAASGVLICFIAYQAVTNFKENFIKSVMALFYLGYNLSQLFIICRWCEEIKTQSGNVKEAIYRSGWEQGIASMPGVKVRMLLLSARAGEPVVMTAGGMYDLSLESFNKVSNMLRADFSHGPDSSSIGYNLMLSLNLIEIEAASTLSLQPILSLGKSVFINFVFLSLRSEKSH

>CsasOR36

MLYQIAEKLDHGKFDFLMVVLCLTCTIQIFLPCFLGTLLQNKNRLAQLIRNDILLVICRSQQPLTIKFTGLPIVSIQTFSSIMSTAYSYFNLIRQYSNAKHK

>CsasOR37

MPMRTPFIEMAWFFHTIFLFEVSTTIILDMWFVLLIFLLCAANESTAKRLSVEDIMENESETEYAKRLNDVLRIFYHEQLKQVKYLKTLNRMYKWLAFVPLCNAAMCMCIMLLIMSKEVNWKFAPHVMPMFAEIFAYNWFGEQIKTKAQEIKMALLNFDWTSLDAKGKKCYYILILQMNRESGIRTAVGNDLSLVTMSMVLKVTYQSFTVLQSMGD

>CsasOR38

MLRKIRKTSDIAENSELLKEKYRVWNRNLKQIIICYYSFNLFLYLVLYLHDRVDFSNEGYIVNCYGVRPLNKSPNNEICVCVILVQEGFLQILLHTYQAVIIFIIFQCTIMYKILSEELRDLQSHSYDVVKVKIRYLIDQYAVTLKAIEDLKCLYSAAIGGNFAVTAFDMCLIIYLPVEEAANFSAILLHSFLVFFLYCFLCQRLINTSEVFERDIYGCGWENFEVKERITINIMLMMAQKPVELLAAKIVPVNIYTFASTMQGIYQFLAVVKF

>CsasOR39

MHMQLLGHDFEKIAIERKQVQNAQHDERFRLEFKELVQWHKQVIRATKVLDTMYAIPTLYNFFISSVLICLTGFNVIAVKDVARAITFFSFLFVNMVQIFTLCYLGDMVMFASIAISDGVYRSRWYIAEASVAKDLLMIQQRAQKPCKFSACGYADVNLAAFMKILSTSWSYFCLLNTMSS

>CsasOR40

MSVQKFFKELSNNKALCDSSGEYETSFFAPIYRFGYLSSYSYYDRSVGYVVYSTVIKLALAVFLYCELFLTEMSTLDQLADNVNIVVIQIIGFYRYYNMSSNKAVFKELAATMTSPYFDTTTMRRREIVQYWCDCNYRYFKLLLSLGIATMFAWYVYPVVDDLDYNFMIPMDLPFHYQTRVRYTYMYLTMIVFFNYFPCMVIFNDTVIQGHLINLICQFNVLADCFENMLDECKRGFEDISYNIDELVLNEDFRKHYIQRLGQLVEQHKFILT

>CsasOR41

MKDSNEIETSNQKYKDFNETFKYCTFGLAMGLLYPNRSNVRKRQFYCVVVVLINGFTLFWFCMYLYKCITIVDLYNLSNNITIGVLITIFFFKLIYCNYKTEEFAVLLEKISSDLLKANELAEDYQEIYDDYIRQAKVGQMLWLLIPVLVSSQFPIYAGCGLIYITFKNDAMDRRGYMVHEMQLKFLEDKQYETPYFEIMFAYTCIQCITLAPNFAGFDGSFCIATTHLRMKLHIMLHNLRKCFKEAKDSSDIRRKLKSTVMEHQEAVAFYEELQDIYGSWLLTIFLLTSLLISMNIYQLYLSDHINAKYTMFGISSVLHMFAPCYYASTLTKYSEDLSSEIYSVPWEVDMDPWVIRILTFMITKSQQRMILTGNGMVVYNMELFISIMQTSYSLFTLITMT

>CsasOR42

MLSSVCVVKVGSFLIFHDRWKKIIDYVTEADKQERDTQDKVQREIIDRYTKYCRRISFGYWFLVFCTFISVSQTPTLRYGFSSTSRENFRNGTEPFEHIFSSYIPFDKNSFPGLPVTIVWHFLMCAYGAWLMAAFDSTVVVIMVFFGGKLEIVRERCKHVLGRRQEFTEDQMYASISELHDVHVELVK

>CsasOR43

MKPFTMFHKTYYVTALFMSVGMIYPNPHTDRARITFIIFLIVTSLPLCFTMLLDIYNSWLRYDILNIIRHSTVLGPFLGGFFKMLLMFTKRVKAGELIKEFNRDLSLYNDLSAPYKNAARASIRNSNIYSERCWLIAIASCVSLFPLMAICQMLYSVKDVPTKYMIHDTNKPFSKDLDARFESPYFEFMFVYSLFYSMWYIINFVGYDGFFGLCMNHACLKMKIYCMMLEDAFKLDNISDVRRAIVKVIDEQIRTYKFIGNAQETFNEWLGLIFVATIIQICTCMYQVTEGYSLDVRYMIFMIVSVAHIYVPCRYAAKLKDTSVRTAVDFYCSGWEDLNDQSTRKMIMFMMARAQIPIIMSAFKMIDFDMKLFVSMLQSSYSMFALLKS

>CsasOR44

MAQRGITFLKGLEDPKYPLLGPNIRGLYLFGLWLYGSKLRTYCFYIIHSFSIMFVFTMTVQLYLYIKRNDYAKMLENISITALSIVAISKISYVVTHMGKWRELVENISEEERNVIKSRDPEILQITKDYTMYARLVTYFFWGIVLFTNAVTLVTPFITCLTPDYVEKLNNGTEVLPHILNSWFPFDDSKGYGYIASVIIHAYMTTQGAGIIASYDAHAMVIMTFFKGQMMILGSKCRRLFVIDDLITSEGIYSRIKECHRLHNYLMTQFKLFDSLLSPVMFVYMLVCSITICCSVIQLNLPNTSLSQKLWAIEYAAAQIFQLFLYCWHSNDILMESLAVDRGVFNNDWWAADTRIRRHLVMLAAKLNNVFQLKAGPFTRLSMPTFIDIMKGSYSFYTLFAQIPENK

>CsasOR45

MDRTFSIFNRVLSFAGITIYKKENWNSKLWLSFQIFNVIIGFFTFIFTSGFVLTHLSNLVICIEAASIWTTGVIMFISLCVCLIFKNDFQDFLTEMGFADGVLEMPLINYVISRQTLLGKPDQIWDKRLPRLKELHSLVIDSQETLLRYSRKLFIAYVLSVWLVATLYLCDPIYRMAISEDKDLRLLAFDMWLPWSLQNFKVYVASFIFHVYAGYLCCYAYPGLQLTIIFLVGQTIRQLKIISFIILHLNEIAIELLEKRDERWQECCSDLLKQCIVHFVKIKKFSNQLNVICRPFYLALILVAIMLVCVCSVRIAISANKLSLGILKYYVHEFCFIIVVMTFCLLGQQVQNECEKLESAVVEKWYMFDRRHKIMVRIFNMALSQRIPIYIFGSITLSLPTFTWFIKTGMSFFTLVMSVLEDNNY

>CsasOR46

MFKRLRSIFYKEYVDLTQEIEPKRFYTTFYFWLKAFCVIDDEPIPNWAYITVAFLRACYSLAVLLALISTSYGIHSFDLYHITDCGSYVIILSYGLEINLQATIDKKHIYYLQQCIREDFAYVHHEGRKYREQFIGKIIETWQLQIISIVVIFVSSITMAIFTLFVLMWYLWTYQEGDVWQRPLPIPFWLFTVDFHASPLYEFTFSFIIMMTVLFPYNYIFMLQTEVTWIREITLKADLVIWALCDLLTDIDPNYSEEDTSMLLKDRMKGIIIQHQSMFRLIHTFALVYRKNLLFEQKFIGPLVCLSAFCFFEKLEHGEVQVFLMLLGLTAVFLVFLPCWLCTYLDGKNKSIIDACLNIPFWNVKGKILRPYLVLMMQMSLKPLPLTRAGFEEISIQTFSAKMASAYSYFNLLRQSNISI

>CsasOR47

MADSILEDAKREIDSTLSLCTFSMHRIGLSFDPPNTASALFRQKLMFTASVLGICYHVFSEIVFIAITLANSPRVEDVVPLFHTFGYGALSIAKVGVLWYKKDVFGHLLQELVGIWPMPPMEEAAKAIKDQKLLALKIAHQWYFMVNVAGVWFYNLTPIGVYLYHVWAGHDATIGFVWVSWYPFDKHQPIAHVVVYIFEIFAGQTCVWIMICTDLLFSGMASHIGLLLRLLQRRLLTLAETTKTDEENYQEILDNIKLHQRLITYCNNLEEAFSLVNLINIVLSSVNICCVVFTIVLLEPLMAVSNKLFLGSALIQIGMLCWYADDIFHANADVASCAYNSHWYRTSPRCRRALIFLIQRAQKPIAFTAMNFTNISLVTYSAILTRSYSYFALLYTMYSDN

>CsasOR48

MAEKPPNTSLNTEKINDYIHYVVWPLKLVACWDWYPNPTQLYQDMINNIHLALVLFVLVHTPIALFVHLYIEWVDVMSSLKTIADGLPPVVSTAIVAYFGMFKKDLYELVDYMNSNFKYHSARGLTNMTMLKSYASAKRFAHVYTASTISSVSIYVFLPVFLHLWTKEPLQPWIYTNADHSPFFEMVFLRQCLVQAFIGLAMGQLGVFFASNAILICGQLDLLCCTARNVRFTALLSNGVRHDCLRKQFSMIQDDEEHSYLYNVAEIKDSVYHYDRKVDMSSVNKKTHFDIYSPEYDAATDEALAECARMNQIVQRYIELFERFVSPLVAVRVVQVTLYMCTLLYAATEKFDLITGEYLMAVALDIFIYCYFGHQITLQTDNVSMALYQSNWPSMGGKTRRMLLNCLLSTKRATDVRAARFLPMNLHTYIAIVKNSFSYYTLLVNVNERKKG

>CsasOR49

MEVLKNLPMEFVKPFVLSFDMLHRGGAGFLLDVRYPRNVKKYVRFVFTIPCILVTYVSLSPAFLDVFGEKLDLSLLANLVALYGTVIQGFFKIVVVCKNKREIKSVTTRLGTIWRMTDLNGEQIKKKNGMLKMLRYFQSVFYWVNMAGLWQYIVAPLVETLFCAFMSKEECEWLFPFPCTFPFNPTRNWLIYILTYVFEAYGIYTAICLYLSPEFLVITLCAHLSTEFMLLQEDLRNIDLGKTNVSTSNTEETVCEEYSNVISLNGFVRRHQKLVKLAQELDAIFNKMIFVNLLFPTITICFFGFTARLAEGPLQIVGGYVTVIALILPIFNLCYYSQVLSEASVGLADAAYELLWYKNNKDIQRKICFIIQRCQKACSMTSLGYSPITLKTFTKVLSTTWSYFSLATTIYEDE

>CsasOR50

MANKQIDCFSTNIKFWKFLGIWPENVHSHYKYYSHLFLATFVYLYWILFSINFIYLPRQLDTWIGEMIFYFTDISCLSKVLTFRLMHDHIAKLLNMLESDIFQPATDEGLKIIADVKKFNVKYWKIMAVVSVTSHFTHIFSPLLAHLISHVKLELPVSRYSFIPEKVKEQVAYPLYFYQTIGMHFLMWYNINIDTFFLAIMMFAISQLEILNLKLRNITSAAKLPRESRADAQVDVQRNDEYYVHKLTECIIHYTEVSKFCELIQNIFSITLFIQFSVASCIICICLFRFTLPAPLDYYIFLATYTFIMVIQIMLPCWFGTRIMDKSYELSMAVYSCDWTARSRRFKTNLRLFVERTNRPLSIIGGLMFTLSLSTFTSIMNSAYSFFTLLRHMQSREG

>CsasOR51

MKWFQVADYQEIYIKIKRNMKDYFIFKNICKCIYYVGPGNFWYKPGEVREDNTKGYKAFSTVLFSVYSIVTILEFMAAIFGDFPADEQSDSVTFAVSHTIVMIKIFSVIRNKQVLKNLCRDMIRVCEPYEDPSKMSEKYRIVKINVIAYMISVYGSVLFYVFEGIRKLYAGRNFVTVVTYYPVSEDYSALANCFRVATTIVLCMMMLTMLLTVDSFTMINLIILKYKLITLRDYFYNLREKFEVINKADPRLAADDLANGLIEGVKMHRELLRLSKEILNVFETVITCQVCQSVGAAVSLLLQIALSNDLTFSASMKIIFFVIALFFLLALYLCNAGEITYQASLLSDAIFHCGWHIVPNPARHRRIGRVVLQAVALAQRPLVMKAYNILDLTYGTFIQVVRGTYSVFALIYARNK

>CsasOR52

MSTIMRYYIGNVFVSLRVALTALWMVGYWAPKQLTEWKKTGFFCYGVTWYMVQIGFVALSQIADLIKIWGNFSLMTATAFLLFTKVAVTVKIFNLILRNDTIREIVDECNQELESEGKEAGSAEGEVIISSEIETRNLSSSFAVLSMLTVCSWAAAAEKNQLPLRAWYPWDTTKSPAYELTFAYQIVIGSIAAAMNNGVDAVAIALIGQCRCRLQLIALALRNLCQGLEPGEYKLMSSEQDKILRSRLITCIKKHQAALQSAKRLQACFSLPFLAQFAASVVVICVSGYQLAFEAWKPFRLMAMVSYLLAMMVQVFLYCYQGHFLIEDSTYLVNAACESPWYTLSVSSRRLLVIMMIRAKTATKLTACGLFDLSFQTYMAILKASYTFFTMLQQVVVRE

>CsasGR1

MDNELHRFRMYNPNQNEEINRRDMFGKTDGKELDDREMKNFYGPEITEKDGELLDKHDSFYITTKSLLVLFQLTGVMPIMRVPKDAQTTKRTTFNWISKATFWAYFVWSLESIIVIKVGRERLSNFQQNSTKRFDEVIYNIIFLSILIPHFLLPIASWRHGHQVAIFKNMWTQYQLKYLKITGTPIVFPNLYSLTWGLCAFSWGLSIAVILSQHYLQDDFELWHSFAYYHIIAMLDGFCSLWYINCNAFGTASKGLAMNLHSALEAEHPSLKLAQYRHLWVDLSHMMQQLGRA

>CsasGR2

MFLLTMTSILSHAKWFGVSVKSCSVFFFWAIIVLILLTAIEVGAIWKVIKVWMGLDGRSDTLEGSLTIRLAGSIFYGNAFISLLLLWKLASSWKMLSIYWAIAEVNSGLKLPSDSKLRKRIVTVTNFIAIFGVAEHFLSITGNINFELQTSGYLQQYILKSHAFLIKPNDYNIWLALSIFIVSNLATVLWNFQDLVIILISMGLSSRYYRINSYLYDTVKTERRLRNVEKGSTTIYKQQQTWRRLREAYVRQAALVRMIDRELGALVLLSQINNFYFICLQLYNGLHRSTTSLSSHIYYMSSLGWLLFRAISVVLAAADVNISSKRALPYLYMCPRSAYNVEIKRLEQQLTHDFVALTGMGFFSLNRKMLLEVAGNILKYELVLIQYDF

>CsasGR3

MRATEFPNFNRNTKQRVCKYSYKPLKALLMLFLLDFRGQSAHNLCSILRSTVIVSAITFIQVYSFYFKITRMTAKVIASIRITDTVQMIFDYCQYLIDLFFVHKYGQDVSIEYSKQYDRIDGIMGMSYYPNLKQRMKKVILLFMFVWMTITTCDFVAWALAFGWFISLIYAVNFIFLLIKMTTILDLTYQVSNIEIRLQMIADAVQQYCVSTACVSDLIGDPTSNKKWLYNEGYLGKKLNRPEPLTVFTRSLDDPKWLNRCYLLLLEQCAFINHVYGVRILLNSLSLLIDMVRFINLGVRVLIGTQHVMYENTCFPIISSIMKAAACAFILVVLVYYCERAYRQGDRVISLIDHLLVTKCPKDALRSSMQELRNLVQSRPIDFHLAFFFRLNYALLISIASVVSTYTIILLQSPD

>CsasGR4

MYTEYISKDILEHEFMKCFTPIHILQSFLGSAQVDMKYNFVTDTSIYHKLYSLLWSIVCSVSLYEFIKHYYFKFYMKCFPLFCTCTIGISIQYVSYITNMLFIRCYKRDKNVKIYLMLQKIDHSMELIGNKTLNRTQYLLHLLCLFAMITPYSVGYGVHLYYNMERPLLNIFLGMGVTTVYLELLATGSFVFYLAVRLKAINNALSSNLILEKSIASNFIKSDLNRLLVCFKDILATFKAVSKIYSFPVS

>CsasGR5

MSSVFKISRIFGVAGSSSPSWKTWGVFVLAALMIIVIWAIWKVIKALSGIAIDIVGHRSVTARLAGTMFYSISTLSLILCWRLSCLWTNLSHCWVTVERSATIDLPPDGSLKKRMRTVIGIAILCSVCKFFHIILL

>CsasGR6

MIVKNKPQVKVIGCFETKAKANKNMRRMSFWIPSRNNKINVSKLKPAKIATFQRCLRTTLLWGQIFSLIPVSGITSDNAGNLKFVKTSWRSMYAILSIFGQFFMTLMTIVKVVYADTSLKSTTTVIFYGTTCITMIMFYQIGKSWPNLVQHISRTEDLDPSFDKKLTKKCKITCAVILILSLFEHILSLLSAFAGSLVCHSKKSTYEGFVFHFYPWVFNFIPYSPFTGAIIQFLHIQSTFIWNFSDLFVICMSYYLTSRLEHVNRKLIAAQGKYHPEVFWKTTREDYCRATALVRRVDDVISGVVFISFANNLFFICFQLFNTLEDGVRGKGECRSKDMLLGGYESPTYFMFSLVYLISRSIAVSLIASQVNTASAVPAPILYEVPSPVYCTEVQRFIEQVNGDNVALSGLQFFSVTKGLLLSVA

>CsasGR7

MLKKLKRNSTVKPETDLQLDMLKIFKPFYTLVSLYGLCPLSIKFSKSGNEISSIPKSIYFNIVYISCILIACHTFLAIHIHSVFTFETKESMTAALLTQMNYVLELFLLLLSCDITYICAFLNRYKYINIMKKVVAMWRALPYQDSNQILREFRYEVRMVVLGTLLIYNVIMQCINFSRHSNLWKMIMVLMTFDLYQSIQYAMVFFYYVFIMMLVTLLKNIRVNLNKLAMEKQKLDNYVKDYKLSTFVMP

>CsasGR8

MGLSSRYNRLNLYLARVFRREQENIGKCDVNTNISKWRRIREAYVQQALLVRRVDAVLGFMILLSCYGNFYFICLQLFLGITQGVTNSLPKRLYYIVSLSWLLTRTVSAVLAAAEVNVHSRNAVQYLRASDTGWFNVEMERLLVQLTKDNVALSGMGFFYLNRNLLLKMAGAVVTYELMLIQFDNKGYIDA

>CsasGR9

MFLFPEDNREVEYMGVDSVKPEGALGPPALLVSPETKIKTSYSIVGGAHAFILRISSIFGLAPLKFESRGNGFSVSLSSGMCVYSYILVTVLIICTIFGLVAEINVGVELSVRMTSRMSQVVSACDVLVVVVTAGAGVYGAPRRMKNIIEFMDKVAGVDISIGAQYSAMTERRLTGILLAILIFFTVLLGDDFCFYALQARKGDRHWDVVKNYIGFYLLWYVAMILELQFAFTALTLRARFQAVNDALALTGKHLSLPMEKSRESAPLNIFAIRVTPSAESQRASNVSLLMEPIPGRDQPVIIKRSVTGEPRLLVSPFEAIRRLSSLHGTLCSVVRAIDDSYGLPVIIILISTLLHLIVTPYFLIMELIVSTNRVHFLVLQFLWCLTHVLRMFIVVEPCHHTIVEGKRTAELVCRLMTSPSVGGAAAWLELFCRTLLLQAAAYTPLGMCTLDRPLVATVIGAVTTYLVILIQFQRYDN

>CsasGR10

MSTIYCEILKSVSFLSRYIILELLWNRMREIRRNLEKCIANTKMDNSTEISERIYNITTHVKCYKNLLDTLNCTNFSVKLTIFCNILIFILEFLIHSYTWLKNPRYFSSTETLFFVAFNVTLSGFMLLCVPVIFVELTAWEVNNIRTIISKQLMMCKDNWFRMKIQDCLTYMRLRPFKYTIWRLFSVDITMPYSILAFCITYLIVILQFSRIQ

>CsasGR11

MNKNKVTIVNNNMKATCVHSYTLLNSLLKLFIINFHTYSTIDKGRFCLLLSVLKVSFISIIHIYCFYFQLKYVNGTLLVSVELADSLQTVYDYCQYVVDLYVVHKYGMLFSLEYCRQYVSLDATLGIPHWTDAKKRFKVVIKSFILLWLITLVTDFIAWTIGYGWLITIHYGTSYVFLFIRLLTVIDLIDQVTNVGSRLQAVADYVQQYFLWAEFAPDFIGDSINHKKWLCFEKVNDTDRNLKQAHRLEVVGRKDAKWLSRCYLLLLEQCAIINRVYGVRVRYFCCRP

>CsasIR75q.2

MKMIYYLKILLLTISNTICYAETDFEPNIVHDVIQAMGRPSSVIATLCWSDQKKMQLVSTFSGSISKQAAMIVFVQQGQVEVQTYDDHHIVFVVDLTCPNITDHLVQNNRANHFRVPFRWLLIGYENSESSVPEELTVLDLLPDSEVIIVQHVGNSVYDINYVYKISVNGTWYTEAFGYWNKGGGFKKIIRPESTALRRLDLKGYTITICYVLTDDDSINHLTDEVNDHIDTITKVNFPTTNHLIDFLNASRKYIFVNTWGYKVNGTWNGMTGYLVREKVEIGGSPMFFTSERVSIVEYISSPTPTRSKFVFQQPKLSYENNLFLLSFQNSVWYSSLALVTIMFLAIFIATLWEWKKRGYNEKTDTDAHVGLLRPKMSDVIILIFGATCQQGSPVELKGSLGRIVMLILFIALMFLYTSYSANIVALLQSSSSGIKTLDDLLHSRLKFGVHDTVFNRYYFTTATEPTRKAIYETKVAPPGVKPRFMTMEEGVKKMQQGLFAFHMETGVGYKFVGKFFHESEKCGLKEIQYLQVIDPWLAVRKNTPYLEMFKIGTKRIQEHGLQSRENHLLYERRPKCSGGDGNFVSVSMVDCYPALLVLSYGSIMAVFFCVLEILYKRKHEILTRLSCHSDIE

>CsasIR76b

MDTGVALIISSICNATFCENVYDNPLVEQQLSHNQNVLRELAREVNGKRLKIASYSNFPLSWIDEGDNGTLVGKGVAFVLVNILSKKFNFTYDVVRPEKNFEVGGTRPEESLIGLVNNSMVDMAAAFLPTLMRYRQKVTFSTVLDEGTWVMMLKRPKESATGSGLLAPFKNHVWYMTLAAVLCYGPCITLLTRIRANIIKDDNYCINLSPSFWFVYGAFIKQGTTLAPDANTTRVLFATWWIFIILLSAFYTANLTAFLTLSRFTLDIEYAHDLYKKNYRWVAQEGGAVQFIIQDPNEELHYLQQMVQKGRAEFRSLSQSVDYLPLVVGGAVLVKERIAVDHLMYTDYLVKAKAGVVESDRCTYVVAPNAFISRIRAFAYPIGSKLNVLFDPVLAHLVQAGIVKFLANRDLPSTKICPLDLQSKDRQLRNSDLMMTYMIMITGLSAAAAVFIGEIVFKRYIHIKIRKNKGDKPKKKKTNKRIRFNEYDDAHPPPYDALFGKSSKYKTENSTKKIVNGREYWVVDTVNGDSRLIPVRTPSAFLYR

>CsasIR1

MKHFYVVLYFAFVNEITGDDYNHFLSELNHKIECPLRLTHTDQTWSLLLDLTTKLEQELLNCLIKGEKEDTRWLIISEFNERLINHLTSNEYNSKGYPITVIIKNLNYSVIDHPLIKHFLAKSQWSIIYFIIDTKEISYSCQNGAFENDTMLIIEDYFNEIWQKYQAVNVVMTFPDCPRYYIIFSEVKELIDTDNLYRRKITIVQADDQKNLQRNVMKKSRRVSEGYPLRANIFDRFPTSINDCSGMVYYVNPKRDFIKNFCGMDAMIMSDVVKHFKFHLSFPDLGEDGSMYGFLKNNKTLSGTLKHIVQKNIDVSFNSRFMANYMEKREYDFLLFVSVDALCLVTPRPDFVPLWHYPYNVYSMIIWALLVVVLSITGLFTWLIVKYVQKPTKKSFSSFVGYTIDCIITGLFGISMTRRFIITRAFCLGVSIILSALYQSHINYVFTTLVHYDSMKTIEEVYRSCTIYLSPSIADLLKPPSNDLQETILAAMVTEPPTNGSRLAFLLEHPNTASVERKADIILEIFKYHTDDRGFPKLYVIEECFRQYYLSYISRIGFLFNEQLATFIARLNEAGLPSQYYTWTRYALRLPKTSFNMGPEPRPFSKITLGEQIIPFYILFIGYVSSTIVFVVEIWQAQRKAHIKPIIE

>CsasIR41a

MNLSQIIEPIGTLLQIILDQYFITSFCVTIVSETPLDLKIPINFMYIKPRAENLADILLDASDKGCSDYVVLMNEAEIFMAAFEKVNHLGLVRRSDRKIVIAPIANGNNILNILSLKESEFVPNILVVAPEELIDGCQVYDLVSHNYVGLDVESNLPLYLDRWDSCTEKFEKKTHLFPHDMSNMMGKTLKVACFTYKPYVLLDLDITKSATGRDGIEMRMIEEFCSWVNCSIEIVRDDVNEWGDIYENNTGVGILGNIVEDRADVGISALYSWYEIYRVLDFSAAGVRTAISCIAPAPGLLDSWETPLLPFTWEMWLCIIFTYIYASLALSIANGWSIDRVFLTTFGMMITQSQFDAGTSWRIRSVTGWLLITGLILDNAYGGGLASVFTVPKYESSIDTVQDLIDHKMEWGATHDAWIFSLTLSSEEKVKQLISKFRVYSAEELQRKSFERSMAFSIEKLPAGYFAIGEYITKDAVPSYQLMLEDFYYEQCVVMLRKSSPYTAKISDFVGRLHDSGLMLAWETQVAINNLDFKVQLEVKLSRSIRDVENVQSLSFRNVLGIFFIYIAGVTVAILAFLAEKLTNNKKQKRANMVIKFT

>CsasIR8a

MWDMFQINMEIYFVLFIFLINLACVVSEISLRFVFIVEAQEMDLPQKIGRALKLSEEMRPEIRVSEDVVMLDRENDEESYRKLCAALSNGASMLIDLSWAPWQAAEQVSMDSGLPLIRTQLGSQQLLTALDSYLETRNATDAALLLESESDVDRTLYELLGQSNVRVWVHAGLTKDSARSLKIMRPEPSFYAVIGEGGFVADTYRRAVKEKLVRRTYRWNLVLTDYLPFDVAQLVLPTMILQIDPAECCRLINQRENCNCPGDVPRKQQILTALLQYIIETYYKLEQDLPLMINRVECDVVVVDFNGTRDRLYRQFAEDTAMSNDTIFYWNSERSGLFLRSGFVLSTYTPDEGQQTVATWSASEEFKLLPGITLEPLRLFFRVGTAPAVPWTMLKIDPDTGEPMFDEEERPIYEGYCVDLIDKLAETMGFDYEIVPPTTGGFGKKLPNGTWDGVVGDLTRGETDIAVSALTMTAEREEVIDFVAPYFEQTGILIVIRKPIRKTSLFKFMTVLRTEVWLSIVAALVLTGFMIWLLDKYSPYSARNNPEAYPYPCREFTLKESFWFALTSFTPQGGGEAPKALSGRTLVAAYWLFVVLMLATFTANLAAFLTVERMQTPVSSLEQLARQSRINYTVVEGSTVHQYFINMKFAEDTLYRVWKEITLNATSDQAQYRVWDYPIREQYGHILLAINASIPVPDAKTGFDQVNEHTEADFAFIHDSAEIKYEITRNCNLTEVGEVFAEQPYAIAVQQGSRLQEDLSRALLELQKERFLEQLASKYW

NETARQACPDADESEGITLESLGGVFIATLFGLGLAMITLAWEVFYYKRKERNKVQTIDNKNVEAFAEPKKNIEKKIADGVARIRKRKKLGKVDKVGKSVTIGDSFKPAAEKGVSYINVYPKGGFQP

>CsasIR21a

MILHRKNICLLIFYLELVINFCIIEVVRSEDVEYYPSQSVSLSSKKSKVFHSHRRKRYSRNGSKLEFRRFNNPAKETDVVKIKRSADPVFHGHPKTREELWNEHFLNLSNAFDQTPSLIKLLHNITMTYLDDCTPVILYDKQVKNQESYLFQNLFKDFPVSYVHGYINERNELMEPALLQSVKECIHFMVFLKDVSVIAKVLGKQSQSKVVIVARSSQWAVQEFLAGPLSRMFINLLVVAPSFKDDDDTSIEAPYILYTHKLYTDGLGASQPRVLNSWTHGKYSRDMNLFPVKMTAGYAGHRFLVATANQPPYVFRRIISDSDGGNPRVIWDGIEIRLLQLLASRNNFSIEFIEPREPNLGPGDAVAKEVAQGRADIGVAGMYMTDDRARDMDMTVGHSQDCAAFITLMSTALPRYRAILGPFHWHVWVALTFTYLIGIFPLAFSDKHSLRHLIHNSGEIENMFWYVFGTFTNCFTFVGKNSWSKTTKITTRLLIGWYWLFTIIITSCYTGSIIAFVTLPVFPETVDSIQQLLMGFYRVGTLDRGGWERWFFNSSDPKTNKLLRKIELVPNVAAGIRNTTKAFFLALRILGIACGIRIHCTS

>CsasIR68

MLKAIVFVFFTLGQLNGANANISNMLKEVEHTKDLQYLLIDLVNVMTRHYDVTCIAVLCDTTYLNVFEGTLFRWTIAVPILMIVVEEYEDLLSPNFDTLEALKEAKKNGCNAYVVLLANGLQASRLLRFGDRHRILDTRAKYAMLHDVRLFHSDLHYLWKRIVNVIFIKHHQKISGVLKSKAWFDLSTVPFPNPIKGIFVPRRVDIWDNGRFHYNRHLFADKTKNLNYEVLNVVYLDHVPSVIVQKMNESNKVGGVEIEILNTLSQKMNFDPKLYQPSNVELHKWGQKQANGSFSGLLGEMVNGKADMALGNLQYTPYHLELTDLSIPYTTQCWTFLTPEALTDNSWKTLILPFKLYMWITVLLVLLITGAIFYGLAKYYMYLEKFIKDQRKFNKSVSNEKQDEEDAKPVGLFLFGEIINSILYTYGMLLVVSLPKLPSGWSIRLLTGWYWLYCILLVVSYRASMTAILANPAPRVTIDTLKELVDSKLTCGGWGTQTKAFFEGSLDEIGQKIGERFEAINDPFDAANRVAQGVYAYYDDSDFLKFLSVKRKNTFLMEDMSNSTANATEIMALKTANERNLHIMSNCVVNIPISIGFHKNSPLKPLADIYLRRIVEVGLVEKWLNDAMQSIRSLGSNIDEVKALMNLQKLYGAFIALAIGYTLSLFCLIGELLYWNCVVKKDPNYDKYALDLYYKNKRH

>CsasIR7d

MVGNNKFDPRYAEQTRQYLVLGTDLSNIRNMLDWLKMHKFDNTGKHIVVCGSSIQKDCDEVKAINLLWEYRITNILFMKFDMDSKIVTFTYNIHENKCGLSEPKQLRTLCIEEQNNYIPGNHVNLFFNKFRNLHRCPIIASTFEHKPYMMIKNGTPSGADGNLLLSLAEGLNATLKIITPTRGEGWGGLDDNNEWTGSMADVYYDLANLSMTSAGLTLARCTNFEMSKTYNYKTLIWVSSPAVMEPASLKLLH
