# Supplementary material for: Antennal transcriptome analysis of the chemosensory gene families in Carposina sasakii (Lepidoptera: Carposinidae)
Source: BMC Genomics. 2018 Jul 20;19:544. doi: 10.1186/s12864-018-4900-x (PMC6053724; doi:10.1186/s12864-018-4900-x)
Supplement: Supplementary file 2 — Amino acid sequences used in phylogenetic trees. (DOCX 196 kb) [file 12864_2018_4900_MOESM2_ESM.docx]

**OBP**

>GmolGOBP1

MSKNLVRLLLALTAVAVAQATQEVLKDVTLGFGEALEHCRESTGLTTEKMEEFFHFWSDDFKFELREVGCAIQCMSKYFNLLTDGERMHHENTDKFIKSFPNGEVLAKQMVTLIHTCEQQFDDMEDHCWRILRIAECFKTGCQERGIAPSMELIMAEFIMEADA

>GmolGOBP2

MCRRQVHIVIKMALYWMVTVLLVVGGRMVDGTAEVMSHVTAHFGKALEQCREESQLSPEVLDEFHNFWREDFEVVHRELGCAIICMSNKFSLLQDDARMHHDNMHDYILSFPKGDVLSAKMVELIHNCEKQYDDISDDCSRVVKVAACFKVGATQAGIAPEVAMIEAVLEKY

>GmolPBP1

MCKQIKLILVAFMCLSLSKLVDSSQQVIKDMGENFGKALDVCKQEMDLPDSIYMDFMNFWKEDYELTNRFTGCAIMCLSKKLELVDPDLQLHHGNAKDFAMKHGADENLAADLVKIIHDCGNSVPPQEDQCMHVLEWAKCFKKEIHKRDLAPPMDVVVGEVLAEV

>GmolPBP2

MKKLTTGFATALDKCKTELNVQENVMQDFYNFWREDYELLNRDTGCVILCMALKFDLIDDDAKLHHKNAHEFAKTHGADDDLAKQLVAMIHDCEKQNSDADDCIRTLGIAKCFRTKIHGLKWAPSMETVLEEVMTEVKPS

>GmolPBP3

MKAITSGFLKVLEQCKQELNLQGHVISDLYHYWKEDYSLLNRDTGCAIICMSKKLDLIDASGKLHHGNTAEFAAKHGAASEVASKLVEILHACEKTHDAIEDDCMRALEIAKCFRTDINQLNWTPKMDVIITEVLTEM

>GmolOBP1

MAFYLYSVLVMAIVAACVNNCSAITEEQKEFIKEKLKANALACGSELGFAKEQLTQWKEQKTSDDSNKCFIACMFKKSGLLDDQGLYSEEKALEKVKTYVSEARQEELAKAAKTCSAVNEQSVSDGSAGCDRALLLFKCIQEQKELLGITLDFVH

>GmolOBP2

MKLGVTCSRDHMITPNEMIMMQKHKMPDSENARCLMACVYRKASYMDDKGMFDNAAADAMAEKDHGEDTKMIENSKKLFDHCKSVNDEPVKDGEKGCDRAALLFNCLTENAAKMGFKI

>GmolOBP3

MRWWRLGDIVIKMALILMVLALLLGAGEMEAASEQSITMSHVNAHFGKTLEMCREEANISREIMEEWHHVWDDNFEIVHREIGCVLICMSHKFSLLQDDHRMHHNSMHDYIRSFENGDNLSAMVVELFHGCEKQFEDIADDCSRVTKVMACFKVAAKEADIAPDVALIEAVLEKY

>GmolOBP4

MNRQAFQSMCFLELLICFLLFNVTYAMTRQQLKNSGKMMKKSCIPKNDVTEEQVGSIEQGKFIEERNVMCYIACIYTMTQVIKNNKINHDAVTKQIDTMFPADMRVAVKATVDKCKDVSKKYKDICEASYWTAKCMYDADPANFIFP

>GmolOBP5

MSFNNFVVFIAVCLSYSVQADSVANMKMKYLQYMLTCAEQHSVATTDLKEITQQKMLKDDNVKCMFACVFKMTGMTDDEGNLSVEGIKQVTELVYANNPEKKAMSEDFIKACKHVNDEELTGEKKECQRAALIFKCSVENSASR

>GmolOBP6

MLHSLSWKGEASLFNKMLRISFLLLVAVCMVALRKVHAESDEEKEIHEALVPIIAECSKEHGAKVEEVMESKKNKDYEAIDGCLIECVYKKMGMMDDEGAFIVEKLVENAKQFMKEADSEKIEEIVKHCTSETEKTPEDKCGKSKMLLACMMEQKEALHL

>GmolOBP7

MPRTTKGVVRCRAYTPPTSKADMADAIKAIYLLADKAMTEDQKNIIKQHFHEIGMKCIKDNPITAEDISMLKDKKLATGPNVPCFLACMLKDVGVMDNNGMLSKESALELAKKVFNDAEELKAIENYLHSCSHVNTESVSDGDKGCERAMLSHKCMLENAAQFGFDL

>GmolOBP8

MVARVALCSAVVALYVIGVQACTEGLDAETAELAKMLRDNCGEETEVDVDLINQVNAGADLMPDPKLKCYIKCVMETAGMLDNGDVDVEATLELLPEPMRTKNEHIVRTCGSKRGADDCETAYNTQSCWQKMNKAEFCLI

>GmolOBP9

MTDEEVKIQFTKFVMKCHKDHPVEMTELLALQSLKVPTSKEAKCLLACAYMTEGTMNNKGLYDLEHAYKVAEETQKGDDKRTANAKKLADACSKVNDMPVSDGDKGCDRATLIFKCLIEHAPKLGFKL

>GmolOBP10

MMFIFIVKFLILITFCNAMTMKQIKATGKTIRKTCQPKNNVSDEKIDPLGKGEFIAEKEVMCYMACVMKMAGTVKNGKLSYDAAIKQADMLLPEEIKEPAKAALTACKKVPDAYKDICEAAFHVTKCVYNQNPDIFYFP

>GmolOBP11

MTRCGLSCAIAAFLVLGVSCMDDEMAELMKMLRDNCGEETGVDLSLVEKVNQGADLMPDPKLKCYTKCIMETAGMFSEGEVDVEAVVALLPEDMKTKNEKNIRACGTQKGGDDCETAFLTQVCWQKANKADYFLV

>GmolOBP12

MERQTWIFVMVFAFLANGSDALTKAQMKKSAAAFKKKCMAKENVTEDMIGDIEKGKFIEDKGVMCYIACIYQMANVVKNNKLSYEASIKQIDIMYPPDLKDGAKKSVEACKDVSKKYKDLCEASYWTAKCIYEHDPKNFFFA

>GmolOBP13

MSRSLPVLCALVALAYGAKETPVFSDEIKEIIQHVHNECVGKTGVAEEDIANCENGIFKEDTKLKCYMFCLLEEASLVDENDTVDYEMMVSLIPEQYYDRVSKMIFGCKHLDTPDKDKCQRAFDVHKCSYSKDPDFYFLF

>GmolOBP14

MRYITALCLIFALECTCEVVIQLEPEKTAKVIESAVKCTGKYGLDLEVLQRLRNKERTKDEKFLKFLYCTLDDLKVVKKNGYYIEEEALKFVPKQHKALIKKALEECNKEPGKNRIDVLYNVSRCLEDKSNVRVTI

>GmolOBP15

MSKSLILVTTVVLLLSLSLVQCAEKENKDVKPKVEEPDVGQDNMNDMDVMGALSDCNETFRIDMSYLLALNESGSFPDETDRTPKCYVRCVLEMVDVASADGQFDPERAEDALMGIRGVRALSNVKEVAVTCSDRQETCKCERSYQFIKCLMEMEIKMAEKS

>GmolOBP16

MLGAFIILTFLPVFISCTGDGNIRILEEEIAEALRSCLVPGNASKETRQRRSEEYGYDEQYTDRTPRIDNNSKPETNQYGHERRNTSMREQIHVLNATDFDYGGYGAGNGGEKLVNTVPRPAINGEYNNMTAANRTRRSEPLLNKIDSDQCLSQCVFANLQVVDSRGIPRESELWARIQASVTSQQSRAALHDQTRACFQELQSEAEDNGCSYSNKLERCLMLRISDRKPNASGPQTNGQH

>GmolOBP17

MKYRALILVLVFVCLDTTFGMTREQLKKTLTMTKKQCMPKTGVTEEKVGRIEQGVFYEEKNVMCYVACIYKSIQVVSIDHQIDHSIIECTYGVVW

>GmolOBP18

MSECLKEVQAKDKRAVKRLSPKTESPVNGECLIACVLKRSQVIHNGKIVKANLISLVSKFYAKDTKVMKKLDKNLD

>GmolOBP19

ECSNPKLPGPCNDIQCVFEKSGFLVDKNTLNKEAYRTHLKKWSEANSGWSQAVERAIADCVDKDLRQYLDYSCKAYDVFTCTGIAMLKKCPDSAWKC

>GmolOBP20

MEWLMAVCLLAVGVQAEFPTKEFLDTLKPVVAKCEASTGVDKALVDDFSKGTMVEDEKLKCYMKCIFLEFQVLDETTGHFRYEKMLGALPQEMKSIAYDMGRNCIHFKGEGGANLCQVSYDLHRC

>GmolOBP21

YYSAKMKFLLVLAAFVVVTCANNVHLSKSQKDKVQQYTVECMKQSGVKPEVLAEAKKQNFKDDEALKKFMLCFFQKSGIMDRDGRLNVDVALSKLPQGADT

>GmolOBP22

MMEQKEAAISGVQKTLIQAQFVTRGLSCIKSNPLSLEDINSLKMLKLAEGGNSKCFLACLFKNIGILDNSGAVSASSTRMSAKQVFANDEESLNKVEGLVKECERVNAENVEDGDKGCDRAAL

>EposABPX1

MSGHAAFCCAMVAALVLGAHCMDEEMAELAKMLHDNCGEETGADLSLVDKVNAGADLMPDPKLKCYIKCIMETAGMLTDGEVDVEAVIALLPEDMAAKNGPALRKCGTQKGADDCDTAFLTQVCWQTANKAEYFLI

>EposABPX2

MTKSTKYAFAAVLLFLVLSPAQAASTKEAESPKNDETKEKSEDSDNVDIMSAMMDCNETFRIEMSYLLALNESGSFPDETDRTPKCYIRCVLEMVEIASADGQFDASRAEPALLSIRGVRVLSDVPETAAACAADRNESCKCERSYQFIKCLMEMEIKMAEKS

>EposABPX3

MGGKDWLFLMVVVVAAHGINALTRQQLKKSSQMFKKKCMSKIDVTEDMVGDIDKGKFIEDKDVMCYIACIYQMAQVIKNNKLSYEASMKQVDLMYPADMKDAVKKSIDNCKDVSKKYKDLCEASFYTAKCIYEDDPKNFVFA

>EposABPX4

MYFIQVSLVFMIFFVWDTSAMNRQQLKNSGKLLKKTCMPKHDVTEEQVGKIDKGVFLEERNVMCYISCIYSVGGAVKNNKIVHEAMIKQVDMMFPAEMKDPVKAAMENCRGVAKKYKDICEACFYTAKCLYETDPANFMFP

>EposABPX5

MVTFRMNIITIGLVIAICCHMACGMSRAQLKKTMTVMKKQCMPKVGVAEDKVAKIEQGVFPEERNVMCYVACIYKTIQVVKNNKLNKELISKQIDIMYPPEMKAAVKKYVAKCVDIQDNYEDECERVFYATKCLYEDDPPNFIFP

>EposABPX6

MRAFEVCFFTGFICFLLFNVSHAMTRQQLKNSGKLMKKSCMPKNDVTEEQVGQIEQGKFIEERNVMCYVACVYTMTQVVKNNKLSYEAVIKQVDMMFPPEMRDAVKAAAEKCKDISKKYKDICEASYWTAKCMYEFDPKNFVFP

>EposOBP1

MYFKFCFLAFVVLCLKSSMGDSSEEYKLKYVNYILECSKEFPITSEDILKLRNKEVPTAENVKCLLACGYKKSGMMDADGKLSPEGIQKISDEVFADQPELKQRSEDFTNACKFVNDEDIAGDKKDCERAAMIFKCSVERAAEFDFTT

>EposOBP2

MSLEKMVLELEAISDEKKAIIRQHFEQLGMECMKDNPITSDDIASLRAKKVPSGPNAPCFLACVLKKSGVMDESGMLQKENILEKARKVFDDEEELKSIEGYLHSCSHINSEAVSDGEKGCERAVLSIKCMIDNAAQFGFDI

>EposOBP2b

MAKISVFVCSLYVLLMTSYSMAISDEKKAIIRQHFEQLGMECMKDNPITSDDIASLRAKKVPSGPNAPCFLACVLKKSGVMDESGMLQKENILEKARKVFDDEEELKSIEGYLHSCSHINSEAVSDGEKGCERAVLSIKCMIDNAAQFGFDI

>EposOBP2cMIETYRYFQAISDEKKAIIRQHFEQLGMECMKDNPITSDDIASLRAKKVPSGPNAPCFLACVLKKSGVMDESGMLQKENILEKARKVFDDEDELKSIEGYLHSCSHINSEAVSDGEKGCERAVLSIKCMIDNAAQFGFDI

>EposOBP3

MSKFFQFLLCCCVIGVSYGKTEAEVKAYFMKLGIQCSRDWNITPDEMVMMQKHKLPDSENARCLMACVYRKAEWMDDKGMFDVAAAEAMVVKTHEEDTTMIENSKKLFEVCKAVNDETVKDGEKGCDRAAHLFKCLTENASKMGFKI

>EposOBP4

MTDEEIKIEFTKYVMKCQKDHPVEISEIMALQSFVVPKNKDTKCLLACAYRAEGSMTAAGLYDLEHGYKVAELTKNGDEKRFANAKKLADICAKVNDEAVSDGEKGCDRAALMFKCVTVNAPKLGFKL

>EposOBP5

MYSSFITNHRSFQALSTIQKTLVQAQFLSKGLICVKNNPLTLEDINTFKMLKMPEGDHAKCFAACLFKNIGILDDMGKLTSSGARQSAKQVFANDESSLSKVENIVQECAKVNDEEVKDGDKGCERAALAFACLTQVGPKYGLDLQF

>EposOBP6

MAQIRVLILVAVVCAVMKKSHAVTEEEHKEMHELMMPIVVECSEQHGSKPDDIIAVKESKDVDGMDSCLIDCVFKKIGIMNDDGMFVVEKFTENMNKIMKKDGDSAKIDDLAKHCASVNDKDSDDKCGRSKALLACLMEQKDAVRF

>EposOBP7

MVLFVILKFLLLTAFCEAMTMKQIRNTGKMIRKSCQPKNNVEDEKIDPIKDGVFIEEKEVMCYMACVMKMANTVKNGKLNFETALKQADMLLPEEIKEPAKAALTACRKVPEQYKDICEASFHVTKCIYNQNPGIFYFP

>EposOBP8

MTTCSDFAKMAFSCNRYRLIVVILLSLIMWQLFSMFPGDSAKTSTKLTQSGIVELEDDKFNIHKQDKVKVRVYYEALCPDSKHFFVRHLWPVTEKLSEFLEIALIPYGKATTKEENGQYSFHCQHGETECYANKIHACSIETIGNMTTSVKFTECMISDNSDPDEALTRCAKSMNIDMEPISTCATGELGSALLKKYGDDTQIINPYFIPTITLNGSKPKQTLPAILKNFLLEVCKRIAMPLPPPCL

>EposOBP9

MKLLLLVCAAALVCGTLAKNKKSDDNKVKIAVYYESLCPDSKRFITSQLAPVWRDFRGAVKVKLVPYGKATHDKVNGKWQFTCQHGADECYGNKIQSCILKDRSLQDTDKMELVICLMSQASPDKSLDTCLEQIKKQSESDKLKRCAGGDQGDNLHAANGDKTDAVQRPLSFVPTVVVNERFDQAIQDEAVNNLKAVVCRLAPTKPAVC

>EposOBP10

MVRKLSALICCFCVFGISLSDSAISAESETRCRNPPTAPQKIERVITLCQDEIKLSILREALDVIKEEHTMPAQKRRNKRDVPFTHDEKRIAGCLLQCVYRKVKAVDGYGFPTLEGLVGLYSDGVNERGYFMAVLEASRECLMKNHDLFSRTVPMDNGRNCDVSFDIFECISDRIGDYCGNSGL

>EposOBP11

MRYVAVFCFIFVLECASEMVLQLDNAKTPKVFESAIKCTSEQGFDLSILERFRSSETKKDEKFLKLFYCALDDLKIVKKDGHFIENEAVKFVPKKQREDLKKALEECNKETGIDRLDVLYNVSKCLKEKKGLRLAI

>EposOBP12

MKTFIVLAVCLVAAQALTDEQKEKLKKHRSECLTETKPDQQLVDKLKTGDFKTDNESIKKYVLCMMIKSELMTKDGKFKKDVALAKVPNGADKPLVEKVIDSCLSEKGNTPHQTAWNYVKCYHEKDPKHSILI

>EposOBP13aMRTFFILFVVLAVAYAKNVELSRAQKDKVQKNTVECIKQSGVKPELLAEAKKGNFKDDEGLKKFTLCFFQKAGIFNRDGKLNIETALSKLPEGADKAGVKKALEQCSTKKGRDAADSAYEVFKCYYRATPTHVVF

>EposOBP13b

MKTFFILLAVVAVAYAKNVELSRAQKDKVQKNTVECIKQSGVKPELLAEAKKGNFKDDEGLKKFTLCFFQKAGIFNRDGKLNIETALSKLPEGADRAGVKKALEQCSTKKGRDAADSAYEVFKCYYKATPTHVVF

>EposOBP14

MYRVLVLAVVLTVVLADSDDDKKKCHRLGHPGTMRCCKTEIPLPKTDMSDLKECMEIPHQPHSCEHDICIGKKRGYGKDDGTLDKAAFEKMFAEEFASSPTLVEAVKENCIGGDLTAYGPPDECELMKIKHCVHTQAINDCKEWNDEGPCAGVKDLVKECAKLMP

>EposOBP15

MYRGCVLFAVLPVIFAQGPPPPPPNLPPNFPPKCLGPPPAVEKPHECCQIPAFFPDEDFAACGFKKLDEDSPDRKPGPPDCSKQLCMLKKYNLIQDEAIDEKGIRQFLDDWSAKNEAFQPAVEVAKERCIGKNLFGPPQICEANKLVFCVSSTLFEQCPTWQGTEGCSTLKEHMDECKAFFPK

>EposOBP16

MFKVIVLLAVVVVAHADVAVTPPPIRCGELPQNIYKCLGNPRIVKSDVAAQCTKSTNDCDRMKCFFTKSGWMNGDAIDKTKVAAHFDQFAKDHPDWTPAVNHVKAACLAGPLPAQGWEVNCPTYDIMHCSLTGFFKNAQPSQWSTGAECTYPRQFAAACPICPESCFAPAIPYGSCNACRILPQTP

>EposOBP17

MFKLIAFVTFCAVVIQADPVPFKCGEYPWAILKCIGAPKVIKKEIADQCNSDPSECENNKCVYRKLGWMNEEEVIDQSKVAAYFDEVAKEYPLWADAVAVVKAECLVNSLPNLGTERNCLPYDTTQCAMARFIKSAKPSQWKTDAECEIPRQFVANCPICPKGCVAPFPEGLCNQCKV

>EposOBP18

MFRDTLPVLSALVALVYGGKDTPVFSDEIKEIIQHVHNECVSKTGTSEEDIANCENGIFKEDPKLKCYMFCLMEEASLVDEDDNVDYDMMVSLIPEQHFDRVSKMIFGCKYLDTPDKDKCQRAFDVHKCSYQKDPDFYFLF

>EposOBP19

MAISLYTVLVLAVFAASLDNCSALTDEKKAEIKEKLKVHINECGADYGITPEVLKEFKEQKKQPDDTNKCFFACMFKKIGLMDSQGLFSEEVAIQKLSQYAEESKLEKAKAAAKACTSVNQEAVSDGEAGCDRAKLLFTCIMEQKELYGFAV

>EposOBP20

MVYYILAIFIVAAPQVFADEEADIMSCLEMFKPDHIEEKCCEDMNMFDEDFESCKTKEGEWECENAKCVLEKAGILNGDTIDEKKAAAALEKHKKEYPANKAMFDRVQAQCMDGKYEEYPPKEACPLIKLQICSYIQSVVECQSWKQSDTCKKMSDHAKTCKTVLDNSGAK

>EposPBP1

MMNQKELVLFAVVCLSLYQAVEPSQDVVKDMSLNFRKGLDACKKELNLPDTINSDFNRFWNDDHVVTNRDTGCAIMCLSSKLELVSDTGLHHGNTLEYAKQHGADDTVAQQIVDLLHSCAQAVPDLEDPCLKVLEWAKCFKAEIHKLNWAPSAEVMAAEMLAEV

>EposPBP2

MATVSKWRMLVLTLCLTGVWQVESSADVMKKLTTGFATALEKCRDELNLPDAVMQDFFNFWREDYELVNRDMGCAIMCMATKFDLVTEEQKLHHGNAHEFAKSHGADDSMAKQLVTMLHECETQTSSISDDCSRTLEIAKCFRTKIHGLKWAPSMETILEEVMTEV

>EposPBP3

MARLSILVALVVLGVNISEIDSSEEVMKDLTSGFIKVLEECKKELNLSESIINDLYNYWKEDYSLLNRDVGCAIVCMSKKLELIDTSGKIHHGNAEDLAKKHGADSEVAAKLVAILHECEKTHDAIEDQCMKALEIAKCFRTNIHELNWAPKMDVVITEVLTEV

>EposGOBP1

MCQATRALVLAALLAAASATVDVMKDVTLGFGEALKQCRESSQLTEEKMEEFFHFWREDFKFEHRELGCAIQCMSKYFNLLTDGERMHHENTDKFIKSFPNGEVLSKQMVALIHACEQQHDAEPDHCWRILRVAECFKEGCQQRGIAPTMEMLMAEFIMESEA

>EposGOBP2

MASYWAVCVVLVAGSHLVAGTAEVMSHVTAHFGKALEQCREESGLSTAVLEEFQHFWRDDFEVVHRELGCAILCMSNKFSLMQDDARMHHENMHDYVKSFPQGEVLSAKMVELIHNCEKPYDDIKDDCERVVKVAACFKVDAKKAGIAPEVAMIEAVMEKY

>HarmPBP1

MEFHRSTMMSVRLALVVAAWLFIRVDASQDVIKNLSMNFAKPLEDCKKEMDLPDSVTTDFYNFWKEGYEFTNRQTGCAILCLSSKLELLDQELKLHHGKAQEFAKKHGADDAMAKQLVDLIHGCAQSTPDVADDPCMKTLNVAKCFKAKIHELNWAPSMELVVGEVLAEV

>HarmPBP2

MADSRWLFARVFCLVLMMGSAMSSKELLTKMTGGFTKVVDACKTELSVGDHIMQDMYNFWREEYQLVNRDLGCMIMCMTAKLDLIGDDQKMHHGKAEEFAKSHGADEALAKQLVGLIHGCETQHQAIEDHCSRALEIAKCFRTKIHELKWAPSMEVIMEEIMTAA

>HarmPBP3

MGSRHVFFALVVLAVSVRKAEPSKDAMQYITSGFVKVLEECKHELDLNEQILADLFHFWKLEYSLLGRDTGCAIICMSKKLDLLDANGRMHHGNAAEFAKKHGAGDEVASKIVTIIHECEKKHEQDGDECLRVLEVAKCFRTGIHELDWQPKVEVIVSEVLTEI

>HarmGOBP1

LLADINVMKDVTLGFGQALDKCREESQLTEEKMEEFFHFWRDDFKFEHRELGCAIQCMSRHFNLLTDSSRMLHDNAEKFIQSFPNGEVLARQMVELIHSCEKQFDHEDDHCWRILHVAECFKGSCVQRGIAPSMELMMTELIMEAESR

>HarmGOBP2

MTSKSCLLLVAMATLTGSVIGTAEVMSHVTAHFGKALEECREESGLSAEVLEEFQHFWREDFEVVHRELGCAIICMSNKFSLLQDDSRMHHVNMHDYVKSFPNGHVLSEKLVELIHNCEKKYDTMTDDCDRVVKVAACFKVDAKAAGIAPEVAMIEAVMEKY

>HarmOBP1

MSKFTFFVLCVVAVSLSKVYASDEDKAKLHEALKPLVEECMKDHEVSLDDLKAAKEAKSADGVKPCFLACVYKKAEVLNDKGEFDADHALEKLKEFVSDEDVLAKVAEVGNTCKAVNDKAVSDGDAGCERAALLTACFLEHKAEILV

>HarmOBP2

MMDRKRLCLLIIALFLAQGSDAMSRQQLKNSGKMLKKNCMNKNQVTEDQIGSIDKGKFVEDKKVMCYIACIFEMTNVVKNNKLNYDASIKQIDLMYPPDLKESAKAAVEKCKDVQKKYKDICEASYWTAKCMYDFKPEDFIFA

>HarmOBP3

MSKFTCFVLCVLAVSLGEVRSNALEKAAIRAAVYPLIVDCAKEHGVTLEQLKAAKASHSAEGINPCFQSCVYKKTGIFNDNGEYDVANAKTKLQKFVTDEDEYARIAEVGKTCASVNDKSVSDGAAGCERAALLTACFLEHRAQIII

>HarmOBP4

MSKLTCVVFAAVAVVFSNVNADDETRASFRQVLGPLVMECRNEFGITEDDLKKAQQERSPDALKPCFIACVFKKFGIITSAGKYDSDASISRIKDVVKNDDLLAKLKSVGEKCNSVNDASVSDGDAGCERAALLAKCFIENKSELSI

>HarmOBP5

MSKFTCLVLCVVAASLSQAYASEEEKAAFREAIKPIVEECSKEHGVSHDELKSAKDNQNADSIKPCFLGCVYKKAEVFNSKGEYDVDKALEKLKKFVSNDEAYAKFAEVGKKCASVNDKAVSDGDAGCERGALLTACFLEHKAEVPL

>HarmOBP6

MSKFTCLLLCVVAVSLSKVHATEEEKEAIRAAVRPIMQECGKEHGVTLDDLKAAKAAHSADGIKPCFQSCVYKKAGIFNDNGEYDIANAKTKLQKFVTNDEEYARIAEVGKMCASVNDKPVTDGAAGCDRAALLTACFLEHRAQIII

>HarmOBP7

MFRFGVLSFVVLLFCMESSYALSSEEELSIKEALHPFVVECAEEYGMTEEMFEEAKKKGSAEDIDPCFMSCFLKKTGFFDDSGKFDAEKSISFAKEHITSESAIKFLEAGAGECVKINDEDVSDGENGCDRAKLLFDCLTELKKKMSE

>HarmOBP8

MLLIEIVKFLTLVAMCEAMTMKQIRNTGKMMRKSCQPKNNVADEQIDPIAEGVFNEDKEVKCYMACIMKMANTIKNGKLNYEAAIKQADLLLPDDIKEPAKEAITACRKVADAYKDICDASFHITKCIYTQNPGIFYFP

>HarmOBP9

MCKFSVLFLYSAVMAVNIWSASCISEEDKAAIITAIAPLAQNCGSECGLDNDDFEKYKEDGSDMDPCFKACLMTQMGVLDKEGKYDGKGLHKAMEEADYPGDKDDAQKFLDELDRCFDAKGDNSGSDEEAKMKRADVLFRCMQDMKEK

>HarmOBP13

MFTGTLPLVVFLATFAYGGKEKPVFSDEIKEIIQTVHDECVAKTGVAEEDITNCENGIFKEDPKLKCYMFCLMEEASLVDDDDAVDYDMLVSLIPEEYVDRTTKMIFSCKHLDTPDKDKCQRAFEVHKCSYEKDPDLYFLF

>HarmOBP16

MFKSIVFCALIIVASHADVLKKRDSKGASLKPLSVCCDIPELGDPKNLEKCSNPKMPGPCDDIQCIFEASGFLIDRNTLNADAYKNHLMKWQEEHKPWKVAVDRAIEECANNQTRQYLDFPCKAYDVFTCTGIAMLKKCPEAAWKC

>HarmOBP17

MKTFVILAACVMLVQASGLTDEQKEKLKKHRSECLTETKVDEQLVNKLKGGDYKTESEPLKKYALCMMMKSELMTKDGKFKKDVALAKVPNAADKPTVEKLIDACLANKGNTPHQTAWNYVKCYHEKDPKHAIFL

>HarmOBP18

MKSFVVFCVLVAGAFAANVSLPPKQNEKANQIATECMKESGLKPEVLAEAKKGHISDDEHLKKFTFCFFKKAGIVSEDGKLNTEVALAKLPPGVDKAEAEKLLETCKGKTGKDVTDTVFEIFKCYHHGTKTHILLGF

>HarmOBP19

MEQCGIKRASGEGSEELEKIQPGPKVPCKEGICLMQKANLLQENNSVDYTKLRSFLDQWADTNAEFTDAILTAKKICAQDGGPAGPPVCEQDRIFFCLTSNILWNCNLRKLDGCDILQEHMDECRQYYVQDEPEE

>SlitPBP1

MANARWRFVFVVYALYLTSAVLGSQDLMAKMTKGFTRVVDDCKTELNVGDHIMQDMYNYWREDYQLINRDMGCMLLCMAKKLDLMDDQTMHHGKTEDFAKSHGADDDVAKKLVSVIHECEQQHTGIADDCMRVLEVAKCFRTKIHELKWAPSMEVIMEEVMTAV

>SlitPBP2

MSLRVALVVAASLLVVVQASQDVMKNLAINFAKPLDDCKKEMDLPDSVTTDFYNFWKEGYELTNRQTGCAILCLSSKLEILDQELNLHHGRAQEFAMKHGADEAMAKQIVDMIHTCAQSTPDVAADPCMKTLNVAKCFKLKVHELNWAPSVELIVGEVLAEV

>SlitPBP3

MGSRNVFVALVVLTVAMRETEPSKDPMKYIASGFVKVLEECKHELNMNDHLIADLFHYWKLEYTLLNRDTGCAIICMGKKLDLLDANGRMHHGNAQEFAKKHGAGDEVASQIVQIIHECEKKHERDDDECLRVLEVAKCFRTGIHELNWQPNVEVIVSEVLTEI

>SlitGOBP1

MLLLLALPLLAAVLPLRADVNVMKDVTLGFGQALDKCRQESQLTEEKMEEFFHFWRDDFKFEHRELGCAIQCMSRHFNLLTDSSRMHHENTEQFIQSFPNGEVLARQMVELIHACEKQHDHEDDHCWRILHVAECFKQACVQRGIAPSMEMMITEFIMEAEAR

>SlitGOBP2

MATVTSSVMGTAEVMSHVTAHFGKALEECREESGLSAEVLEEFQHFWRDDFEVVHRELGCAIICMSNKFSLLQDDSRMHHVNMHDYVKSFPNGHVLSEKLVGLIHNCEKQFDSMTDDCERVVKVAACFKVDAKAAGIAPEVAMIEAVMEKY

>SlitOBP1

MFKLCVFLALGFVACHGASNSNPGTPNANPGTYCGVTPDNIYRCLNNPRVVTPEVSTKCGSQFTECEKMTCIFRELKWSKRGAIDKAKVRAYFDQYETEHPEWAQAVQHVKAFCLASELRAQGVFLNCPAYDIMQCVLASFIKHASPSVWSTATDCAYPKAYAADCPVCPSDCYSPQIPYGSCNACYTQPRTV

>SlitOBP2

MVRKISGLLCCLCVFGISFSDSAISADSESRCRNPPTAPQKIERVITLCQDEIKLSILREALDVIKEEHTMPAQRRRDKREVPFTHDEKRIAGCLLQCVYRKVKAVDGYGFPTLEGLVGLYSDGVNERGYFMAVLEASRECLMKNHDKFSRTVPMDNGRNCDISFDIFECISDRIGEYCGTSGL

>SlitOBP3

MKSFVVICIVFVVGVCATEKGNKIASECIKESGVKSDVLAEAKKGNLGDDPAFKEFTYCFFKKVGIVGEDGKLNRDVAIAKLPSGVDKAEAEKLLDSCKSKTGKDAVETVYEIFKCYQHGTKSHIMFAS

>SlitOBP4

MKTLLVFAACILVAQALTDEQKEKLKKHRTECLTETKVDEQLVNKLKGGDYKMDNEALKKYALCMMMKSELMTKDGKFKKDVALAKVPNPADKPTVEKLIDACLANKGNTPHQTAWNYVKCYHEKDPKHAIFL

>SlitOBP5

MTKVLFAIVLTMVTFAVVLSASTKEAMTTTMSDQVNSIDVDVLAVMDMCNDSYRIDPTYLQALNESGSFIDETDKTPKCFIRCVFENVGIVSEDGKQFNPARAAVIFAGERNGKPMEDIADMTALCATDRQETCPCDRSYKFLRCLMSMEIERYEKS

>SlitOBP6

ESKFGEIVKRTVIATAHTCMDHVNATAKDLEHLRDEPPYPETSACIVKCLLEKVKYMRKQTN

>SlitOBP7

MFTEALPLFVILVAVTHGGKNKPVFSDEIKEIIQTVHDECVAKTGVAEEDITNCENGIFKEDAKLKCYMFCLLEEASLVDDDDTVDYDMLVSLIPDEYYERTTKMIFACKHLDTPDKDRCQRAFEVHKCSYEKDPDLYFLF

>SlitOBP8

MFGSKIVFSLFMIVSVCYGAVDIKKYFKVCDRNAIDVNDCMAEAVRQGIATMINGIDELGVPPIDPYLQKDFRLEYKNNQLAAKLTLKNIYVEGLKEAIVHDARLRADDDKFHLEVDLSGPRVSVRSDYYGEGQFNALKIVAYGQVNTTMTDLVYTWKLAGVPEKNGTETYIRIKDFYMRPDVGSLVTHFRNDNPESRELTDLGTRFANENWRMLYKEFLPYAQANWNRIGVRVANKLFLKVPYDQLFPTSS

>SlitOBP9

MCLVKYHVLVLCVILVGSYALNCRSSGGPKEAELKNIYKKCLKMQEGKNSSKGNSAQDWKEPRVQIQSNDNDGSGNRGRGNKNNRNDMNGGRDDRFGRDDYFNGREDFPQSDEYGGDMGQYNNNYYSTTQSSRRYKRERRPSNSGQRSQYNPNNHKISGYEDNFRSDERNTTDNNSSKETDNKSCALHCFLENLEMTGEDGMPDRYLVTHAITKDVKNEDLRDFLQESIEECFQILDNENTEDKCEFSKKLIDLSVRE

>SlitOBP10

MKEGNRYSHERRITNDSGDQLMVINATDDDYSGYGSGNMGEKLLTSVPRPATPSNNINKNNINRTKRNEPLLNRPDSDQCLSQCVFANLQVVDSKGIPREAELWNKVQSSVTSQQSRSALHDQIQACFQELQSEAEDNGCSYSNKLERCLMLRFSDRKVDGKGNAKKSSTEQTG

>SlitOBP11

MSKFTCLVLCVVAVSLNGVHATAEEKAAFIEAVKPYVQECSKEHGVTPEDIKSAKAAGNADGINSCFLSCVYKKAEVINEKGEYDVDKALEKLKKFVSNEDDYAKFANIGKKCASVNEKSVSDGEAGCERAALLTSCFLEHKSEISA

>SlitOBP12

MSVVRCSSFLVALFCFVSVNAMSGDEEAGIKEALRPFVQECADEFGITEEQFEEAKKKASAADIDPCFMSCFLKKAEFFDSQGKFDVDSTMAFAKEHLTSEPAMKFVEAVGDECVKINDEDVSDGDKGCDRAKLLFECIAETKKKME

>SlitOBP13

MITSSSLLVLTAVVQVLFAQQPVFESGPPEPWGPPQRPAHRRQFLPRIPKRCWVPPQRINVYNCCPIPTLYPDEDMQSCGFEKTSGNTDQPQKPVFRPEGTCKEGYCVMGKFDLLFANNSVDFVKFREYLDNWAESYPEFANAIRIAKQECAQDGGPEVPPICEPDKLFLCLTSTIFWNCKLRDGDGCAALQEHMNECKQYYTRQMEPTMKDIEVR

>SlitOBP14

MDQKRICLFVIAMFLASGSDAMSRQQLKNSGKMLKKNCMNKIGVTDDQVGSIDKGKFIEDRKVMCYIACIYELTNVIKNNKLNYEASIKQIDLMYPPDVKESAKAAVEKCKDVQKKYKDICEASFYAAKCMYEYKPEDFIFA

>SlitOBP15

MFNNCFVYSMTREQIKNSGKLIKKTCSAKNDLTEDEVKDVDKGKFIEKKDFMCYIACVYKMGQTVKGSTINHDMMLRQVDMMFPNDMKAPVKAAIEHCRPVAKNYKDLCEASYWTAKCIYDFDPANFMFP

>SlitOBP16

MLAEELKDCFDGSGPKDPMKCEIDLCIAKKKGFATDDGKLDIKKFEEVITKDVGSDKDLLDEIKTNCINGDLNNYGPPEFCDFIKIKHCVTLHMMNHCSEWSDDGNCKVVKELVGKCAKVI

>SlitOBP17

MRTFRLLCCILSIFFIFDQSYGMTRQQLKNSGKLMKKSCMPKNDVTEDEVGDIEKGKFIETRNVMCYIACVYTMSQVVKNNKLSYEAVIKQVDVMFPAEMRDAVKAAATHCKETTKKYKDLCESSYWTAKCMYDYDAQNFVFP

>SlitOBP18

MILXYTQKLTNMLLTKIVKFFILVATCEAMTMKQIKNTGKMMRKTCQPKNNVEDEKIDPLSDGVFIDEKEVKCYMACIMKMANTIKNGKLNYDAAMKQADLLFPDDIKEPAKEAITACRKVADAHKDICDASFHVTKCIYNHN

PSIFYFP

>SlitOBP19

MYSKICILLFISYTCLVTADSVSFIKKCKWDDGKCAKESGQNVIQKFAAGISEYNVGVSDPLHIEYVDASSPNMKLIVTDVVVTGLRNCEVKKIQRFEDSSKLIVKLLCAAELNGKYDMKGQLFVIPIEGNGGLYSKVPKIQINAEVDLNTKQGKDGKDHWIVKSWRHTFELKDKSTVKFENLFPDNEFLRTSTNELIAQNGNDVIIEIGANLIKAIVGKIVENIKKFFIAVPIEDLSL

>SlitOBP20

MWVQALVLTLATLVTLVAAAVEMDEDMAELARMVRDNCAGETGVDVALVEQVNAGAELMPDDKLKCYIKCTMETAGMMADGEVDIEAVLALLPPSLAEHNAPALRACGTQRGADHCDTAFRTQQCWQNANKADYFLI

>SlitOBP21

IHYLCAARAVHKIKNITNCENGIFKEDAKLKCYMFCLLEEASLVDDDDTVDYDMLVSLIPDEYYERTTKMIFACKHLDTPDKDRCQRAFEVHKCSYEKDPDLYFLILRREQLASRDDCVAISGIN**SNMP**

>SlitSNMP2

MLGKHSKLIFAVSMGFLVVAVIMAAWGFQKIVDKQIQKNVQLENNSMMFDKWLKLPMPLEFKVYIFNVTNVEDVNQGEKPILNEIGPYVYKQYRERTILGYGPNDTIKYMLRKRFEFDPEASGVLTEDDEVTVINFSYLAAVLTVHDMMPSFVGMVNKALEQFFPSLEDAFLRVKVRDLFFDGIYLNCDGDNAALGLVCGKIKSDTPPTMRPAEGANGFYFSMFSHMNRTETGPYHMIRGRENVYELGNIVSYKEQKVMPMWGDKYCGQINGSDSSIFPPIKEGNVPKKLYTFEPDICRSVYVDLVGKKEIFNISAYYYEISESAFAAKSANPNNKCFCRKNWSANHDGCLLMGLLNLMPCQGAPAIASLPHFFLGSEELLEYFGSGIMPDKEKHNTYVYIDPTSGVVLSGLKRLQFNIELRQIDTVPQLKRVPTGLFPMLWLEEGATIPASIQQELRDSHKLIGYVEVARWFLLTAAIIAVVTSAVAVARANALLSWPRNSNSVSFILGPSVTQVNKGN

>SlitSNMP1

MLLPKELKYAAIAGGVAIFGLIFGWVLFPTILKSQLKKEMALSKKTDVRQMWEKIPFPLDFKVYIFNYTNAEEVAKGAVPILKEIGPYHFDEWKEKVDVEDHEEDDTITYKRRDVFYLNPELTAPGLTGEEIVVIPHVFMLGMALTVQREKPAMLNMVGKAMNGIFDDPPDIFLRVKAMDILFRGMIINCARTEFASKATCTALKKEAVSGLVLEPNNQFRFSIFGTRNNTIDPHVITVKRGIKNVMDVGQVVAVDGQTEQTIWKDTCNEYQGTDGTVFPPFLTENDRLQSFSTDLCRSFKPWYQKKSSYRGIKTNRYVANIGNLAEDPELQCFCPQPDKCPPKGLMDLAPCIKAPMYASMPHFLDCDPALLSKVKGLNPDVNAHGIEIDFEPISGTPLVARQRIQFNIQLLKTDKLDLCKDLSGDIVPLFWIEEGLALNKTFVNMLKHQLFIPKRVVGVLRWWMVSFGSLGAVIGIVFHFRDHIMRLAVSGDSKVSKVTPEEVEEQKDISVIGPAQEPAKINI

>CsupSNMP2

MLAKHMKVFFLASLAALVLAVILAAWGFPRIVSKQIQKNVQLENSSVMFEKWRKLPMPLTFKIYVFNVTNAEDINSGAKPMLTEIGPYVYKEYRERTILGYGENDTVRYTLKKTFIFDAEESGPLTENDEVVVINFSYMAAILAVQEMMPSLTTVVNQALEEFFTDLKDPFMRIKVRDLFFDGIHVNCVGNHSALGLVCGQLKSDTPPTMRPTEDGTGYYFSMFSHMNRTESGPYDMVRGTEDIRELGHVVAYKGERSMSQWGDPYCGQLNGSDSSIFPPIDGGNVPQRLYIFEPEICRSMFATLVGKTTVFNMSAFHYSISSDVLAARSANPNNKCYCRKNWSANHDGCLLMGVMNLAPCQGAPAIASLPHFYLASEELLQYFASGINPDKEKHDTYLYLEPVTGVVLKGLRRFQFNIELRNIPEVPQLAKVPTGLFPLLWIEEGATLPDSVVKELQSSHKLLSYVEAARWILLVVAVIATVVSAVTLARSGVLPVCPRNSNSVSFILNPHPTVIDVNKVH

>CsupSNMP1

MQLPKHLKIGAGTAAAGVFGIIFGWVLFPAILKSQLKKEMALSKKTDVRGMWEKIPFALSFKVFLFNYTNVEEIQKGGVPIVKEIGPYHFDEWKEKLEVEDHEEDDTITYKKRDVFYFRPELSGPGLTGEEIITMPHILMVSIATVVNKEKPAMLNMIGKAFNGIFDGPQHVFMNVKALDIMFRGTIINCARTEFAPKAVCTAIKKEASGLIIEPNNQFRFSLFGMRNDTIDPHVITVKRGIKNVMDVGQVVAVDGNPEQSIWRDSCNMYEGTDGTVFPPFLTENDRLESFSTDMCRSFKALYQKKTSYKGIKTNRYVVTIGDLANDPDLQCFCEAPEKCPPKGTMDLMKCMNAPMYASLPHYLDCDPEVQKKVKGLNPDVNVHGIDIDFEPISGTPMVANQRMMFSLVLQQIDKLDLFKDLPGTMTPLFWIEEGIALNKTFVKMLKNQLFVPKRIVGALRWLLVAVGVCGVIVTGIIHYKGSILGFTLPRGSATVAKVNPETNQPKDISVIGNAQSPPKVDM

>SlitSNMP3

MCGVIGSVSTLVIGAILVIGSCIVGFLVVPNIVRNVIISEVVLNEDTIQMDRFEEIPFSLNFTVMIFNITNPETVLNGGVPFVTEVGPYIYRLYQTREILGIDGDIIRYKRHEHFVFDPVLSHPRTEEDILTIINVPYHAIIQVAETLYPNLMPLVNLAINGVFGKNNQPFVNITARELLFDGITLCKDTSLIATIVCNIIRNIAQGARNIEQLEDDSLVFSILDYKEQLPSEEYEVLRGLNDPADLGRILKYGGYNRFRHWAKNPEGGVTPCNQINGTDAGIYPPFVNREESIFAINTDICRSVELRYEYDTEYKGIPTYRYAANEWLLDNDEGCFCLNQTRGLNREDGCLLKGAMELYSCVGAFLVMSYPHFLFADNLYRNSVVGMWPDEDRHKIFVDIEPNTGTPIRGAKRAQFNIFSRPVNGVPVTQPFRTALVPILWVDESIVLPDEFVEELTGRLLHSLRLVDIFIPVIIAACGLVLVVGAGLTIRAFYVRKSVKKTESVPGDKTQPALETKPEPETQPEPLSQPEPRTENEVAK

>SexiSNMP3

MCGVIGSVTTLVLGAILVIGSCIVSFVVVPNIVRNVIIGEVVLKEDTIQMERFEEVPFSLNFTVKIFNVTNPQAVLNGGVPFVTEVGPYVYRLYQTREILGIDGDIMRYKRHEHFVFDPVLSYPRTEEDMLTIINVPYHAIIQVAETLYPNLMPLVNLAIDGVFGENNQPFVNITARQLLFSGITLCKNTGLIATIACNIIRDIAQGARNIEQLEDDSLVFSILDYKEKLPSEEYEVLRGLNDPADLARILKYGGYNRFRHWAKNPEGGVTPCNQINGTDAGIYPPFVSRDQSIYAINTDICRSVELRYEYDTEYKGIPTYRFAANEWLLDNDEGCFCLNQTRGLNREDGCLLKGAMELYTCVGAFLVMSYPHFLFADDLYRDGVVGMWPDEDIHKIFVDIEPNTGTPIRGAKRAQFNIFSRPVSGIPATQAFRTSLVPILWVDESIVLPDDFVEELTGRLLHNLRLVDILIPVMIAACGLVLVLGTGLTVRAFYVRKSIKKTESVPEPYTQPEPETQPQPRTEIQPAN

>SexiSNMP2

MLGKHSKLIFAVSMGFLVVAVIMAAWGFQKIVDKQIQSNVQLENNSMMFDKWLKLPMPLDFKVYVFNVTNVEDVNRGEKPILNEIGPYVYKQYRERTILGYGPNDTIKYMLRKRFEFDPVASGDLTEDDEVTVINFSYLAALLTVHDMMPSFVGMVNKALEQFFPSLEDAFLRVKVRDLFFDGIYLSCDGDNAALGLVCGKIKSDTPPTMRPAEGANGFYFSMFSHMNRSESGPYEMVRGRENVYELGNIVSYKGQKVMPMWGDKYCGQINGSDSSIFPPIKEGNVPKKLYTFEPDICRSVYVDLVGKKEIFNISAYYYEISESAFAAKSANPNNRCFCKKNWSANHDGCLLMGLLNLMPCQGAPAIASLPHFFLGSEELLEYFGSGIKPDKEKHNTYVYIDPTSGVVLSGLKRLQFNIELRQIDTVTQLKRVPTGLFPMLWLEEGATIPASIQQELRDSHKLLGYVEIARWFLLTVAIIAVVTSAVAVARANALLSWPRNSNSVSFILGPSVTHVNKGN

>OnubSNMP1

MQLQKPLKIGLGMMGAGLFGIIFGWVLFPVILKSQLKKEMALSKKTDVRAMWEKIPFALDFKVYMFNYTNVEEIMKGAAPIVKEIGPFHFDEWKEKVDIEDHDEDDTITYKKRDYFYFRPDKSGPGLTGEEVVVMPHLLMLSMATIVNNDKPAMLNMLGKAFNGIFDEPKDIFMRVKVLDLLFRGIIINCARTEFAPKAVCTALKKEGATGMTFEPNNQFRFSLFGMRNGTIDPHVVTVRRGIKNVMDVGKVIAIDGKTEQDVWRDKCNEFEGTDGTVFPPFLTEKDNLESFSGDLCRSFKPWYQKKTSYRGIKTNRYVANIGDFANDPELQCYCDSPDKCPPKGLMDLMKCMKAPMYASLPHYLDSDPQLLKDVKGLSPDANEHGIEIDFEPISGTPMVAKQRVQFNIILLKADKMDLIKDLPGTMTPLFWIEEGLALNKTFVKMLKNQLFIPKRIVSVVKWLLAGVGFVGLVGSVVYQFKGKMINFALSPSSAPVTKVNPEINQQNQPKDISIIGESQNPPKVDM

>OnubSNMP2

MLGKHTKLFFGVSLVALIVSVILAAWGFPKIVSKQIQKNIQIDNSSVMFEKWRKIPMPLTFNVYVFNVTNVEDVNNGAKPRLQQIGPYAYKEYRERTVLGYGDNDTVSYTLKKTFIFDQEASGLLSEDDEVTVIHFSYMAAILTVNDMMPSITGVVNGALEQFFTNLTDPFLRVKVKDLFFDGVYVNCAGNHSALGLVCGKLKADAPQTMRPAGDGNGFYFSMFSHMNRTESGPYEMIRGRENIKELGHIISYKGKSFMKNWGNDMYCGQLNGSDASIFPPIDENNVPEKLYTFEPEVCRSLYASLVGKSSIFNMSAYYYEISSDALASKSANPGNKCYCKKNWSANHDGCLIMGILNLMPCQDAPAIASLPHFYLASEELLEYFDGGISPDKEKHNTYIYLEPVTGVVLKGLRRLQFNIELRNIPMVPQLAKVPTGLFPLLWIEEGAELPDSIIQELRQSHTLLGYVEAVRWALLAIAIVATAISAIAVARSGLIPVWPRNANSVSFILSPHPNSDVNKVH**OR**

>HvirOR21

MDHFSGYYKSSKTTEFLINLNQFVFIFGLPNFWVQELDISDSFRKIVGYLNKYGNWSIFGLILAEYGAFFTQKNLNQRQSSDLVLFMISHSIITGFRVRICHQEVEIRNVMYKLGIALKEVHNDSEAEEQMIKRSKFFSWALILNCVISFLMYTIEAVLRVIRAGVTFTTVITVYPDVEDRSGLSNGVRVMFYIIWCIYLTRVFAVYTLVICLTIAMSHQFKNLTSYFYSLSSIFDDDQMTQAEKEQEYERAFRVGIKIHSDTLNCTGDIQKICRDVFSGQIIFNITLLIVLMYQMVNSARSLTNALTLVMVALSILLSTGFFMWNAGDITVEAKSLPTAMFSSGWEHCGRDSSVRVRKLIVIAMMQAQEPVVLTGLGIIALSYQSYVSIVKSSYSVFSVLY

>HvirOR20

EFKPFHETYKVITFTLCIAMIFPNPRTEKWRLISIPLLIATVAPVASMIFLDMYKCWTNNDIVNIIRHSTVVGPFLGGFFKMILMYHKRKEAKQILDEFDRDHFMFNDFSETYKDIARASIRNCQIYSERLWALLVTTCVMTFPVMAIVLNIYNFLFKSEPTKYMIHDLEKPFSESPEERFESPYFELLFAYMFYAAILYVVNFTGYDGFFGLCINHACLKMELYCKALEEAMVADREEVYGRVVAVIREQCRMFRYVDLVQETFNIWLGIIFIATMIQICTCLYHITEGYGFDIRYMIFVYGAVVHIYLPCRYAAKLKAMSMETSNRFYCCGWEKVDDERVRKMVLFMIARAQVPNEITAFNMLAFDMELFLSILQTSYSMFTLLRS

>HvirOR19

MKDRDILFKYCKVMFYIGSGNCWYKEDEIGNDRSILYRVCSASLMLLYSYMAIFELIAFAFGNFPEEEKRQALIGGAGHTVMLLKALFLTTKKLPIRSLNRKIVSICEDYEDSALMARKYKIMKINAISYLGLVNGGVLLYIIEGLRNMLNGSHFVTVVTYYPSFEDDSMLATIVRVFNTIIFIMIMLTIVISVDTYIVTYFIMYRYKFITLRKYFENLRNDFFTLIERKEVELATEKLANGLVEGIKMHSSLIRLKPEIDKAFATVSAIRVFESSSLAVCLLFEISPSDQRIPIEETVKTMIFIFALFFAMGLFLCNAGDITYQASQLTDAIFYCGWQSCPPRPRSAPNHNIRKMVLLAIMQAQRPPVMKAFKVLELNYATYIQLVRSTYSVFTLLCAQKT

>HvirOR18

MEMKVDVLPEKKYKGFNETFKLCAFSLAFAFLYPNRTTALRRCITITLIVTFCGGQLFWFITYTFKCLYTLDIYNFARNMTLAVVLVLFFIKTYYVIYATSKFAPLLDKISEDLLEANNLEEEFQVLYDDHIKIAKVGEISWLLIPTIMSALFPIYAGALMTIESIQTDDYERRMVHDMELLFVEDIQSETPFFQCMFAYNCVQCVVLVPNYCGFDGSFCIATTHLRLKLKLMTLKVYKAFKYSKSRQELRVRLYESIKDHQDALDFYVQLQNVYGPWLFAVFLLTSFMISFNLYQIYLLQRIDPKYTSFGVVGVLHIYLPCRYASDLTRVSEEIPDDLYLAEWEAWADPSITKMLMFMITRAQKEMIVTGMGLVVFNMEMFKSILQTSYSFFTLITA

>HvirOR17

MSLRSECARSVAPHVRVLRRVGFLRGAALPARPRAVRLALRGYHALALAATSTYVLQQAVYAYQERGDMEKLSQVMFLMLCHVTCVVKQIAFHVDADRIDRLIAGLDEPLLNQCEGERGALLRGTARGAARLLRTYVGCAVATCVLWIVFPILNRIQGISFEFPFWTGFSYDHNVVFSLVLLQSFYCTNLVAIGNTSMDAFMATILDQCKTQLRILRINFESLPERASALHMETGENYDTILDELFVDCLVHYNKITEMCAELHDVFTAPLLVQFAVGGWILCMAAYKIVSLEVLSIEFASITLFITCILIELFIFCYYGNEVTVESERVSQSLYSMEWRRARLAFRRSLVLVMERAKRPLRPAAGRVIPLSLDTFVKILKSSYSFYAVLRQTK

>HvirOR16

MGLRQFLFENEAVEGINTASDYLYIKILRFTLVIVNSWPRKEIGEPESPRLSAFAKYFYLFLTVLAAIGSIAYVAVHNRELTFLETGHMYIVVLMSLVDVSRVATLTMSTTYREVARDFLTKIHLFYYKDRSKHAMETHRAVHKISHLFTLWLVGQMLSGLSLFNLIPMYSNYAAGRYSGDVSKNSTFEHSLYYSYPFDTSTDIRGYSIACVIHWVLSYLCSTWFCMFDLFLSLMVFHLWGHFKILINTLNDFPRPSSKVEGAQFSDEELVDVAARLKDCIVYHREITLFTDRMSNVFGPMLFVYYSFHQASGCLLLLECSQMTAQALMRYVPLTIILTQQLIQLSVIFELVGSESDKLKHAVYGLPWECMDVKNRRVVLIFLANTQEPVHVKAMGVANVGVTSMAAILKTSMSYFTFLRSM

>HvirOR15

MTGFRDFVFNYQPKDGITNPVDYPYLIIARYLLTFISMWPKKSVVYHSARAELKARIWLWVQKFYHLLLCAVAFFGGVLYITLHKKSMTFYELGHLYISLLMMACTFSRITTLCFNDEYRVVAKDFVTKIHLFFYKNRSDYSMQIHKKVHMISHVFTLYLSGQMMLGLFLFNVTPMYNNYSAGKYKSGGLKNSTYEHSLYFSWPFNASTDMRGYIVSNILNWMLSYTCSSWFCVIDFFLSLMVFHIWGHFKILLHDLDHFPRPLNKVNSVIEDSITITNEMYSQTELDQVFDRLGKCIDYHREIVSFTDKMSEVFGPMLFAYYGFHQASGCLLLLECSQMTVAALVRYLPLTIILFQQLIQMSIIFELVGSVTDKLRDAVYGLPWEAMDTKNRKTVAFFLMNVQEPVHVKALGLAEVGVTSMTAILKTSMSYFAFLRSM

>HvirOR14

MTGIRDFFFNYEAKDGVTNPTEYPYMIMSRHLLTVITCWPKKPKEGLNARAKLRAKIWVIVQKIFHMSLCLLTTLGMAMYIGLHKKSMSFLELGHLYISLLMTVVIFSRITTLCLNPKYRAVSTEFLTKIHLFYYKDDSEFSMQIHKQVHKISHLFTLYLTGQMIAGLSLFNLTPMYNNFSAGKYKKGGLKNSTFEHSLYYSYPFNASSDVGGYIVSNICDWIISYLCSTWFCTLDLFLSIMVFHVWGHFKILLHDLDHFPRPANLTTFKLDNSNITLTSEKFSSIELGQVSEKLKKCIEYHRKIVSFTDEMSEVFGPMLFVYYGFHQTSGCLLLLECSQMTVAALVCYLPLTIMLFQQLIQLSIIFELVGSVSDKLKDAVYSLPWEAMDIKNKKTVAIFLMNVQEPVHVKALGLAEVGVTSMTAILKTSMSYFTFLRSK

>HvirOR13

MKILSDGSDLEGVEKVEDIFYINLARKSMWILDSWPKAFNASSKYRYFVLALNVATLIGGAIYLRNNTGVLSSFELGHTYITVFMNCITCSRCLMILSKDYNHVMTLFVQKIHLFHHKHKSDYAYLTHIFIHKISHFYTVYLLGLALNGLFLFNMIPFYNCYSRGMFRDVIPANATYDHAVFYSVPFDYTTKFKGYLAMTSFNVFISYTCTSYFCVVDLTISLVIFHLWGHMRLLTYHLANFKKPASVLESNDNNKDEIKDHSYTEEELKEVFSKLREYIQHHNLILEFSSEMSNAFGPALLAYMVFHQVSGCILLLECSQLDTKTLVRYGPLTIVIFQQLIQISVIFELLGSSNDKLIDGVYLVPWEYMDTKNRKLVFTMLRQSHRSINLTMMSMVTVGVQTMTAILKTSFSYFVMLKTVAEEE

>HvirOR12

MMEEEPLLIDKTVKKVEFLFRWTGINIKSGTKTRMDTIKSRAVYIINFIWLNSDLAGAVVWFFAGIANSIGFTELTYVAPCITLSFLGDLKSLYLIIREKNVDNLIQMLRDLEINERARPKSEEKDAIIKYEHNFVTTVISVLNVFYFVLLVAFALSPVTLVALKYYTTNELELLLPFLIVYPFNPYDIRYWPWVYLRQIWSEVVVVIDICTADYLFYTFCTYIRMQFRLLKHCIERVIPEDDGSGRLLNIEQVRAEFVQLIKWHQDLISSANMLETVYTRSTLFNFVSSSVLICLTGFNVVAISDVAFVVTFLSFLFMSLLQIFFLCFFGDLLMTASTEVSDAVYNCRWYLADTSFGKDLLLVQTRAQTPCKLTASDFSDVNLKAFMKILSTAWSYFALLQTLYGAPT

>HvirOR11

MHLAGNAVTGITGPMDYKYMKVLRFVLRIISGWPGKALGEKTLRIEGMGHAYYNTILSLVYLALGIAYLKKNFHRFDFLELGQLYIVLLMNMLSTSRAFTLCLSQKYREVAKIFIQKIHLFYFKEKSDYAMKIHVIVHKISFISAVYLSVLLFIAAVMFNLIPMYNNYSAGRYSSFDNLENTTYEQAISCLYPWNFETNFNGYLVATLSGWYGTMLCGSSVSMFDLFLCLMIFNLWGHFKILIHNLEHFPRPASEIVDAEGAERSGRIIGSEMYSQAELEQVAVLLRECIQYHMLIFDFTNNMSDAFGMALFIYYSFHQITGCLLLLECSQMTAAALTRYLPLTIIMFGELVLLSIIFETIGTMSEKLKDAVYKVPWEYMDTKNRRTLLIFLIKVQEPIHVKAGGLVDVGVTTMASILKTSFSYFAFLRTF

>HvirOR10

IDAVSEASYLLFTQASLCYKSTAFMVNKQSLLELLEIMDCEIFEPKSAEHEKILAAQARKIKRLCLFFLTSATTTCTLWAMIPLFDAASKRSFPFRIWMPVTPLKSPDYELGYLYQMVSIYISAFLFISVDSVAVSMIMFGCAQLEIIMDKIQKIKYVFESADSEEKRREIIKINNEFLVECIKQHQTVERFIQLCEDTYHANIFFQLTGTVAIICNIGLRISIVEPNSVQFFSMLNYMVTMLSQLFLYCWCGHELTIRSENLREWLYQCPWYEQDTKFKRALFIAMERMKKPIIFKAGHYISLSRPTFVAILRCSYSYFAVLNRVNTE

>HvirOR9

MVDQFQKCLKSVNLYLKFIGLHLESKDTTKTFIERSRSHRLYFAHFFSLNLEVVAQILWVLEAVITRKSFVEITRLIPCLILTLISDFKTLSLLYYARHNNEFIVTMKSLLLNQKQLEEKETRFREDLIDKHVLMLTSITKKISYLIGMGLLMFALAPAFIIIPHYFKTNEVKLEMPFIAYYPFNEFDSRIYPWVYLHQVWTACVAMIMVYGPDCFFFTCCTFIHIQFSLLNNDMERIVNEDTPRYDKTKFKELAVRHIELMRCVNLLEKIFSKSILFNALTSSVIICVTGFNVLVVDNIVMMASFTAFLLFGLMQIFLYCYYGDTIMRSSMQVSTSIYNSPWYNIRAADRKGFFIVIIRAQKPCELTANGFFKINLSAFTSILSTSWSYFALLKTMYHPE

>HvirOR8

CTRVIIFYKFKYVDFSNLLDSYCKIHNSFAIKIFFIMVFRQIDCFKINMKFLKFLAIWPGKDFTRRYKYYTVAFLTAYFIIFMILFTINLFFLPKQLDIFIENMVFYFTDSATLSKVMTIGFMRKKILQLFEMLESDIFQPDNAEGLAVIEKAKKFNKLYWNIILAVSFASCASNLFPPIIAHFILGTELVLPICNYGFLSEDFRQMFGVPLYLYQGSAMMFDMLYSVNIDTLFAGLMVLAIAQLDILGIKLRRVTDKEVLEETDSETSRQHRDNHKEAIKKINHCIIHYEKIHKYCSLVEDVFSITLFVQFGMASCIICICLMRFTMPAPLVYYLFLATYMFVMILQILVPCWFGQRIIDKSNLLAFSAYDCEWTSETRQFKSSMRIFIERAHKPLSITGGKMFCLSLVTFTSIMNTAYSFFTLLQNVKSRK

>HvirOR7

IEKLGVLFRFSGMNIKNKIVTPLDTIKYRWLYTLNFLVVFSAIIGSVYYVILGIKQGKNFIEVTSVAPCLTFSILSMIKSLYHLMYEEHIQELIELLRELELRENNREKCIEKEEIIASETGFLNKVINVLYVLNCSMIVVFDMTPMIMIAVKYYKTNEFEMLLPYLDVFSFIPYELKYWPFAYIHQIWSECVVLLDMAAADYLFFTCCTYIRVQFKLLQYDFERMIPDRGISKGKFYEENELRNKFTELLKWHQDIIYSSTILEIIYSKSTLFNFLSSSLVICLTGFNVTIVDDIVIIITFLTFLSMALMQVFFLCFFADLMMTASLEISNAVYNCRWYSANIKVGKQILFVQTRAQEPCKLTAAGFADVNLNAFMRVLSSAWSYFALLQTVYGGK

>HvirOR6

MNLRKFLFENEAVEGINSPADYLYTRILRFNLDFIRTWPRKELGEPENLAFTVFMQYFYLILNIVTVMGSTSYIVVRGSELSFIEAGLMYLIFLIGIVDTLTVVCLTFSEKFRVLAKDFLTKTHLFYYKDRSKHAMEIHKKIHLISHLFSLWILFQMLSGLSLFNIIPMYSNLAAGKYRKGGLQNSTFEHSLYYLYPFNTSTDITGYIIACILHWIISYLCSCWFCIINLFLSLLVFNLWGHFKILISTLNEFPRPSSKSVDTQESPYKYTEEELIEVAEKLKDCINYHREIKIFTNRMSDVFGPMLFIYYAFHQASGCLLLLECSQMTARALMRYLPLTIIMLQQLIQLSVIFELVGTESEKLKDAVYGVPWDCMDTKNRKVVMFFLMNVQEPVHVKAMGLANVGVTTMASILKTSLSYFTFLLSQTKEE

>HvirOR5

AYLDKVFLWSCLYGVFGSKRFISLIWSTLILGSLVIIEVLAIWKVIRALAGVARDMSGHRSVTARLAGTIFYSISILSLVLVSKLYYNWRTNIAGVWGKVERSVGVKIPVDRTLKCRMTFVAGLMTFFSIFEHAMSILSSVGLDCPPSLILKRYVLVSHGFIFMGQDYSEWFAMPLVIISTIATLLWNFQDQVIVLISMGLTSRYRRLNECLAKVCELEKQHKDSDKKIEAVKVYTWRKIREAYVKQAMLVRKIDVALGGIVILSCSCNFYFICLQMFLGITQGLSSDLLSLIYYVISLAWLCTRVISVVLAASSVNTHSKLALNHLYNYETHCYNVEVERLQDQLTKDYIALSGMGFFYLNKTILLQMAGAIVTYELVLIQFDDQGNDALNATKI

>HvirOR4

EVTAAPVPSESGSRPSRPTHCVVGGAHAFILRISSFFGLAPLRFESRSNGFTVSISGAMCVYSYILVTVLVICTLFGLVAEINVGVELSVRMSSRMSQVVSTCDVLVVVATAGAGVYGAPRRMRNMLKFMENIASVDTSIGGQYSRVTERKLCGIILAILIFSVLIADDFTFYALQAKKLDREWDVVTNYLGFYLLWFVVLILELQFAFTALSVRARFSAVNDALALTARQVSIPVEKPKTSSPLNIYAIRVAPVDSQRSANVSLLVDTMTGREHVVIIKRTASGEPRLIVSPCDAVRRLAALHGTLCDVVNSIDDSYGLPLVVILISTLLHLIVTPYFLIMEIIVSTNRIHFLVLQFLWCVTHMLRMIVVVEPGHYTIAEGKRTEGLVCRLMTSAPSTGVLPSRLEIFSRQLMLQSVSYAPMGMCTLHRPLIASVIGAVTTYLVILIQFQRYDN

>HvirOR3

LIDDGFFSFNLKYLYFVGLWPEKTLTANQKILYKLYEYFITFLTTTFIVLASIGTYQHKHDLVVVFCNVDKCLVVYNFFLKTIIFFIKRDKLRDLIDEIEMSGDKVTEERKKLMANYVMFITGMTAAVIGAFSLLALLEGTMSVEAWLPFDPMESLMNQILALEVLAFCVFPGLCRAFAMQGLVCSMIMYLCDQLTHLQTELRDLTYVKESEMAMRLKFKNAIRKHIRLMGYSGRMESIFKEYFLVQNLAVTVELCLNAVMVTVVGVHQITLLVSFVAYLLLALVNAYVYCYLGNELIIQSQGIALAAYESTWTSWPVDLQKDLLIVILAAQRPLKLSAGGMALLCIQTFSQALYNGYSIFAVLNDAVN

>HvirOR2

MMTKVKAQGLVSDLMPNIKLMQMAGHFLFNYHSENAGMSNLLRKIYASTHAILIFIHYACMGINMAKYSDEVNELTANTITVLFFAHTIIKLAFFALNSKSFYRTLAVWNQSNSHPLFTESDARYHQIALTKMRRLLYFICGMTVLSVISWVTLTFFGESVRMITNKETNETLTEVVPRLPLKAWYPFNAMSGTMYIVAFAFQVYWLLFSMAIANLMDVMFCSWLIFACEQLQHLKAIMKPLMELSASLDTYRPNTAELFRASSTEKEKIPDTVDMDIRGIYSTQQDFGMTLRGAGGRLQNFGQQNPNPNGLTPKQEMLARSAIKYWVERHKHVVRLVASIGDTYGTALLFHMLVSTITLTLLAYQATKINGINVYAFSTIGYLSYTLGQVFHFCIFGNRLIEESSSVMEAAYSCQWYDGSEEAKTFVQIVCQQCQKAMSISGAKFFTVSLDLFASVLGAVVTYFMVLVQLK

>HvirOR1

WPLFSTCSYIVMGPTSLKRNMFFWIPVKKNKVDVAKPKVKNITTFQDALRATLIIGQVFSLLPFVGVFTNVASNVKFIKTSWKCGYSLLSLIGQMFMAVLCVNKLAKSNVSLNGTSPVIFYVTTCVTMMLFFQVARRWPALVQHISKAEDMDPNFDCSLTRKCNITCAVVLILALLEHILSLLSAFAGASACYTGMDTYQGFVTHFYPWVFNYLPYSIVLGVITQFLHFQSTFIWNFSDLFVICMSYYLTSRLEQVNRKLLAAQGKYLPEIFWRATREDYCRVTQIVRKVDEVISGVVFISFANNLFFICLQLFNTLEDGLKGTGECTQLNSQSKLKKIVVSKSGPLGGHEAAAYFLFSLVYLLSRSVAVSLIASQVNSASSVPAPVLYDVPSPVYCVEVQRFLDQVNGDKVALSGLQFFSVTRGLLLTVAGTIVTYELVMFQFNSSTPSLNITSPTSATHIITTLAT

>CpomOrcoMMGKVKSQGLVSDLMPNIKLMQMSGHFLFNYTEETGGMSLLLRKIYAAMHAFLILLNFVCMGINMAQYSEEVNELTANTITVLFFAHTIIKLAFFAINSKSFYRTLAVWNQSNSHPLFTESDARYHQLSLDKSRRLLYFICGTTCLSVVSWVTLTFFGESVRLIADKESNDTLTEPAPRLPLKAWYPFDTMSGSMYIMAFVYQIYWLLFSMLIANLLDVMFCSWLIFACEQLQHLKAIMKPLMELSAALDTYRPNTAELFRASSTEKSEKVPEPTDIDIRGIYSTQQDFGMMLRGAGGRLQNFNSTNPNPNGLTQKQEMLARSAIKYWVERHKHVVRLVASIGDTYGTALLFHMLVSTITLTLLAYQATKIDGLNVYAFSTVGYLRYTLGQVFHFCIFGNRLIEESSSVMEAAYSCQWYDGSEEAKTFVQIVCQQCQKAMSISGAKFFTVSLDLFASVLGAVVTYFMVLVQLK*

>CpomOR1

MSLKSRVWEKLKKKFDDGEVDSPLKYTYVKQVSCLMSSVGAWPHRQFGRQKLHTLLSIYNLGLHGVCAGMFILGLLYWRQRRHNMSFFDSGHIFLCMLFDLLVLLRFMVTQTTKYQETIKAFLLEFHLFYFKDRSPYAAKVHTQVHTISGMFSFYVICQMAHGMALFVLMPCYSNLRKGMFGKNRPENSTFENSAYYYLPDACYTTLRGYWILLAFNAFSSYIITIGLFEFDLMISMMVFQIWGHLKILKNSLLTMPLPVNSKDGLYSPEENIKIKALLKEIIEHHTLIIKFVDGCSNTFSEYLFTFYLFMQFITCILLLEVSSFTADALGKYGPLTIGMHQQLIQVSILFEVLNTKSNELIDAMTQIPWEHMNTSNRRTVLFLICRIQIPVSLKAGGMVPVGVNTMQAVLKGSVTYYMMLKAFAAEG*

>CpomOR2a

MIIKQVYDVLKKRFDDGYVNTPLDFKYVAQLQFVLTTVGSWPYKQFGRNRLAATLSTYNAFLILVSTNLCVLDLIYIRVNRVKLSFFDLGHNILCLIFTFLYLQRLLTARTSKYQEVIKDYLLDFNLFYLKGRSPYAAKVQAQTHIISGMFTIYVMWQMVIGVSLFIFMPWFNNYNRGMFSENRPQNSTFEHSVYYYLPDAVYTTEEGYWILFVFNIPISYVTTIGLCVFDLLLILIVFQIWGHLRILKHNLQNIPLPENSIMYSVEENNNIRMLLKENILHHNIIIQFVDRCSDAFSEYLFAFYLFMQFITCILLLEVTTFTANSLAKYGPLTVVMHQQLIQVSILFEMLNTKSEQLIDAVYAIPWEHMDTKNRRTVLFFLHRIQTPVSLKAAKVVPVGVNTMSAILKTTFSYYMMLKALAGER*

>CpomOR2b

MILNQVYDVLKKRLDDGYVNSPLDFKYVASLQFVMTTIGSWPYKQFGRNRLAAILSTCNAFLILLGTVLCVLGLIYMRESVVKLSFFDLGHNILCLIFDTMYLQRLLTARTKKYQEVIKDYLLHFNLFYFKGRSPYAAKVHAQIHIISGMFTIYVMWQMFIGLLLFIFMPWFNNYNRGMFGENRPQNSTFEHSVYYYLPDDIDTTEEEYWLLFIFNMLLSYMTTICMCVFDLLLILIVFQIWGHLRILKHNLQNVPLPENSIMYSVEENNNIRMLLKENILHHNFIIQFVNRCSDAFSEYLFAFYLFMQFNTCILLLEVTTFTADSLAKYGALTVVMHQQLIQVSILFEMLNTKSEQLTDAVYAIPWEHMDTKNKRTVLFFLHRIQTPVSLKAAKVVPVGVNTMSAVLKTTFSYYMMLKALAGER*

>CpomOR2c

MNQVYEVLKTKFDDGYVDSPLDFMYVVRLQFLMNTVGSWPYKQFGRNRVAAILSTYNAFLILVGTTVCGLGLIYMRVNIVKLSFFDLGHNILCWLLEILYLQRLITARTNKYQETIKGYLLDFNLFFFKGRSPYAAKVHAQIHIISGMFTIYAMWQMVIGVSLFMFMPWFNNYSRGMFGENRPQNSTFEHSVYYYLSPGIYTTEEEYWLLFIFNFSLSYVTTIGMCVFDLLLILIVFQIWGHLRILKHNLENIPLPENSIMYSVEENNNIRMLLKENILHHNLIVKFVDRCSDAFSEYLFAFYMFMQLITCILLLEVTSFTADSLAKYGPLTVVMHQQLIQVSIMFEMLNTKSEQLIDAAYAIPWEHMDTKNRRTVLFFLHRIQTPVSLKAAKVVPVGVNTMFAVLKTTFSYYMMLKTLAGER

>CpomOR3

MFSYENEDSQITSPKDLSYIKQVATTLNRVASWPVLESKNKKYTFHVKWNIICFLFMSIIFILQIWYIRCNISTTSFVIMGHNYITLAMNIICLQRLTMPWMAEYRQVIKEFLENIHLFHLKDKSEYANKIYKKIEKICYIFTVFVHIQLYCGILLFNVTPTYKNFRAGMYGSNKPVNATYETAVYISLPFDYVTDIKGHIFVSFMGWSSTCIGSTSFCLWHLLLSLIVFHLWGNLKILEHNLDNFPKPANHQMKSIGIAWYTEEESKMISTLIVELVNHHRNIMGFISKTSSAYSFFLLLNFSFYQIVGCIILLECSTLDTEALGNYAPLTVVLFQQLIQISVVFEILGSQSEKIIDAVYDLPWECMELKERKLVLFFLQNVQEPINLKACGMIPVGVQTMAAILKACCSYFIMLRTVTSTEEMT*

>CpomOR4

MSPLNFTYVCILSSMLSSIGSWPHKQFGRRRLDFILSMYNILLIVVAIALPSLGATYIWNKRQTISFFDVGHLLLCMFLEFLFLQRLFMPWTAKYQVIIKEYLLKFHLFYFRNQTQYASKIHTQIHNISVIFTLLMACQMFCGVSLFTFMPWYNNYNNRMFILDRPANRTFEQAVYFYCFTEDVYTTIKGYWVLFVFNIPTTYNTSCVVAFDLLLSLIVFQIWGHLKILKHNVLSIFPKEGMYSPEENMKVREILKEIIEHHKFIIKFVNKCSDAFSEELFVFYLLMQVITCTSLLEVSALTADALAKYGPITLVVHQQLIQVSILFEMISSKSEQLIDAVYAMPWQSMDTSNRKTVMILLQRSQTPIALKAAKMVPVGLQTMAAVLKTSISYYMILNTVAGER*

>CpomOR5

MAPPRVFRSLKRWFDDSDAKHPLEFNYVRHLIFLLSFIGSWPHKQFGRDRLHFVLSIFNLFLIVVGITISIAAVGYIWSKRETISFYDMGHVILCILLETLFLQRLMTGRTEKYGEIVKDLLLNFHLFYFQSRSQYASKVYKQVQFASKIFTIYVTCHLITGFTLFSFMPWYNNYKNGMFSPDRPPNRTFEHSLYFYCFTDEVYTTLKGYWILFSFNVPTSINTSSGILTFDLLLSLIVFQILGHLMIMKHDLLSIPPTTDKYSPEENMRVKETLKGIIDHHNIIINFVDKCSDAFSEYLFMFYLLMQLLTIVVTVDLSTFTADALAKYGPLTIAIYQPLIQISILFEMISTQSEKLVDAIYEIPWECMDTSNRRTVMFFLLRAQTPVTLKAAKMVPVGVMTMTAVLKTTFSYYMLLNAVAESAEQ*

>CpomOR6a

MQTKRQTSEPKVFSLDYMKSLRFTLETIGQWPNRSLGDLSRRAVMLATYHKFLICTFCFTEILAFSYMIKHRKTIRFIDMGQIYTNLFLTGLFLQRASLPFQKNYKKCVKKFVLEFHLMHHEHLSEFAAKELRKVNKICKIATKVIYLQLACGMLAYNLSPLFRNYYEGMFAGELPENKSFVHSVDYLLPFDAYRSFKGYLVIFIWNWFPTYNIPTAMGIYDLLVFVMVFHMVGHMNILLNSLKEFPRPQEDGLPSTREYNEEIFGLLKNVIRHYQIIKDFMGDMTAAFDLTLCCYLAFHQVMCCLMLLECSTLEPEALVKYGMLAAVIFQQLIQTSVAFELIKSKSKRLGDEVYAVPWEYMNVKNRRIFVLFLRNVQYPLGLKAGGMVPVGVMSMSTIIRTSISYYIMLATFAD*

>CpomOR6b

MQTKRQTSEPKVFSLDYMKSLRFTLETIGQWPNRSLGDLSRRAVMLATYHKFLICTFCFTEILAFSYMIKHRKTIRFIDMGQIYTNLFLTGLFLQRASLPFQKNYKKCVKKFVLEFHLMHHEHLSEFAAKELRKVNKICKIATKVIYLQLACGMLAYNLSPLFRNYYEGMFAGELPENKSFVHSVDYLLPFDAYRSFKGYLVIFIWNWFPTYNIPTAMGIYDLLVFVMVFHMVGHMNILLNSLKEFPRPQEDGLPSTREYNEEIFGLLKNVIRHYQIIKDFMGDMTAAFDLTLCCYLAFHQVMCCLMLLECSTLEPEALVKYGMLAAVIFQQLIQTSVAFELIKSKSKRLGDEVYAVPWEYMNVKNRRIFVLFLRNVQYPLGLKAGGMVPVGVMSMSTVILYMLAYDGWLST*

>CpomOR7

MTTQTRDRALDLDYMRVIRLYLDTIAHWPNEKFGPKTMRTRILSIYHISILTMLVAVVAAEILALFFVRGRMPTHGFIDLGQDYLSILIGCVMIPRLTLILQEKYCSHIKTFVSKFHLLEHKHESGFAAKEYQKVNKICRIATMIILLECLLGQMMFNVVPLYVNIQAGLFTSRDNRPQNVTFVHSLNYYFIIDQYNDAIGYGIASFINAYVSYMCGVEFCGIDLLIYIMVFHILGHFNILVDKMRNFPRPVNFPDESQKYSERQYNEEALKVLKNLIQHDQLIKEFMNNTSKTFSITLCICLLFHQVSGCISLLEISPMTAEALTRYGPLILVLFNQLIQMSVIFELISSKSNKLSDEVYALPWELMDAKNRKTMLLFLVNVQRPRGLKAGGLVSVGVLTMAQIIKNSVSYFLMLRTLGNF*

>CpomOR8

MLQKMDTGATDVFDVAYMRMIRFSLSSIAQWPYNSFGRKSIRTRIMSVYHYIMISVSTFLEISCVFYVRNNQDKEFIVLGHDYFTLLMGVVIIQRMTLSFQKRYCLLVKNFVSKFHLVNHQYKCEFAAMELRRITRICNIAAVIIHIQIFFSMMFFNMVPLWKNIHAGMFSDHRPENGTFVHSGNYLSFVNQYTDIKGYFIVFFLNFYPSYNAAVTFLCMDLLIFIMVFHIAGHLNILVHDLRYFPRPNEIEQCLETGSKKYNEEVFVRLKDLIDRDQTIKEFMINISETFGISLCIYLAFHQVTGCVLLLECSPMTPEALGNYGFLTLMMFQQLIQTSIIFEFISTKSDMLADEVYSLPWELMDVRNRKAVLLFLKNVQPPRALKAGGVVSVGVLTMSTIIKTSCSYFLMLKTLTVEE*

>CpomOR9

MPSYPKDVFSLTYMNRIRFLLNMIASWPNQEFGGSKIKWQAASLYRCLLITFVIFNMTTTISYLQKYVNHDLAHSYVNMMLASVYLQRLFLPFQKKFCLMIKRFVLEFHLIHQKHKTENTAQVYERVNRICAIVTAVSVTHTVGLPLFYNGIPLYNNIKAGMFTKHRPANGTFQHSVYFDLPFDQYATLDGYLIVFFYNIYVSYNACIGICMYDALVFSIVFHIWGHINILIHKLKQFPPTTPQAFTTTVPAAGRQVTQEDMFVRLKDIIRYHQMIKEFMRCTSEAFSISLCCYLLFHQLSGCVLLLKCSSLDPIALGRYGLLTIMVFQQLIETSVIFELVNSKSDTLADHVYGLPWEDMDLRNRRVALILLHNVQKSLALKAGNMVPVGVLTMSTVWLGLRR

>CpomOR10

MVSVQKIISLAKRLEDPKHPLLGPNLKGLYVYGLWQSGSKFRNTCYNVIHFCAFLFVISQLIELWIIRHDYLEALHNLSLTALGMVCIFKAVSYVMWQSDWKKLVEGISAEEISQSDSLNDACIELKQKYTNYVRIVTYLYWNVVVSTNITMVSAPFLKYATSSEYREQISNGTEPLPQIFSSWFPFDKTTMPGYSLAIFIHILINIHGGGVIALYDSNAVAVMVFIRGQLGMLREKCKHIFDEYELVNQEIILGRIKECHRHHNFIMRHSSLFNSLLSPVMFLYVLVCSGMICCSVIQFTSEEATAAQKVWVLQYTTALVSQLFLYCWHSNEVVVECQHVDGGVYDSEWWKGDTHVRKQLAMLGGKLTHNIVFSAGPFTTLCVPTFIDVIKGSYSFFTLLTQMQE*

>CpomOR11

MSLLFDESLKSIDYIFKFVGIYLDRTILNTAEHIIKFRSLYVINFLWLNTDVIAEILWIIQGARHGKSLIELTYIAPCTTFCILANIKALSLLLNDDKVKQLFKQLRDMENNINIGDEIVKKKIVAEEKKFLRAVIKALSVVNALTLILFSLSPVLFMGLEYKKSGQIELVLPFLIVYPFNPYDIKYWPFVYMHQIWSANLVVTQFAGTDCLFYTCCTSICTQFRLLHHDIETIIPERNFGENEFLEKFKKLATRHEGIMQSVIQLESIYTKSTLFNFVSSSFLICLTGFNVTAIGDIGFMLSFLSFLLTSLMQIYLLCFYGDMVMTSSMEVSNAMYNSKWYTVSARAAKHLYVGQMRAQKPSKLTAFGYADVNLNAFRKILSTACSYFALLQTMESPTQA*

>CpomOR12

APKLSDNSKPTPSFKQSDSFKQNRFCWTVFGLWPGKIPEKYYKVFSFIYLIISYVAYNALLTLNLYHTPRRIETLIREIIFTFNETVVACKLSMILYKRKKIAAIFEMLDCEEFKGNDEVGREIVAKHNGYYKKYLLFNTVLSNFTYFSQVLFPVFGFWIFGNALDLPICKYYFLSDQTRNDYFTSLFLYQSFFMYGHMMYNVNIDTLIAGFMVLAIGQVKVLCHDLENLKMEKSIGDQSTTDLKQQYKLRKVLNHYELLLEYCDKFQDVIGGTMFVQYGIGSGIICVVMCGLLLPSSLETQMFMVGYFMVMNLQIFVPAWLGTQLTYKSEELTTAAYKSEWLPCSKRCKSSIKLLMERAKSPVIITGLKIFPLSLATYIQIMKTAYSCFALLRIIQDRQEQAAP*

>CpomOR13

MRPLRQIDCFKVNMKFWKLLAVWPPNDLQSYYRYYQMFFTAFILLNNLLATVNFIFLPRQLDMFIDEMIFYFTELAVTSKFLTFLFMHEKIVKILSVLESDMFQPESENGLKTIDKAKKFNVRYFKIVAAVSATAHISHIVPPILLHFILHVKLELPVCNFSFLSDDTKQKFIYPLYEFQALYMQSQVLFNISIDTFFLGLLIYAIAQLDILNDNFRKVTGKNQIVTRADDSIERAEKENTIKKLNDSIIHYGELWQFCFLVQDVFSITLFVQFSVASCIICVVLFRFTLPAPWQYFIFLGSYMFIMILQILVPCWFGTRIQDKSQQLSQAVYDCDWTAESRYFKSSLRLFVERANKPLSITAGKMFPLSLTSFTSIMNSSYSFFTLLRHMQSRQN*

>CpomOR14

MDATKAFRTSDSSAVLAPVFEDMEFKPFRETYKIITFNMIVGMLYPTPETAVCRLLGIVLVLISISPAALIALLDVWHSWQRGDIINIVRHITVLGPCLAAIFKMMLFYYTRDEAWRIIRKIDADHARYNILSESHKEIARRHIQNTQYYSEKCWAITVAVTVLTFPLTAVVLNFYNFVFKEEPVKYMIHDLEKPFSPPEDRFASPYFEIMFGYMAYCSLWYIISFIGFDAFFGVTINHACMKLELACKIMEDAMLEEDRDSRQSRMKEVISEQNDFFSMVELIQETFNFWLGLIVIATMCQICNCMYQIIEGYGIDPKYIIFILGTIAHIYLPCRYAAKLQVTALDVATHLYCCGWEHVNDERARKMVAFMIARAQVPLKITAFNMFYFDMDLFVSILQTSYSLFTLLRS*

>CpomOR15

MKSFRRKESSTLPLVPVTEELEFKPFRETYKIITFTMIVGMLYPTPNTEVCRVVGILTILVTMSPVCIVALLDMWNSWFRGDIINIIRHTTVIGPFLGAIFKMMLFFYSRKEAWSIIKKMDSDHARYNTLPEQHKEIARRHIQNTQYYSEKCWSITVATCVLTFPLTAVVLTFYNYTFKENPVKYMIHDIDKPFSPREDRFTSPYFEIMFFYMGYCSLFYIISFTGFDAFFGITINHACMKMELACKTMEDAMLERDRDSRHRRMLDVISEQNDLFRMVELIQETFAIWLGIIVIATMLQICNCMYQIIEGYGIDPRYLVFIVGTIAHIYLPCRYAAKLQVSALEVATHLYCCGWERVNDERARKMIVFMIARAQIPMKITAFNMFDFDMELFVSILQTSYSMFTLLRS*

>CpomOR16

MVKNKINIEDLYLSRAKFVMSFLGVWMPPPNESIFQKYFRFFMLSLQYTFLLFQVIYICQVLGDLEEISQSSFMLLTHACLCCKITVFHVNIEYFRELLAQMNSEIFMPQTEGHDKILKLQASRIKRLLMGFMVSSQTTIILFAIRALFDDANRYFPFKMWMPVSPDHSPQYELGFLFQFITLSMSAFMYFGVDSVCLSMVIFGCAEIDIIKEKIMNVKPIAERLVNRSITTKNVLDEHYKVLIECVAQHQAIVKFVKQVEDTFHLYLLFQLSAGVGLICMSALRIVVVDWKTIQFMSLMMYIVVMISQLFLCCWSGHELTATSLELHTVVYECCWYEQDVRFKRALLFTMLHLGRPMEFRAGGYVTLSRQTFVAILRMSYSYFAVLQQTNSRNEALELEN*

>CpomOR18

MTVSTVDNVTLFLNRPRNILLYLGIWLKPANYVSLYVAYAIIVMLTQYSFVFFEFIYIALAWGDMDAVTEASFLLFTQASVCYKVTRFMINKDNLVFLLSFMEEEVFQAQNERHVRCLLNQSIMIRRLCLFFLGSALTTCTLWGLMPVVDSTGGERIFPFLIWMPVGPEKSPQYELGYFYQMVAIYISAFLFIAVDSVALSMIMFGCAQLEIIMDKVQQIKRVPMSGKVKKQDREQLIQENKVLFVECLKHHQAVIRFIESAEDTYHANIFFQLSGSVAIICIIGLRITATTPGSVQFISMLNYMVTMLSQLFLYCWCGNELTIRSEILREVMYLCPWHEQSNSFRRLLWVAMERMKRPIIFKAGHYIPLSRPTFVAILRSSYSYFAVLNQTRNKEK*

>CpomOR19

MKNYFILKNLCRKIYLVGAGDFWFEEGEISKGKSLRYQFLCFVLFSIYIFMTVLEIIGVFFGDMPKDERSDCTTFAVSHTIVLGKMFSVILNRKRVKELNRKLVEICANHEDEHRVAENYRIMKINIWAFAVSVYGSFIFFLFEGIRKMMSGSHFITIVTYWPFYEDNSIIAVSFRFFTTLVLAVMMATMICIDSFAMIILIMYKYKFITLRYYFEGLRERFDRNNYTGNEEYATELLHAGFIEGIVMHSNLTRLSKDIDRSVGTVLALQVCLSSGSAVSLLLQLALSKDLTVAAQLKIIMFVIAVFFLLALFLCNAGEITYQASLLSDSIFYCGWDASSMRRDLRRLVLFSCAAAQRPIVMKAFNMLELTYGTFIQVVRGTYSVFALISAQNESLAQ*

>CpomOR20

MNQSNCLKYKSFNETFKFCSFALALGLIYPNRKNVCLRTTIFFFVLLFNFGTLFWFIWYTVKCLWELDIYNSTRNITVGVIILLFVFKTIYVNLKTDMFASLLEQITKDLLKGNNMDEDYQEIYDYYIKQGLFGQKCYVWIPLILTSIFPTYAGISMTYGSLRSDDFKKVMLHEMDLKYIEDKQYDFPYFELVFAYYFLGIYILIPNFAGFDGSFCIATSHLRMKIKLMTHGVQRAFTDSKDILELKARLKTCVKDHQEALEFYTLIQRLYGGWLFAVFLLTSFLISCNLYQIYLTGIDPRYTMFAATGVFHMYTPCYFASCLIELGEQTCTDIYCAKWESWADPTVTKFLIFIMARAQKRLLLNGLGIVFFNMESFVSLMQTSYSFFTLITSK*

>CpomOR21

MDTPERRAPRASPLDALSIGYIKILKAFLTISASWPYLTVGGKVHPVYKYYVRCIIPFGLTAISLEVWFLIDHFNVLSLFEVGQMYLTCFFAALSIARMFLPFCSQYGEIVERFLLSFHLIHFKHKGSYHLKIYEKLEWLSHRVVIITLVLGMFCAMAYNMMPIINNISSGAYKDDNKTVELAVYFSYPGFDPQDHYKFATVFNFYSVFECAILIAGIDILMSLFVMQIIGHIEVLKNSLLTFPEPKKSTNIINADFGRINQFAVLRAPMFTEEENLIIKEKIKDCVKHHLFIVSFTDDMSALFGPVLAIYLLFHQVSGCILLLEISAGGPDAFTKYGPLTVTIFGQLIIISTIFEIVNTKSELLATTAYSMPWECMNVSNRRSVCILLRRLQR

>CpomOR22

MKFEEADLIKHVNNNVDIKENLKFEYYLLPPKQQIFYQKLAFAMNVFRMGNQTWWGFPPIHKIFICNTWMVLIFSPMCLILQFVYMYKNFDDLNFRTLGTMFSIIPATAVVVAKIFICMIPAYPQIMKELMDKIHLNNFIDDEDLFIKKKLIQVERYTRWITLCLVTFILFDWLLWIFVPLMNNIKNKELIEKRLVRMETCLYLWMPFDYGYDYNTWAITHAMNVYLVGTGCCVFALYDSINFIFIFHFLSHIDVLRYKIKTYFATKLDESQTKRRIVDIIKYHSFILSTFKDIQAAFGLNVAINYAHNLIVDSLLLYQIMIGDKANRLSYVIMMQFHMGGLILMSLALEQIHIKTDDLPLLLYSVPWEKMSVPNQKLLLPILRRMQTPLVFKASGGLRAGVRPLASILKSTFSYYVMLKSSIE*

>CpomOR25

MFGSLLELSDDFFAYNLKYLFLVGLWPDDAWAKTHPSLYKIYENITHVLSIIFLITSGIGTYQIKDDVVLLMTNLDKCLVAYNFVAKVGIFVWKRRQVEILISEIVNSGDQLTEERKKMMLMIIIVVTGLSTSIVGAFSALALYHNELSVEAWMPFDPMESKMNLLTASQLLAITFVVPVIWRAIAMQGIVCSLIMYLCDQLVELQDRIRSLEFTSMTERVVREEFKNIVNKHVRLMGYTQDMNKIFEEYFLIQNLAVTLELCLNALMATMIGFEQKTLLATFFAFLCVALMNAYIYCYLGNEMIIQSGNLALAAYESSWISWPLDLQKDLVILLRVAQKPLYLSAGGMVAMSIQTYSQTLYNGYSIFAVLNDVVA*

>CpomOR26

MAEYDGELLSYLSLTPHLKVLRNCGIFPLDSTSPNIKKRLHGVYICISFCLIMLYTLLQIIHVFQVRTDIEKVMDAMFLLLTFLDCIFKQVMFMKKPHKILEILNIMKGPSFNQGLAEHRPLLVRTINHARFLLRLFNKLCILTCFLWITLPIYLHLNNEIVEFTIWVPFDTNENSKFYIVISYVWMQTTWLGLNNSTMDIFIVYLFAQIKTQICILRLNLENLVSRCQEEARNTSHSFTQLLELRFRGIIYHYNQIIKFSKINEEIFSNAILFQFLVSGWIICTTAYRTINMNPLSGEFLSMILYMICILSELFLFCFYGNEVAHESQRLMESAYCMQWEELPVKYRRYLIIFMERIKCSILPKAGKIVPLSINTFAQIVKTSYTFYTFLSKSNAN*

>CpomOR27

MLNKYVARLEDPNHPLLGPTLWGLQRWGMWQPNSGSRRIIYNLIHVAAILFVVTQYVELWFIKADLELALRNLSVTMLSSICIVKASTFVVWQTYWQDVVQFVSTLERSQLEKKDKTTCTIIERYTKYSRNVTCFYWGLVVATGLMVIFAPLGVFLSSSELRELMLNGTIPFPEMVSSWVPFDKTRGFGYWFQIVEHSAICFYGSGIVASYDVNTVALMSFFCGQLEILVANSKKLFSEDGKLVSYSEAMERIKQCHKHHLSLIKYSKILNSLLSPVMFLYVVICSLMICASATLLTKEGTTTMQRMWVAEYLAALIAQLFLYCWHSNEVYFMSESVDRGIYESEWWQCGVGLRRCVVLLGGQLRKTIIFEAGPFTNLTVATFVAILKGSYSYYTLLSNNEG*

>CpomOR28

MRVWARRKSATLAGASVVSLLRTLRWCGFCRLPPSAASGPQTSLLTSIARATHDVYCGFALVVTSTYLVQELIYAYLERGDMDTLARVMFLLLCHVTSIAKQIVFMARASRIAKLVQDFDDMAYNPEETTRENLLIERAQGASRLGAAYAGTAALTCALWTIFPFLARLGGTRVIFALWVPFGYYSWPEFLIVLLYTYYVTSLVGIANTTMDAFIATILGQCKTQLTILKMDFESLAERANERARETGEQVGAAATALLVRCIKHHHKICDTSREVQAIFGGAVLLQFAIGGWILCMAAYKIVGLSVASLEFVSMVMFLMCILTELFLYCYYGNEVAVESAQVSDAVYGMEWVGPNGVGKEVRRALPFVICCSVGAARRPLRPAAVFIPLSLETFVTIIKSSYTFYAMLRQTQH*

>CpomOR29

MIKEFLENIEDPNRPLFGPNYWLLNKIGLLLPKNRLDRILKIIIHEIATFFVLSEYMELYVIRSDLDLVLTNLKISMLGIVIVFKSNTFVFWQGNWRQVIDYITEADKFERDNQDEAKGNIINTYTRYCRRVTYFYWVLVFTTFITTMATPLMKYYSSETFREGFHNGTEPFPHIFSSWMPFDKENSPGCWITVMWHTGICAYGAMIMAAYDTSVVVILVYFGGKLDLLRIRCREMLGTEEKGVSNENADKVVQQLHQIHVLYLKHSRLFNSVLSPVMFCYVVMCSLMICASAFQLTSATNTTQKLLMAEYLVFGIAQLFMFCWHSNDVIHKSQAVMNGPFESDWWAANLQQRNNVLILQGQMGIVHIYTAGPFTDLTLATFVAILKGAYSYYTILRK*

>CpomOR30

MSKILDEDMNFDKIFWIATTAMRLNRSHPYVARDKIWRNQFIAILLLSLFCFIFLLYSILFHDIQCGLFADASKNAIMAIVAFTITYKYYILLRYQDSVTELIRIVDDDYELAKEFCEEEQRIVLHYSKRGVKVCQYWFISACSTSAIFPLKALMLMGKSYMAGEFQLVPLFELTYPWILEDYKTVPIIFITLFGLALFFDVYATSMYVGFDPIVPIFMLHLCGQLDILNLRISKMFSDTEHSDEAVRKNLTEIILKLQDVYKFIQVIKTNFTVLYEFMMKTTTFLLPLTAFQITESLRNGEINIEFIGFFTGVILHFYIPCYYSDLLMETGEKFRLAIYSCGWEKHSDMRTLPTILFMLTRAIKPVVISTIFCAICLDTFAQMCREAYSIFNLMNAAWA*

>CpomOR31

MSKILDENMKFDNIFWIATTAMRLNRSHPYIPRDKNWRTQFTAILILSSFCCMFLLYSTFFHDIPCGAYADACKSTIMAIVAFTITYKYLIMLRYQDSITDLIRIVDEDYELAKGFCEEEQRIVYKYSKRGVKVTQYWFVSACSTSAIFPVKAFVLMGKSYLAGEFQLVPLFEMTYPWILNDYKNVHVVFVMLFGLTLFFDLYATSMYVGFDPIVPIFMLHLCGQLDILNLRISKLFSNTQDSAETIRENLRRIILQLQDIYKFIEIIKNNFTVLYEFIMKTTTFLLPLTAFQITESLRNGEINLEFIGFFTGVILHFYIPCYYSNLLMETGENFRLAIYSCGWEKHWDKHVMRTILFMLTRALKPIVISTVFCAICLDTFAQMSREAYSIFNLMNAAWA*

>CpomOR32

MMKQIFMNLLSRYIPVWNQKNPSIANTALRLICNTGIWHYQSLGLHWVAKFAIICFISTNLTQVATLLIERDDSTRMFETFSVLSFCGMGTLKLFNLYTNRKRWTSIISQLQCIEHEQLHGKLLSCIDSDIEEDYSPQIIAKYTQRHTFISSVLLRLYSITAIVFIATPFVEYAVTADASYFPHILPGWAPLDNIGFAGYFLTLIFEIVASVYCVFIHVAFDCTSVGIMIFICGQFSLLRRKTEDIAGSGEDCMPSTMRDVRAHLKIIESHGTHIALRTVIKELDTVLRGILGVYFLVATLTVCSVAVRLNSESLSFMQLVSLLQYMAGTLTQLFLFCRYGDAVFHESSFNMGEGPFGAAWWSLCPRMRRQLAMLGAGMMQPRSLHAGPFNRLDLPSFVQIVRAAYSYYAVLGQTSK*

>CpomOR35

MSIEGYKLVRNKKISRFSLQNMLRCLEDPKHPSAGPYLRFLNLTGNWHPNMELKSTRFKQLIYYMTMAFFFSQYLKCVISLNLSAVLFILQTAPFHMGTPKTIYFRKDYHLWEKLIDYISRTELRQLSDGDVEVIDVMDEYIKKGRRVIYPFWFMVICCNISIFTEPYQKNQMVENGTDIYVPLFHFYVPFNQDIPPGYYYSMVLQTILGNIMSSYIISWDSLVISTFIFFTGQLKISRVYCTKIIDPESKERSHENIIKCHRFHTALIEHQKLFQKLISSVMFLYLIVISINLGSCIIQISNASGDLPVMMGAMLFVFGILTQLLIFYWFSNQVTVESLSVSSGIFESKWTTMDAKTQKEVALLQLTTSKRLCFRAGPCNEMSLDTFIAILKTSYSFFTLLKETK*

>CpomOR37

MECFSRAQDGFHRLKKRLRENSFDNLVCLVMVMPSLVGFEITRKKIFVPFWIIHLSLLTYVYGVGSLVYQAKHARVASDFIKSFVNVSILVLTVNNSYWWLTHRDLLRNVLRKANASDKMTIQAGLFVDKHQHSLSIIKRILIIFYFINLTNEFTSYLPKRAELNEKTFSMTPCVGIKPLTSSPQREVCIVLTSLQELTIVIVVLNFQTMMLLLIAHTSTMYQLLSDEIMTFNTILTNPSNYDLLKERLGVIIKRHILTLDIIKDIRVLYSIPMGINFGSNAVCMCFFFFLEPEEYFNFMPISMYCFIVFFLYCFLGQRLTNAAEVFSRAVYSCGWELMDIKEQRAISIMLLQSQKEVDLLAADLIPVNMLTFASTSQGIYKFVTVFKL

>CpomOR38

HHHDLPTMLWNADVLLRVMALDVDGRNNKRIPIILYLTSAIVLVLYFYTYHLSTYWYIFWRGGGDMLFIILLVSLSISSSIGVVKLLYMYFNGTKLKKLVLEYLECDAALAPDSRMFRNVNATLRSVKKRAIIFWMIIIGNGVVYVGVPLLKPGRHLTEDEQILLGLEPMYESPNFELANIALFAGVFLTVYAPANITGFIIIIVGYSEAQMLALSQELLHLWDDAHTHYKQIQSHSTDDPDCSNGISRELEAIKKNKLRMVNIYVKNRLVSIMKSHATNINLINQVESIFKSAIAVEFALLSCGLIAELLGGLENTYIEVPFALVQVSMDCITGQRLMDASKAFEDAVYACKWEHFDVRNRKLVMLMLQNAQKTLRLSAGGLASLSYTSLMSVIKSIYSAYTALRSTMNK

>CpomOR39

METLDKFGLAHCDLPTMMWNVAVMLRVVAVKIEGGATSIPIFFYLLATVAIVLYFFNYYASMLVFVLVGCRETGDILAGIMVLSISMNSLIGINKLFYIYRHQDKVQSLVADYVAYDRIAPWPGTSALMAELMRSVKKRLILFWTVTMGNAFIFNLQPLVMPSRLNLYDKHVVYGLKFILGITNYPIAIVNIVACTFFICYITSSIGGLLIVTTGYSEGRLLALSQEMRDLWSDAHKHYSENFEGDSEDKKAAKELNNYVKYRLQVIVKSHAINIDVIKKLEGIFRNAIAVEFILLTAGLSIDLLGGLEDTYIILPFSLMQVGMDCYLGQKLMDACKVFEDAIYDCKWENFDVKNRKTVLLMLKISQRTLSLSAGGVATLSFECLMAMYKAVYSAYTALRSTME

>CpomOR40

MLNLDYDKMFKISILALKLNRSYPTIPKDKFWFCSIIPIHAYFSLSFCLILYSMFFHDLKNNDFAAACTNGILSVLYIAVTFKYVVLLVKVEDITFAMNKVKGDFAAAKHLCSDEQAITTEYAYKACWVTKVWLLTASSVFCVFPIQVIVLSIYNYAIGDFQFVHMYQMTFPEAIETRKYETNMYLFLLILQTYFGVYVLLMFAGFTPLGLIFMLHVCGRIEIVKYRISKLFEGEHYDPREIHQRLKNIVTPLQDALDFVDLIKKTFRLVYEVYMKFTTIVIPIASYEVLESLKEGRLSIEFMTFIVAGAVLCFAPCYYSDLLMEKGLSLRMSVYTSGWEAYPDSAMRRTLCIIMCRLERDVAIRTLFQTVNLDAFSELCHQSYALFNVINTAWS*

>CpomOR41

MDVRFDNTFKLTSAALYLNLAHPFLTRNLQWAIRIFIFYVFYTPSFIFLIYSSFYDTFTKICTNLSISVLYCINLFNYGLLIIYKKPFMDMIKIVEENLRNSRELIDEDEKTVKEFTAKGIKAAKFWTFCCVLVGVMFSSKAVIGTSYSAFTGNFKPVAIHELTYPAYIEERKNGFLMYIVIFGFHTFYIVFTILMDAGFSPLGPIFILHACGQIQVAIQQVERLFLDNDIDVDDILNKLKNITRRLQNIYSFVDQIQYTHRLLYEMCLKASTIFVAISLFAIIESYKEGSLNFDLLCYSFSALLLCGIPCYYCEALLGKGAELRVAIYECGWERFWVPKSRSIILVLLTRTVRPLGIKTVFCTLSLEAFGDVINQVYTIFNVMNAAYN

>CpomOR42

MSTPTFYEVFRQIRINLSVMGIQEGKSRTGVIFYIFYAMLFTMVSSEVVFFTANMAPENFLELTGLAPCICVGILSLLKIAALAWKKETVFSLAHKLERLSTETLKDPIKTDIVSPDINLLKTLIKYYFILNAVLICVYNFSTPFYILYHYLTTNEEIFILPYAVTVPFSTEAWPGWTFVYVFSVICGFICVLFFTAVDALYFTLTSYVCTIFAVLSNEIICLNQPTGDILDQIIKKHQNVLELAEDLEDIFTLPNFFNVLVGSLEICALGFNLMIGDWNNVPGCMLFIMSVLFQLFMMSVFGEKLIGSSIKVGESAFLCDWYKMNQKTQKVLLVLITRTRKPTRLTAFKYSVICYEGFTKIISNSWSYFTILRTVYSPEDQ*

>CpomOR44

MKILSEYMREKLSFLTPCLPYGVLESWEDLNPRLYHAVHIYWLKFYGLWYNTHPKTSLLFWAHIVYAVVVLWLVCFLPGIGEVFYLLKRRDNIGDIAEGLYLFLSEMYTYIKLSVFWLKRKEIMALLEYLHMDEFKVKEPEHRQILRKSIKRARFVMTYYSSMCVGAVSVGILMPLAEQFEVLPTNVEYPYFDVYKSPAYEIIYIHHIYYKPATCIIDGVMDTILAAFIASAIGQIDVLAFNLRNFNLLAQRRREMLPFTTVNNMSPKHMPLVDHEKTRHCVRAVFKDIIKHHNSIIKYVSLIESAFSLASAVQLMLSVMVLCLVGIQFLSIEEPSSHPIQIAWMAIYLTCMLIEVFIICWFGDELIWKSWELHQAAFDSPWPSTDPKTAMFIVIFMERCKRPLRVTAGKIFTLSLDTYTNLINWSYKAFAVMRKMKK*

>CpomOR46

MGIIEKNVNFSVSVSITALKLFGFWAPEGLTREQNILYNCYGFLSFMFLLGTYLIIQVVDLFLIWGDVALMTGTAFVLFTNLAQTTKIVAVVARAGTLRPLVSSADTLLAAETGPGKEIVDSCNRETWQQQLVYFCLTTVTVAGWAGSAEKNQLPLRAWYPYDTSVSPAYELTYLHQVGALFAAAYLNVGKDTLVTCLIAQTRCQLQLAALRLASLTDDLTPTAQGTLTAEQEAEAWSRLEVAARRHQEALHAAAQLQACFSAPVFAQFGVSMLIICVTAFQLTAQNGNLVRLASMGTYLLNMMFQVFIYCYQGNQLSGESMDIATAAYSCGWTACGVRLRRGLLLVMVRARRAARLSAGGFATLSLASFMAIVKTSYSLFTVLQQADEQK*

>CpomOR47

MTNIVSSDRPSRYFGVHYGLLRFLGLGWWHHPDEGDFRNFPSWYLYYSILTQVVWVAGFVGLETIDPFVGEKDIDRFMFSLSFVITHDLTCIKLYLFFFKNRAIQEIVRTIEIDVYDYYQNVDKNRRTIRITRIMTASFVFFGWITIGNTNVYGTIMDLRWKREVALLNGTALKPPRTLPQPIYIPWAYQSDESYIATFVLETVGLLWTGHIVMTIDTFIGSLILHMSSQFSILQEAFMTAYDRALSQLISDMPLDIDTQDELINRNIYILKHRLDEIEAKVKSFYAEIQIESAIEKSVKSCLRQHQLLISCVEKFRVTYSYGFMTQLLSSMAAICVVMVQVSQDASSFKSIRLVTSLAFFMAMIIQLAIQCFTANELTLQAERVSDAVMQSKWERMSPRVRRYLLMAMMRAQRPLRLSAAGFAYMDNRCFLAIMKAAYSYYAVLSQKEV*

>CpomOR49

MIKYILKKLENPKRPLLGPNVKALQFWGLLLPENVIMKYVYICLHISIIFFTATEYVDIWFIKSDMNMLLENLKITMLASVSVVKVSTFLIWQNSWRDIIDYVTEADLNQRKTTDETKLTIIKKNTKYSRKITYLYWSLMYTTVVVVMVQPIIKYVFSQTYRDNIKNGEESYIQVVSSWVPFDKSEVIGYLAACAFQSYAAIYGGGWITSFDTNAIVTMVFFKGELQLLRRDSAEIFGIENNPVSREEAEKRLKECHRRHVNLIKYSSLFDSCLSPIMLFYMFVCSVMLCVTAYQIRYGTSMMQTILQVEYLVFGVSQLFMYCWHSNDVMYTSENVIHGPYESRWWSENALRKDLVILLGQYRKEIVFSAGPFTNLTLPTFISILKGAYSYYTLLTKSRTDI*

>CpomOR53

MKLTQCFKVSYFWLILGGVWYPRSLDNSRMLYAVNFYRVFATIFINVGMLAIQFIYFFTVVGKDLDKTVDATALFTFVGYLYKAITVIKNRQRINKLLDIIDNETDKDDLLRNMAANINFVSFFYNGWACLTAIMWNLIPFTKATLTLPFYYPDLTPSSPWFVTWYIYQATILIINGVAQTSADHLFGGLMAFAATQLKLLQHKLEAIGTKTDSTMEIDAQRQQEDYEETVSCVEYHLKIIWFVDELTDIFGGAAFGQFLLAAPLICLSMFIIMTSSDVTEIVTRILYFGCLSGQLFIYCFCGNLIKTQSDLVATAAYKSHWTSTSVRTQKALHLLIIRGQKTMSVVAGNLFELSLVTFGALLKSSYSFFAVLSKQRDE*

>CpomOR54

MKNSDCLASSIAVMKYTGVWMPDNLTYGGRMTYMVFRCVTQTFIFVFIILAEIAYVYKHRHDSERMVDAAVLLLSHLVQAVKLMTIIVRQERIKRLISLGDGPAFTPTEPKLKAQLERAVKLTGLIGNLVLWSACITGVFWFVVPALKDVLTLPLKITFPFDISGQYIFAVMYVYTSLSVLTCGVGDAAENFLVSGVLTLASTQVGLLHEQLLDLKTDGKDGCYKKAVLCVKFHQRIIEYVEEVAKIFGLPIFCQCVTSSIVVCMTVYKITITQEPVEMVTLVFYLICVMMELMMYCYPADVLLNKSLQVSDAAYPEWSGNIKTAQVLLLTTLRAQRALVVNAGGMFKISLPTAAAVVQTSYTYYALLQQKLKKE*

>CpomOR56

MERWRTYSLEYSDMPTMVANVADLLKHLGLNIDGKVRVIPILSVIWTVISVVLYFYVYVFSILWYVFWRGTGDPVVDAVLLSLACACIIGLLKLFVLHFNRKSLQYTLISYLSYDKRLCRESRMYRRLVKNLRVIKRRASAIWILLVVNGAFYCFMPLLLPGRHLAEDMQVIYGLEPMFESPNYEIAHILFWITIMVTVYCSGSIAALLITLSGYIEAQMIALGDEILDLWPDAQALTCETGQDEDKLRNLYIKHQLEYIVQAHAANLALLRLTESIFSNAIAVEFCLLGLALIAELLGGIENTYIEIPYALNQVSMDCFTGQRLMEASLRFSDAVYDCKWENFDASNMKTVLMILKNSRTMVLSAGGVAELRYTSLMSVIKSIYSAYMALRSTVD*

>CpomOR57

MTTTYSTFEAFRPHFNALAYVAYFKIIPKPSSGVKHTLHTVYRAVVWFLVIIYNLQHVIRVIQARHSTEQAVNTLFVLLTTINTLGKQVAFNSRVERVDRLVATIEGPLFMARNTYDEKVLRSNAWIMSRLLMMYHGSIYLCGAMWGISPLVSKLSGEVELTGYFPFDTSGWLGFGIAVAFNTIVITLQGYAHVTMDCTIVSLHAQTKVQLQMLRNSLEHLTDSVGKTGREICVQSTVYKDIEDTAFGVVLKKRLTRCVEHYKLIVWFHSEVEAVFSEAMIVQFFVIAWVICMTVYKIAGLSLVSAEFFSMFVYLGCMLGQLFIYCYYGTQVKAESEFINYSIYRCDWVSLSPRFRALLLILMSRGMRPVAPRIAYIIPMSIETYISVLRSSYTLLTFLERK*

>CpomOR58

MMPIKPFQNNRTSDLFHTICKIIYLSCATNFWFEDIDYPAIFMKIYNSTSRVLEVTVAVLIISDWGAFWTQPNLTEKQSNDRMLFAFSHVVLYSVYCSVIYYKREIRELVMTLTVRLKEVCYDGSIEKMMLRTTFRYTTAFVFVCSSTLFSFGIGSGFQALTTNATFTTIIPIWPDVEDRRLVAGAARIILYIVWWIFLVRFISVYIILLISTIGIAHQFKNLCKYFEDLTDIFEGSGSQEEKERRYENAFKVGIKMHSITLWCMRQIQLVGGVAFSGQVIINVSVLGLLMIQMMFTERTLIAVMPIIFMVSSVLVGTGVFLWNAGDVTIEASRLPAAMFHSGWHNCTRQSSVRVRKLVTIAIAQAQKRVRIKGLGFIELSYESYVTIVKSSYSLFSVIY*

>CpomOR59

MNTNAEARREIGATLTLCTFSMQCIGLSFERPDGTARLLRQKLMFVVSVCTIVYHVFSEIVYIGLTLSNSPRVEDVVPLFHTFGYGALSIAKVFALWSKKNVFTEHLDELSGIWPMEPLDEDARNIKEKSLTALRLVHQWYFSINVGGVLFYNVTPICVYMYQLWQGQDAVVGFVWMSWYPFDKYKPINHVFVYIFEVFAGQTCVWIMICTDLLFSGLASHIAMLLRLLHKRLETLAETEKSQEEYYQEIVANIKLHQRLIRYCNDLEEAFTIVNLINVVFSSLNICCVVFVIVLLEPFMAVSNKLFLGSALIQIGMLCWYADDIFHSNADVALAVYNSGWYRTDPRCRRALIFLIRRAQKPVAFTAMKFTNLSLVTYSSILTRSYSYFALLYTMYNDS*

>CpomOR60

MKYKPKISNSSTRYFQKLTQFVFIVTATNFWYKDVKLPNRFVKIYSHVSKFLEAIIITFVITGFGTSYTQKNLTPKQSADILMKSVSSFFVYTMYGFIVYNKEEIKELLFSLTVSLMEIYNDKIEKKMMMKIRIYVAGLMFVSSCPMIAYGVEGAFHVLTSNATFTTVIPIWPDLEDRRLVAGFARILIYIIWLLLIAHVIATYCLMICISICLSYQFANLCEYFLHLNNIFNGEGSQEYQEKRYEKAVKVGIKMHNTILRCVNQLQSSCEVVYGGQILINVCVVVLLMVQMMQSDRSLVQLAPIVLSVTGVLVTSGLFIWSAGDITFEAERLPTAMFHSGWHNCRRQSSVRVRKLITFAMIQAQHVVIIKGLGVIELSYDSYIAIVKSSYSVFSIIY*

>CpomOR61

MSSSAKEEFLAGMDYLSVITSRIFLYPFLGRSKTKLLCYYFICSLIIFASFQQFVFLCVSKLNSFLDIVNIAPNIGVCAMSVTKYIKVNSNKELYNLIFVHFRTDMWDIVSEKCQENVKILKRYQKIIHFITIWFVYYVVPLILIVTSFPILIMYYDNMVLGKELEHRYPFEAWYPFDKVKWYYAAYAWESFITGLVVCIYTFSDLINVSYVAYICLELKLLGTHLKELIGAEDIKQLKSSQNATAIHYKIRQKLRGYIIKHNFLANISSQLDIIFGDIMLVNYTFGSVFICLTAFTFTVTDELYSTLRCFFFLISLVISMLNQCVIGQCVSDHSEQLTQALYDSKWTYGDRQTRQLVLMLIMRMQKPFQLTAKGYIAMNLDTFTTICSTSYQFFNLLRTMYDPKAN*

>CpomOR62

MVFLTSLWRAITHTKALEESSGEMETTFFETVYRITYIAGLSRSDHSFFYKLYSNTVKLMIATFMLGEVWYMLTYVSSLDIVIEQMNVIVIQGMALFRYRYMRMHERVYKRLATSMQISNLDTSTPARKALLETWMKRSETYLKLMLGLGSLTLAAWYVYPLVDDIEYNLTVGLRLGVDFSRPSRYPIAYTIHIVAFHYTAFFIIVNDVIMQAHLIHLVCQYTVLADCFENILVDCEKHFKGLTRDQLVRDSRFREVYISRLGLLVGQHKKILMHTMELRKTLSPPMLGQVAASGLQICFAGYQVAMTLTVSFTKFFMSLLFLGYNLFELFVVCRWCDEIKIQSENISNALYCSGWECGVATMPGVRARFLLVLTRASKPLVLTAGGITDLSLNSYSNLVKTSYSALTVLLRLRHE*

>CpomOR63

MLEKLKRLYNKYDFDYSTGQVDPYKFHSTFYFILKAFTVIDEPLPMWSYVSLAVNVFDATVAVFFAGIATVHGISLLDISITTEAGVYCIVLIYKCLILTCTQLDKAHYHCFLRVLREDFRYVCAEGAKYRERFFENQLETWKVSLCSVIFTFGIAVGMASFALVSLLFYLMTRTPGDGSQRPLLVPFWFWDLDFGKTPIYEIALNFSNFCFVTYAYNYVFMIQTQVVWVRQIATKADLVIWAIQDLLQDIHPATNEKEKVHYAELIKYRMREIVSQHHSMYTLMEAYAGVYKKLLMFEQKLCGPVVCLTAYCTAEKLDEGEFNAILVLLCIATVTLVYIPCYLCTFLGLKVRSVSDACWNISFWNAGREIRPYLVLIMQRSLRPLPLQAPGFEEISIQTFSTKMTNAYSLFNMLRQTNI*

>CpomOR64

MKAFVSTAKTFLYKNDFEWNKEITLQNFHPQLQIFLAINGVFFNNRESKIRFILPVLSTLITLVAVAFEIFFIWHGISMNDYGFATECFCYFFILGSVGIVYSSVLLNRVKVFKLLHNMNNDFLFICNLKAEYRDTFLTGQLLIWRLCWSWIVFISFVSLLYISNTLLYLLYQSTLATQDEHMIRPLIFPMWLPEDDPYRTPNYEIFLALEVILIFVVLVTFGLYVYILFHLLLHYYNLMDVILIALDDLFDGLDESVVALNRGDPRRQAVQDELNIRMGQIVRWHLSVFDSVDDISSVYGPTLVYQVMFSSIVICLMAYQVAEQLSEGKVDYLFGILGIGACLQLWIPCYIGTLLRNKGFFVGDRCFYCGWHETPLSRLLRPDLIIFIQRTQRPVAIKFTGLPHLQLETFSSIMSNAYSLFNMLRQYK*

>CpomOR65

MTLLTIFRDIKSFVNKDGYDLERPDVTLQNFHPQLEVFFAIKGIFFNNCSSKKRFIWPALSSFMASVATGFELLFIWRALTIKNYAMATESFAYLIILGSVILTYLGVLTNRTTILTLLSEMSKDFRYICNLASNYRKSFLDGQLLIWKLTMTWGIFVICVAILYVLNTLLLLLYQSLFATLDEHYVRPLIFPVWLPHDDPYRTPNYEMLLVFDIAIILVAMASFGLYVPLSLHLFMHYYKLLDMILIAIDELFEELDESVVTLPVTDQRRLDVKAELSRRMGRIVRWHQSVFDSVGAITSIYGPMLVYQVMFSSVIICLMAHQVAIQMADGKFNYLFALLTFGAILQLWIPCCMGTLLQTKALSLGERCFYSGWYKTPLTQLVRQDLLIFITRTQVPVEIKFTGLPEMELHTFSSIMSTAYSYFNMLRQYN*

>CpomOR66

MESREYRKNKTTELFHNLDKVIYVFSGMNFWVDDNNVPKLVFNAYKRISKVINVAIVLFLMAEIGSFFTQNNLTEKQKADRVMMTFSHIILYYFTHSLIHRKETVTEILYTLAVSLKKDFNDEETERLMLKRTKIYMCVFVALCCISFVFYGVEGLARVLFSGKAIYTILLTNINTSNLSSDKKIVYRSYVFQTNAT

>CpomOR67

MMWLKSHPVITSTFTGHLTFYHTMEHLKDFASEFSKPFAICFDLLAKSNISIYNESKIRGKLRILALVVFYFTFYSSLVVSFKKVFTGELGFYELANLLPIFIVATQGAMKGIVIVSNLSKAKTVIDELGSMWRTTGLTKTQLMKKGVMLKRLNLCNAVFYWMNITGTWQYILVPLFETLFRNFVLGQDQLLFPFLCSLPYDAKRNWMVYLGTYFWESYSMLHLIYMYLGVEFLMITLCSHLATEFELLREEMLHAKPILEHSENTSYKDHIGRTLSYSNNEDDDAIDYFEEDSNENEVRADENGPNIQEVIRRHQTLIKLSELLDDIFNRMIFFNLLFATITICFFGFVAKIARDLPEMANNFVGVVASMIPIFNLCYYAELLSGASAGVADSAYHNLWYEGDTRYQKIIIFIIVRSQQPCCLTSMRYAQVTLNTFTTVLSTTWSYFSLAISVYET*

>CpomOR68

MASLDHLPSSFIDTIIVPVKLYRFIGLQFFDDDRARFKNYCKLILFILLSFIFSCGLILFFIKINEIDAGILEIANATPCLCLVIQSLLKLSLLRKKHLIRSVVYEIAEMWPGEMENREQKELMDNWLHRNKLICDSILKFTIFGLLIYNGVSLVIYFILRILDKNPAYVFPFELYYPFEIDSVWKYVAVYLMHILATTIIYECSYLSCDMFLFSLTVNVSMLLRLLHYDLVNIDVRRRGQEADESLANLKNIVKRHQKLLKLAEDLDRIFSAVMFTVLVFSSLIISFFGFLTIVIKGKFQQFMNLIAALEVLFSVFYIMLPGQILSDTSSGVADAAYQSLWYNSDERFRKIIIIMIARSQKPCILRAMGYADINFETFYKICGTTWSYLSVVNQMYQDSL*

>CpomOR71

MVHLKNVSFWRKKEYGVRKRYYDLEDYDATFAIPWSVQKWVGLRLTKTDPPHVRIFWDTYYWLENLNLWLAAVLELIYMVVRSTEYVEIFLSMPCLCNLLLAIFKSYKMVVYRPVFNNLVWELRTMWPQGTVTEEEDRIVSRTLRSLNMVVKGYYWCNILLVLIFLSPSFVALGYRAAGHDTPLILPYWYWYPFDPYEGGLGYAFALAFEDFHGCSAICFMVMGDLLFCIFLSHISIQFDLLAVRIQKLVPTIEPKHRLSAFTTEQMRRENCNSPEWEKTHLKELAAIIDRHRALIRLSGDVEEMFSGALLLNFLNSSMIFCFCGFCSVIVEKWNEFSYKSFLVTALAQTYLLCAHGQKLIDSSKGITNALYNCLWYNASKKVKGSVLITMHRSQKEIHVTTYGFSVINMASYATILKTAWSYLSLLLNVYK*

>CpomOR72

MSEFTNFEPMFQETYKFILDRIKSNQIYIMDEVSWRGHLCWIKLVVIILAAISHTAGVFERIGQGADLVELSTDLSAVLILWQVTLLYIQFCLNRKLLKNFILHMGSNWRTDDQLRPDMIAVKHDYVTTFLSWITVFYKAVNIYLFLYLMPRLLYIAVKHFILKDSVAFVTPFYVKMPFKFDDNFLLYCLVYLADSKILQDVGYLVTFDLLFMNAAMHHLRLMFVMLQADLRHLHDVGVEQAENTLKKLIPHHQNLLNLMVELSNAFGAIFIIHLAFFSGTMCFFGFAARIHCSPESIKNLFAANIILVCIFTCCYYGQNLTDASVDIAQAAYESQWHLKSQEYKKCILFIMLRSQKAQYIKSTSFTDVSLQTFTKILNVTWSFLSLITKVYEA*

>EposOR1

MDVFNLKYMRMIRFTLRSIGAWPSHEFEDVPATKLTLLSSYSYTCFLCIICCFGTIAQIAYLITNQGILGFIDLGQTYLTVLMCFVYIQRTMLPLQTSYQAEIIEFSTKFHLMYHKNETEFAAKMHNKVKRICEIVTGIQHLQIYYVLVMYNIAPLYSNFKSGMLSSEKPINGTYEHSVYYVLPFDHNNEVWYPVVGLYNFYVSYNLGAMFSCHDLLISVYVFHIWGHLNICEHNLNNFPRPSITRNSKTVPLRYSAEENKKVAAGLKEIIIHYIMIKKFVEKTSNTYSVTLCFYYGFHMVAECILLLQCSTLEVEALAKYGFLTVAVYQELIQLSVVFELIYAKGTSLIDAVYGLPWECMDNSSRRTVLILLQIVQQPLSLKACGMVPVGIQTMQAILKGSFSYFLMLRTFANQ

>EposOR3

MEETIQVFHRVLSCAGVTIYAKNNWDSKLWLTLQVFNVIIGFFTIVFTTCFVIINVSDILVCIQGACIWTTGLIMFISFVVCLLFRKQFRLFLNEMGFRDSMLEMPLIKYVLKLESGGKLNELKGMVVTSQEKLLKLTRVLLKTYVGSVWLCASLYLGDSIYQMITREDDSLRLIGFDMWIPWSLQNVNVYIATFIFNAYSGYLCCIAYPGLQLTIILLVGQTIRQLRILTFILLNLDELALEIVGQRGESWQEHCTEILTQCVDHYIKIKRFSNKLNVICQPFYLALILDAILLVCVCSVKIAISDKSAPDTMKYYIHEFCFILVVLMFCLLGQQVANESQQLEAAVTEKWYIFDRTHKTHVRIFKMALSQRMPIYIFGSITLSAPTFTWFLRTGMSFFTLVMSVFDEN

>EposOR4

MIKFLLDKLENPKRPLLGPNVKALQFWGLLLPKNQTMRYFYMLLHFSIIYFTATEYVDIWFIKSDMNKLLENLKITMLATVSVIKVMTFLLFQEKWISIIAYVTEADLNQRKTEDEINKSIIQNVRGYCRKITYCYWFLMYTTVVIVMVQPIIKYIFFPTYRDNVRSGKEAYLQVVSSWVPFDKNTVSGSIAAYVIQSFAAIYGGGWITSFDTNAIVMMVFFRAELELLKRDSVAIFGSEFALVSEEEAKRRLKECYKRHVDLVRYSSLFDSCLSPIMMLYMFVCSVMLCVTAYQITSETNVMQMLLQVEYLVFGVSQLFLYCWHSNDVFYNSQELVRGPFESVWWKRSVLRKDLAILMAQFRTRIVFSAGPFAKLSLPTFVSILKGAYSYYTLLNQSQVEKKPFK

>EposOR5

MSKIFSDDMKFDKIFWISTKAMRLNRSHPCIPRDKLWRIQFTSIFILSAVSFALLLYSIRYDIRDGKFPDACKNIIMAIVAFTITYKYGILLHFQESITSLIRIVDEDYELAKEFTEEEQRIVLHFAIRGHKVGKLWVISAVTTGLIFPIKAFLLMGKSYFAGELKPVPMFELRYPLGLEEYKDVYVIFVMMFLLTLFFDVYSTSMYIGFDPLVPIFMLHLCGQISILKIRLSKVFSDPSDSDEMVREKLRTLILKLQDIYSFIEVIKTNFTMLYEFNMKTTTFLLPLSAFQVTESLRHGEINLEFFGFFTGVILHFYMPCYYSDLLMETGNDFRQALYSCGWEQHSDLRARRTILFMMTRATKPLVISTVFYAICLDTFAKMCREAYSIFNLMNAAWA

>EposOR6

MSIARIFEYLKVKLDDGEKDSALKCSYVKKVELALSLIASWPHKEFGNPKLDASLAKYNLLLCFMATILPISGVTYMWYYRDIITFFDMGHIYLTILLNLLYFQRIALSRTDRYRQTIKEFLEKFHLLYVKDKSKYATELYDQIDVLSKIFTIFVLSQMSCGIFLFNFMPCFNNYSKGMFGENRVPNSTFEHAVYYYSPIDDIYTTEKGYWMLFIFNIYMSYNVSCGLCAFDLLLSLIVFQIWGQLKLLKHNLQIFPLPKMQDGDNKQPLLYSNEENEHIRRLLSENISHHKYILDFTDKCSNAFSEYLFLFYAFHQVTGCVLLLEVSKLSPEALAKYGPLTIIMFQQLIQVSLVFEIVSAKSEQLVDAVYELPWEYMNTSNRRTVMIFLHKVQTTISLKACKVVPVGIQTMTGIMKKSFSYFMMLTTFAAGDRN

>EposOR7

MDEGHLTNVYDLTYVKMVRFTLNCIGAWPNKDLGNSGRSAKILSIYNYCLLITCSMAMVFEFKYLKKNTGVQDFTVLGHTYITLFMGLLFVQRLTMPMQRGYCNVMKEFFTKFHLIHSKTRSKYADKMNTRVQKLCFIFSIMSHFQLYSGVFFYNLIPVYKNYSSGMFSEIRPVNGTFEQSIFYDLIIDQYTDTYWYLIVSIFNVFLSFTVVCSYCCFDLLICLNVFHIWGHLNILKDNINNFRKPEKPATVESPAWFTEEESKLVHQELCAMIEYHKMITEFMAQKSSAYSVFLYLYFIFHQVSGCVLLLEVSQMDAEALGKYGLLTFSQFEQLILMSIVFELISTKGETIIDDVYALPWECMDSKNRKTVAFFLMNVQKPMAIKAGDMVPVGVQTMFAIIKASCSYFVMLTAFAQEED

>EposOR8

MSLIKTKQLLKQFCKYVYYAGAGNCWYEDIHHETIMYKLYSFISFAIYTTMIFLENVAAAFGSFPAVEKNSAVMFAAIHNIVLTKMFLLLYHKDSIKKLNREMATVGENFEEEKVMKKQYRKVKYGILIYVVSVYLSLIAYGVESTRKAVVEGAPFYTVVTYFPYYEDTSMIAAIFRVFFYLTWLYMMLPMMSADCMPITFLICIAYKFITLRRHFERIREKFDEDMLTMDKKDAAENLSKGCLEGILMHQKLMFLADEINRVFGIIMSLQVCESSAVAVLLLLRLALSPHMDLTNAFMTYTFVSSLFILLALNLWNAGEITYQASLLSHAVFYCGWHLCEPGRRARRDVRRLVLIACAQAQKPLVLKAFGIQDLSYSTFVSVTRMTYSVFAVFYQRGG

>EposOR9

MKNYYILKNLCQKIYLAGVGNFWYEEGXIGNDQTLKYMMLCSLMFFIYICMTILEIIAVVFGDLPEDEKSDCVSFAVSHTIVLIKLSSVMANRKLVKELNYKMVEICQQYEDEKRVAENYKVMKMNVYAYVSAVYGSCACFIFEGVRKMQTGSHFITVVTYWPFFEDNSLPAVLFRFFTTWVLCVLMVPMISIDSFVMVTLITYKYKFITLRLYLENLREEFDKENNAGNEESAANKLQAGLIEGIVMHRNLIRLSKDIDQSFGTVMALQVCLSSGTAVSLLLQIALSNDLTFVAGMKIIFFVAALFFLLALFLCNAGEITYQASLLSDSIFYCGWHACPMRRRSDLRRLVLMACASAQRPIVMKAFKMLQLTYGTFLTVVRSTYSVFALFYAQNE

>EposOR10

MIVQKIISFAKRLEDPKYPLLGPNLKGLYVFGLWQSGSKLRNSCYNFIHFCAFLFVFSQIVELWIIRHDYLEALHNLSLTALGMVCIFKAISYVLWQTDWKELVECVSMEEIAQMRAQNDSCAKLMRNYTNYARIVTYLYWNVVVSTNITMVSAPFVKYATSPEYREQISNGTEPAPQIFSSWFPFDKSENPGYFVAIFVHILINVHGGGMVAIYDSNAVALMVFIRGQFAILREKCKGLFDDTEKITRNEVLGRIKECHRHHNFIIRQSILLNSLLSPVLFLYILVCSGMICCSVIQFTSDEATASQKIWVLQYTTALISQLFLYCWHSNEVIVESNNLDGGLYESDWWKGDVRIRKQLVLLGGQLRHPVAFSAGPFTTLCVPTFIDVIKGSYSFFTLLTQMQEQ

>EposOR11

CVVVLEIGAADCLFYTCCTVIRTQFRLLQHDFEGIISTNENEFQEKFQKLVVRHQELMRSVHLLETIYTKSTLFNFVSSSFLICLTGFNVTAIDDVAFVLSFLTFLLMSLLQIFLLCFFGDMIMTSSMEVSNAIYNSKWYLAKPNTAKQLFLVQIRAQKPCKLTASSYADVNLRAFMRILSTAWSYFALLKTIDT

>EposOR12

MPPPQSDSFNQNCFFWKVFGIWPGREPNKYYKYFSFTYLVTTLVAYNALLTLNLYYTPRKIDLFIHEVIFCFTEVAVVTKVSMILFKRKQLVAIFDFLDCEEFKGNDDTGRKIVESHNSNYKLYYKINLILSNFTYLSQVLFPVIGMLIFKTSLDLPICKYYFLTEEEKDDYFSLIFIYQSVGMYGHMMYNVNIDTLIVGFMVLAIAQMKVLSHDLENLKIEKGDTGSQEELDHKQLQKLHKCVKHYALVLNYCADFQEIISGTMFVQYGVGAACICVTMCGLLLPDIKIETFIFMVGYFFALSLQVFVPAWLGTQLLYESQELMVSAYKSDWIPRSRVYRQSMKLFVERAKHPVIITGLEIFPLSLATYISIMKTAYSCFTLVRFMQDREDH

>EposOR13

MTQWKQIDCFSINIKFWKFLAIWPSNRMHPFYKYYYKAFVAFFVFLCNFLFTINFYYLPRHLDLFVEEMIFYFTELAVTSKVLTFILMHDKIVMILNTLESKIFQPETEEGLKIIDEAKKFIVRYWKIVAVVSVTSNLTHILSPFITHLFLPVQLNLPVCSYSFLSENTTQTYIYPLYWYQALSMHFHMLCNVNIDTFILGVLILAISQLDILDGKLRRVTEKEPRPGEIGEFGEVQAVVAVQSDESMLKLKESIIHFDELGKFCDLLEEVFSLTLFVQFSMASCIICVVLFRFTLPAPWQYFIFLATYMFIMIIQILVPCWFGTRIKDKSQLLSSAVYNCDWTAKSRYFKSSLRLFAERANKPLSITAGKMFPLSLTTFTSIINTAYSFFTLLRHMQSRQG

>EposOR14

MFQRRINTVGEGEIIRISTEDEFKNFRDTYRITTFHMIVGQLYPNPETDRQRAICIGAIIISVTPVTAMILLDVYNSWLRRDIVNIIRHMTIIGPLSFTLLKMILFFMRRSEAYQIIKTIDNDYSSYNTLPEPHKVIARRGVRDTKLYNEFWWAIVVSLNVVTFPLTAGILNVYNCLFKSDPKLYMVHDVEKPFSKPEDRFMSPFYELTFVYMVYCALIFVLSFVGFDSFYGITINHVCLKIKLLCLRLDDAMMETDSDAIFKKMVDVVKQHNAVFRMVDLVQDTFNIWLGIILLATMAQICNCMYQIIEGYGIDIRYLMFIIATMVHIYMPCRYAAKLKDAAAEVSNRVYCSGWERTSDLRARRMAMFMIARAQIPVNITAFNMVNFDMELFVSIMQTSYSLFTLLRP

>EposOR15

MDDDFKPFRETYKIITFNMIAGLIYPNPETERQRAFCIGLALITMIPGYATVALDAYNSWQRGDIVNIIRHITVIGPLAFALLKMILLYIRRAEAYQIIRTIDKDHAEFNTLPEQLKIIASNAVRDIKYFSEYCLAWIVFLSVLTFPLTAAFLNVYNYLFKTEPVLYMVHDVQKPFSEPEERFSSPFFEFMFVYMIYCAFIYILSFIGFDAFFGVTIGHVCLKIELLCERLDDAMLETDSDALFHKIVDVIKQHNAVFGMIDLVQETFNVWLGAILVVTMAQICFCMYQIIEGYGIDPRYLVFICATVIHIYMPCRYAAKLRAAAAEVSNRVYCSGWERTSNLRARRAAMFMIARAQIPVNITAFNMVNFDMELFVSLLQTSYSLFTLLRP

>EposOR16

MAKTKTKLNLGDLYFFRARIIMSILGVWNPPDNESILRKLYRYFMLSLQQVFLLFQVIYMVGVMGDLEEVSQSSFLLFTQACLCFKVTVFQVRIGSFRELLAQMNSDVFMPQNKVHEEILKLQATRIKRLLLGFMISSQTTCSLFALRPLFDDANRNFPFKMWMPVKPDTSPQYELGFLFQLLTISMSAFMYFGVDSVCLSMVIFGCAQCEIIKEKLLTIKSASEKRLNNSIDANSILEDNYKILIECVVQHQAVVKFVKLLENTYHAHLFFQLSGSIGLICMTALRILVVDWKSVQFASMVLYLSVMCSQLFVCCWSGHELTVISSELHTILYSCCWYEQHAQFKRALLFAMLRMSRPLEFRAGRYVPLSRQTFVTILRMSYSYFAVLKQTNTRNEILDS

>EposOR17

MEPKCLSINLSHILSCSSGLSPKYETPFYEATFVITCLGTAFSAINQTGYIVLFVTLVAHELGHFYAITENFNDIFVILTKRKNPEEPNELLTNPSKLVDEKLAFCVRHHQFLIRYHGKIRELYQAIFGTQFVLMIMVLVTTLQTMNSWDFSNTILTGVTGVMPLTIYCFGGELMISAAADMSQAVYSCGWELMNVKQARTVYLMLSLSQRPLHLSAAGFATMNRETFANVVQAVYKIYAVFN

>EposOR18

MSDXNXVLFLTRPRNILRYLGIWLQPDSFTLLYTIYMLFILLTQYSFVLFEFIYIVDVWGDLDAVSEASFLLFTQASVCFKVTRFLIHKDKLIMLLQFMEEEVFKPQNKEHERFLTILSTRIRRLCLFFLMSACTTCTLWGMVPIVDSASGERFFPFLIWMPVGPSKSPQFELGYFYQMAAIYISAFLFIAVDSVVLSMIMFGCAQLEIIMNKVEQIQKVPLSGKLKQTKQEQRIRENYELFQECLKHHQAVIRFMDIVEDTFHANIFFQLSGSVAIICIIGLRISIVEPGSMQFISMLNYLMTMLSQLFLYCWCGNELTIRSELLRDVMYLSPWYEQSNPFRRLLWVAMERMKRPILFKAGHYIPLSRPTFVSILRSSYSYFAVLNQAQNKEK

>EposOR19

MKSQSIEIPKQKYKSFDDTFKFCSFSLAVGLLYPNKRNLRTRIILFLCVLLFNFFSLFWYIWYTTKCLVKLDVYNSTRNITLGVVISLFIFKTFYAHWKTAIFAKVLKQITKDLLKGNDMEEDYQEIYDYFIKQGKLGQSFYLFIPSLLSFMFPIYSGIAMIGGSLKNDDFKKYMIHEMDLKYVEDKQYESPYFELIYAYYTTPCFVILPNYVGFDGSFCIVTSHLRLKLKLVAHGVQKAFEDSTSTTELKERLKICIKDHQEALNYHVLIQNMYGGWLFAVFILTTFLISCNLYQIYLVGVDPKYTLFALTGVFHMYMPCHFASCLIALGEEVCTDLYCVKWEAWSDPAITKLLIFMMARAQKKVLLTGLNLVTFNMDTFVSLMQTSYSFFTLITS

>EposOR20

MNGNPSSGEAEFKPFRETYKIITFNMIVGQLYPNPKTDRQRAIYIGLAIISVAPFCAMALLDVWHSWQRRDVINIIRHGTIIGPLCGALLKMIIFYVRRAEAYRIIKTIDDDHSRYNTLPEAHKVIARNGIKDTQLYSEYCWAWIVALSVITFPLTAAILNIYNSLFQSEPTFYMVHDIEKPFSKPEDRFTSPFFELMFVYTMYCAIIFILSFTGFDAFYGVTINHVCLKIKLLCKGLDDAMMETNPEAIFHKMVDVIKQHNAVFRGYGIDPRYLVFIFATVVHIYMPCAYAAKLKDAAAEVSNRVYCSGWERTGNLRARRAAMFMIARAQMPVNITAFNMVNFDMELFVSLMQTSYSLFTLLRP

>EposOR21

MKILRVFLTIPASWPYEVFQGSVHPVYWFYRRCIILWALLAISGEVWFLVTNIKLLSFFEMGHMYLTCFFAILGFVRSLLPLFDKYGYILKSFLMSFHLMHFKHMGGRYQEIYVKIERISYRFVSITMTLAMICAFCFNLIPIVNNVSSGAYRDHNKSIELAVYFSYPHYDPQDHYFFVTGWNMFIVVACSILIAGIDCILLLFILQIIGHIEILRHNLSDFPKPEDVRAVKVDCKGSRKPVFVNVAMFTKEQNLIIKENIKKCVQHHLFIIKFTDNVSQFFGPVMAIYYLFHLVTGCLMLLEMSAGDAASLARYGPVTVVIFGQLVILSMVFETVNTRSEMLVDALYDQAWDSMELSNMRSACLLLHRLQNPIAIQALGVTPINVRTMVMILKTSFSYFAFLKTLDV

>EposOR22

MGVAMTLFRLGNSVWWGLPPTPKIIRLHGDTFVIVALICLVLQIVYLYTYYSELSFMTLGTMFAMIPATIVCNAKLIVSMKPAYKRIMQNMMDKIHVHNFLDENDEFIKKKLVKVERNIRWITSFLVGFIFMDWLLWIIVPLRNNIINKERIANGTVRLETCLYMWMPFEYGYDFKTWLIIHSMNVYLVFTGGLVLPFCDSINFIFIFHFLSHIDILRHKIQTCFDGELDDKETKEKIVGIIKYHSFILRTFDDMKAGFGANVTLNYLHNLINDSLLLYQIMVGDKANRLTYLFMIQFHMGGLIVVSFALEQIRIKTDDLALVLYHVPWEKMSTTNQKLLIPILLRMQKPLVFVGALGLETGVRPLANIIKSTFSYYVMLKSSIE

>EposOR23

MTTKDLEESLTITKLTLLFSGIKMERSNFDPFLSFLFIFNFIWLNTDVVGEFYWLIEGVELGKSFLELSYIAPCTTICLLSTAKTLPFYFNQDVFVKVFDKLKAIHPKTEEDNEQLNDEIKENVKDSTKFLKTLVRMHLTSCSVVVLAFCFLPISAMGYTYFKTETSEVIFPFHIKYFFDPYEKNVWPFVYFHQVWSTFIAFTNVFGTDTFFYAFCVYTKMHFSILQKRFQNAVASWSAQRPEDGKKKFVEVIKRHQELIHLVDQVEILYSKSTLFDIVSSSVLICLSGFNSLEDMGVVFSFLAFLFMSLTQIFLLCYFGDMLMRSSMELSEAVYNSSWYQMDQSMKKNILFVLARARKPCKLTAANFADVNLRAFTTILSRSWSYFALLKTVYK

>EposOR24

MASLFDDSLRRIKLVFALSGMHLEHKSSKTMSDVIRYRWLYVMNFFWLNTELIGEILWIIDGANNGKKLRELTYVALCVTNCVLANIKCLFLILNEDELTQFTKCLQDLEEDEKESENSGKTTIVEEHRRFLNVFISTLSSSMALTCIALNLTPLVLMALGFRKTNEIELILPIPVVYPIDPYDIKYWPLVYLHQIWSTFLVAFKIGGIDCLFYYCCTVIRAHFRLLQHDFECINTCNEMEFQEKFQKLVARHLLLIRSVDLVDTVFTKSTLCNFMSSSFIICLAGFNVTAIDDLAFVLSFLTFLVSCLLQIFLLCYFGDMVMTSSMEASNGIFNAKWYLLKAKTAKQVLLVQIRAQKPVKMTAYKFADVNLQSFISILSTAWSYFALLNTLNDG

>EposOR25

MFGNLFELSNNIFAFNLKYLFLAGLWPSDKWARENRFMYKMYEVSLHVLAAIFLIVTGIGTYRSRNDIMTLLGNVDKSLVGYNLIAKVIVFLYKRKQLEILITKISHSGDQISEERKKVMLMHVVVVTGLIMTIAGAFSCLAFYNNEMTIEAWMPFDPMDSKMNLMLSMQILIVMFTVPCVWRGIAMQGMVCSLVMYVCDQLVEVQERIRALVYSPESERAMREKFKDIIKKHIRLMGYSQAMTGIFKEYFLIQNLAVTLELCLSAFMATIAGLEQQSLLASFIAFLCVALLNAYIYCYLGDELIVQSQGVALSAYESTWTSWPLDLQKDLLVLIRAAQKPFKLSAGGMAAMSMQTYSQTLYNGYSIFAVLNDAVD

>EposOR26

MSEPEGEPLSYMSLIPHLKNLRACGIFPLPPETPILKRKLHKIYMSLNFAFIMTYTLQQIVNVFQVRNDVDKVMDSMFLLLTFCDCICKQLAFLMYPHKILEMLEIMKGIAEHRPLLLKTVYQAQLLLKVFNKLCLLTCVLWVLFPIISHFKQETVDFTIWLPFDANVDPQFYIAIFYVWLQTSWLXYNNSTMDIFIMYLFAQTKTQLTILRLDLENLVSRCKAEAAVSSCTYTDSLERRFRGIIQHHNEIIKFSTMNDSIFNGPILFQFLISGWIICTTAYRTVNMSPASGEFLSMILYMICILSELFLFCFYGNEVAYESEIIMESAYGMDWTELPVKYRRFLIIFMERIKYPIKPKAGKIVPLSINTFIQIVKSSYTFFTFLNKAGK

>EposOR27

MLKKYVSRLQDPNHPLLGPTLWGLQSWGMWQPNHGLSRIIYNLIHLAAILFVLSQYVKLWLIRTDREAALRNLSVTMLSSVCVVKAGTFVFWQQNWREVINFASTLEKSQLEKKDKTARDVIEGYTKYSRSVTYFYWSLVTATVFTVILAPLGVYLSSVEQHELMRNGTIPYPEIMSSWFPFDKTKGVGYWVAILEHSLICFYGGGIVANYDANAVALMSFFCGQLELLSENCRALFGDGSEIIDYSESMHRIKMCHYHHLSLIKYSKILNSLLSPVMFLYVIICSLMICASASLLTNESTTAMQRLWVAEYLVALIAQLFLYCWHGNKVYFMSERVDRGVYESEWWQCGVRLRRGVLLLGGQLRKTILFDAGPFSTLTVATFVAILKGSYSYYTFLSNNED

>EposOR28

MCKKTVCTAEKYKAPISYVEIVLFTLRICKFRNFKAESVSDRVARKLHDAYCWFSLVVTSAYLGQELIYAYQERGDMERFSRVMFLLLCHVTSIAKQVVFLVSASRITALLEGLDDAAYNPSEAKRASLLLERAQGARRLGALYAGTAAATCALWTLFPLLSRLRGGSVHFAFWMPLDYNATWLEFFIVLVYTHYVTTLVGVANTTMDAFIATILGQCKTQLTILKMDFESLGERAAERAKATGERSGAAAASLLVQCVTHHHKICHTSRELQSVFGSAVLLQFGIGGWILCMAAFKLVGMNVASLEFVSILMFLMCILTELFLYCYYGNEVAVESTQVSDAVYGMRWTGGEVEGVGGRVRRALPFVLCCCAGAARRPLRPAALFIPLSLETFVTIIRSSYTFYAMLRQTQH

>EposOR29

MIKSILDSLEDANRPLFGPNYWLLNKIGLILPDNTFERIYYILIHEIVTFFVFTQYMELYVIRSDLDLVLTNLKISMLSIVCVVKANTFVIWQNNWRQVIDYINSADKFERDNQDEAKRKIIHAYTKYCRRVTYFYWVLVFTTFVTVVTTPLMKWYSSSTFREGFRNGTEAFPHIFSSWMPIDKEHSPGSWITVVWHTGLCAYGATIMAAYDTSVMVILVYFGGKLDLLRERCKEMLVVAGEVKTDKDADAAVKQLHEIHVMLLKHSRLFNSVLSPVMFVYVVMCSLMICASAFQLTSATNKTQKLLMAEYLIFGIAQLFMFCWHSNDVIYKSRNVMMGPYESKWWTASLQLRKSVVILQGQLRIVHIYTAGPFTDLTLSTFVAILKGAYSYYTLLRK

>EposOR30

MAVLKFEEVYNVPITCLNLNRAHPKIPKDWKWALQVGIMHGIFTLTFSTFIYSLIAVDIKGDDFGKACTNGIFLVIWVVVTFQYGVVLKFKEEISTLIRINQENFDAAESMSDEDQAVIRDYAFRARWVAKVWCISCCVNCALFPLKSLVLTLYSLYIDDFRLVPWYDCSFPEFIDKIKYRPDIFSIIFSTHVFYGVYAIGMYIGFCPLGPIFILNTCAQLELMAKEFKNIFNVTPYNEKAVSEQLCALIKKSQDIYYYVQLINDVFKVLYEISLKASAIMIPITLYQVLDSMKRRELCMEYMAFILTASLLCYVPCYYSDLLMEKGEALRLAIYGSGWEARFHKGSRNSVIIMLTIAIRPISIRTLFRTVCLDAFAGLCHEAYSIFNLLNAMWG

>EposOR31

MSTLQFEKVYDRTLICLKKCCTHPSTPIDTKWAWQMGFIQVFYTTGFVSIVYSFSMNLMKRDFSQAYNDAIFMALYLVVTYQSAVVLWYRNPLNTLIQKTEEYFSLADTLPAEHQSLVIEYAKRGRWVMRLWYFSNMTTCALFVIKASVLTLHSLYAGDFQLFLLYDYTLPPALEAVKYRYEVFSFILGLQVFYGLCALRTSVGICPLGPIFILNACAQLELVALEIKTIFEERSERNVAQKLVAIVKNLQNIYSYVESINKMFKVMYELTLKTSALLMPILLYQVIESVKRGEASFEYMMITYTAVLLCYLPCYYSNLLMEKGEVVRLALYDSGWEXHFDKRTRVCLTIMLTVALRPISIRTIFRTVCLDAFTDLCQQSYAIFNLINAMWN

>EposOR32

MKNIIGMQLRRYLPVFKSEHPKLVNTIQRLIWNTGVWSNESMGLHWMAKLVIVCFFSTNFTQLAAMIIERDDSERVFECFSVLSFCGMGALKLLNLYLNRERWLFIFTQITLLENKQLNNAIDKQCDDYDTDDEEECLSPHIQRYTRKHTYTASLLLTLYSTTAVIFILAPFVEYALQHETKGYPHILPGWAPLDAVGFAGYFVSALFEVIGSIYCVFVHVAFDCTSVGVMIFICGQFAMLRESTENIGGRGRGHTISRNRDARARLRIAKTHSTHIILCNVINELGSTLRTILGVYFLVATLTVCSVAVRLNTQTLSFMQLVSLLQYMCGTLTQLFLFCRYGDAVFHESFFSMGEGPFGAAWWGLQPRVRRELAMLGAAMAKPRPLRAGPFNTLDLPSFVQIVRAAYSYYAVLGQTSK

>EposOR33

MQHLQFEKMYETTSFCLKKSCTHPSIPIDRNWAWQIGLKHGLYTMGFVSVVYSFSINMTKGDFSRAYSDAIMMIVYLAVTAQNGAVLWFRDPLNTLIQKTEENFELADILPVEHQSLVVKYARRGRWVMNLWYFTNLTMYTLYVIKAAALSLHSLYAGEFRLFLFYDYTFPPALEEIKYRYEVFPLIIGLQIFYGLYALRSTVGFCSLGPIFILNACAQLELVAMKIKTIFNEHSEREVAKKFVDIVRSLQNIYSYVRLINEVFKIMYELNLKMAALMMPVLIYHIIERVKQGEMCFEYILLACKAVLYSYLPCFYSNLLMEKGEAVRLAIYECGWEQVFDKSSRNSLIIMLTVALQPISIRTIFRTVCLDAFADLCRQSYAIFNLINTIWD

>EposOR34

MEDKLVFEEVYKINMTCLRLNLSHPSVPRDLKWLLTFILMQGLYTAFNAICLYNLIFINVKENDFPNACSNGVYMVIYFVVTFKYGVMVWYQKDIKDVIRYQQEYFDSFREYTVEEQAVVKDYIQRGQWVSKLWLRSTIVTAGMFPVKSFIDSAYSAYAGDFRLHSFNENSYGPYIDEIKGRVDVFILMYAIFSVYTTYTAIMYSGFAPFGPLCILNACAQMDIVMMRVNHLFDEGFDKEKSPKQLQNLVKFTQNIYGFVDQINDIFQVLYEMCLKASAILIPISLYLIIEGFSEGKLYYDYVMFSYMASLLCFVPCYYSDYLKEKGDDLRCAIYASGWEKFYDRNTRVTLRIMLIRATRSLSIKTVFRAVCLEAFSDLCKEAYVIFNMMYAVLH

>EposOR35

MEMSNIHGAEVKRTKKKNKCRFDLRNIFKFLEDFEHPSVGPHLRLLSLTGQWHPDQDSISTRLKLAIFFITVILFCSQYVKCFVHIDISSLLLILQYAPFHMGILKTTFFQKDYSKWEELIGYMSSTERTQLREDESEVKPVMENYIKRSRRVTYFFWALAFFSNFSIFSEPYPKNQAVENGTIVYVKIFNFFTPFNQDAPPGYYVSMAIQTVLGHIVSAYVVAWDTLVVSTMIFFAGQLKISRIYCVRIIAAGSMEQNHRNIVACHQFHSSLIKYQQLFNSLISSVMFVYLVVVSVNLGVCIIQLSQLKDDLAALLASVLFVVACLIQLLIFYWYGSQVTEESVLVSYGIFESNWVEMDAKLLKEVTLLQLATSKRLVFRAGPFNEMSLTTFVAILRSSYSFYTLLNETK

>EposOR36

MSISQFENVYDITFICLKKSCTHPSTPIDAKWAWQMGFIQGFYTLGFVSIVYSFSMNLMKRDFSQAYNDAIFMALYLVVTFQSAVVLWYRNPLKSLIEKTEEYFNLAHTLPVEHQSLVIEYAKRGRWVMRLWYFSNMTTCALFVIKASVLTLHSLYAGDFQLFLLYDYTLPPALDAVKYRYEVFSLILGLQAFYGLCALRTFVGFWPLGPIFILNACAQLELVGLEIKTIFKERSERKVTQKLVDIVKNLQNIYSYVELINEVFKVMYELTMRTSAIMMPVLLYQVIESVKRGETSFEYMLFTYKAFLLCYLPCFYSNLLMEKGEAVRLAIYDCGWEQVFDKSARNSLIIMLTVALQPISIRTIFRTVCLDAFADLCRQSYAIFNLINXXWD

>EposOR37

MGLFNYFRENMRASKAKLSENSLDSLVWLVNIIPGIVGFDIFREKIFIPFWILHIFLLTYVYGVGSVVYQARDAEVASDFIKSFVNVSILALTANNSYWFLTKRQLLRSILKKVKENDNFVVQSGLFAEKHARSLFLIKRILLIFYGLNLTNEFTVYLPKRVDLHKETFSMTLCVGLEPLTSSPNREICKAFLGIHELTIVCVVLNYQTMMLLLIAHTAVMYQLLSDEIMTFNTELEDHRNYESVQESLTVIIRRHALILEITDDLKLLYNMPMGVNFGSNAVCMCFFFFLTLEEYLGFMPVLVYCFLVFFLYCFLCQRLTNAAEVFARAVYSCGWEKMEIKEQKAIYTMLLQAQKPVELLAADLIPVNMYTFGSTIQAIYKFTTVVKL

>EposOR38

MEALQRFGLKHHDLPTMLWNVTALLRVLALDVDGRNRRSISPVAYLFMTVTFIFYFYTYHLSTIWFVFWRGGGELLFYILLFSLSISSLIGVIKLIYMYLNSKNLQKIAAEYLLCDAAVIPGSRLAENIKLTLRKVKRRATIYWWLIISNGLVYVVVPLLTPGRHLTEDDQILWGLEPMFESPNYEIAFTIMSFGVFLTVYAPANITGFLIIIVGYTEAQMLALSQELLHLWDDAQNHYQRIHGNGNSSMATSIRTSNLFTPRSKEPVIFRYRAFQRNSSIASSETSVDAVNREKTIINDFIKQRLVSIMKMHAINIGLIKKIENIFQSAIAIEFCLLSGGLIAELLGGLENTYVEVPFALVQVAMDCITGQKLIDANTAFAEAVYDCQWENFDVSNMKLVMVMLQNAQKTMKLSAGGVNHLNYSTLMSVIKSIYSAYTALRSTMNKHN

>EposOR39

MWKTVRKFGLSHHDLPTMVWNVAVMLRVVCVRIDPRNTKPIPPALYILTGVCAICYLYIYQISMLWFVFVRCKETGDVVAAMMLLSTGTTSLIGITKLFYMYLYKERIQSMVAAYIEYDRLPVAPAALSLVDTSLRDVKKRALIFGAVIIGNGFVYNLQPLFKSGRHLMDDDQILYGLEPMLETPNYQIALINIVASVNFICFIVSNITGLLIITTGYSEARFHALSLELRNLWQDAKQHYKENFDVDYKEDKYDKRAETELNKYVKHRLFHIQKSHSTNIELVKSLEDIFRGAIAVEFFLLTLSLIADLLGGLEHTYIIMPFALMQVGMDCFTGQRLMDASLMFEAAVYDSKWEHFDAANRKTVQLMLQISQKTLILSAGGVTALSFGCLMSLIRSVYSAYTALRSSMSF

>EposOR40

MDLDYDELFKISIWALKINRSYPTIPNDKIWYITMIPMHGLFFVEFCLLLNSTICHDLKNDNFSAACTNGIFAVLFNCVTFKYTMLFIKKQDIITAINKIKGDYESAKHLPVEEQEIVVQYAKIANWVTKVWFMTSCVSFSVFPVVTIVQTVNYCWIGEPKLVYLYDMTYPEAIETRKGDVPVYLFLMFLQIYYALYTVLMYVGFTPLGLIFMLHTCGQLEIVKYRIRNLFNGDHYDPREIRERLKNIVMPLQDVLEYVDLLKETFRVIYEMYMKATTIVLPIAFYEIVESFKEGQASFEFMTIIVASVVLCFAPCYYSDLLMEKGESVRLSVYTSGWESHPDISMRRTLLITLSRLERDVAIRTLFRTVNLDAFAELCHQSYALFSVINAAWN

>EposOR41

MDAMDLRYMKRLRFSLMSVGAWPDHVFKDSTKLSVFKKFGFSIILFIICCVSIVAQLAYLIINKKVLAFIDIGRSLVPFLISFVYAERTTLPMRSSYRSVIEEFVLKFHLIHHKNKSEYTMKMSNKVNRICEIVTIVQHLQLYVAGFLFWVVPLCQNYSTGMLSSDRPANVTFRHSVHYALPFDQNSAITITMVCIFNWVASYNVGCLLCCHDLQVCVFVFHIWGHLNIIKHNLNNFPRPRVPVNAKSVPLRYSNEESKDVAVGLKEIIQHYIMTKDFVSKISNAYSISLCVYYTLHLVCDCVLLLECSTLEPEALATYAFMTLVMFQQLIQVSVVFELISTKGDSLADAVYGLPWECMDNSSRRTVLILLQIVQQSLAVKACGMVPVGVQTMLAVLKASLSYFLMLRTFANS

>EposOR42

MDIPTFKEVFKILKINFSLMGIPLNKSMLTIKFYVLIFLLVLMIIEEASFFVKMMAPENFLQLTMLAPCLCVGCLSILKIIPVAIHRETVRDLADKLNNLSTEILNDPEKKKVIQQDITMLQTLIKYYFILNLVLISVYNFSTPFYILYHYITTREEIFYLPYSVLVPFSTETWCNWCIVYIHSIFCGFICTLYFTTVDGLYFVLTSYVCSIFAVLSKDIQELKNASDATLKDIVKRHQYVLELADDLELVFTLPNFFNVLVGSLEICALGLNLMIGDWGDVPGCFLFLSSVLLQIFMISVFGEKLISESTKISDSAFLCNWYEMNSTSKKTILVLMTRARKPQWLTANKFSVICYTGFSKIISNSWSYFTILRTVYSREE

>EposOR43

MDALELRYVQRIRITLGTVGAWPSHVFEDNPSKKWAVLRRYGYTSLLCILCSLGMVGQAMYLKENKSTLQFIDLGQTCLTLLMSCVFCQRTTLPLLRTYRETMKDFFLKFHLSHHKYTSEFSLQTCNRVNRFCEIATIIQHVQLYTSAFLFNIVHHYQNYKSGMFSSDKPINGTYEHAVSYYLPFDQNTGVWFTVICIYNFFVSFNLFCCHDLVICVIVFHIWGHMHIFEHNLNFFRRPNAVNGVESLKYSCEENKQVELSLKEIVKQYIMIKEFLSNTSEAYSVTLCVYYGFHLVTDCVLLLECSTLDPSALATYGMTTFVVYQQLIQLSVVFEIISSKGDALIDAVYGLPWECMENSSRRTVLILLQIVQQPLSLKTCNMVPVGVQTMLAVIKASFSYFMMLRTFAN

>EposOR44

MKILNEYTKEKLSFLTPLLPYGVLESWDDLNPRLYHAVHIYWLKFYGLWYGTVSTCSVQFWTHLVYGLLVLWLVCFLPGIGEVVYLLRRRDNIGDIAEGLYLFLSEMYTYIKLSALWLNKKKIIDLLEYLHREEFKPSEVEHREILRKSIKTARFVMTYYSTICVGAVSVAIIMPLAEDFEVLPTNVEYPYFDVYKSPAYGIIYIHHIYYKPATCIIDGVMDTILSAFIASAIGQIEILSFNLRNFNMIAERRRQRLSRMGNLSNDQSTRYYVRSVFKDIIKHHNSIIRYVSLIESAFSLASALQLMLSVMVLCLVGIQFLSIEEPMSHPIQIAWMAIYLTCMLIEVFIICWFGDELIWKSKELSQAAFDAPWPTTDPKTAMFIIIFMERCKRPLRVTAGKIFTLSLDTYTNLINWAYKAFAVMRKMKK

>EposOR45

MDAMDLRYMKRVRFTLRSVGAWPDHVFSGCPTTKLSVVKRFGYTTFLLVTCCLSIVSQFAYLMKNMAVFSFVDLGQSVVPFMLSLVYAERTTLPMRRTYRSVIEEFVLKFHLIHHKNKSEFSFKMYNRVNKICEIATLIQHIQLFTASFMYNIVPFYQNYSSDMLSFERPENVTFKHSVNYLLPFEQNNGFVYTLVCMFNYFVSYNLGVLLCCHDLQICLIVFHIWGHLKIIEHNLNHFQRPSIAVNAKTVPLRYSNEESKDVALCLKNIIQHYIMTKDFVSKTSRAYSISMCVYHGLHLITDCVLLLECSTMDPNTLATYVLLTLVMFQQLIQLSVVFELISSKGDSLADAVYGLPWECMDNSSRRTVLILLQIVQQPLSLKACGMVPVGVQTMLAVLKASFSYFLMLKTFSNKNEHRTM

>EposOR46

MIFEKNINLSVSLSLTALKMFGFWAPDGLSREKRLLYNCYAFISFMFLLGTYLIIQVVDMFMIWGNLPLMTGTAFLLFTNLAQATKIASLVVRRRTVGAIIDDADLLLRGEEGRGRDIVKSCDRETSLQQLLYFCLTTVTVAGWAGSAEKNQLPLRAWYPYDTSKSPAYELTYVHQVGALFIAAYLNVGKDTLVTALIAQCRCRLRLLGLGLRTLCEDLVPGEQGMLTPEQEKTAWSRLRVVVQRHQAALEASSLLQECFSAPIFAQFLVSMVIICVTAFQLAAQTGNLVRLFSMGTYLLNMTFQVFLYCYQGNQLSEESIEIAGAAYSCPWYSCSTKLRRGLLVVMVRTRRAARLTAGGFTTLQLTSFMAIVKTSYSLFTVLQQAEESKT

>EposOR47

MAEAIADVQPKRYFAIHFYLLRFLGLGWWHHPDEDNHNNFPGLYIYYTIITEIVWVAGFVGLETIDPFIGEKDIDRFMFSLSFVITHDLTIIKLYIFLFKNRAIQDIVRTLEIDMYNFYQNNERNRATIRITRIMTGAFVFFGWITIGNTNVYGTIQDIRWKAEVAQLNDTSLRPPRTLPQPIYIPWKYQTDASYISTFVLETVGLLWTGHIVMGIDTFIGSVILHMSSQFVILQEAFITSYDRALSQLVTEIPQSRNTVENIVMPIENGDNAHEDSLDKMEAKVKSFYTDEQIEIAIEKTVRNCILQHQVLISCVEKFRMTYSYGFMTQLLSSMAAICVVMVQVSKDASSFKSIRLVTSLAFFVAMIIQLALQCFTGNELTLQAERVSDAVMQSKWDRMTPRVRRYLLMAMMRAQRPLRLTAAGFAYMDNGCFLAIMKAAYSYYAVLSQKEV

>EposOR48

MLKEFLDRLRTKEMPLLGPNIWCITTIGLRLPKSRSGKILCFLFHEIITFFVITQYIELFFITDLDTMITNIKSSSLSIICVMKSNTMLFWQDKWQKAFDYVTEADIFERTNGNPARDKIVANYTKQCALVTYYYYILLFFTNFGVISLNLIANLSSEEFRVALNNGTVNFPHIFSAWTPYDKNKDPYTWITVVWQAYICTIGAVIISAYDTTAVVLMIFFGAKFELLRLRCEELFKEDEVVTEEEFDGRLRQLHSLYTHMMENIRLVNSLLSPVMFLYVVLCSLVFCTSAFQLTFATSISQKVMLLEYLGFGITQLFLYCWISNDVLEKSSKLMLGPYESAWWVGSPRQRRAVLMLAVQMDRAYVFSAGPFTDLTLPTFVAIVKGAYSYYTLLRT

>EposOR49

MLKNFLKRFEDPSTPLLAPNLKCLRFVGLLLPESKIWKIIYILIHALQTLFGITQYVDICFITSDKSLLYNNLKTTMLGTVNVVKALSFLMWQKHWKDIINYVTKADLEQRRSNDKENHKIINRTTKYCRKVTYIYWSLEILTAVFVIMSPILKYILSSTYRENVRSGKEEYLQMINSWVPWDKTTFTGYAFACVYQTYAAILAVAWLSGFDATAIVVMVFFKGEMELLRRDSSKIFGSDDDPVGVEEAKRRLMKCHERHQEMIKFARLFDSCLSPCMLLYVIVCSIMLCVTSFQITTEKSPTQRILIAEFFVFGVAQLFLYCWHSNDVFYSSQDLYLGPYESAWCLKPVHRKDLMLLMVQFNKTLVFSAGPFAKLGMPTFISILRGAYSFYTILIKSYHEE

>EposOR50

MLKSFLAGLEDPKRPLFGPNYWFLNKTGLLLPENLFKRILCIIIHEIATFFVISQFIELYMIRSNLDLVITNLKISMLSIICVIKANTFVIWQDNWRQVISYITESDNFERENQDESKGKILSAYTRYSRRVTYFYWALVATTVVTVTTTPIMIWYSSQSFRDGFWNGTEPFPHIFSSWMPFDKERSPGCWFTVLWHIIICAYGSTIMAAYDTSAMVILIYFGGKLDLLRLRCQEMLGVGEGVTDQEADAVVRQLHSIHVLLLKHSRVFNSVLSPVMFFYMVMCSLMICASAYQLTSATNTTQKVLMAEYLIFSVAQLFMFCWHSNDVINKSQNAMTAPYESRWWTAGLHQRRSVLLLAGQLRVVHIYSAGPFTDLTLPTFITILKGAYSYYTLLNKTTKDTN

>EposOR51

MADTLKATHPQKYYLRVVSRLMFYFGFGTYWYEGSPTRYPRLYAAWCILIQSYIFIVVMNLVMAIIRPDLREEEKSEVVQLGFSQLLVVLKLIIIILQRHRIRAGFKKLLEEDRDIFCSLEVEKQSVKTAKAFFLPFIIGSYSHLSTTLVRWIFVSVMNGTPIQTQITYFPTPALAGFPYNVLRILIIAHWWLYVTIMITSDCLCCLPIFFIAAKFKQVRMYFENLGESDLREWTADDFMKAIVNGIKLHQNALWSASNIQSAMGILYGVQILLTVILAGISLFQLASLERTFMNMASGAVYIICISFLTGAYMINSGVITYEAEAVATAMFHCGWQRVPAERRLRTLLVVAIQRAQMPVYMTAFGVINLSHESYVTVLRAAYSFFALVY

>EposOR52

MSSLEKLADGLPLLVSVAVVIYFALYKKELYELVEFMNGNFKFHSARGLTNMTMDRSYKSAKNFAFVYTACTLFSVTMYVALPMIIHLWTKQPLQNWIYADVDATPYVLMAFLNQCLAQAFVGLAVGQLGVFFAANAILLCGQLDLLCCSVRNARYTGLLQTGVQHKALLQEHAGIVDDERHNYIYSESEARDSEYHYDTKVTSYFVDRRSQFDIYSAEFDAATADALRECARVCQVVAAYKDKFEDFVSPLLVLRVVQVTLYLCTLLYAASVKFDMITVEYLAAVALDIFVYCYFGNQIILQASRVSTAAYQSVWPAMGVRPRRLLLNILLANKRPVAVRAGRFLPMDLHTFVIIIKTSFSYYTLLDKINH

>EposOR53

MKPVSECFKISFFVLIVAGVWYPPSLENSRYVRLVNIYRVLAMITICIGMLSIQFVYFFTVVGVDLDKTIDATALFTFVGHLFKALTVIKNRKRINELXKTIDDEEDKDELMQNMATKLSLVSKIYNGGAGLTAVMWNLIPFTKPTLTLPFYYPDLPSNSPWFVSWYTYQSIILIINGVAQTSADHVFGGLMAFAATQLKLLQHKLETIGRKETELDVTADQKEQDDYNKAVSCVRFHLKIIRVVDELTSIFGAAAFGQFVLAAPLLCLSAFILTTSSDRTEIMTRLLYFGCISGQLFFYCFCGNMIKTESDRVATAAYNCSWESTSVRTQKTLKLLILRGQKTMSVVAGNLFELSMVTFGALLKSSYSFFAVLNKKHDD

>EposOR54

MTVVETMKNSHCLSSSITVMQYNGVWKPDGLTHRGEVAYKIFGVVTQVFLFYFMILAEIGYVYTFLDNVERVVDAAVLLFSHLVQAVKVLTIILRQERIKRMIQVADGPAFTKSDPKLKAILEGTTSLAANIGNMVLLSAFTTGIFWSIVPALNPELTLPLRTAYPFDINGPYIFAAMYAYSTISVTVSGVGDAAENFLVSGMFTIASAQLDVLREELEAIGVEGYSAENFEKATRCVQYHQRIIGYVEEMADIFGLPIFCQFVISSVVICMTIYKITVTKEPVEMVTMVFYLICVFMELLMYCYPGDVLLNKSLLISDAAYPNDWSGDRKTSRVLLLVAMRAQRPLVIDAGGLFRVCLPTAAAVVQTAYSYYALLRQQLQD

>EposOR55

MFRETYTLVLKYFKNNLIDVLDEMCWLRKLFWLQFVVYILAVVSHTGGVLERMADGADMVQLSGDLSATLVLWQVSVLYVQIYVNRKLIQKLILNIGAQWCSDDNLSPEMMEVKHQSVKSIHKWITYFYKILICFMHLYFCIPLFVTTVRHFVLKENFPFATIYKLKMPFSYEDNFLLYWMVSMADYGVLYNTGFLITSDLLLVNVSMNHLRTLFVILQDDLKCIVHSSAPFEEKTATSRLKEIIPKHANLLQLMVELSEAFGAIFLIHLAFFSGTMCFFGFAARVHCSPESIKNLLASSFILICIYSCCSYGQDLTDASLDVANAAYEGPWHVMSHEYRICILFIMLRSQKAYYIRSTSFTNISLQSFTKILNVTWSFLSLIDKVYED

>EposOR56

METLRRFGLKHHDLPTMLWNVTALLRVLALDVDGRNRRRIPIFVYLVTLASIACYVYVFVVSACWFIFFRNVQVELVEKIIIFSLGTSSTIGVTKFAYMYWNKKQLQDMVAAFLLCDSYVTPYSRMSRSIATTLRDVKKRAMVFWITIVGNATIYILVPFIIPGRNLPDNNQVIWGLEPMFDSPNFEMAHVIILLCVPIVAFAAVNITAFLIVVVGYAEAQMIALTQEMLNLWEDAQIDYKQKALEINHFVKDDSKQGTAINYFVKHRLESIMKTHAINIQLVKQVEDVFKNAIAWEFALLSSALIADLLGGLENTYLTIPFAIVQVAMDCFTGQKLLDANKAFETAVYHCKWENFDVSNMKLVMVILQSAQKTMKLSAGGVTCLSYMSLMSVVRSIYQAYAALHSTMNKG

>EposOR57

MYPSFQAFRQNFDALARVGYFKIVSKPQTSLKQSLHDAYRTAVWVLVLTYNLQHVIRVIQSRHSTDQMVDTLFVLLTTLNTLGKQIAFNARSARIDRLVQIIEGPLFASRNVYHEKIMKAHALEMSRLLKLYHVAIYVCGFMFGLFPLVNRILGEEVEFTGYFPFHTNDVLPFAIALSFNTIVITFQAYGNVTLDCTIVAFFAHSKIQLQMLRYNLEHLVDRNWAELDAKVNNTSVPRAFKDIEDAAFGELLRKRVAHCVEHYKLIVWFTNEVETVFGEAMVVQFFVMAWVICMTVYKIAGLSLFSAELVTMAIYLCCMLAQLFIYCFYGTQVKYESEFINHSLYCCDWVSLSPRARRLLLVMMARCGRAVAPRTAYIIPMSLETYIAVLRSSYTLFTFLERKS

>EposOR58

MQPLDVAETYRTNKTVHFFRKICKIIYLSCATNFSFEEMKLPPLFIKIYSPLSKILEVIVVVFIASEWGSYFTQNNLTEKQTADMHLFAFSHIVIYSIYASAMYHKDRIRQLVLTLTVTLKKVCNDEEIERMMIKKTYWYLTAMXFXCSGSLFSYGLDAGVQALTSNATFTTVIPIWPDVEDRRFIASVARXIYYIIWWIFVTRVISIYFIVLSITICLGHQFTNLHKYFLDLXDIFEEVGSQEDKERRYEDAVKVGVKMHSITLWCAEQAQVTCGVAYSGQVIINVSVLVLLMIQMVNTERTLSGVVPIVMLGSSVLVGSAVFMLNAGDITIEASRLPTAMFLSGWQHCRGPASVRARRLITIAMAEAQKPVVIKGLGVIELSYQSYVSIVKSSYSLFSVIYSY

>EposOR59

MASKVEEARGEINETLSLCLFSMRWIGLSFEPPASTRAYLRQKLMFIASVCAIVYHVFSEIVYIGLTLSNSPRVEDVVPLFHTFGYGALSIAKVFALWYKKDVFAEHLQELSGIWPMPPLDENAQLIKETSISALRLVHRWYFAMNMGGVLFYNVTPICVYFYELWMGQDAKVGFVWVSWYPFDKYEPTNHVFVYIFEVFAGQTCVWIMLCTDLLFSGLASHIALLLRLLHSRLETLAATEKTNEEYYKEIIGNIKLHQRLIRYCNDLEEAFTIVNLVNVVLSSVNICCVVFVIVLLEPFVAVSNKLFLGAALIQIGMLCWYADDIFHSNADVALAVYKSGWYRTDPRCRRALIFLIRRAQKPIAFTAMNFTNLSLVTYSSILTRSYSYFALLYTMYNNS

>EposOR60

MSQTDDTSRREIHRTLDLCNFCTRMIGLSFIDEPPTSVFAKIKTTSIFVISALALILLVVGEIAYVTSLILRTTSVAEFVTSLHIVGYGSMSISKLLTLWFKRHTFRKLIEDLEDIWPVAPENQEEEDIKQKSLKDLRLGQSWYAFFNVLGVVMYNITPIGIHLYRRSRGMPSVMGYVWHVAYPFDKTQPINHVFVYAFECFAGASSMWSMVSSDMIFTTMASHIGLLLRVLQVKIRRLGLTATDGDASPSTELLHTGYYQEIVNLIKIHQRLISYSDDLEETFSLVNLINVMLSSVNICCVMFVIVLLEPWMAMSNKFFMGAALTQVGILCWYADEIYRASVGVADAVYASGWYRGDARSRRALVVLIQRSQKPLYFTALKFRPITMTTYSSIITTSYSYFTLLYTVYRSD

>EposOR61

MDFVTTLSSRICLYPFHNRPKYKLFCFYFICFLIFFVTAQQFAALCIYGFKSFLDIVSIAPNIGVSIISVTKYLKVHRNKEIYESIFNHLRNNMWDIVDKKSQETCKILRRYHKVMTFIILWYTYYVVPLILFVVTFPLLIMYYDSEILGRDLEYRYLFEAWYPFDKIKWYYAAYAWESLMTAVVVCIYTFSDLLNVSFVGYICMELRLLGTHLRQLIEPKDIMNLKRSHDVTAVHEKIKRRLKSIILKHVFLANVVSQLDEILGDIMFVNYTLGSILICLTAYTFTVVDEFYSTVRFFCFFISFMFSILHQCVMGQVISDHSEALVEDLYCSEWTYCDRDTKKLVLLFIMRMQNPFQLTAKNYIIMNLHTFTTICSSAYQCFNLLRTMYDPKLN

>EposOR62

MALLASLWRRLTPTKALEDSSGKLETAFFESVYRVIYITGLSHLELGIGYRLYSSWVKLMMSLFAAGETWYLVSSYQLVDIFIEQLNVMVIQLTALIRFKSKRDHERLYKKLATSMELSNYDTSTPARSALVELWRLRSEKYLKLIYALGTCTLIMWFVYPLIDDVECNLMVAVRLPLDYCTPVRYPIAFLTSMVAFCFVAYFVMTNDVIMQAHLMHLLCQYAVLIDCFENILVDCERDFQGVSRNELIHNNDFRTKYLERLGGLVDQHKQLLNHTMELRKILSAPMLAQVMASGMQICFAGYQVTMTISESASKFLMSFLYLGYNMFQLFVVCRWCDEIKTQSVKIGDALYCSSWERGLTTIPGVRTRLMLVAMRAHKPVVLTAGGLYDLSLSSFADLVKSSYTALTVLLRLRDD

>EposOR63

MTLLASLWRRLTPTNALGDSSGKLETAFFESAYRVIYATGWSCYDDRIGYSLYSNTVKLMIALFAVGEAWYLASNYQSLDIFVEQLNVMVIQITALFRFKSMREHQHIYKKLAATMESSTFDTSTPARQALVEYWRLRSERYLKLILGLGICTLAAWFVYPLIDDVECNLPVSVRLPLDYCSPILYPVAYLTTFTAFNYAAYFVMTNDVIMQAHLIHLLCQYEVLVDCFENILVDCEEHFKGVSRNSLIQNKNFRLKYLERLGQLVDQHKLLLNHTMELRKILSLPMLAQVAASGMQICFAGYQVAMTITDSITKFLMSFLFLGYNLFQLFIFCRWCDEIKTQSEKIGDALYCSGWERGLTTIPGVRRRLLLVGMRANKPLVLTAGGLYDLSLSSFANRTTRYGDCYSKTNVLVLVLLD

>EposOR64

MTIVQNARSFVYKEGFDWDKPEITLQFFHPQLQMFFAVNGIFFNNRESKIRFVWPLLSALLAMIANCFELMFMWHGFEIKDYGFVTECFCYFFILGSVVIVYISMLRNRDKIFMLSNNMNKDFLFICNLGSQYRDTFFKGQLLIWKLCWSWLCFAIFVALLYISNTLLYLLYQSTLATQDEHMVRPFMFPIWLPADDPHRTPNYEIFLALEIVLIFIVFCSFVLYVYILFHLLLHYYNLMDMILIAFGELFDGLDEDVATLEPEDPRRRAVQAQLNSRMGRIARWHHSVFESVETVSSVYGPALVYQTMFSSVVICSIAFQVAEQLSEGKVDYLFAVLGIGACLQLWIPCYIGTLLRNKGFSLGDRCFYCGWHGTSLGRLLRRDLVIFIQRSQEPLAIKFTALPHLKLETFSSIMSSAYSYFNMLRQYNS

>EposOR65

MTIIQNAKSFVFKEGFDWDKPEMTLQVFHPQLELFFAVTGIFFNNRDSKIRFLWPLLSSMLSLVAGSLEIMFIWHGIDIKDYGLATESFCYVFILNSVIIVYFCMLMNRGKLFMLLHNMNKDFIFICNLGPQYRDAFLKGQLLIWKLCWSWFSFAIFGACLYICNTLLYLLYQSTIATQDEHMVRPFIFPKWLPADDPYRSPNYELLFAFDITLIFVVLCSFVMYVYILFHLLLHYYNLLDMILIAFGELFEGLDEPAAALPREAPRRRAVQAELNRRMGRIARWHHSVYESVDTISSVYGPALVYQTMFSSVVICLIAFQVAEQLSEGKFDYLFGILGIGACLQLWIPCYIGTLLRNKGFSVGDRCFYCGWHGSALGRLLRRDLVIFIQRSQDPLAIKFTALPHLQLETFSSIMSSAYSYFNMLRQYN

>EposOR66

MTFPKYQKTKTTEFFFNLNRVIFFCSAINFWVZEIGIPTLFVKIYQNLSKLIHVAAYLFIAFEWGAFYTQNSLTEKQKADRFLMSFSHTILYSFTIVITHHQERVRELLFTLAVTLKEDFNDAATERLMMKRTKFYTSAFVVICGNALIFYGIDGIVQVAYSDGTFVTLIPFWPDVGDHRAVAGAARIATYVFWWIFMARVTSAYLLVLAITICLSHQYINLQLYFKSLEDIFKQKYLTQRIKEGRYEQALKVGVKLHATTIWCTQQVQNTCGMVFSGQIIVNITVMVLLMSQMVNSESTMSNTLPIAATIVSMLFSTGLIMWNAGDVTVEAARLPTAMFLSGWQHCQDKASRRIRRLLLIAITQSQKPVIIKTLGVIELSYQSYLSIVKTSYSIFSVLY

>EposOR67

MDYLKESPLEFSKPFAICFDLLAKANITIHDQKRSIRSKIRSLLLLVCYVTFYVSLAISFSKVFTGELGFYELANLLPIIIVATQGAMKGIVIITNLTKARTLIYELGAMWRTTGLTRKQLERKGLMLKRLNLCNAVFYWMNIVGTWQYILVPLFETLFRTFVLGQDKQLFPFICTFPFDSNKNWLVYLVTYFYESYSMLHLIYMYLGVEFLMITLCSHLATEFELLREEILHARPILETVDVPNDSRNDINICDDDIDHDIMIINDAEDDIRLDEDKPDIKDVIRRHQKLIMLSETLDDIFNKMIFFNLLFATITICFFGFVAKIARDPPEMANNFVGVVASMIPIFNLCYYAEMLSGASAGVADSAYHNLWYEGDLRYQKIIIFIIVRSQKACSLTSMRYSPVTLNTFTTVLSTTWSYFSLAISVYETDKQ

>EposOR68

MPSTLAGSFAKHLRIFKIIGLDFLGDPCMEHRCRHVSFVVMLLLFYTGQLLFFYKSDEIGADFLDIANAVPLLMLATQDLFKIVALRKKYRIKAIILEIAELWPNDVENEERKYIMDSWLRNLKMFNDCVYKFVAFAIIVFVWVTFVITVFSSGDGETTYQFPFMLYYPFEIDSLWKYSAAFLYQAVDGSIIHLCLYLPCDLLLFTLTVDICILLRLLQYDLENIRVVGKDGKGFHNPAEAEASYRAVAQLARTHQKLVQISESLNEVFGAIIFAAVSLSSVILCFFGFLSITVSGSQYQMLRSFLAALVTLFIVFCLALPGQILSDASCGVADAAYKSLWYESDLKFRKIVVIMIARAQKPCQLSAMGYSIMNFNTFCKVCSTSWSYLSLLNQMYQDNER

>EposOR69

MGSREVALFDENLKKANEVLKYIGLRTDDEESHQSFVERARSKWFFWFFNLTLYSELLGELAWFVEKGIMGSTFIEATYTLPCATLCLLGSAKTYYRIKYGDLINDLIYSLRKLQSADPESGIYADPIAYEAIRKIVNKLGLAIKALTIFNTCGLIAFLFGPLAIMLFLYQTTGVTKLRFPFLILYPFNAFDLRFYPFVYFHQMVGGVFAVYTIFGPDCLYYLFCTFVQVQFCSLKYDLERIVPSRSHLRLQADKDAYKTKFEACITRHRELIRCVNLIETIYCKSALFNFMSSSLLLCLAGFNIVAYEDVAMLGNFLSFLVMCLLQIYLICYYGDNVSRASVAISQAVYNSEWYNLDAKTAKDLRMVLIRSQKPCKLTAYTYTEINLNTFTRILSTSWSYFALLQSVYSRN

>EposOR70

MGLRQLAVFDANLEKANKVLKYIGLQTDDEESDQTFMERIKSKKFFWFFHLMLYAEVLGELAWFVEKGVIGNTFIEATYTLPCATLCFLGSAKCYYRIKYGDHINDLIQALRELQSSESEFGIYADPIAFEAIRKIVKTLGLAIKALTIFNTFVLIAFLLGPLAIMLFIYLTTGVVELRFPFLILYPFNAFDLRIYPFVYVHQVVGGMFAIYTIFGPDCLYYLFCTFIEVQFCSLRYDMERIVPPNLRRQPETDAYRKKFRACFTRHTELIRCANLIETIYCKSALFNFMCSSLLLCLAGFNIVAYEDVAMLGNFLSFLVMCLLQIYLICYYGDNVSRASVEISHAVYNSHWYNLDAKTAKDLSMVLMRAQKPCKLTAYSFTEINLTAFTRILSSSWSYFALLQSMYNRK

>EposOrcoMGKVKTQGLVSDLMPNIKLMQTVGHFLFNYSDETGGMSMLLRKVYASTHAVLIVINFLCMAVNMAQYSDEVNELTANTITVLFFAHTVIKLLFFALNSKNFYRTLAVWNQSNSHPLFTESDARYHQLALNKMRRLLYFIGTVTVMAVVSWITITFFGESVRLIADKESNDTLTEPAPRLPLKAWYPFNAMSGTMYIVAFVYQIYWLLFSMAIANLMDVMFCSWLIFACEQLQHLKAIMKPLMELSASLDTYRPNSSELFRASSTEKSEKVPDPVDLDIRGIYSTQQDFGMMLRGAGGRLQNFNNPNPNNPNGLTQKQEMLARSAIKYWVERHKHVVRLVASIGDTYGTALLFHMLVSTITLTLLAYQATKIDGLNVYAFSTIGYLSYTLGQVFHFCIFGNRLIEESSSVMEAAYSCQWYDGSEEAKTFVQIVCQQCQKAMSISGAKFFTVSLDLFASVLGAVVTYFMVLVQLK

>BmOR1

MLLSFKDDSRSPDIQKPQNFQYMKILRFNLKIICAWPEKQLNEIRSLGHSIHRVILPIQSVVCLACGILYIHFHFNEIPFFILASTFITVMMNLATCSRTALVMLFERYLVLTGRFITVMHLFNFQKNSDYAYKLCTFVNRMSHFYTLYVLFSMFMGLGLFNLLPLYNNYVSGAFSDPYGPNVTFFHSVYFAFPFDYSHNFRGYIIMALFNSYVSVTCSIGLVMFDLLMCLMVMHVWGHLKILSHNLINFPRPKASHVITTPNGPTNVETYTEEESKEVFARLRECIKHYGTVDDFANDMSETFGVILLVYYGFHQVSLCMLLLECSDLSTKAMLRYGPLTLIMIQQLIQISIIFELLGSVADRIPDAVYQLPWECMDVKNRRVVYGFLRRTQNPVRFKAMGMLDVGVQTMASILKTSISYFVMLRTVAT

>BmOR2

MMTKVKTQGLVTDLMPCIRLLQAAGHFLFNYHADTSGMNMLLRKIYSSAHAVLIVVHYICMGINMAQYKDEVNELTANTITVLFFAHSIIKLAFFAFNSKSFYRTLAVWNQSNSHPLFTESDARYHQISLSKMRRLLYFICGMTVFSVISWVTLTFFGESVRMIASKETNETLTEPAPRLPLKAWYPFKTMSGGGYVFAFIYQIYFLLFSMALANLLDVIFCSWLIFACEQLQHLKAIMKPLMELSAALDTYRPNTAELFRVSSTDKTEKVPDAVDMDIRGIYSTQQDFGMTLRGAGGKLQNFNAENNPNGLTAKQEMLARSAIKYWVERHKHVVRLVASIGDTYGTALLFHMLVSTITLTLLAYQATKINGINVYAFSTIGYLVYTLGQVFHFCIFGNRLIEESSSVMEAAYSCQWYDGSEEAKTFVQIVCQQCQKAMTISGAKFFNVSLDLFASVLGAVVTYFMVLIQLK

>BmOR3

MIFVDDAVIGIKDPREYRHLRVLRTSLRLLGAWPGHYLGEETGSKYECAPMFLLMFIKIACLYLTIVYLRNNADVLGFFELGHVYLTIFMTFVTLSRGFSLTWNPNYHKVVKKFITEMHLLYFKDNSEYAMKTHRRVHKISHFYTVFLKVQMIAGLTLFNVIPMYNNYRQGNYASDRPANITYDLSIYYETFDILNTPNGYIFICVFNWFASYICCSFFCSFDLILSLMISTVSGHFRILIHNLLTFPLPEAITASKKFVDKHRCNGNRSEFVLEEAKLYSPAEMWQVTDRLRQCIDYHRKLVEFTGDISEAFGPMLFVYYLFHQVSGCLLLLECSQLNTAALVRYGVLTVVLYQQLIQLSVIVESVGTVTGRLKDAVYEVPWEYMDTSNRKTVAIFLMNVQEPLHVNALGLAKVGVQSMAAILKTSFSYFTFLRTVSE

>BmOR4

MFKIIKNIIVENDALKQVEKPQEFQYMKWVQYHLKYIDGWPNMDMNKKNVSKIRFHKRHLLVVEQTITFLSQMFYIVKNYGKLSFFEIGHSYITALMTIVIFSRSVVTALGRYRKIARYFVSSLHLYHYKDISEYALQTHLLVHRLSHYYTVYLISLVVTGMLLFNITPLYNNISSGVFNSPRPENMTFQHAVYLGLPFDYTTDIKGYFVVFILNWHLSHIAASYFCTFDLFLSLLILHLWGHLRIILNNLKTFPKPYTNNSMYTEEENQVVLLKLQECIRYHNFIISFTVMMSNVYDVVIIVYYLFHQVTGCLLLLQCSTLDWESLSRYGPLTLIIFQQLIQVSMIFEILGFLSDKLPNAVYSIPWEAMNVTNRKLVQVLLQKSQKPIQFKAMNMMSVGVQTMASIIKTSISYFIMLRTIARD

>BmOR5

MLLYYPNTQVKEKVNNVEEFTYIKFLKSFCKIMDFWPEREEKNSKTRIFRLRYILVLQFCFTLVAGVLYLTNSVGKQTFYDLGHTIITVLMNVVSLSRLILRCFKKYDVVGQQFINKIHLYHYRNDSEYAMKIHTVVHKISHNMTYIFSFCIIFGTVTFNLTPIFNNIGSDAYKNPRPDNVTLQQCVYYALPFDYTGNFKWYLLVAIFNVQKTFFCTSLFILFELSLSLMIICLWGHLRIFIHNLNHIPAPRNSFEYTKEERQEVDDTLKKCIQHHTLIIGFVRIMSETYGLAVLIYYAFQQVVGCLLLLQCSQMELKTVTRFGFLTLVLNQQLIQISVIFELLGYMSDKLQDAVYCVPWEYMDTSHRKMVYMMFRQSQIPLQLKAMNMLSIGVKTMVSILKTSVTYYLILKTVTTD

>BmOR6

MKEEYYLQHPRTQLFYKVLAHVSTIESTIDLTWWGYTFPKYVGWFYHLQCNVVRLFGKCVVVSQILFIILNYQTIDKSVFIIAITITPLGALVGIKAESAKAECYVNLMKNFMDKVHIHSIYRKNENNEFVKKKVIQIERVSRFTAYFLVILIAINCLSWMLKPTLHNIKHFEEIMNKSMEFQYYIYFWTPLDYKYNLRDYIIIHTLCIYLGATAVTVIVTFDIFNFIAVFHVVAHIQILKNNVKSNWSDDFNESEKKGYLVSILEYHAYIIRIFGEVQSAFGLNVASNYLQNLIEDGLFLYQIMNGEKENVLMYGLMIILYLGGLIFLSIVLEEIRRQNYDLCEYVYALPWEGMSLENQKIFVVFLQRTQPDLEFETVCGMKAGVKPAFSIVKSMFSYYVMINSRF

>BmOR7

MLLYHPNTQVEEKVNNVEEFTYMKFLKSFCKIMDFWPEREEKNSKTRIFRLRYILVLQFCFTLVAGVLYLKNNFGKKTFYDLGHTIITVVMNVVSVSRLILRCFKKYDVVGQQFINKIHLYHFRNDSEYSMKTYKAVHKISNNMTYIFSFSIFVCVVTFNLNPVFNNIGSGAYKNPRPDNVTLQQCVYYALPFDYTGDFKWYMLVAIFNVQKTFFCTSLFILFDLLLSMMIIHLWGHIRIFIHNLNHIPAPRNSLEYTREERQEVDNTLKKCIQHHTLIIGFVRIMSETYGLAVLIYYAFQQVVGCLLLLQCSRLDLKTITRFGFLTTMVNQQLIQISVIFELLGYMNDKLQEAVYCVPWEYMDTSHRKMVYMMFRQSQIPLQLKAMNMLSIGVKTMASILKTSVTYYLMLKTITANEA

>BmorOR8

MSLSTRCLLKDFCKYVYYAGAGNFWYEDIYKETVPYKMYVVISFFTYTVMIFLENLAALFGKLPEVEKNSAVMFAAIHNIVLTKMFLLLYHKRSISKLNCEMAAVGENLEEASIMRRQFRKMRLGTALYFISVYLSLVAYGVESARRTIVEGAPFYTVVTYLPDYDNTTVLASFLRIFFYITWLYMMLPMMSADCMPIAHLITMTYKFVTLCRHFDQIREKFQINVKIMAKTEATEILKLGFIEGIKMHQKLMYLADEIHRVFGIIMALQVCESSAVAVLLLLRLALSPHLDLTNAFMTYTFVCSLFLLLALNLWNAGELTYQASLLSNAMFYSGWYFCDFEKDWCRDIRRLVLIGCAQAQKPLILKAFGVLDLSYETFVSVARMTYSVFAVFYKRGD

>BmOR9

NVDNVEDFKYVKWLRNHLKTVDAWPVHSKSKRKIQKRYVLPIFSAACFISQTVYLKNGIGTLSFVVLVHSYICFLINGSCLCRGILIATERYKRLATCYLKTVHLFHHKNRSEHAMKIHVIVHRLSHYYTIYLISLVFVGMVLFNFMPIYNNINSGAFKSPRPENVTFQHAMYLALPFDYTTNIKGYFVVFILNWYISLVTTSHFCTFDLFISLMIIHLWGHIKILMCSLEDIEGFVPGSSFKFTIEQNRKIYLILQECIRHHQFTIDFTNEMSSTFGLVILFYYFFYQVSGCLLFACMLTNESLSRFGPMTFILFQQLIQLSIVFELISSLSENLPNAVYNVPWEFMDKNNRKMIQVLLLQSQKLIQFKATSMMNVGVQAMATILKTSVSYFIMLRTMYQEH

>

>BmOR10

MRTNAKSFLFVPSKVLTLCGVWPVEKTSIFSLIYRSIMLSSQFCFLVFNGIYIGLMWGDLKAVSDALYMFFTQTTCCSKAIGFYFNFMKIKRIVASMDDVLFTAMSIEDQATIFSHSRTVNKLYKGVLGFTGFTLVQWTVLSLIGSGRTLPFNEMWVPTDISKSPNYEITFVVELWMMVISAALFMSVDTITVATMMFSCAQLDIIMKKTQQIQEIPLSPDLSSRNRSELHEKNNGILIDCIKQHQAIVRFSELCEGTFQVHSFFHLGGIVFMICVIGFRMAGESPVSAQFWAALSYLVIILGQLYLYCWCANELTTKSEQLRDKLYLTPWYDQDVKFKRNLCIAMECMAKALTFRAGSYIPLSRAMFVSILRSSYSYFAFLNQANEQ

>BmOR11

MDEHSHFETSLNKIKVLFKYSGMNLENTVTNTYEFLNHRWVYILNHAWTLAAVTFICIGISNGQNFIEMTCIAPCVAMTVLAVSKSFFHYINENAVKSLLENLIELERTDFERTKSVQRTEIVATEKQLLNMVINVLYVLNCSMILVFDMTPLIIIAIKYWTTNKFVRLLPYLDIFVFVPYKFEYWVMAYILQIWAECIVLLFIGAADCLFFTCCTYIRIHFRLLQYDFERLTSSRRESDGLRDDEDFRETYTNLVKRHQGLIESSSILEMIYSKSTLSNFVLSSLVICLSAFNVTVIVVNDVTIVMTYLIFLAMSLMQVYFLCFFDMLMSASEEVGNAVYNCSWYTEKASTGKDLLFTITRS

>BmOR12

MTRITDVFSLNFIFWKFLGLWGKSAPSKYNMAYTVFYLFASLFVYDIFLTLNLIHTPRKLETLVRETMFYFNHLVAVTKILMMFIMRKKILVIFDLLDCEEFKPNDENSQEIMKRKTDFYYIYWRIVAVTSNLSCFMLVIGPLIKMLIWKIELGLPVCKFYFMSDELRNKYFVIWYIYQSFGIYNQMVNNLNLDTFNCGMLWMAVGQLQILKTKFVNLKLNDFENGLDLKSRDDMQIERLRKYLTHYEIILKYCATVQDILNITIFVQLGMSSIVICVGLCGFVAMPSNTETAIFMSSYLITMTMQIFVPSWMGTQISFECGELMSAAYCCEWIPRSKLFKRSLILFVERAKTPVRITGLKIFTLSLDTFTSIMKTTYSFFTLIRQLQVDEVN

>BmOR13

TSRPYKFFNILRLCTMAPKQIDCFEINWKFWKFLGIWSENKPHRYYKYYSKIFITFFVILYDVLYTINFYFVPRQLDLIIGEMLFYLTELSVLSKVFTFIIMRHKLKIIFEILESDAFQTDTEEELKILHRAKVFIKRYWKIVALVSITANLTHISSPLLKNLIFKVELVLPVCSYSFLSESFLKTFEYPLYFYQIVGIHFHMLYNLNIDTYFLGLMILIIAQLDILNVKFRNLKSGKDHTQLNESIMGLNKNLDHYNEIERCVLKHDYYYNPIPFYFNKQQTSRICLICILQSVPVEYYIFLATYMFIMIIQIMVPCCFGSRIMDKSILLSSAIYNCDWTSNSKDFKINMRLFVERANKPLSITGGKMFSLSLATFTS

>BmOR14

MSNYIFKPFHETYRIITFTMIAAMIYPNPATEKRRLIYIGLMLLSVIPLAFMIVTEMYEFFMASDLNNTIRHSTVIGPFIGGFVKSPFFEIMFVYTFFSSFIYIINYVGYDGFFGLCINHACLKMKLYCRALEDAMRSDSRRHEKIVAVIEEQRRTYEYIALIQDTFNIWLGLIYVATMIQMCTCMYHIVQSFNIDVRYIIFVISIIHIYLPCRYAANLKCMAAETPTLIYCCGWESVSDLRIKRMMPFMVARSQVIVEITAFNMFAFDMELFVWIMKTSYSMFTLMRS

>BmOR15

MMTLVYQTDIFKPNVFFWKMFGIWADRKSSKTYKYYSFVFLFITLIMYNSLLAINLLYTPLKIELLIREVIFCFTEITVTTKVLMILFKRNKILDAFDLLNKNEFRGNSEESSAIIQKNNSAYKTYWKLYAILSNFAYSSQVLGPLIVKLIWKTKLELPICNYYFLNEELRHDFFSGWYIYQSFGMYGHMMYNVNIDTFISGLLMMAVTQLKIIQTKLLSLKLNPRERKMDRGLMNITEVLKLNEILKHYELVLKYCSTVQSILDVAMFVQFGVASAIICVAMCGLIMVRSSTETLLFMVTYLFAMTLQIFVPAWMGTQLHFQSQELVFAAYNSEWIPRCQSFKRSIIIFVERAKIPITITGLKMFPLSLATFTSIMKTAYSFFTLIRNMQTLQEE

>BmOR16

MPVSPERSPHYHLGYSFQLVTICMSAYMYFGVDSVAFSSVIFGCAQIGVIKDKIMSIKPLGIYRNHKTYTNISRYNRKTLIECVKHHQAVISFTELVEDTYNSYLLFQLVGSVGIICGLAQCPITIPIAILCYLSVMISQLFVCCWCGHELSATSEELHTILYNCAWYDQDVKFKRDLNFMMARARRPILLRAGYYISLSRQSFVSILRMSYSYFAVLNQTNK

>BmorOR17

MREDKMEINNSQKFYTKMIFRYLYSVGLGDWWYQHEDRSDSHRKLYCLWAVISNAYIFLNICNELLANFRKDLTDVEKNDAIQFSFAHPLIFAKIASFFFNRKKIREVFGRLLEENRSVYSCGELEKESMKQIKRYSLAFIGVSYMTLVMSTIDGLRAHFKEGIPIRTEVTYYPSPSNSGVIVNILRFLVEFHWWYIVSVMVAIDSLAVASFVFVTFKFKLLQRYFKDMGLTVRRDQSNMTDEALADKFRRDFIVGVKLHENALWCAENVQKAFGWVYSVQVFETVALLVMCLVKLVTTNHNMIFLLANFAFMLCVIILNGSYMMPAGDVTYEASEVPTSIFLCGWELVRQTDLRFLVVVAIQRSQVPVIMKAFGIMTLSYSNFIAVSLFKFYVQFQINLF

>BmOR18

MGDRMVTRGHFFDFNIKYLFYVGLWPSNEAKRIEKIAYKIYEYQLHVLSLIFLVTTGIGTYKNHKDIIALLTNLDKTLVAYNFVFKVIVFVYKREELRKLIEQIVQSGDQITEDRKALMAKLVIVLTGISTVIITAFSCLALFEGEMTIDAWMPFDPMKSKMNLFAASQILAATFVVPCGYRAFAMLGIVCSLILYLRDQLVDLQNKIRDLRFATGNVEKLRDDFKLIVKKHVRLLGYSKVIEMIFKEYFFIQNMAVTAELCLNAMMVSVVGLEQKTLAASFLAFLSVALLNAYIYCYLGNELIVQSEGIAMAAYESSWILWPVDMQKDLLIVITAAQKPMKLSAGGMAVLSVQTYSQTLYNGYSIFAVLNDIVN

>BmOR19

MHEFVINVQNETTKLYDQLNIILYILGLQGIWVDEIKLSRRFHVFFKVVTFILHIMCGMFAGLQFFAIFTQNSLNSQQKSDVIVIGISNPMAYIFCINFIRNRNEIKDLFYHLAVVLKIYYNDVEIEKSMVNKIKSYLSTYVFASITILVSNGIIAFFQTINSDEPFLGIITAWPDKTDTSKTASYARIGFYLFWCIHFFRISTVFAVIVCILISIKYQYKFLCSYFESLNKIFDDETSSHEVKEAEFENAFCNGIKIHTQIIWCVRRCQIMCRTVFSANIMLDTFVLVILMLAMVNSENDFYGLCSQMSSVLVTVVLMAFFMWTAGDINVQASQLPDAIYGSGWYNCRGKSSARIRSLVTISMNKAQQPILMWALGFVELSHKNFVAIIKSAYSVFSVFY

>BmOR20

MIQASKYPNSKTKELFRKIAHIAYICGLPNFWIEELNLPKSFIRVYDKIVRIFNVATYFFLGIEIAAHFTQHHLTNKQKFDLLLYSISHPILNGYGVIVSRQVGNVKKVLLDLIVNLKVKYNDPVIEEAMIKISMTYSVSFITNCVLSMLTYTFDALLMVYKKGVTFNVIITAWPDVEDTTTEASIGRIGFHIFWWLFVTRPFAVYVLVINLTTCLSHQYMNLQSYFFHLEDIFKENLSQNEKEAKYEAEYKIGVMLHANTLRCTRRCHMVWNGVMSGQIIFNISLIVIIMAQMMNSDRTLVNTFGTVLTASAILISTGFFMWNAGDVTVQASRLATAMYCSGWQNCRGKSSVSIRNMVMNTIAVAQRPLVLRGLGVIDLSYQSYLSIVKASYTVFSVIY

>BmOR21

MNKNMNKNHYILKTYCDKIFLVGSGNFWYQKTESRNDKTLLYKIYSCVLFFTYGFMTVLEIMAAMMGDFPEDEKRDSVTFATSHTVVMIKFISIIKNKELLKTLNRKMMMICEAHEEQTLMDEMYRTVKINVVAYCVAVYGSATFYVFEGLRKFYNGSHFVTIVTYYPSNDDDTLAATIVRIATTLVLLMMLLTMIISVDTYTMAYLIMYKYKFITLRHYFKRLRENVDELVAAGKARLAAEKLAQGLVEGIKMHNELLSLSKDIDKAFGTVMALQLCQSSGSAVSLLLQIAVTMYLLLALFLCNAGEITYQASLLSDEIFYCGWHKCNSPVLSTQRNIRDIVLIAILRAQSPLVMKAFKMVVRSTYSVFALFYAQNK

>BmOR22

MNKNMNKNHYILKTYCDKIFLVGSGNFWYQKTESRNDKTLLYKIYSCVLFFTYGFMTVLEIMAATMGDFPDDEKRDSVTFASSHTLIMIKFISIIKNKELLKTLNRKMMMICEAHEEQTLMDEMYRIVKINVVAYCVAVYGSVTFFVFEGLRKFYDGSHFVTIVTYYPSKDDDTMLASIFRIATTLVLLVMMLSMIISVDTYTMAYLIMYKYKFITLRHYFKRLRENVDELVAAGKARLAAEKLAQGLVEGIKMHNELLSLSKDIHKAFGTVMALQLCQSSGSAVSLLLQIAVTMYLLLALFLCNAGEITYQ

>BmOR23

MRAKTEFEKTIKLTKTALFLSGINIFLGEWNHWTRTFVDSIAYYLNIVGLYFVLIGEMYWLIDGTITGKSFVELSLIVPCLTISVLATAKVHYLYHNKESLLDVVDKLREIYPDEIEETANDNDQCLNDKKETVYDNDVTEVGIVNEANELLKFVNFLLSTVSFVVTMTFCTMPLFGMAGEFMETGKFVVLYPFAVKYPFDVYNTSFWVIVYVNQFWATIIVCTNIFGVDTLFYALCSYIGMNFRLLSYKFEHLEIKRNDRIINEIIVLIKRHQELIELVNKTQSLYSLSTLFNIVTSSLLICLSGFNITILSRSWSYFALLKTIYS

>BmorOR24

MPEELFLDRSIKKIESYFRWMGINIRSGDNNNKKDVFKIRCIYFINFVLLNTDVLGAIFWFRSGLEQGKTFTEVTYNAPCLTFSFLANFKMLSLIFYEKTVHELIAALQKLEIKHFLRQNCAEELKMLKDEKNFLHAVFKGSKIVNYASILTFGCSPLVLIASNYYKTGRMDYLLPLIVLYPFDVDNITVWPIIYVRQIWSVITAVIGVCATDYLFYTFCVYISTQFRLLGHSIERVVPNNGLSVRTRLNGNLRMKFVENLKWHQELIRAASLLEQIYTKSTLYNFVTSSVIICLTGFNVAVVEDFAVILSFLFFLFMSLLQIILLCFFGDKLMKSSTNISDAVYNSKWYLTEKNVGKVLLMVQIRSQRACRLTAYGFAEVNLRAFMKILSTAWSYFALLQSLYSSHE

>BmOR25

MFEKALRSANFYMRVIGIPTDIRDGNRTLMERLRNRWFYCINFLWLNTDVAGEITWFVKGLLNGSSTLIENTYLIPCLTLCILGNVKTFFTIKYANHIIDLVAILKDLEIKNNAARKNETEIVKERLKFLTTSNKFLLFVIGTGIIAFGIGPLMLTASIYFSSGDMKLKLPFLIWYPFDSSDIRYWPFVYVHQVWSACIACCAVYGPDCFYFTSCTFIHIHFIHLQNDITNVIVESSRARKNGLYRGCHQAFLELTNRHKDLIRCVNLLEIIYSKSTLVNVVSSSLLICVTGFNVMVTFCWFAAPFASFLALGLVQTYLLCYYGDTIMCSSTEVSDAVYNSTWYGTNISQMRDYLFVMKRAQKPCKLTAYGFSDVNLRTFSRILSTAWSYFALLITIYRGNGQQ

>BmOR26

MSTGSAAGDSVAPHLRRLRQVGFCQLDPTSQSRRPILALMHRVYHRLVLAATVLYIFEQLTYAYQARNDMERLSRVLFLMLCHLTCIAKQFVFHSDADKINQLVVGLDDALCNQPVETHRLLLLETSRRAARLLMLYSGCAVSTCILWAVFPLLDQLRGRTVEFAFWIPIDYRHNAFQFAVVLAYAFYSTSLVAVANTTMDAFIATVLYQCTTQLRILRMNFESLPERAYALSRKTRQDYHTVTHELLVDCLLHYKKITETCNLLEQIFGKAILVQFGVGGWILCMAAYQIVDLSVLSIEFASMILFISCILTELFLYCYYGNEVSTESERLVTSIYSMEWVGARLGFQRGLLVLLERARRPVRPAAGLVIPLSLQTFLKIIKSSYTFYAVLRQTK

>BmOR27

MPSSFFLPNLENPDYPSLGPTLKGLKYWGMWQSGGIKRILYNSIHAFATFFVITQYVELWIIRNNVELALRNLSVTMLSTVCVVKAGTFVCWQKYWSGIIGFVSNLEKEQLSKNDAATQAAIVKYIKYSRRVTYFYWSLVTATVFTVILAPLVGFLSSPERELIANGTLPYPEIMSSWVPFDRSRGFGYWVTALVHTLICFYGGGVVANYDSNAVVLMSFFAGQMKLLSINCSRLFDDGNEVISNNEAMKRIKECHYHHVFSTIFNSLMSPVLFLYVIICSLMLCASAVQLTTDGTSNMQRIWISEYLMALIAQLFLYCWHSNQVLYMALEDRLGGLFEACLESGRFPSKWKTGRLVLLRKDGRPADSPAGYRPIVLLDEAGKMLERIVAARIVRHLTETAPDLSAE

>BmOR28

MHTLALVFALLYPSNCNIIKRAIGITLIIALSGGQLFWCMTYTFNVCVLILNYSGFDGSFCIASIRLCMKLKLVVYKVQKAFAESKSVSELKHQLNDAIKDNLDALKFHEQIQNVFFIALVGRRAYGPPDGEWLPSPMDFSNTRGRTKPLSTVYEPWLFLIFLLTFLII

>BmOR29

MFDFLQNLEDSERPLLGPNFWLINKTGLLLPKTNFGKLAYILVHEIVTFFVVTQYVELYVIRSDLDLVLTNLKISMLSIVCIVKVNTFVFWQTSWREVLEYVNEADKFERNQTDETRGKMIETYTKYCRRLTYFYWSLVFTTFLTTTNTPLMRYWSSPIFRENLRNGTEDFPHIFSSWMPFDKNHSPGSYCTIVWHVLLCAYGAAIMAAYDTCIVVIMVFFGEKLNLLRERCKKMLANDLYNHAFVIGQLHDIHVQLIKQSRLFNSLLSPVMFLYILMCSLMLCASAYQLTSATSTAQKLLMAEYLIFGIAQLFVFCWHGNDVLFKNANVSLGPYESNWWSSSPRVRADVLLLCGQLRVRHVFTAGPFADLTLSTFIKILKGAYSYYTLLRK

>BmOR30

MSVSNLKFEVLFKPTTMSLHMNRSHPSIKRNKIWLLQFISLMTLTVFCATGLITSLLFHDLKFGKYMEASKNGTIAMLSFTTTFKYSLLLYLQKSLNRLIAKIDMDYEIAKGLTPQEKAIVLNYAKKGVIVSKFWLFTAFAITFCFPLKAFIIMGYRFFIKNEFRLEPMFDMTYPEPIESYKTSFPVYFILFVVFFLFGCYASSLFVAFDPLVPIFVLHACGQLDLLSLRITKLFSDTKNPRIIAKELKVIISKLQELYGFVNFIKVNFSILYEYNMKITTISMPLSAFQVVESLRRGEFNIEFTYFFFGCILHFFMPCYYSNLLMERSENFRFAIYSCGWENHNDKNIRQMLLFMLTRATEPLGIATVFTNISLDTFAEMCRQSYTIFNLMNAAWA

>BmOR31

MKYLGVWVPPENENFARKFYKVFMMTLQHLFLFFQIIYIVEVWGDLEAVSQASYLLFTQACLCFKITVFQINMNKLKELLKQMNGYVFQPKNINQENIYIFILSIIKVQATRIKRLLFAFMISSQLTCGMWALKPLFDDVGSRKFPFDM

>BmOR32

FRKSLRGSQEEPRANGKSENVRFLINSHILHCGLRFNETNCHTHYIAKVAIFCFIVTYMLQVMELYWSKGDQEKLFECFSILSFCGMGVMKLVILRVYHQRWRFLLNQVSILENRHLDPGPLSYDSDNDNDDNEIVTFITKYTDKFKRTSSILIKMYASTLVIYVLSPFVEYIFRQFRGDLNIAYPHILPAWTPLDEFSVTGYLIMVSFETVACIYCVFVHVAFDLTCVGLMIFACGQFYLLRYRSERIGGKGRICRLLKSTEVRAHYRIVFCHGIHVLLV

>BmOR33

MIYYRKCKMELNFDKIFKIAIISQKFSGTYPYTKRDKKWATHFILMHGELTIICMLFIYNIIEFDLKAADYSQMCRNMCLSFVYMVITLLYINMLYYQSKLKMLIETMKAEYELAKTMSEEEQNVILEYAKKGRWLCRAWAILTTCGMAQFFLKSIVCTIYSAIQGNFRIVQYYEVICPEVIERHRNNPVIFITLYFCTFFYSLYTSALYTSVLPLGPIFLLHGCAKLEIVRLNIKNLFDNDDYVVQERLKKTVLQMQDIYCYSHEINECFQILYEFLLKATSLVLPITIFAVIQALGRGQFIPEFFAFIFGAFMVGTTPCYYSNMLMEKSEDVRMTLYSCGWETRFDLNTRKCIILMLCRALRPVSIRTIFRSVSLTTLTDVFQQAYALFNLLNAVWN

>BmOR34

MIYYRKSKMELNFDKIFRIAIISQKFSGTYPYTKRDKKWATHFILMHGELTIICMLFIYNIIEFDLKAADYSQMCRNMCLSFVYLVITLLYINMLYYQSKLKMLIETMKAEYEIAKTMSEEEQNVILEYAKKGRWLCRAWAILTTCGMAQFFLKSIIVCTIYSAIQGNFRIVQYYEVIYPEVIERHRNNPVIFITMYFCTFFYSLYTSALYTSVLPLGPIFLLHGCAKLEIVRLNIKNLFDNDDYVVQERLKKTVLQMQEIYCYSNEINECFQVIYEFLLKSSSLVLPITIFAVIQALGRGQFIPEFFAFIFGAFVVGTTPCYYSNMLMEKSEDVCMTLYSCGWETRFDLNTRKCIILMLCRALRPVSIRTIFRSVSLTTLTGVFQQAYALFNLLNAVWN

>BmOR35

MKLWQSIREFGLEYCDLPTTLQNVASLLRAITLNIDSRHTARIPFICYVMTVVITLSYFYVFLVSMAWFVFVRSAETRDYLAAMVVLSLGISSEIGTLKFFYTFIYIKKVQRIVREYLECDHMVVPESRFADNVLKTMRNVKKRAILYWVVVIGNGVVYVTKPLFMSGRHHMEDRYIVYGLEPMFESPNYEVAYFLMMFGLCFICYPPANVTVFLIVVVGYTEAQMIALGEEMLRIWEDAVAHYNNKYHTVGALTNSSEKNKIINQYVKFRLTEIIKMHTTNIQLLRQVEFVFRSAIAMGYVFLVLGLIAELLGGLENTYLQIPFALIQVLVDCYTGQKVMDASSLFEQAVYDCKWENFDKSNMKTVLLILQNSQKSMRLSVGGITVLGFSCMMSVMKSIYSAYATLRTTMS

>BmOR36

MVFNSKKNIISLFSLLEDSRHPSVGPHLRLLSLTGIWYPNSKTNITLLKRACFYVIVLFFVSQYLKCIIKFKIDSLQLILEYAPFHMGIVKTCFFQKDYNVWQDLVSFISKTERDQIAKKDPKSIKTIQSYISRNRKITYSFWALAFIANIGVFSKPYQNNQSDVNGTVTYNHLFDGYTPFSEEPPGYYFSMGIETILGHVVSFYVLGWDTLVVSIMIFFAGQMQMSRLQCSRMINGSPERTHKNIIKCHKFHTDLIKYQKQFNSLISPVMFVYLFVSSINLSVCIVQIAEIEDDFATVLSSFIFLLACLIQLLLFYWHSNEVTVQSELVSYSTFESNWTSTQNKLQKEVALLGLTTSKTLVFTAGSFNHMTLATFISVSLTFELFELID

>BmOR37

MELGCSRHLKLPCSLHPIGISKHGNTLSELLIYFPAIPKITYAILAVLLTVYYYIYLCSITWFVFVRCPQTGDLAAASIVFSLGVSSEIGAIKLFIIAKLRDITGEYLQCEADMAPGRLRARVGRSLRTVRRRAFVYWLVLVVNAFAYDLMPAFLPGRHLSEDVFVIYGFEPMFESPNFEIASTLMGVSVVFICYTAGSISAFLIVIVGYSEATMLALSDEISCVWDDACASECQQPNDFIRARLGKIVAIHTKQIRLIREVEVVFRGALAGGFACVAFGLIAALLGGLENTFLQLPFCVIQISVDCFVGQRLRDANVAFETAVYNCKWEYFDKSNMKTVLLILQNSQKTMGLTAGGVAALDFTSLMTIFKSVYSGVHHSQTDD

>BmOR38

MNLSQSVNEQANEYVKMRLERISKIHSPMLPFEDIQDFRELCCIPLAVYAVTGSITASYVYAFLISLLWFLFARCTDPEDFQVAMVVFSLGISSEIGSTKFFNSIIYIKELRKLFKDYLLYDATCPAQGRLRLHLLTTLRYVKRRAIIYWLVIIGNGFIFAIKPLLVEGRHLAQDDLVLIGLEPMRQSPNYEIAYAIMTMGVCFICYPPAHVTMFLIIIVGYTEAQMLALSEELKHLWNDAIEHYEKHSRTEREADAAMKSKILNSFVNFRLVQIIKSHSTNVNLIGRVENVFRGSLAVGYVFLIVGLIAELLGGLENTYLQVPFALIQVAIDCFIGQRVNDANIDFEKAVYDCKWENFDKRNMKIVLLLLQNAQKTVSLSAGGIAKLNFSCFMSVIKSIYSAYTTLRTTMK

>BmOR39

MLWSVFSYFTRADDVLAGIVIFSLGVSSEIGLVKLCFMYANIDKIQKITEGYLKSDAASARNSRFSKNILHTMQSVKKRGVIFWLVIISNGVVYLVKPIVTPGRHFMEDQFIILGLEPKYETPNYEIGFFMMAVGVCVTCYLPANITAYLITVAGYSEAQFLALGHELANLWPDAQLHCRAMNLSQSVNEQANEYVKMRLRELVKIHSTNVNLLRDIEGAFRGAIAVEFLLLIVGLIAELLGGLENTYMQVPFALIQVSVDCLTGQRVMDANLALERAVYDCRWEEFDASNRRVVLLLLQNAQKVATLSAGGIATLNFSCLMAVIKSIYSAYTTLRTTMK

>BmOR40

MTGAGAGTFRTGAGPGRGDGVARRGESGETTTLGRDAFAALGCFGAADGSTARARFFPRVTVLNPSEVPGSGLAADSNSISDSESEPELDAAQDAIDAGAGVGGDIGESRARTVFGIQGHDASDSALRMHNNVAIYAKTTMSGNSQLTFATAATIFLKNASGPNGVAIGTDYAICVVSLSLFFCYRFTELVEDTYNSYLLFQLVGSVGIICMSALRILVVDWRSVQFFSILCYLSVMISQLFVCCWCGHELSATSEELHTILYNCAWYDKDVKFKRDLIFMMARARRPILLRAGYYIGLSRQSFVSVSIPRIRFNAILVI

>BmOR41

MMGNSTDLFLDRTKSILNFFAMWRSFEKPIPLKVYMAFIMTTQYLFLIFEIIYIVNVWGDMAEVSEASILLFTQASVCYKITSFISKTNNFVILLGLIESEIFSAQTELHEKILILKARKIKRLCMFFLVNAVTTCSLWAVIPLLDISSKMLPFKIWMPASTGESPHYELGYLYQMITIYISAFLFIGVDSVPLSMIMFGCAQLEIIMDKIGKVKSRPLDQQPMQRQAVLNSNYELLVECVRRYQSVVRFIELTEKTYHANIFFQLSGSVLIICNIGFRIAIVDSNSLQFYSMLTYLVTMLSQLFQYCWCGHELTIRGEELRETLYQSPWHEQDIRFRKVLIITMERMKRPIIFKAGHYIPLSRPTFVAILRCSYSYFAVLNRVRNE

>BmOR42

MDIPKFEELLKQIQMNFWLMGIPFDNPKIQIRYYVLLLTLSLMLIDEIAFFGSRMSSENFLELTQLAPCICIGVLSVLKILALTAKRQKIYELTQNLECLHKIILNDTRKTELVRKNLVLIKFITKYFFVLNAVLIFVYNFSSPVIIAYNYIVSNEVQFVLPYAVLLPFKTDSWIPWLIVYVYSIFCGFTCVLYYATVDVLYCVMTSLVCNNFSLISFKLQKVNRNTAHLLKEVVKEQQYVLKLAEDLENIFTAPNLFNVLIGSVEICALGFNLMIGDLTQIPGCILFLSSVLLQILIMSVFGENLISESSRIAEAAFLCKWYEMDQKSKKTILTIMIRSHKPKKLTAYKFSIISYGSFSKIISTSWSYFTILRTMYTPPGTKFQDDL

>BmOR44

MYTYFKVLVFWLNKDKVISLQKILHCKEFKPKEPEHKEIIRKSIRKARFVMTSYATMCVGAVSVGIILPLTENFDILPTNVEYPFFDVYKNPTYAYLYLHHIYYKPATCIIDGVMDTILAAFVASAIGQIEILAFNLRNFDVLAERRRKRAISGNKYIGKYTNLYFTKRILKECILLHNSIIRYVSVIESAFSLASALQFMLSVMVLCLIGIQFLSIENPTSHPMQMVWMAIYLTCMLIEVFILCWFGNELIWKSNDLRQAAFDGPWRNLNRKTCMFIIIFMERCKRPMRLSAGKIFTLSLDTYTVLINWAYKAFAVMRNMKK

>BmOR45

MKVLDNVNHAVKVTMNCCRLYGLFVSDDLTKRQLIIMRAFSLMLYLFFVGFFITTQSALIITMWGDLNLMTNVGLVLGTHLTLSAKVFTLHYKEKEITNVIYKNEVRLRAETREQGKYIISEMNRETTLFMRLFIPFGMGTVTAWLLCTPKGELYTPAWYPCNTTKSPAHEIILAHQGIAVILTATLEIAIVLLMTSIVAVCRCRLKLVGLSFETICDDLPSNIMNKLTADEQVIVAKRVRENVIEHQAVLECINDIQDCFSSAMLVHIAISTMIICATAYQLAVEKSLDLTQRMTMASFLGGMSTEIFLFCYQGGHLSIDSMEVATAVYSCPWYTFPTSLKRSLLVIMIRAQQPALLTAGGFAPLLLDTFVSIMKASYSFFTVLQNASE

>BmOR46

AATYVQIADLIDIWGDLDLMAETSLLLFMELAVISKILTLIFKYDKIMEIINGTEDILCSENRLEGQKIIASIDKETTRFFQYYTSSVIFTTFFWFLGEHSSTFFIRAKYPFNELKSPGYEFALIHQCMMMVFTGYFEFNINIFFASVVAGCRCRLKLVALSLRNICINIPVNKKNLITPEEEKLITERLHCAISQHKYALDAAEDVKHCLSKVLLVQLTVSIVIICTTAYQMAVVRILYWWQEKINYYASLTMAGYLFGTSLEVFLFCYQGEFLRESSEEIADAAYECPWYTLTRPLKKTLLIIMTRAQRPATLTAGGFVTLDITEYMAIMKASYSFFTVLQQVSE

>BmOR47

MKLVFDNFIFALKVTLNWCRYFGIFIPDELTGRRQKLLVQAYSVFMFMLFIGFFIITQIILFILVWGDLSLMTDVGLVLGTNLALSAKIAVFFFKREELASILKKNDDTLRFETREEGKKIISEIDRETNAFMKVFFCFGVGTVIAWFLSTPKGELHIATWYPCDTKRSPAYEIIMIHQLAITADLLMLSMIAVCRCRVKLVGLYLQTICDDLPCNVKNKLTSDEEVIVAKRIREYVIEHQAILDCISELQNHFSPALLVQLLTSVVIICVTAYQLAVEKSSDLLRKFTMASFLFAMSTEMFTFGYQGGHLSHDSMEVATAAYSCPWYTFPTSLKRSLLVIMIRAQQPALLTAGGFTTLSLETFVTIMKASYSFFTVLQEATD

>BmOR48

AAACVQIADIIDIWGDINLMAETALLLFMEFAVISKILTLLLRYDRIMEIINGTEEILYFENGLEGQRIIASVDKETTRFLQFNSAFVVLSTTFWFTGEHSSTFFIRAKYPFNELKSPGYEFALIHQCMMMVFTGYTVFNINIFFASVVAGCRCRLKLVALSIRNICINIPVNKKNLITPEEEKIVKERLHCAISQHKCALNAAKDIKNCISEFLLVQFTVSIIIICTTAYQLAVVCLFQNKAIGNIQKTSMFGYILGASLEVFLFCFQGEFLRNAVRDCEEIADAAYECPWYTLTQPLKRTLLIIMMRAQSPVILTAGGFIDLSIREFMGILKASYSFFTVLQQVSE

>BmOR49

MLTCFATIFSAVNQTGYIVLFINLLAHELGHFYVITDVLNGIFEKNDADRDPVFIDRKLKFCAKHYQYLLKFHNEIKNLYKIIFGAHFLMMTIVLVTTLQTMNSWDIRNTVLTAVTGIMPLFIYCFGGELLITAGMDMSTAIYQCGWEKMGVKQAKVVSVILCLSQRPLCLTAANVFVMNRETFGGIAQVVYKIYAVFN

>BmorOR50

MPSLLKTESLALTLTLNTLSWAGLILRDDYTKTQRIIMKVYGGLVFLYLFVFTAYVQIADLVVIWGNIDFMTETSLILFMQLAVSAKVLTLMLKSKKIMEVTNEADAILNSEKKVEGQRIIASIDKNTTLFLKYYGFFVAFTIICWFMGENTSTFFIRSKYPFNELKSPGREFAFVHQCIVVIFTGSFDFNVDIIIISLVAVCRCRLKLVALSLRNLCLDIPMNKRNLITSDEEKVITERLRNIISQHKRALDAAEAIKHYLSGALLVQLMVSIVVICTTAYQLAVKKSTTMQSLTMAGYLFGTSLEVFLFCYQGEFLRESSEEIADAAYECPWYTLTRPLKKTLLIIMTRAQRPATLTAGGFVTLDITEYMAIMKASYSFFTVLQQVSE

>BmOR51

MDCTIVAFYSQAKTQIKMLRYDLEQLGKIDNIETKFTENIFERSSHIWKALKDEKIKIHSKLVFCVEHYRQIVWFVKEVESIFGEAMTVQFFVMAWVICMTVYKIVGLSIYSAEFVSMGVYLGCMLAQLFIYCYYGTQLKVESESVNTSLYCSNWLSSLPKVRRQMLIMMQYCSKPLTPRTAYVIPMSLETYISVLKSSYSLFTLLNQKH

>BmOR53

MALKKMLALTKGLEDPTHPLLGPTLKALSVFGLWQTGSQKSTVIYNTFHFLTFLFVITEYIDLYTVRKELSKMLNNLSVTVLSTICMIKTLSYVCRQSHLKVLVREISELELELMKTTDKNIVKRLRQYTVYTRAVTYVYWFLVVGINVVLLTSPLLKYASSEIYRSEIKNGTEPPPLILCSWFPFDSARMPGYFWATMVHIIMSIQGCGVVATYDMNAVAVMSYLKGQTSILKDKCKAIFDETASSRDVLNRIRDCHRHHNILLRHYYMFNSLLSPIMFVYMLICSFTICCSIIQLDSSETTISQRIWIIQYSIGQISQLFLYCWHSNEFAAKVKKKHFPLFPINLF

>BmOR54

MGLNTIKEFFVNVKRRFQDVSIDSLLWIVNIVPSLAGFSIRSDRVSAPFWIVHWSLLVYVYAVGNAVYQWKFANEAIDYITSFINVSLLILIGNNSWWFLANRRLLKSVLHKIEVNDELSRRSEQSRLKHKKLLKIIKRIVLVFYMSNYVNASFIYLPNRVDVLNNYAMTPCVGMEPLTVSPNRELCLTILCMQEFSIMTVVLNFQALLLCFIAHTAVMFQILADEIMALNNYENLEEHQAYVKEMLPIFVKRHSLTLSAVDNYKSLYSVPLGVNFGSNALTILLILYLPVLEWFKFIPIFVFCFMLFFLYCFLCQKLVNASEAFETAIYCCGWENFALREMKMIYVMLHQAQKPVELLAADIVPVNMNTFATTLQAMYKFVTVVKF

>BmOR55

MCFLKIKQQIIDIQKHFKDYSLNGSLWIVNLLPRLMGFNLRADKVGVFFWTIYILLLVYVFGIGIFVYLWKHVDTMSGLMKSYLNLSLILVIVNNSCWFLSKRSLLNKVLKKIHLIEDLSCESEHALAKYRRVFKIVTHLLLASYVLFYFTEIYFMFLFRNYDLLEDYSLAPCVGLEPLSSSPNSEICLIIVLIHEFISTTVMMSFAALFLVLIAHTAVMFLVLAEDMTKLTDLINLADHRKMIRESLRSLIHRHSLLLQIVYELRLLYSVPLGINFISNAMSILVLLCLPIHEWPSFLHIIGYCFFAFFLYCFLGQNVINASEKFIDAIYCCGWEHFGVAEKKLVHVMLRQAQKPVEIIALGMISVNMNTYVEALQLIYKFVTVLKI

>BmOR56

MKLLEKLEDPDRPLLGPNVKALKFWGLLLPESRSKKYFYLFMHFAVTVFTATEYIDVWFVKSDLALLLNNLKITMLATVSVLKVTTFLLWQNAWRDLIGYVSRADLEQRATSDSRKLALINGFTGYCRKITYYYWFLMYTTVAIVTVQPIFKFFSSAAYRLDVQSGNGTYLQVVSSWIPWDKNTLPGYLLASIYQTYAAIYGGGWITSFDTNAIVIMVFFRAELELLRIDCAALFDDEKSFGDMAFMRRLKECHRRHTELVKHSRLFDSCLSPIMLLYMFVCSVMLCVTAYQITIETNPMERFLMTEYLVFGVAQLFMYCWHSNDVLYASQDLSRGPYESAWWSRDVKYRKNLYILVAQFNKVIVFSAGPFTKLTVATFIRILKGAYSYYTLLSQSQMNKT

>BmOR57

MPSLIKNRIFGLTLTLNTLSWAGLILRDDYTKTQRIIMKVYGGLVFLYLFVFTAYVQIADLVVIWGNIDFMTETSLILFMQLAVSAKVLTLMLKSKKIMEVTNEADAILISEKKVEGQRIIASIDKNTTLFLKYYGFFVAFTIICWFMGENTSTFFIRSKYPFNELKSPGREFAFVHQCIVVIFTGSFDFNVDIIIISLVAVCRCRLKLVALSLRNLCLDIPMNKRNLITSDEEKVITERLRNIISQHKRALDAAEAIKHYLSGALLVQLMVSIVVICTTAYQLAVKKSTTMQSLTMAGYLFGTSLEVFLFCYQGEFLRESSEEIADAAYECPWYTLTRPLKKTLLIIMTRAQRPATLTAGGFVTLDITEYMAVSLISNT

>BmOR58

MKLVFDNFIFALKVTLNWCRYFGIFIPDELTGRRQKLLVQAYSVFMFMLFIGFFIITQIILFILVWGDLSLMTDVGLVLGTNLALSAKIAVFFFKREELASILKKNDDTLRFETREEGKKIISEYPCDTKRSPAYEIIMIHQTIAVAVIASLAITADLLMLSMIAVCRCRVKLVGLYLQTICDDLPCNVKNKLTSDEEVIVAKRIREYVIEHQAVLDCISELQNHFSPALLVQLLTSVVIICVTAYQLAVEKSSDMLRKFTMASFLFGMSTEMFMFGYQGGHLSHDSMEVATAAYSCPWYTFPTSLKRSLLVIMIRAQQPALLTAGGFTTLSLETFVTVS

>BmOR59

MDTNPSAAGDSVAPHLRRLRQVGFCQLDPTSQSRRPILALMHRVYHRLVLAATVLYIFEQLTYAYQARNDMERLSRVLFLMLCHLTCIAKQFVFHSDADKINQLVVGLDDALCNQPVETHRLLLLETSRRAARLLMLYSGCAVSTCILWAVFPLLDQLRGRTVEFAFWIPIDYRHNAFQFAVVLAYAFYSTSLVAVANTTMDAFIATVLYQCTTQLRILRMNFESLPERAYALSRKTRQDYHTVTHELLVDCLLHYKKITETCNLLEQIFGKAILVQFGVGGWILCMAAYQIVDMEILSIEFASTALFMGCILTELFLYCYYGNEVTVQSGLVSESVYAMSWLSLCPRERRALVVVLERARRPLRPAAGRVVPLTLNTYLKILKSSYSFYAVLRQTK

>BmorOR60

MVRPCRYFAIHFILLRFLGLGWWHHPHENETRNYPGLYLYYSILTQLVWVVGLVGLETIDPFVGEKDMDRFMFSLSFVITHDLTLIKLYIFYFRNVEIQDIVRTIEIDLYRYYQNDDKIRATIRISRIFTAAFLFFGWVTIGNANIYGIVQDLRWKDIVKNLNETTSKPLRTLPQPIFIPWPYQEDKHYILTFILETMGLLWTGHIVMTIDTFIASVILHMSTQFAILREAIVTAYDRTMIALSEGALQSGVLCENSNGNEENNQIFLESFYSKEHIESVLESTLLSCIRQHQLLIGCVEKFSKTYSYGFMTQLLSSMAGICVVMVQVSQGASSFKSVRLVTSLAFFFAMVIQLAIQCFTGNELTIQAERIADAVMESKWEKMPVRLRRLLLVTMMRAQRPLHLTAAGFAYIDNTCFLSILKAAYSYYAVLSQKQG

>BmorOR61

MARITDVFRLNFIFWKFLGIWGKSAPSKYNMAYTALYLSASLFVYDIFLTLNLIHTPRKLETLLRETMFYFNHLVAMTKILKMFIRRKKILVIFDLLDCEEFKPSDEDSQEIMKRKNEFYYIYWRIVAVTSNLSCFMQVVGPLIKMLIWKSELGLPVCKYYFMSDEFRNKYFVIWYIYQSFGIYNQMVNNLNLDTFNCGMLWMAVGQLQILKTKFVNFKLNDIENSLDLKTRDDMQTERLRKYLTHYEIILKYCATVQDILNITIFVQLGMSSIVICVGLCGFVAMPSNTETAIFMSSYLITMTMQIFVPSWMGTQISFECGELMSAAYCCEWIPRSKLFKRSLILFVERAKTPVRITGLKIFTLSLDTFTSIMKTTYSFFTLIRQLQVDEVN

>BmorOR62

QNVILEYAKKGRWLCRAWAILTTCGMAQFFLKSIIVCTIYSAIQGNFRIVQYYEVIYPEVIERHRNNPVIFITMYFCTFFYSLYTSALYTSVLPLGPIFLLHGCAKLEIVRLNIKNLFDNDDYVVQERLKKTVLQMQEIYCYSNEINECFQVIYEFLLKSSSLVLPITIFAVIQVSSLHICFIFLIPNTQWNVCSQNKHFLGSLEMSCRNCHSK

>BmorOR63

MKLWIRNANFTISLSLTLLRCLGFWSPDGLAGNKRLLYNCYSFVFFMFLLGIYILIQVVDMIKIWGDLPLMTGTAFLLFTNFAHATKVINIVIRKNRIQRVIQQANAVLMGVQSEEARRIVKSCDFETSIQLCLYFLLTFVTTVGWATSAEKHQLPLRAWYPYDTSKSPAYELTYIHQVAALLIAAYINVAKDSLVSSLIAQCRCRLRLVGLALASLGQDLKIDYQSQLSPAQENILNLRLKTCVLEHQTVLAAVTELQACFSKPTFAQFTVSLIIICVTAFQLVSQTGNLVRLLSMGTYLMNMIFQVFIYCYQGNKLSVESSEIAGSVYFSPWYLGSVKLRRALLIVMVRSRRVAKLTAGGFTTLSLASFMAIIKASYSLFTLLQQVKQKK

>BmorOR64

MGVSNGRGTVKPFLYPLVDELDYNLIVGVHLPFEYKTPSRYPLAYITVVIAFIYVSYFVMVTDLIMQAHLLHLLCQFNVLADCFENMLNDCVKGFEGPLVSLHEYIHPLIDEFEYNLMVGLRLPFSFDTPLRYLFTYVIVLIAFNYTAHYVMVTDLIMQSYLIPLICQYAVLADCFENILIDCSNDYGDHARRNDIVYSRSMELRAILSRPMLGQLASSGLLICFVGYQATTSISVNIVKCLMSLFYLGYNMFTLFVVCRWCEEITNKSLNIGNAVYCSGWESGMTVVPTVRSTILLVILRANKPIVFTAGGMYNLSLTSYTSLVKGSYSALTFLLRIQHE

>BmorOR65

MRLGFEVSISEYLYRNIFYIYTLFHILLHFYYILHMIKLDLEAIFDDIDESVALLPHRDTRRIEVQKILNGRMKRVVTWHISVFKAVEAVSSIYGPPLAYQVMFTSIAICLIAIQITQKLENGILDIRFTMLGVAACLQMWIPCYLGTLLRNKAFGVGEACWNSGWHQTPLGRMIRQDIIIVLLRAQQPVTIKFPGLQSIQLETFSSVIFNLYGYYYFLLLRWVDELTAHLVLSGYWSP

>BmorOR66

MRFGLKVYIYTLFHILLHFYYILHMIKFDLEAIFDDIDESVALLPHRDTRRIEVQKILNGRMKRIVTWHISVFKAVEAVSSIYGPPLAYQVMFTSIAICLIAIQITQKLENGILDIRFTMLGVAACLQMWIPCYLGTLLRNKAFGVGEACWNSGWHQTPLGRMIRQDIIIVLLRAQQPVTIKFPGLQSIQLETFSSVIFNLYGYYYYYCLDG

>BmorOR67

MRFGLKGGAAVVTILETLELISQGGFIETIQVTFGGQLSSMLFISACIICSTAVQILAIESPLDNLTTVGWILVYLSLCILILFVDCYFGNTITVKCAYLPTAVFSIPWLDQPKNIQVSTLLFMAKTQQPVQLIAAKLVPVSLTTFTQVSYCPPLDLKCLQGGVIAHLAKD

>MsexOR1

MIFMDDPLSKSIKDPRDYRYMKLFRSTLRLIGSWPGRDLKEEGATKYEIAPLYWVLVIKITCFLLTIIYLIENTNKLGFFEIGHVYITVFMTMITLSRSITLSLNPKYRRVMTKYITKMHLFYYKDMSDIALKTHIRVHKLSHFFTMYLSTQVVLGTVTFNIVPMYNNYKVGRFENNILVNDSYELSIYFKTPTKFLSTLNGYIAITTFNWYSSYICSNFFCMFDLALSLLIFTVSGHFKILIHNLNNFPLPAVVSDSSKVLKTDEIQAPLYNKTEKKDITLRLKQCIDYHREVLEFTQDISEAFGPMLFVYYLFHQVSGCLLLLECSQMDAAALMRYGLLTAVLFQQLIQLSVVVESVGTVTGYLKDAVYNVPWEYMDTQDRKTVCIFLMNVQEPVHINALGLAKVGVQAMAGILKTSFSYFAFLRTVSN*

>MsexOR4

MKFFVDGSEIAHITKPQDIQYMQMLKFFTNSLAGWPIEAVEGIDGKKNFYWRNGLVVIAYAYFFGQVFYIYRYINDYTFLVMGHSYITVLMTIVTIARHTLPYFKCYDDTTADFVHNIHLFNYRNKPGYYKEFHLKIHKISHAFSVYLCTLLVTGPSMFNGIPLYNNYASGAFSFNRSPNVTYEQAVSLLLPFDDTNNFKGYFVVFLANCCVSYISSCCLCIYDLLLSLMVFHLWGHLKILTKTLDNFPKPGFLNPQAIEADPNKSLKFSDEELKVIHKKLGECVAHHQLISNFSTRMSNTFGLSLFIYYGFHQLSGCLLLLECAQLEAAAIICYGPLTLVVFQQLIQLSFIFELIGTVNEGLTDSVYCLPWEAMDQGNKKIVFTFLRQSQKSMNLKALNMLSIGVQTMAKILKTTMTYFLMLQTIAKDES*

>MsexOR5

MKVPLKKFRPTETTKLLDELNKLFYIFAFRGFWVEEVKLPKTFVKIYDAMYPALNVSLVIFCALQIGAHFTQKHLNIQQKININIMGIAQPLTKLCCLNCMYYKEECKQVLYHLFVAVKEIYNDEETEKMLVKKLKFSLWVYMFSSVSTTLLYGMYALIEMVRSGSTFVGTVTAWPDTTDTSALAACARVYLYFFWIVYTSTGSMVILMVLTFFMGLTYQYKNLQKYFENLNSIFEDKQVTHEELEMNFEKALQKGIKAHSDTLWCVNQCQTICKRINMAIVLINTGILIVLMQGFLDSKDDILKVGTCLIVLGMCLMILAFFMWNAGDVTVEAQKLSDAIYSSGWYNCYGKRSARIRSLVVIAMIQAQEPVVFTAFGVIELSYETYVAIIKSAYSVFSVLY*

>MsexOR6

MEETKKYAPTDTTKLLDKFNKILYVFGFRGFWMGDVKLPKTVMKIIDILYPVLIITQGTFGTLQLGAYFTQKHLNSVQKIDALVMGFGQPMMLLYCLNCRYYKKACREVFYHLFVVLKTVYNDKETEENMVKRLKLYFGAYLIFSLLVVLLYGSYASVETVRKGATFVTVVTAWPDVTDTSKLASYARVGIFMYWSVYSFTTSVVIIIVLIIFLGLTYQYINLQRYFENLNSIFENDRLSHEEMEKNFEKALQNGIKAHSETLWCVKQCRMICSAINAGVILLTTGTLVILMPAILGSKDDLLEAMMYLMVSNIVLMIMAFFMCNAGDTTVEAQKLPNAIYSCGWYNCYGKRSARIRSLVVVAMIQAQEPVVFTGFGVIELSYENFVTIIKSAYSLISVFH*

>MsexOR7

MDEPEFKPFHKTYQIITFALSVGMIYPNPATDKMRLASIPISILTILPLACMIFLEMYQCWTQGDIVNIIRHTTVLGPFLGGLFKMFLMYHKRKQAKQILDEFERDYHLYNSFTGDYLNIARDGIRNSLIYSERGWAITVTTCVMTFPVMAILLNMYNYTFKFQATKYMIHDLNKPGASPEARFDSPYYEIMFFYMMYCSLLYVINFIGYDGFFGLSINHACMKTNLYCKMLEDAWKAEPNERYRRVVAVIDEQCRMFEFVNLIQDTFNIWLGIIFLATMIQICTCLYHITEGYGFDLRYMIFVSGATIHIYLPCRYAAKLKAMAAETATHFYSSGWERVNDRRLRNMLLFMVARAQTPLQITAFNMITFDMELFVSIMQSSYSMFTLLRS*

>MsexOR8

MAQTLFDKSLSKLSMVFRWSGTNIAIGEAAPTNTKRNRCIYSFNFILQNTNVLGGIYWFISGLKTGKSYTELTFIAPCIIISILSVMKSMSIIIYEKKVYQLMENLRMMEAHERNRENTAERRKIIEKGVNFLNLVINVLCGLYLIMFVCFAFSPLVLMILKYMKMNEIEFKLPFFIAYPFDPYNIKVWPMVYLRQLGTEIVTVSNMCVADFIFCIFCSYITIQFRLLQYDIEHVITGTRESIYNDEMHGGKIRNKLVEIIKWHQELIMCVNLLENIYSISMLFNFISSSVIICLTGFNVTENHDIVLVITFITFLFMGLVEIFLLCFFGDMLIDASSDVSDAVL*

>MsexOR9

MTSPDSKIKEFFRKFTLITYLCGLADFWIEDLDLPTQFIKCYDTFCKIFNNFLYLYVIAQILSLFTQHNLTEKQRNYQLMFCVAHPFVVTFPTLIAKHRKKLQSVLFRLVVSLKLVYNDLDIEKEMIKRIKFYLFAVSAPFWISMISYCVDSYVQVVTSGTTFNIMILAWPLVDDHSVIASLARFIYHIIWILFETRVTAAYVMVISLTTCISYQYRNLRSYFESIHRVFEEDLTQKEKEKKYEKLLKIGIQAHSDTLRVTKDGTEACSAVLSAQVLFNTVFLVILMYQAVVVNENRSLVKMFSTLCTVITLLFSTWFFTCNAGDVTYEASLIGTAMYTSGWQNCRGLSSLSIRKLLVIALARAQEPIILKGFWILTLSHQSFLSIVKFSYSIFSLLY*

>MsexOR10

MALHFDDTIKKNDFVFRYAGINIKSGERKKNDAIKSRRMYVFNFFWLNIDVYGSIFWFIDGLRMGKDFIGLTYIAPCISLSTLSLIKSLFLISSEKHMFKLIDNLRELETRENARPRSVQKEEIINTEIKFLNYVLKTINILYVVLVMTFALSPLIIIAIKYMQTGEVELILPFLILYPFDPYNIKLWPFVYLHQFWSECVVTLNICCSYIRIQFRLLQHDFERIISAPSGNRRVRENDFKAKFIELVKWHQDAIESVSLLETIYSKSILFNFMSSSLIICLTGFNITVVNDFAFVVTFLSFLFMGLVQVFFLCFFADLLSESSVEVSNAVYNSQWYMADSNIGKQLLLVQTRAQKPCKLTAWGFADVNLNSFMRVLSTAWSYFALLQTVYGQ*

>MsexOR11

YSFRKVFLNETLGYPHILPCWAVLDELSFLGYLLTIVAEAVAAIYCVVVHITFDITAVGIMIFICGQFESLRRCSESIGGKGKVCNVTAERDARARFRIKKCHRIHVILIHSIKELKELIKNILGVYFFVATFKLCSLAVRLKTENMSKMQLVTLVQYLGASITQLFLLCYYGDAVFNESAITMGQGPFGAAIWCVSPKIRRDIVILGMGMMKPHSLQAGPFNVLNLPSFIQIVRTAYSCYAVIGPK*

>MsexOR12

MEQAKREIDESLKLSAFCMRRIGLSFEKHKNASAHLRQQLMFALSVCSICYHVFSEIMYIGLTLANSPRVEDVVPLFHTFGYGALSIAKVFALWYKKDVFSQLLRELVGIWPTPPLEDEAQAIKDKSLDALRITHKWYFAVNVLGVWFYNVTPIAVYFYRLWQDGDAQVGYVWVSWYPFDKHQTIAHVAVYIFEIFAGQTCVWIMVSTDLLLSGMASHISMLLRMLKRRLESLASTEKTDDEYYHEILENIKLHQRLITYCYDLEDAFSLSNLVNIVLSSLNICCVVFVIVLLEPFVAVSNKLFLGSALIQIGMLCWYADDIFHANADVAAAAYNSGWYSTNARCRRALLFLMQRAQKPIAFTAMKFTNISLVTYSAILTRSYSYFALLYTMYNEN*

>MsexOR13

MENNRNFRQAIYSCGWEKQPDKGVRQLILFMMTRASLTLGITTVFYEICLDTFAEMCRQSYAIFNLMNAAWS>MsexOR14

DRWIQWKVTAVSAFIGVCALMEHILSMMSAIGLHCAPSQYLRKYILNSHGFLLRINEYSLWFAIPIFILSKISTMLWNFQDLMIIVISMGLTSRYNRLNMYVGHIIKIERKLSDSPKTGSDLHVHNEIWRRIRESYVRQAELVGMVDKEFGALILLSNINNLFFICLQLFLGLNATARGALINKLYYFISLGWMLFRACTVVLAASNVHMHSKKALVFLYSCPKSGFNIEAKRLKHQLIHDHVGLSGMGLFSLKRQLLLQVAAVVMKYELVLIQYDK*

>MsexOR15

MLYIYTRWNNIAFSTHGTIFTIVPITFLILTKVLSAQKESYRRLMKTFLQEIHLCNFGSNSYMKQRAVEVEKYSRYLATFFFVFLVVYRALWTIVPIVFNLKNSKAIENKEIPLKTCFYMWLPFDYEHEYKYWVITHIANSTLIALGCIVVTSYDTINYSIVFHLIGHIKSLKHLIKTNISQNLSDDETKRGLVEVIRYHCFVLKIFGEIERAFGINVTGNYLYNLIADSLLLYHLMLGDKENKLMYGVMLLVFMGGLIVMTLILEEVRRQTFDIPQTVYDMPWEKMSVSNQKIVVIMLARTQPTLEYKSAGGLKAGVNPTIQIIKSTFSYYVMLKSSL*

>MsexOR16

MGIFVQNVNRSLRFCLTMLKLTGFLVPNGVIEFTVFHNIYWFFWMMFVVGINVITQTGDLIQVWGNLSLMTSAAFLLLSDVAMMMKIINVVMRGRVIQTVIDGMDLELRSEARAKGRKIIKECDDQTTRHLYLFLCLSGVTVLGWAGSAEHNKLPLRAWYPYDTSTSPAYELTYIQQNQLITTGQNTIDLRLRSCVLKHQAALRAASQIQECFSEPILTQFTASTVIICVTAYQLRIEIHQSNLVRVISMMAYLLCMMLQVFLYCLQGNQLAEESSNIAEAVYECPWYRLPLPLRRSLLLIMVRSRRVAQLTAGGIATLSLACFTSIIKVSYTFFTVLQSVED*

>MsexOR17

ELDDLLGDAMLLTYIFGSVFICLTAFTATVVGDIYMTVRYVSFFLSLLVEVFVQCIIGQILIDHSEKFERAIYSADWPHSELKTKKMLLILLTRAQKPFVYSANGYLVMNLDTFCGICSLSYQFFNLLRTAYN*

>MsexOR18

MLSFLKNLEDSERPLLGPNYWILKKMGLLLPKNKISKIFYILVHEIVTLFVVSQYMELYVIRSDLDLVLTNLKISMLSVVCVVKVNTFVFWQSDWKQVLEYVTKADMFERQNQDVPKSNIINSYTKYCRRLTHFYWGLVFTTFLTTTNTPFMRYLSSSNFRNNFANGTEMFPHIFSSWMPFNKNHSPGCWITILWHVLLCAYGAAIMAAYDTCVVVIMVFFGGKLYLLRERCSNMFKNSDENASEATVKQLHGIHIQLIKYSRLFNSLLSPVMFFYMVMCSLMLCASAYQLTSATNAAQKLLMAEYLVFGVAQLFLFCWHSNDVLIKNENVTLGPYESNWWSASLRQKKNVLILSGQLRISNKFSAGPFADLTLATFITIIKGAYSYYTLLRK*

>MsexOR19

DLLIACIKHHQAVKRLVLKIEDTFHSSVFCQLCTAVLIICIIGIRLSQDSPNNASFYGIVSYLTLILSQLYLNCWCGTEITDRSLDFRDWLYEVPWYDQDKRFTSTLSVLLQCTKKPLEFRAGHYVLLARATFVSVLRCSYSYFAVLQQANEG*

>MsexOR20

MAVSVLKRLLTYGDNIFEFNIKYLMLVGLWHNENWTRNQKLLYKIYDNTLDVLGLIYLTLTAIGIHGNMDDITAALAGVDKSLVAYNFMLKIIVFHFRKHQLRKLVKEIIASGDVVPEEHKILVAKLSLATTTITTIIVTIFTGISVLAGELPAKVWLPFDTSKNFMNLLAGVQICLVTFGVPICYRGLALKCFVSTMIFYLRDQLIDLQVKFKELDNFKDAEEEVRTNFKKIVKKHIRLIRYSKTIDNLLREYFLIQNLAITIEVCMNSVTLTLSEGISSAAYNTSWTSWPVDMQKDLLTVIIVAQSHFKLTAGGMAVMSLETYAQTLYNGYSIFAVLIDAVN*

>MsexOR21

MLVGFWHNENWTRNQKLLYKIYDLTMHVFGITDIALSAVGIYQTKHDLTVALAGVDKALVAYNFMFKIIGFHFTKDQFKKLVKEMIASGDVVPENHEILMAKLSLATTITSTIIVVIFTGSSMMVGDLPARVWLPFDISKSFMHLLAGVQFCLVIFAIPVCYRGLALQCFVSTLIFYLRDQLIDLQDKFKELENFQNVETELRRNFKKIVKKHIRLIRYSKEMDYLLREYFLIQNVAFTTEVCMNAVILTLIGFSQKTLAINFIAFLITALIHAFVYCYLGDEIIEQSKAYRLLLTIQAGPRGPSTMQKNLLTVIIAAQSHLKLTAGGMAVMSLETYAQTLYNGYSIFAVLADAVH*

>MsexOR22

MGWIERIKGFILKKSFDFDRPDICLYNFHPQLRILFALKGIFFNKQNSKLKIILPTYFNLLTILGMVFEGMFAHRGLTIKDYSFAIESFLYFIILTSTPLVYLCLFYHKDKIIQLLDDMNEEFKFVCSLGPRHRTPFLKGQLLIWKLCYAWYTLSISTGTAFMMFPVMALVYQTLFVTHTEKTIRPLAFTMWLPNDDPYRTPNYEIFLFFEMNYCVIIVQTFGVYIYTLFHLLLHHYFILDMMILDFEAIFDGLDESVAALPSKHPRRREVQLILNARIKRIVTWHNSVIKTINTLSIVYKPALVFQILLSSIMVCLIGYQIAESLDNGVIDFLFIMLGVCTCMQVWIPCYLGTLLRNKVFAVREACWNCGWHRNSLGTLIRLDILIIIQRTQVPLLIKLSDMSTVELETFSSIMSAAYSYFNMLRNSN*

>MsexOR23

MSSFCQTDIFKPNFFFWKCFGIWGGRTENKNYKYYSFSYLFVTLFVFNILLTINLIYTPLKIESLIREVIFYFTEIAITVKVLMILVMRSKILDVFNLLDCKEFQGDDEESKQIIETNHSFYRTCWKLNAVLSNISFASNVFAPLFINLIWTAKIEFPVCKYYFLSDEMRDKYFIFWFIYQSIGIYGHMMYNVNVDSFIAGLLLMAITQLKVLNAKFTKFKLEKRHEKHHILIQNKIQILRLNRYLKHYDCVLRYCEIIQDLLSVTMFVQFGMASAIICAIMCGLLLPSTTETFMFMVTYLFAMTIQIFVPAWLGTQLSHESCG*VFAAYNCEWIPRSMSFKRSIMIFVERANNPIQLTGLKMFPLSLATFTSIMKTAYSFFTLFRNLQDHDDGAN*

>MsexOR24

LLSLLTKYFFVLNAVLISVYNFSSPIIMLYQYIAKNKVVFVLPYAVLLPFPTDGWLSWFLAFVYSATCGCICVLFFTTIDVLYCVLTSHVCNNFSIISDQLQHLQVNNVNIIGNIVKEHQYILKLADDLEDIFTAPNLFNVLVGSVEICALGFNLTTGNLAQLPGTILFLTSVLLQILVMSVFGENIITESRKIGESAFLCKWYEMDEKSKKMILTIMIRSRKPQILTAYKFSIISYGSFSKIISTSWSYFTILQTVYKPPELIVKNNIN*

>MsexOR25

MELCKTIWRIITPTKALQQSSGHLETLFFESVYRVSYLMGLSTSDHDMFYLLYSTTVKFMITLLVCGELWYIFTETSSLDGIASSINVTLIQFITIYRYKNMMDHKDIYKKLATSMESPYFDTSNEKRKQLVVFWAKRNEKYLKLLLFLGNCTLAAWYLYPLVDDIEYNLIIGFHTPFSFKTPLRYPVVYLVVVIAFTYISHFVMVTDLIMQAHLIHLLCQFTVLADCFENLLDDCQHGFEDVPRNMLVNNKQFAAKYIRRLGHLVEQHKKILKHTVNLRNTLSRPMLGQLAASGTLICCIGYQATTTMTESIVKCLMSLFYLGYNSFELYIICLWCEEITTQSMNIGDAIYCSGWECGVTKLPGVRSTIMLVLARANKPLVLTAGGMYNLSLTAYTTLVKTSYSALTVLLRFRHE*

>MsexOR26

ITYLYLFGLPNFWIEDFKLPKWFMRSFDIFTKIINNVLYFFILMEMIAFFTQENLSERQKSDLLVYGISHPILYSYRVFISYKEDNLRAVLLDLVVTLKRVYNDVKVERQMIKKSLLYSSALVFSCILAMFFYTFDSILHVIRTGATFNVVITTWPKVEDRSTLANAGRIVFYILWWFFMSRVSGAYTTVICLTTCLSHQYTNLRSYFENLNNIFETNFDQAVKEQKYEDGFKVGIALHLDTLRCTRECHSICQGVFSGQIILNILLLVVLMSQMVNSERTLVTAFATASSASAVLISTGYFMWNAGDVTVEASRLSSAMYLSGWHNCHGRSSITIRKLVVITMFNAQKPVILKGLGIVDLSYQSYLSIVKSSYSVLSLLY*

>MsexOR27

MYTYVKVVVFWFNKKKVVNLLEFLHCKEFKAKEMEHREIIHKSIKRARFVMTFYSTMCVGAVSVGIVMPLTENFNILPTNVEYPHFNVYNSSTYVAIYLHHIYYKPATCIIDAVMDTMLAAFVASAIGQIEILTFNLRNFDVVAERRRKKAIKGNKYIGLQNNNNYFMKCILKECIVHHNHIMRYVSMIENAFSLASALQFMLSVMVLCLIGIQFLSIENPSGHPMQMVWMAIYLTCMLVEVFILCWFGNELIWKSTGLRQAAFDAPWLATDPKNAKYIILFMERCKRPMKVTAGKIFTLSLDTYTALINWSYKAFAVMSNMKK*

>MsexOR28

MAWFGFIHTPHTGDLLAAMVVLSLGISVQIGTLKFFYTFVYINKETNIVKYYLECDSLIVPGGRFSGNLLRALRNVKKRAIVYWLVIIINGITYVTKPMFMRGRHHMEDRYVIYGLEPMFESPNYEFAYFLMTAGLCFICYPPANVTVFLIVVVGYTEAQMLALSKELLHLWTDANEHYQKNINQHETTLINAQASKNKIINDYVRYRLKEIIKMHAFNIHLVRQVEFVFRGAIAIGYVFLTLGLIAELLGGLENTYLQIPFALIQVAVDCYTGQKVTDASLIFERAVYDCKWENFDKMNMKTVLLLLQNSQKTMTISAGGITMLNFSCLMSVIKSIYSAYTTLRTTMK*

>MsexOR29

MKNYYILKTYCSKIFLLGSGDFWYENKVIGDDKRILYRMYSCALFFIYGFMTVLEIMAALMGEFPTDEKRDSVTFAVSHAIVMFKIISVVFQKELVKTLNRKMVTICEHYEEQALMSEKYRIMKINVIVYFLIVYGSAACFVFEGLRKLFDGSHFVTVVTYYPDYEDNSMFANSVRILATVILFLMLMNMIVCVDSFTMVYLIMYKYKFITLRHYFENLSVTIDKLNTPGNEEVVAKMLTDGLVEGVEMHSKLLRLSKDIDKAFGTVMALQLCQSSGSAVSLLLQIALSDQLTFVASLKIVFFVMALFFLLGLFLCNAGEITYQAAQLSDAIFYSGWHVCRPRHSSRHVRKIVLLAIMQAQQPLVMKAFKMLELTYGTFILVVRATYSVFALFYAQDK*

>MsexOR30

LENAERPLLGPNVKALQFWGLLLPKYRLKRYFYLTMHILVTLFTFTECVDVYFVKSDMNLLLNNLKITMLATVSVCKVNTFLYWQKRWTDIIAYVTRADLNQRNTKDEKKLALIKKFTLYCRKITYLYWFLMYTTVVIVVAQPIFKYAFSKNYRDNVRMGTETYLQVVSSWVPFDKNTIIGYIAASIYQTYAAKPNSADIHHTGGRSSMHLEGPIRPIVSRITIHWPSFYNVVTGKTLALPNLIALQHI

>MsexOR31

MAQNTELFLGRPKKILTFFGIWLPSKQYQDLVKIYMILVMITQYSFVLFEIVYIINVWGDIEVVSEASYLLFTQASVCYKTTAFLIKKRKLVLLLEHMEQEIFASQSKDHEKILLELSLRIKRLCTFFLTSAITTCTLWAMIPLFDDAGHKTFPFRIWMPVTADESPGYELGYLYQMVSIYISAFLFIAVDSVALSMIMFGCAQLDIIKDKMQKVREVPICLNVDEKKEKLKSNNDLFTECVRHYQNVISFVELVEETYHANIFFQLSGTVAIICNIGLRISIVDHHSVQFFSMLNYMVTMLSQLFLYCWCGNELTIRSEVLRDWMYQCPWYDQDTKFKRTLWITMERMKKPIIFKAGHYISLSRPTFVAILRCSYSYFAVLNRANT*

>MsexOR32

MSCIMYLAVSEQLTMTLNSEDLYLNRAKFVMKFLGVWVPPNDENIIRKLYRYFMISLQYLFLIFQIIYIIQVWGDLEVVSQASYLLFTQACLCFKVTVFHVNVDLLKELLLQMNGETFRAQSFDHEKILESQAARIKRLLLAFMISSQLTCGMWAVKPLFDDVGSRKFPFDMWMPVTPESSPHYHIGYTFQLVTICMSAYMYFGVDSVALSSVIFGCAQIEIIKDKLMSIQPVDKNANDAINAFSDNYQQLVNCIKHHQAIVRFTDTVENAYHTFLLFQLVGSVGIICMSALRILVVDPRSMQFFSIVSYLSVMISQLFVSCWCGHELTATSEDLHTTLYMCAWKDQNTKFQKELCFAMLRMSRPLVLRARHYIALSRQTFIMILRMSYSYFAVLNQTVN*

>MsexOR33

MAETLLDKSLSKINILFRCSGTNIAIGKAAPTNTKRNRCIYVINFILLNTDVLGAACWFISGLKIEIVAVFSICAADFVFYNLCSYITIQFRLLQYDIEHVITGTRKSIYNDEMRGGRIRNKLVEIIKWHQELITCVNLLENIYSISMLYNFISSSVIICLTGFNVTSIEVSNAVYNSKWYLVPPKTRKTILLILTRAQKPYKLTAYGFADVSLKAFMKVLSTSWSYFALLKTVYSTE*

>MsexOR34

MKIFIDNANGTLWLSLNLLRWVGFLVPDNFEGRKKKLLPFYSFFWFMFIVGIYVIVQTGDLIQVWGDITLMTGTSFLLFTNMAFITKIINVMVRRDAVLAIIDEGDEVLRSERRIEGKAIVKSSNQETSRLLYLYGLLTVVTVFGWAASAEKGSLPLRAWYPYDTSKSPAYELTYLHQSVAVILLAFLNVSLDVLVTSLAAVCRCRFQLLALSLRTLCHDIPIDEKHLVSPEHKQIVHERLRLCVLQHQSILESAAKIKTCFSGHILAQFTVSIVIICVTAYTLALETRDNPIRLIAMFSYLLGMMMQVFLYCYQGDYLSEESSDIADAAYECPWYACPIPLRRSLLVIMARSCRVAILTAGGFSTLSLACFVSIIKASYSFFTVLQTSRRMSDHESTCCTNSTHPHKIFGSSSF*

>MsexOR35

MVVIEKILSFLEDPRYPSVGPHIRLLSLTGLWHPDLKSPKSRFKIIIFLVTVAFFLSQYVKCIIKFNTDDLKLILQYAPSHMGIIKTCLFQKDYKIWEELIDFISSVELKQISRKDENLDKVMKAYIRRNRRVSYFFWALAFFSNFSIFTEPYQKNNFNVNGTSTYLYIFDGYTPFSREPNGYYFSMCIQTMLGHIVSAYVIAWDTLVVSIMIFFAGQLKITRLYCTRMITANNEESHRNIAECHRFHTSLVKYQKTFNCLISSVMFVYLVVISVNLGVCIKQIAEIEDDLPTLVSSCVFLMACLIQLLLFYWHSNEVTIESDLVSYSTFQSNWAQSNKNIQKEVALLALTTRKKLVFRAGPFNVMSLSTFVSILRASYSFYTLLKGTN*

>MsexOR36

MAETLLDKSLSKINILFRCSGTNIAIGKAAPTHTKRNRCIYSFNFIWQNTDVLGAIYWFISGLKSGKSFTELTFIAPCIILSFLSVMKSISIIIYEKKVYQLIKNLRMMEAHERNRENTTERGKITEKGVNFLNLVINVLYVFNMLVLVCFALNPLILMILNYMKTNEIDFILPFPIAYPFDPYNIKVWPMVYLRQIWTEIVVVLNTIAPDFVFCIFCSHITIQFRLLQYDIEHVITGTRKSIYNDEMRGGRIRNKLIEIIKWHQELITCVNLLENIYSVSALYNFISSSVIICLSGFNVTENKDIVLVISFIAFLFMGLLQIFFFCFFGDMLIHASIEVSDAVYNSKWYLVPPKMRKDFTSNSNSCPTSL*

>MsexOR37

MKIFIHNANGTLWLSLNLLMWVGFLVPYNFECQKNNLLPLYSFFWFMFIVGIYGSFRTRNLIQVWGDITLMTGTSFLLVTNMAFITKIINEMVPRDAGLSIFYEGDEVLRSQRRIEGKAIVKSSNRETSRLLYLYGLLTVVTVFGWAASAEKGSLPLRAWYPYDTSKSPAYELTYLHQSVAVILLAFLNVSLDVLVTSLAAVCRCRFQLLALSLRTLCHDIPIDEKHLVSPEHKQIVHERLRLCVLQHQSILESAAKIKTCFSGHILAQFTVSIVIICVTAYTLALETHDNPIRLIAMFSYLLGMMMQVFLYCYQGDYLSEESSDIADAAYECPWYACPIPLRRSLLVIMARSRRVAILTAGGFSTLSLACFVSIIKASYSFFTVLQQVEE*

>MsexOR38

LSLPLQSSHVTMDLNFDKMYILTVFAMRINRSHPSIAKDTKWFLQLLPMYGIFSIMFALLINCIIHYDLKAKDFSSTCRNGCLCVLYFISTLSYYVMLVHQTTLKTIINTMNEDYAQALKFKADEQKVVLDYAKKGLYVCRQWLAMSIMGVGLFFVKNVLLYAYNYYVDDMKLVPLHDMTYPRIIEEKRDDIIVYASLYALTVYYGVFAAIMYMSFVPLGPVFMLHCCGQLELIVMRLENLFIKYTHEEANEKLKDIIRHLQNIYGFVKNIEKCFTGFYELTLKATTITLPIAVYEIIESCHRRELRMEFLVFIFGAMIISNSPCYYSHLLMENGEQVRLAVYCSGWEVVPIPRRLALLSW*

>MsexOR39

HVVSVEFGQLQGLHRLVYARRVRRQPVLHFISRIPIYHHPPGGTGYPSASHPYLHSAESDELVIELVGNKNPSWQTHCTSVLVQCVKHYIKLKRFSNRLNYICRPFYLTLILVSTMLVCMCSVKIATSEKLSPDTTKYYVHEFCFIQVVHMFCLLGQHVENECEKLEVAVLEKWHIFNKPHQTNVRIFHTAVSQRMPIYIFGTIPLSLPTFTWFMKTGSSFFTLVMSVLEEENA*

>MsexOR40

MTYKNRQHQELEMELSHLPGDYLKPLIACFDLLARCNIGFFHGNSSYFKKYWRYSYIISCVVAYYSSLTVYALKIFLGQMELFELAYVVPVFVVCTQAILKAIIVIIHKGEIRALVLQLGETWRTDNLTTRQLNKKNLLLKKLNFCYGVFRIVYSYLGTEFLLISLCSHLSTEFCLLREDLLNVKPVGNRRFRILQDSSNIELHDIVVKHQKLIKFSEQLNEIFNKMIFVNLSSVTITVCFFAFATKVARGPVDMANNFMAVMALILPIFNLCYYAEMLINASAGNKQESAYHSLWYVANKQYQMSIWFIIRRSQKPCCLTSLKFSPVALHTFTAVLSTTWSYFSLASSLFENEN*

>MsexOR41

DLIIEELVFYFTELAAISKVLTFVFFRDKLAKILDALEDPMFQAANGREQKIIDGAKRFNKRYWKIVATVSLTSHATHILSPIVEHLFLSVPLQLPTCRYSFLSENTIQQFIYPLYLYQTLGMHSHLWFNANIDSFFLGLMILIIAQLQILDLRLRTVTDVKKNDDIGQANTSEARANYSLTQLNKCIVHFDEVGKFCGLVEDTFSMTLFMQFSMSSCILCVVLFRFTLPAPFEYYIFLGTYMCVMISFIFVPCWFGTRVMELSVLLCSSVYECDWTAMPKKFKSNLQLFVERAKRPLTITGGKMFMLSLTTFTSIMNSSYSFFTLLRNVQTHD*

>MsexOR42

MASLWRKYFTKEIQVLKRVYEKSDYEDTYETPRKYLKWSGIRMKHNISKSLSICWLVYYWFCFVNIVFASVAEIIAMCMTASAGTFDDAIAIFQMMPCNGFCGLSLVKSFKMVKHRPVFENLITEIGNMWPQRLVDEEEHKIISSALREIKIFVKGYHWCNNYLMLSFLYPPFWELIKRLSGEKWEPKLHFIYWLPFDPLQPVYYECMLALQTWQAMTVIWTNMSGDFMFCLFLSHITTQFNLLSVRIRKLIYVPVDQQLIESYPLGQYSEEYLRKNKEPVDSYTPQQWEEKHFKEITEIVLQHQALIRLSRDIENMYSLTLLVNVVNSPLVICFCGFCSVVVEKWNETAYKSFLVTALSQTWFVCWYGQKLLDSSEGVAEAVYNSGWYRASKKIRRSLMIMLHGSQKGVGVTTYGFSIISLASYSTIIKTSWSYFTLLLNFSNK*

>MsexOR43

MLERLSKYLENPNHPLLGPTLCGLKCWGMWQPLGVNRIIYNAIHFFAILFVISQYVELWFIRSNMELAIRNLSVTMLSTVCVIKAGTFVFWQKSWNDVIDYVSGLENIQLSKRDRITNSVISEYTKYSRSITYSYWVLVTATVFTVILAPLVGFLSSSDKDLMLNGTLAYPEIMSSRLPFNRSRGFGYWVAAIEHSLICFYGGGVVANYDSNAIVLMSFFAGQLKLLSINCARLFNDNEVLKYSDTMKRIKDCHHHHVEIVKFSMVLNSLLSPVMFLYVIICSLMICASAIQLTAEGTSNMQRIWISEYLMALIAQLFIYCWHSNEVLHMSSKVDEGVYPSHWSAQNVRVRRSVAPPRRATAQAHRLHSRTLYQAHYIYFCSYSEGIVQLLYAAKQERRLRT*

>MsexOR44

FCTIAKTRAKPVNYKINKKKYNFTSITRTRIHFRLISIQPVDKNANDAINAFSDNYQQLVNCIKHHQAIVRFTDTVENAYHTFLLFQLVGSVGIICMSALRILVVDPRSMQFFSIVSYLSVMISQLFVSCWCGHELTATSEDLHTTLYMCAWKDQNTKFQKELCFAMLRMSRPLVLRAGHYIALSRQTFIMILRMSYSYFAVLNQTVN*

>MsexOR45

MDVCIMYLAVSEQLTMTLNSEDLYLNRAKFVMKFLGVWVPPNDENIIRKLYRYFMISLQYLFLIFQIIYIIQVWGDLEAVSQASYLLFTQACLCFKVTVFHVNVDLLKELLLQMNGETFRAQSFDHEKILESQAARIKRLLLAFMISSQLTCGMWALKPLFDDVGSRKFPFDMWMPVTPESSPHYHIGYTFQLVTICMSAYMYFGVDSVALSSVIFGCAQIEIIKDKLMSIQPVDKNANDAINAFSDNYQQLVNCIKHHQAIVRFTDTVENAYHTFLLFQLVGSVGIICTSALRILVSEDLHTTLYMCAWKDQNTKFQKELCFAMLRMSRPLVLRAGHYIALSRQTFIMILRMSYSYFAVLNQTVN>MsexOR46

MVGVSLPFKYNTPFRYVITYILVGIAFNYTSHFCMVTDLIMQSHLIPLICQYSVLADCFTNLVSDCEVGFEGIAREHLVNNKKFVKLYLRKLGNLVEQHKFILNHSIELRTILSVPMLGQLAASGILICFVGYQATTTISVNITKCLMSLFYLGYNMFTLYIICRWCEEITIQSQNIGEAIYCSGWEQGMSKIRGVRTTILLVMLRSSKPLIFSAGGMYKLSLTSYTTLVKTSYSALTFLLRIRRE*

>MsexOR47

PSPSLRMVRQRGLHSRLLLRRSHGLQLPLVLVFCHHRDPERVAGCVLCSHLPCRCGTGLKPQDSSLDRIICMGILTVQEITSIMVVASYDMTLLFLFSHTTAMFQILYEDVTNFRELAENYYDSSEMKSAVFERLKNLLIRHSSILRTVQKMQDVYSVVVGIGFGLNAISMCLFFVLPIDVCLNFAPLIFHSLFVFFLYCYQGQRLTTASEKFEIAVYSCGWEHLGVRDQKTILLMLIQAQKPVIMMAAGVIPIRIRTFAYTLQNIYKFVTLFKI*

>MsexOR48

SLDHSQSSNKEVSVICSFQILTVLLDFPHFHYENFSIFNHTHSQSLDFRDWLYEVSWYDQDKRFTSTLSVLLQCTKKPLEFRAGHYVLLARATFVSVLRCSYSYFAVLQQANEG***IR**

>CpomIR7d

SVDMPIDGELNDDLMLFNKNITAAQNLAINAAKIALNNFEWRYVTMVFHNSSILLGLTAFMQIYRKSVIVGKGTFLHGKESSADRISQFVIFGSDLVDIMCTLDWMRKREFDNTGKFIVICNNCDERKAMDIFWNHKILNVVFINDSSGTSSLIGFTYSIYDNQKCVISPPEPLLDSCIHNSCMGVYPLKLRNLHKCQIIVSTFEQVPFMSLKTGTPIGADGDLLLLIAEALNATLKVMTPHRGAGWGQLDKDGNWLGSLADVYHDLANFSMTSAAITLTRFKAFHLSTDYHSINMAWVTHPAVPLPGWQKLLRPFKMKARISLAVTFVLIILVAVFVKSNLWAKLSKRINTSARPQTCVLFYSWTICMGMPATSLPSKPTFLTMFLLWMFYCFMIRTFYQTSLIHAMKDNLNYPEFENLQDILNSGYPFGGVPALKDFYIDDPEVYNNWKSINSTEINDMMVSLSRGMKYVLAMNKVTAQSFILKHYGDIHIVPQMIVTSPTVLYFKKFSPMVQSLNLILDRLVEGGFTEKLYKNHASTHARKKTDSTAPMNFEQYMGCYVVLAAGWIVSILVFICEVYCYKFSV

>CpomIR8a

MDFCCLFLAIFIFNLGCVASELSLRFVFIIESHEQDLPQLIGRALKFAEEAQPDLRVSEAIVSLDRENEDESYRQLCSALSNSVSIIVDLSWSPWDSLEELSSTAGVPLVRARLGSQHLVRAVDEYLESRNATDAALLMESEADVDRTLYELLGESNIRVWVHAGLTRDSARALKTMRPEPSFFVVVGSGAFATDTYKRAVKEKLVRRDYRWNLVLTDYSNLELQPVKPAMVLQVDAAECCKVMGQKDGCSCSQDFERKQPILSALLQLLAETYSKLDDDDFTTRVDCDNLVPENGTRSKVYRQLAEELGASNESLFYWDGERSGIFLRSRFILSTLKPDIGPQHAAIWSADDEYKLLPGVTLEPLRQFFRIGTAPAVPWTMPKLDSNTGEPMFNEDGEPMYEGYCIDLIQKLSESMDFDYEIITPKTGTFGRRLANGTWDGVVGDLMRAETDIAVSALTMTAEREEVIDFVAPYFEQSGILIVIRKPTRKTSLFKFMTVLRTEVWLSIVAALVLTGFMIWLLDKYSPYSARNNPDAYPYPCREFTLKESFWFALTSFTPQGGGEAPKALSGRTLVAAYWLFVVLMLATFTANLAAFLTVERMQTPVSSLEQLARQSRINYTVVEGSTIHQYFINMKFAEDTLYRVWKEITLNATSDQSQYRVWDYPIREQYGHILLAINASMPVPDAKTGFRQVDEHTDADFAFIHDSAEIKYEVTLNCNLTEVGEVFAEQPYAIAVQQGSRLQEELSRALLDLQKERLLEQLAAKYWNETARQQCPDADESEGITLESLGGVFIATLFGLGLAMITLAWEVFYYKRKEKNKVRQEDEETKPKKAFEKDLEKKIAGGVARLRKRDKKEKKGQVTIGDTFKPVSEKDGVSYISVYPKTEYKP

>CpomIR21a

MRFLRTALFNYILLHYVISQEIEYYPSQASSFARKLVSEFNSEPYQHKHDLFKREAQWRKFNNNDDTEFTKNKTQKRAVDPVFHGHPKTREELWNERIINESLAFDQTPSLISLIHNITLTYLNDCIPIILYDSEVKSKESYLFLNLLKDFPIAYVHGYINENNELAEPKLVRATRECIHFIAFLSDVTKSAKILGKQAESKVVIIARSSQWAVQEFLAGPQSRMFINLIVIGQSFKDGDDDTLEAPYILYTHKLYTDGLGASQPVVLTSWSHGKFSRQVNLFPRKMTEGYAGHRFVVAAANQPPYIFRTIKTDADGGNPRVVWDGIEVRLLTLLSQMNNFSIEIKEPREPHLGSGESVLKEITGGRADIGVAGIYLTSDRIRDTDMSFSHSTDCAVFVTLMSTALPRYRAILGPFHWTVWLALTLTYLFGIFPLAFSDKHTLKHLLHNSGEIENMFWYVFGTFTNCFTFVGKNSWSKTTKITTRLLIGWYWLFTIIITSCYTGSIIAFVTLPVFPETIDSIQQLLDGFYRVGTLDRGGWEKWFLNSSDPKTNKLLKKLQLVGDVPSGIRNTTKTFFLLPFAFLGSRAELEYIIQSNFTKTKKSKKAQLHISNECFVPFGVSLTFPNNSLYSSKLSGDIARILQSGLMDKIENEVKWEMQRTPSGKFLSAGSGTLKLGAITEKGLTLADTQGMFLLLAAGFVLAAAALISEWMGGCSRKCRPQKKEDEPSSAHSREHLIPTPKSDVDSEIKVISDSAESRFRLNPRPDSEDSRDSLEGTIINVTKESIIIHNNYHTSNWDSRRSSSVDIDKEVQEIFEKDEKRRRINSGTVPLKDNQREATASKGAFGDHLSDH

>CpomIR25a

MASLIILLLFLFVPDSFSQTTQNINVLLINEENNALAEKSFEVAKEYVRRNPTLGLAVDPVIVVGNRTDAKAFLENVCRKYNDMLSAKKTPHVVLDFTMTGVGSETIKSFTAALGLPTISGSFGQAGDLRQWRNLDANQTKFLLQVMPPADILPESIRAIVTKQDITNAAIIFDEFFVMDHKYKSLLQNIPTRHVITPVKSFNRDEIKTQLRSLRELDIVNFFVVGSLRTIKNVLDAADENQYFGRKTAWFALTLDKGDISCGCKDATIVYMKPTPDAKSRDRLGKIKTTYSMNGEPEITSAFYFDLSLRTFLTVKSLLDSGKWPNDMRYISCDDYDGKNTPNRTLDLKTAFHEIKETPTYAPFFIPEDDPMNGRSYMEFNTDLSAVTVKDGASIGSRNLGSWKAGLSNPLSLTDPQNMSDYSAQLVYRVVTVEQKPFIIRDDEAPKGFKGYCIDLIEEIRQIVKFDYEITLVPDGNFGTMDENGNWNGIIKELVEKRADIGLTSLSVMAERENVVDFTVPYYDLVGITILMKLPRTPTSLFKFLTVLEDDVWLSILAAYFFTSFLMWVFDKWSPYSYQNNREKYKDDEEKREFNLKECLWFCMTSLTPQGGGEAPKNLSGRLLAATWWLFGFIIIASYTANLAAFLTVSRLDTPIESLDDLSKQYKIQYAPLNGSAAMTYFERMAHIEVRFYEIWKEMSLNDSLSDVERAKLAVWDYPVSDKYSKMWQAMKEAGLPNSIEEAIQRVRDSESSSEGFAWLGDATDVRYYVLTSCDLQMVGDEFSRKPYAIAVQQGSPLKDQFNNAILQLLNKRKLEKLKENWWNNNPEAMKCEKQEDQSDGISIQNIGGVFIVIFMGIGLACITLGVEYWWYKWRKRPIIGDVTQVEPSKTTRNNADNSTTKIGEGFTFRSRNMGLSNFRSKF

>CpomIR41a.1

MIMPSKLFPVEILLNILINEHLQEYFCLTFVTETKLTVNIPINMSLMIIQPNNSVLAEQILDASEKGCSDYIIQMHEPENFMIAFEKVNHLGDIRRSVKKLIFLPVQDDMNNRSVLTNILALRETGFVANILLVVPSLQSSGDCKVYDMITHTFVGSDEDVQKPLYLDRWDSCTGHFERGVNLFPHNMSNLYGKTVKVAAFTYKPYVLLDLDPSLNSLGRDGMEMRIIDEFCRWVNCTVEIVRDDEHEWGEIYENNTGVGVLGNVVEDRADIGITALYSWYDEFRVLDFSAPIIRTAITCVAPAPRILTSWDLPLVPFTWTMWMCLVFTFFYASFALSIAQRSTDNVFLDTFGMMITQTREDATSWRIRSITGWMLVTGLVIDNAYSGGLASSFTVPKYEASIDTVEDLVDRKMEWGATHDAWIFSIMLSEEPLIKSLLSQFKTYPADILRQKSFSRSMAFSIEHLPAGYFAIGEYITKEAAMDLEIMLDKIYYEQCVVMLRKSSPYTAKLSELVGRLHQSGLMLSWETQVALKYLDFKVQLEVRLSRARKDLEEIEPLSIKQLLGIYIFYFGGVVIALLVFFGELLSKCSKPSIVL

>CpomIR41a.2

MVKMLIPSTIYFPIEILLNTIINNYLQTSFCLTFVTETELMINLPLNMSSMRIIPNNSELVQQILETSEKACTDYIIQMDEPRNFMIAFDKVNHVGDVRKSDKKLIFLPLEDEFYNPSVLTDLLSLKETGYVPNILLITPTGQKSSDCKVYDMITHTFVGAEEQIQNPLYLDRWDCCTEVFEKEVNLFPHDMSNLYGKKVKVGAFTYKPYVLLDLEPSLAPLGRDGIDIRFIEEFCRWINCTVEIVRPDDGQEWGEIYENNTGIGLVGNLVEDRTEIGITSLYSWYEEYRALDFSAPIIRTAVTCIAPAPRILSSWDLPLVPFSWLMWMCLIATFFFASFALFVAQRSTDDIFFVTFGNMIGQSPGDSSSWRIRSISGWMLVTGLVIDNAYSGGLASSFTVPKYEASVDTIQDLVDRKMEWGAPVDAWLYSMILSEEPLIKSAISQFKVYPPETLTKKSFTRSMAFSIERLPAGSFAIGEYITKEGAKNLELMVEDMYYEQCVVMTRKSSPYTAKLTELVGRLQQSGLLLCWETQIALKYLDFKVQLEVRLSRTKKDIDGVEPLNVKQLLGIYLLYFGGLSISIVVFIAELLIKRGKAVIVI

>CpomIR60a

GFYVQATMLKIICLLSIGVNAKVNPHGPTVVSDFSSCVSEIIDKNFAQSGLLFFVDTFNVSTPVAGIRNGIIKSVHTKLKYSVKIAIPTKKDKGICVNNDKTAIEISVKSRMDHFEATSLADYFILIIEDYKDFSYIASRLIRAISWNPRALFILVYFSISNSDDQNIRHAEDMLFCLFKVNVINAVVIIPEVNNVRRANIYSWRPYAPPKYCGHYNESIRNRLIVENVCERGKIKYAKKIFESKIPSDMMGCSLKVLALERQPFISHNPLDPNIESLLINQVAKRYNLSLRYEILNSFRGEKLFDGDWTGALKELTYKKGHLLLGGIFPDDEVHEDFECSSNYLADSYTWVVPRALPKPAWLALFVIFQKTVWLTVITCFVFIALSWMVLAKLSKDPTYRTNLDHYFINTWLSNLGFCAFSRPITNSLRLFFVFINIYCILLLTAYQTKLIDVLTNPSFEYQISTVEELVESGLKFGGSEELHDIFENSTDSIDNYFLDGWIDIADIRDALRDVAIHRNFSLMCSRLELAYVSAIIPELSDQFGKYMYYAFPTNVFTVPLEAVSMKGFPFMKGFSRTLTHFEQHGVNNGVIVYFGGYLLRQRALLLNKFKIEHNSRDPLSIQTLQGGYLALMFGSVCGTFVFIVEIILNTKFVKKLKIL

>CpomIR64a

MNLTTYSALFTIFSTAEINLITDVFKHKHLHFGTIFHCSKPENAIFLQKHLKKMDLRFSTIMMHSNASHFKQTNDSRVGIVLKTSCENWTQVFEHFNCNLFEKSLYSWLIFTDDLSSASEALSRYPIEVDSDVAIIYRQEKSYYIYEVYNTGYFTNGRYHVEPVGYWYYKLRIKGHRRTNLDGIVLRSAVVVTHSIGHQTFEEYISRLKPEVDSLHKLKYFTLLNYLRDMYNFSLIVQRTNSWGYVTNGSFDGMVGTLQRGETDIGGTPVFIRADRAKFIYYVTATWPSKPCFIFRHPKHPGGFLTIYTRPLSYNVWLCIIALLVFAGSLLCVLIKLRVTRTAGDDGDLSASLALLSIWSAVCQQGTTVNLSANSVRLVLFFSFLFSLFVYQYYNALVVSTLLRAPPVTIRSLEDLLRSKLKAGVEDVLYNKDYFRRTTDPIALELYSRKIASSPRPNFLPPDRGMALVKQGGYAYHADTAYSYPIIRRTFTEREICELQEVELFPPQTMFAVMKKGSPYIKHLSYGIRKMAESGLMQRLKTIWDEPKPLCVRTPDSSIFSVTLREFITPLLLLCLGMLAATVVFMAEHVFYRLQWKRIQFRH

>CpomIR75dLQVQTRIFAGMELVSFALAYFAAKRLSLLTAFLCWRPEELSALCRDAQRQGMRISIADWTHLPPLEPYATHREGMLLDVTCPDAPLVLEKASSTRAFNLRHTWLLLHNAPFNASLMEVTLDSTLVLPDADVAWVANDQFLDVYRIKHDQALITMPLGDDAARVALPAAPTRRRDLNNVYLRSSTIISQPQFFKGWNDLTVRQIDTFPKLTWPLMHLLADDLHFRYNLIQVDLYGENRNGSFDGLAGQLQRQEIEVGITSMFLRADRMQVLHFCSETVELRGAFMFRQPSKSAVSNVFLLPFSRGVWIATALTLVLAAVTLALLARRPRLXXAVDASLEQLSIGEAVIFTVGTACQQGFHIVPELASARVVMFCALMTALFAFTAYSAKIVAILQTPSDAIRTIDDLTNSPMTMGVQETTYKRVYFAESTQPATQRLYRHKLLPLGDRAYLSVVDGVAAMRTGLFAFQVEEPSGYDIISKTFTEREKCGLMQIQAFKLPMVAVPIRKHSGYKELFATRLRWQRETGLMDRTRRIWLASKPRCDANSGGFVSVGIIDILPALHVLAAGMVASVLFLVLERSMARLKCCGVRGS*

>CpomIR75p.1

MDIWKLGAIIWLFKSHVEGRAGIGKFLTSFVDNERKPTTVVFHGICWNNSVKLHVMKELSKAGIRSSQSMSKRTSLIDHTVLLLADLNCTGTDDLIINATQRELHRLPYRWLVLSDAPRFGRSSLWDLFLVDSELVLATVDGAGYSMTEVYKPSPTSPAILTPRGTFHHVLTDTRPHRELFRRRRDLMGVPLTITNTIQESNSSIYHLLQEDSLELEHDLISKNSYTLAKVAFLTLNSTPVATFTNSFGYLQNGQWTGVIKELLEYNADIGTNVGMSQSRLKQVMFLDPLDNGRARFIFRQPALSLTANIFSLPFSPDVWIATGLSSFVAGVAYYLSTRLIKTRAEKGTVRDAYLLTMSALSQQGCEVQPRHVSARIVLWVVFTSMMALYAAYGANIVVLLQAPSTSVNSLATLAKSKLALGAADVNYNHFLFRASSDPVRNDIAKRINSDKGPKAFYGLTEGVEKIRKGLFAFHSVVEPVYRQIDRTFQEKEKCDLMELDYIGYAAFHVPGSKKSPYLELLRVTFKRLREVGIKSAVNFRYEARRPSCKESIAMFSSVGITEMRPVLIFMAYGVALSVAVTAAELLVFHANRYRLRQQRLAVQLGRI

>CpomIR75p.2

MKILFSFVIVLFLSLGKAFDDNDINMIVSFVTLDERATAVLTPYVCWSTYELTSLAKSLHDTGISMAASLQPKRPELFLQNLVIVADLRCRRTDDFLIKASDEGFFKSPYRWLLISQDQTELNVLDQLAMLVDSDVVIAQRRGADYQYVEVYKIVENSQLIYNTRALWRPIDKNNNTAIITYYNKSKVVANKYGAVEDYRKSKILSTRRMDIRKHTLTMVNVITDSNDTRKHMDDRLNLHQDSITKMSYMVVKICFEMMNSTEKLIFTNTWGYVDKNGSWNGIIERLIKKEGDIGTLTIFTQERLKIIDYIAMVGTTAVRFVFREPPLAYVSNIFALPFTGAVWLAVFICVLACALFLYITSKWEATMGIHPMQLDGSWADVLILIIGAVLQQGCTLEPRRAAGRIVTLLLFIALTILYAAYSANIVVLLRAPSSSIRSLQDILNSPIKLGASDFSYNRYFFKKLNEPLRKEIYNKKIAPKGKKANFYTMKEGIEKIRKGLFAFHMELNPGYRLIQETYQEDEKCDLVEIDYINEIDPWVPGQKRSPYKDLFKINFIKIRESGIQNCIHQRLHVGKPRCLGAVNTFSSVGIMDMYSAMLATLYGMFMAPAVLLLEIAYKRLMVAREKRMQHNNSHSHT

>CpomIR75p.3

VRRRDLRGADVVLPTVLLHNQSLEDLPDYLHRERDTLTKVVYYMCTHLVEWVNGTKIWNRTTSWGYLQPDGQWDGIVREMQDGRADIAGSCMMAQKERVKYVNYVLAPSKIEAMFVFKKPALASVTNIYVLPFDIGVWVSIIVLIIISSSTLFLSSFGEDRMKNYYSKSWLQKMSDGFFDTLCLMFQQGTAADPLSIASRQILLLGLMAFMFLYTAYSANVVALLQSPTNDINSIETLLTSPVACGSQDVVYAWQMFGHESRPIHRLLADRKINSQGKKGFLSVEDGIRKVREGMFAFHVEQTAGFDQIQKTFLEDEKCNLGFIKYMSTTSPFVAFSQITPIKEMLRIGANRIMEVGVQSRSARRLVPERPRCGASAAMFNAVRLSDVAPAFRVLLAFYALTLPILGLEILVKRREMKRGIFESKSVDTATAEITNNIII

>CpomIR75q.1

MYTSLTIVCAIFICSSFALSISKENDIKVIVDVIQSFNKPTDVISNVCWTNIYKKKLTANLAAADNPRSIKFVNDIHAKDLVHPEKVTFLIDVRCKDSGGFLNKAASRKYFGRPHRWFIINTPANEVSVPLVIDKMHLLPDSEVYVMQLINNSYSINLIYKIKPNREWIIENYGNWSTENGLTISRRAKKVALVMRRRNLARASIVTSMVITDNGSFADLETLRYKQIDSVTKGGFHQLTALYEFMNASREFVFTDQWGHHVNGTWHGMVGHLADGTAELAGAILFITKERMPLIEYMSHPMESSIKFLFREPPLSYQNNLYLLPFQASVWYCVGSFVLVLIFAMYFSAYWEAKKVADEKQKADDTTVLVPTISDVTIFVMCAISQQGSTVELKGMLGRFMILILFLVFLFLYTAYSASIVVLLQSSSNQIRTLTDLLNSKLELGVEDTPYNRYWFMNEKEPIRRAIYEKKIAPSGSKPKFFDLTEGILQLQKKPFALNCNLGVAYKVMERYFYEHEKCGLQEISYLQNNNPWQAVRKGSPYREIFKIGLLRNAEFGLNDRTNRIMFSKKPVCSVRGGSFVSVSLVDCYPILLLLLYGMILGVMLLLVEILYYRKMNTRP

>CpomIR75q.2

MVADVIRAMQRPSAVIAMLCWSSNLKLQLYSALEGENVTQITMMQFLKAGTVPERHAQDQHIVFLADLDCPDIISYFQTSSLNKHFRSPFRWILIDSGNNDTSQSYIPNAVGNFDILVDSEVILAHHLGDGSYRLHLIYRIGNNTDWKKEFYGTWDERRRLQKQVMEGEIILRRIDLESYELPICYVLTDNDSINHLYDNVNDHIDTITKVNFPTTNHLLDFLNASRKYVFANTWGYRVNGTWNGMTGYLVREEVEIGGSPMFFTSERISIVDYISSPTPTRSKFVFRQPKLSYENNLFLLSFRASVWYSSIALLLLLVIVLFIVTIWEWKKTRGHEDKKLEADSGILRASVVDVVLLIFGAACQQGSTVELKGSLGRIVMLILFLALMFLYTSYSANIVALLQSSSSQIKTLEDLLHSRIKFGVHDTVFNKYYFSTATEPVRKAIYETKVAPSGSKPRFMPMDEGVKKMQKGLFAFHMETGVGYKFVGKYFQESEKCGLKEIQYLQVIDPWLAVRKNTPYKEMFKLGTKRIQEHGLQSRENRLLYEKRPKCSGQGGSFVSVSMVDCYPALLVLFYGAVFSVGLLFIEILTKRRNDILRKISRAKTLGVDVEDY

>CpomIR76b

MTGLELIVSSICNATFCEVVYDNPITDTLLPAQKKELLKIAEDLNGKHLKIGTYDNYPLSWVHTEDNGKLTGRGVAFVVLDILRERFNFTFDVVTPLKNFEIGIEGRMEDSLIGLVNSSQVDMAAAFLPIVYKYQQFVDFSSILDKGVWMMMLQRPKESAAGSGLLAPFEIQVWYLILAAVLSYGPCITLLTYLRSKLVRDGEKNISLTPSFWFVYGALLKQGTTLAPEANTTRILFTTWWLFIILLSAFYTANLTAFLTLSKFTLDVEYPEDLYKKNYRWVAPEGSTVQYVVNDADENLHFLSKMVANGRAEFRSVNADRQYLPYVMGGAVLVKEQTAIHHLMFEDYLKKTKAKVPETKRCTYVVAPNPFMEKLRSFAFPKNSKLKLLFDPVLTYLLQSGIVTFLEFRDLPSTKICPLDLQSKDRKLRNSDLSMTYMLMGVGLATAIAVFGGEMIIRYYVRIKIRKNRGERTRTKTVKTSKHRRFRIQDDSHPPPYDSLFGQNSRYKMNGDSTTKIINGREYWVVGTVSGDIRFIPVRTPSAFLYQRDK

>CpomIR87a

MLLLRTIHSGIKYSVMVKDSFYKHANASHFPEKAKNYMLILEEKSELVRNILQLNKLPTWNPLAKAIIYYQLLPDEDGETIAKKFINELREYKLLKSIVFIYSPDDAGLISYTWAPYSDTNCGGECDSVYILDTCKNSIVKQKNAQREMFPLNMKKCPLVTQAIISEPYVMPPVRQLTNTSYPDAYEFQKGGEINLVKLISEFTNMSLIVRISDVPENWGLIYPNGTATGAYGILRNDSVDLVIGDIEVTRTIRKWFHPTVSYTQDEMTWCVPKSAQASTWNNLVIIFQWTTWVATLLSIVTMGLIFHYIYYRENDRKVTKLPTNSLLNTFSMILGWGASFKPKTATFRILIFAWLFFGMIMSISYESFLRTFLMHPRYEKQISSETDLIQSGIPLGGRAIYRSYFETNNASSFYLYRKYISTSFSEGIKRAALERNFAVVASRRQAEYQDQKLGKGEQLLYCFKEGNNLYKYGVVLLARRWFPILERFNNIIRSVSENGLIEKWNQELFIHTVGVDGTSKVVPLGIRHLLGAFIFIGIMYAASVIVFVVELLLNVSKKRKGNKPICRACLSSKYSSRR

>CpomIR93a

MRIWVVLICVVGVRGEEFPSLITANASIAVVLDRQYLGEQYQPLLDTLKDYIKELARVELKHGGVVVHYYSWSTISLKKGFIAVFSIASCEDTWSLFSRAEEEELLLFALTEVDCPRLPPDSAITITYTDPGQELPQLLLDLRTTRAFNWKSAVILHDDTLNRDMVSRVVQSLTSQIDDEDVPTISVTVFKMRHEINEYLRRKEMHRVLSKLPVKHIGENFIAIVTSDVMSTMAETARDLFMSNTQAQWLYVISDTSIRNSNLSSFVNALYEGENIAYIYNITDDREDCKNGLMCYSEEMMNAFISALDSAVQEEFDVAAQVSDEEWEAIRPTKIQRRDTLLKHMQQHIAVNSVCGNCSTWQAMAANTWGSTYGGNVQADNVAAPDNETNEAIQKIELLQVAYWRPSDGLRFTDFLFPHIVHGFRGKVLPIITYNNPPWTILKANESGSISSYSGLIFDIVDQLAKNKNFTLKLIFPGDMKDVLSNKTVTNDMYSQSAKLTMMAVARKQAAFAAAAFTVLSDRNPGINYTIPVSTQSYAFIIARPRELSRAMLFLLPFTTDTWLCLGFAVVLMGPTLYVVHRLSPYYEAMGVTRQGGLATIHNCLWYIYGALLQQGGMYLPRADSGRLVVGTWWLVVLVVVTTYSGNLVAFLTFPKQEVPVTTVSELLENRAVYTWSISRGSYLEFELKNSDEPKYVSLLKGAELTSDSSGLEGNLASGSPLLSRVRQDRHVIIDWKLRLSYLMRAEHLATDKCDFALSAEEFLDEQVAMIVPAGSPYLPVFNKEINRMQKAGLITKWLSAYLPKRDRCWKTSSVTQEVDNHTVNLSDMQGSFFVLFLGFFSASSVLLMEWFYHRRKSQKEDVAIKPYVE

>CpomIR1

RMTMWRLLFLVASAASLPTDWPHMAVDYFQHKHVKYVAHLSCKDAAEIKGVLRLLMNEGIRAAVGLIDQGPMNIMPLLYQYEASVGVLVDGDCINTRDILNNASESMMFDDTHFWLVMNDNCSMGFVEDTFLDLKLSVDADVVVASYCGDIYQLTDVFNFGRVQGNVLETRELGAWTSERGLEIVLQGFKYYNRWDFHNLTLRAVSVIRNSSKEFHEGMLYEPGFTVGVAAMTKISSQLLNLLKEMHNFRFNYTIVGRWIGTPERNSTKAMSNMLLWRDQDISSTCTRLFSNWLDWMDPFFPSVTELETKFYYTISEKGIGDYENQFLTPMSPEVWWCAAATGVVCALXXXXXXXXXXXXXXXXXGVFSVLAAGFQQDYEDGHQTKKDSSSRKLALLVVGLTSMLMYNYYTSSVVSWLLNAAAPSLDSLDGLIKSDFELVFEDIGYTRQWLDNPGFFYYMGYKNEKEDELRAKKVTNVKRTLPLFESIEDGIELMRTGKCAFHTEPYTASQVISRTFADKDLCSLAGLQIMPPSYVYVMGQKNSPYRQFFVWSMMRLLERGHTRATRARVGGQIPPCSGLTPRSFKIFSI

>CpomIR2

MEEETFTVASTPDCMTVLQDERNDPLWRETQVGCKVLLLLQEIHKFNITYSKGPYHYNERGFYEYVDEVDIYAKPRSLWSPVMVNYTPIAPIMDWKFGYILRHPFNIQHFYSLAFSKPAWHFIVAMILLVSVLFYILNRAEQKLTGENLKCYFWSELLIAFGIICQHYISINPMELTSRRIAFISFFMFSYILYSYYTSTLLSDLVYDRDNEMDLETLAESDYEHAVLDSVTSVFKVLVEQLRNNNKMFPQRQSFTENKLINHRVVNISTGLAEVKMSKTALLSDYVSIHSGVIQWFSESEVCDLIKVDIFSNVLKYLVTSKKFKYIDEFKISTLRAYEAGVLQRLLSPHPIQHFSPTCVSSHFQADIGLVRKPFILLALGYVLCGFILLVERVYYNRYKVWPYVN

>CpomIR3

LSAKMVGKSILFFLLSSVAGLEDRDIDFSVDFLKARDVKYICMLTCGDRTWNKKFAKNASKSSIAVSYVRIDDSLSDLDSVRVCLSPEFTDVGVLIDTKCPLYEEVLMYASENLLFDANHKWLIIDIDTWISNISTVFNVETNENFSWLMNTLEKLNMSIDANVMLSLQKGSENNIYEVYNFGKLRGGNVVVKKLGNWRNRADLIQHLNAYKYYRRWDFENFTINYVAVMSTPPKVFDVNMLVGDTPAPGVAVMTTTVTRVLLEIAELHNIRYNYTIVDRWIGKFERNTTPVVATLLYFKEQDITPVLRVTSEVFQRVDMVSPPITSIETRYYYRIPTTGPGKFENQFLRPLTKGAWGCVIAVISLCALVLFLTVRAETRPAALQYAVFSVAATFCQQFFEDGGYDDPRRESSARQLTILVTGASCVLIYNYYTSSVVSWLLNGPPPSINSLQELLESPLSLIYQDIGYTRSWLQNPKYYYNKKNSEVEDQLRKLKVFKKKKGEPLLVPLEEGIEMVKAGGYAYHTEVYNANMLISRSFNQEELCELGSLQSMEETPVYIAIPKDSPYKEFFNWKLVAKNELKFIGKMKHRILQGHE

>CpomIR4

MIIPAVAAFFKYKIVSSIIIFTCGNEFEQIRLVRQLSLQGMRATVSCDPGILNEEHKTLQGVLYFNRPNDTLLDETSWEHFSMWYKWLIIGNEVPSRLNHTTRYDADITLLGLRQLGAIDAIDNSSVAYHESILFEDLYVHLRDGVSRHPWAVWTPAGFQPLYELERIRRRHDLKRYTMRIPTPVGHYDDSYEGTFADYVMDNSQPGRDSAIRCGYGTSSLILEWLQAKEVILQMEQWSTDAGNKSMFTRLAQGTSELSGGILRMQHKRLLKLDYVIPLWIFKVGFTYVAERESSSNMFVIPFTGTTWAACAVVTLVLAIAQRATAKQESEKEGAFVAVMATWLQQDASAVPEGASGRITFMALSICSMLVYAYYSSAIVSALMSAGSSGPTTLRALGDSRYRLASEDYEWIRAQMFDVYIPNWPEMEYLKRKKLQSMANFYLDWQAGMQLVKSGTTAYHAEYNHVYPLMSVLSDDQVCKLQYVDTVPPIMSWLVTTRRGQWTNLLRIGGDWLHETGLVKRMLSRWQLKPPPCRAALLAERVSYGDVAPLIILTVVGLLTSVAVLFLERAVAKWRAKKTDKSS

>EposIgluRMQGLTPSPMSILDSLCKEFLAVNVSAILYLMNHEQYGRSTASAQYFLQLAGYLGIPVISWNADNSGLEKRASHAALRLQLAPSIEHQTAAMLSILERYKWHQFSVVTSAIAGHDDFIQAVRERVTALQDRFKFTILNAVVVKKPADLNELVTSEARVMLLYATREEAADILSTAGDLHLTSENFVWIVTQSVLGSMQQPNKFPVGMLGVHFDTSSSSLIAEIATAVKVFAYGVDSYVSEPENARHPLGTRLSCSGAGAGEARWSTGERFYRHLRNVSVDSEAGRPSIEFTPDGELRAAELKIMNLRPAIGEQLVWEEIGTWNSYPKERLDIKDIVWPGGLHTPPQGVPEKFHMRITFLEEPPYINLAPPDPISGRCSLDRGVICRVAPEVDVAGLEAGTAHRNSSLYQCCSGFCIDLLQQLAEQLGFTYELSRVEDGRWGTLHNSKWNGLIADLVNKRTDMVLTSLIINSDREAVVDFSVPFMETGLAIVVGKRTGIISPTAFLEPFDTASWMLVGAVAIQAATFSIFFFEWLSPSGFDCSTGQNSKRVPQNRFSLCRTYWIVWAVLFQASVHVDSPRGFTARFMTNMWAMFAVVFLAIYTANLAAFMITREEFHELSGLDDPRISRPLAIRPPLKFGTVPWSHTDATLAKYFREPHAYMGQYNRSTVSAGVSGVLTAELDAFIYDGTVLDYLVSQDEDCRLLTVGSWYAMSGYGLAFTRNSKYLSMFNKRLLDLRSNGDLERLRRYWMTGTCKPNKQEHKSSDPLALEQFLSAFLLLMAGILLAALLLLLEHVYFRYMRAHLAASTVGPCCALVSLSMGQSLSFHGAVVEAAARGFGAGGRGHCRSAVCAAQVWRARHERDAAMARARQLAATLAAHGLQPPPRRLASAAALLGEAHDATRPRTLHAPADLLPDLDRPLSCGDLRAKERTRVEMETVL

>EposIR1

MWLILLLLATAVDSRLPPGEMVTDYFLHKDAKYVAYLTCGASAEYKAVTSVLMSEGMRAAVGRIDQETIDLDRLLYQWDAAVGVVLDGACRNTQNVLINASESMLFDAAHAWLVLADEADASARFEDLKLSVDADVVVATYFDEDKYQFTDVFNFGRIQGNALETKQLGTWTVDTGLDLPLPRFKYYDRWDFHNLTLRAVSVMRTIPKVLDEKMLSEPVYTDGITKFTKISSMLLNQLKDMHNFRFNYTIVDRWTGTPQRNSTPSLSNTLLWREQDISAASARLFPIWMNWVDPIFPPVTQLESKFYYIIPDSGVGDYENRFLTPMSADVWWCSAAAGVVCALVLVAAAATEGRPEPGSYGIFSVLAAGFQQDYEDGAQSQEDDSTQSRKLALLVVGLTSMLLYNYYTSSVVSWLLSAKPPSLDSLDALIKSDFELIFEDIGYARGWLDNPGFYYFSGYVNKKENELRMKKVTSAKRSVPLMQTVEAGIELVRTGSYAYHTEPYTASQIVSRTFVDEELCALGGLQMIKPAHVYIMAQKRSPYKQFFVWSMMRLLERGHIKATRARIGGEVPPCSGQTPRALSLGQAAPAFLLLVEFMVLALLMLVVEVWWYRYKKRTNKRGQAPLKVAQKSR

>EposIR3

MLRNILLIFLVTKVSGLQQNVKEFALDFFKTRDVQFICLLACGEKTWEQKFVNNASKLSIAVSSVTIDDSSDYSDALRVCLTQKYTAVGVLIDTKCPVFEDVLLFASENSFFNGNHKWLIVDNDVWMSIVTSDSDGNETNGNLFWLNSMFQKLNLSVDADVSLSLQKGSENNIYEVYSYGTIRGGSTIVSKMGSWKNKSALLPQLNGYKYYRRWDFNQSSMNLVAVMSTPPEVFDLDMLIGDQPKVGVAIITTTSLKVLEELKQLHNIRYNYTIVDRWIGDFDRNSSRVAANSLYFKEQDITPVLRVTREIFQKVDMLLPPLTAIETRYYYRIPTTGPGKFENQFLRPLTPGAWGCVCAVILLCSFMLFLAAKAERRPSAVQYAVFSVMATFCQQFYEDNIGNEDPTRQSSARQLAVLVTGASCVLIFNYYTSSVVSWLLNGPPPSINSLQELLESPLSLIFQDIGYTRSWLQNPTYYFNKKNAEVEDKLRRYKVFNKKAGAPLLVPLNEGIEMVKAGGFAYHTEVYNANTRIAKTFTQSELCELGSLQSMEKSQLYASVPKNSPYKEFFNWNLFRLHEAGIVSRIQRRTSSPEISCGGSSPRALALGGAAPAFMLLAFGFFLSTIILLLERFIHRNNSRWENKFNALAP

>EposIR4 MTEIAGGSSELSGSILRMRLDRIDRLDYVVPVWPFLVGFTYLSERESSSNLFVMPFSRGVWGACAAIALVLTLAQRATAKAPVERDGAYVAVVATWLQQDASAVPEGASGRLTSMMMSLCSMLVYAYYSSAIVSALMAAGGGGPTTLRALADSRYALASEDYDWIRYMMFDEETGWDDLEYLKRKKKTSSFYQSHERGMQLILEGNTAYHAEYNHIYPLMNIFSDDQICKLQYVYTVPPVMTWLTTKKQSQWKEVMHSVGGWLHETGLAKRLVARWQLKQPPCRAALLAERVKYGDVAPLVYLSTAGVIAAVVLLFLEIAVARWNARRSVESNGNDDTIIMEDDVA

>EposIR7dMKVFSALTHSRLRCLVLPISSLHMDHFEPIPLADYFVAIIDSYDDFTRLASRLTRARSWNPKALFIFVFFGITSTDDENIKHAETMIHCLFKLNAINAVVIIPQANNIRRANVYGWKPYDPPEYCGYSNESARRRLFVENVCDRGVVKYAKTVFEEKIPSDMKGCTFKMLALERQPFISTDPDDPNIEQLLINDVAKRYNIKLEYNILNVFRGEKQINGHWDGALNDLILKKGELLLGGIFPDNEVHEDFECSSTYLADSYTWVVPRAFQQPLWLAFFIIFKKIVWFSVVAVFIVAALTWKLLAKLSGDPTYRKNLDHYFINTWISNLGFAAFSRPVTHSLRLFFIFLNLYCVLLLTAYQTKLIDVLTNPNFEHQMSTVEELAASDLECGGSEELRDLFENSTDPMDKYFESKWQNILDIREAMIDVALHRNFSLLCSRLELAHIAAVVPELSDKYGNTKFYVFEHNVFTVPLEMVSLRGSPFLKKFSKTLESFRQYGVNDKVRKYFAGYTLRKKAVLQTDLETENSSRDALSVITLQGGFMALLFGYVFGSIFLVVELIMNTKLVKNIKIFKKSVYTLI

>EposIR8a

MDFCCIFWFIFVINLACVVSELSLRFVFIVEVHEQDLPPLVGRALKIAEDSQPETRLSDSIVLLDRENEDESYRQLCSAVSEGVSTIIDLSWSPWDSADQLASDAGVPLVRTLLGSQQLLRALDEHLESRNATDAALLLESEGDVDRTLYELLGESNIRVWVHAGLTRDSARALKAMRPEPSFYAIIGGSAFVADTYKRAVKEKLVRRDYRWNLVQTDYSPPAVTAAAPAMALHVDPGECCRILAMKDGCSCSQDFERKLPILSALVQLLAETYSKLEDESALTARVDCEGVAPELNDTRAKLYRQLAEDSGASNESVFYWDGNRYGLFLRSRFILSTLKPDAGLQTVATWTADEEYKLLPGVTLEPLRQFFRIGTAPAVPWTMPKLDPATGEPMFNEDGQPLYEGYCIDLIQKLSESMDFDYEIVTPKVGTFGRRLPNGTWDGVVGDLMRAETDMAVSALTMTAEREEVIDFVAPYFEQSGILIVIRKPTRKTSLFKFMTVLRTEVWLSIVAALVLTGFMIWLLDKYSPYSAANSPHAYPYPCREFTLKESFWFALTSFTPQGGGEAPKALSGRTLVAAYWLFVVLMLATFTANLAAFLTVERMQTPVSSLEQLARQSRINYTVVEGSTIHQYFINMKFAEDTLYRVWKEITLNATSDQSQYRVWDYPIREQYGHILLAINASIPVPDATTGFSQVDEHTDADFAFIHDSAEIKYEVTLNCNLTEVGDVFAEQPYAIAVQQGSRLQEELSRALLDLQKERLLEQLAAKYWNETARQQCPDADESEGITLESLGGVFIATLFGLGLAMITLAWEVFYYKRKEKNKVQVLEDTEKKPKKAFEKEPHKKISDSVARLRKGRKKPERVTIGDTFKPVSEKEGVSYISVYPKTEFKP

>EposIR21a

MLVTIKIVLLKIIILYAAGQDVEYYPSQSLLNTFDGKILPQQYKFKSKFGVKSKPYLDSHFEYKREAQWRKFYKEDNVEKNNIRKRAVDPAFHGHPKTREELWNERFINKSTAFDQTLSLIGLLRNITLTYLSDCIPVILYDSQVKSKESYLFQNLLKDFPIAFVHGYINEDDEVVEPKIIRATKECINFIAFLSDVTKSAKILGKQADSKVVIVARSSQWAVQEFLAGPQSRMFVNLIVIGQSFKDGEDDSLEAPYILYTHKLYTDGLGASIPVVLNSWSHGKFSRSVNLFPAKMSQGYAGHRFVVAAANQPPFIFRTIKTDLDGGNPRVVWEGVEMKILSLLAERNNFSIEVKEPQDLHLGSGDAVTREVKSGRADIGVAGMYVTGERTRDMDLSYPHSQDCAVFVTLMSTALPRYRAILGPFHWHVWLALTLTYLFGIFPLAFSDKHTLRHLLHNSGEVENMFWYVFGTFTNCFTFVGKNSWSKTTKITTRLLIGWYWLFTIIITSCYTGSIIAFVTLPVFPETIDTIQQLLDGFYRVGTLDRGGWEKWFLNSSDPQTNKLLKKLQLVPDVASGIRNTTKAFFLLPFAYLGSQAELEYIIQSNFTRNKKNKRAMLHISNECFVPFGVSLSFPNNSIYSAKLSGDIARMVQSGLINKIMDEVRWEMQRGAIGQRLAVSPGSINIVSVEEKGLTLADTQGMFLLLAAGFILGASALISEWMGGCTRKCRLTKKEETPSSANSREHLIPTPKSDMDAEIKIISNSGDSRFHLNPRGSSADSRDSLEGTIINLTTENISVHDQFSVNGWDSRRSSSVDIDREVKEIFEKDESRRRAKSTAGVELTDSQRQATASKGAFGAHLSEP

>EposIR25a

MSSLTILLLFLFVPVSLSQTTQNINVLLINEENNALAEKSFEVAKEYVRRNPTLGLAVDPVIVVGNRTDAKAFLENVCRKYNDMLSAKKTPHVVLDFTMTGVGSETIKSFTAALALPTISGSFGQAGDLRQWRNLNANQSKFLLQVMPPADILPESIRAIVTKQDITNAAIIFDEFFVMDHKYKSLLQNIPTRHVITPIKSFNKDEIKTQLRSLRELDIVNFFVIGSLRTIKNVLDAADENQYFGRKTAWFALSLDKGDISCGCKDATIVYMRPTPDAKSRDRLGKIKTTYSMNGEPEITSAFYFDLSLRTFLTVKSLLDSGKWPNDMRYITCDDYDGKNTPNRTLDLKSAFQEIKETPTYAPFFIPEDDPMNGRSYMEFSTDLTAVTVKDGASIGSRSLGSWKAGLSNPLSLTDPENMSDYSAQLVFRVVTIEQNPFIIRDDDAPKGFKGYCIDLIEEIRQIVKFDYEITLAPDGNFGVMDDNGNWNGIIKELIEKRADIGLTSLSVMAERENVVDFTVPYYDLVGITILMKLPRTPTSLFKFLTVLENDVWLSILAAYFFTSFLMWVFDKWSPYSYQNNREKYKDDEEKREFNLKECLWFCMTSLTPQGGGEAPKNLSGRLLAATWWLFGFIIIASYTANLAAFLTVSRLDTPIESLDDLSKQYKIQYAPLNGSAAMTYFERMAHIEVRFYEIWKEMSLNDSLSDVERAKLAVWDYPVSDKYSKMWQAMKEAGLPNSVEEAVQRVRDSKSSSEGFAWLGDATDVRYHVLTSCDLQMVGDEFSRKPYAIAVQQGSPLKDQFNNAILQLLNKRKLEKLKENWWNNNPEAMKCEKQDDQSDGISIQNIGGVFIVIFMGIGLACITLGVEYWWYKWRKRPLVGDVTQVEPSKSTRNNADHGTTKIGEGFTFRSRNLGLSNLRSKF

>EposIR41a

MLEPPIILHPVEVLLQILINKYLLSSFCITMVTETELIIRPPSNLSFMYIYPEYNLTDQILDASEKGCSDFIIQMNEPENFMDAFETVNHVGDIRRSDKKLIFLPRQDDNFNASVLMDILGLKASGFVANILLVLPSINCTDDCNYYDLVTHNFVGTDDEVDQPLYLDRWHFSQEQVDNGVNWFPHDMSNMNGKTLKVAAFTYKPYVLLDLDPSDNALGRDGIDVRIIDEFCRWVNCTVELVRDDENEWGDIYDNLTGVGILGNVVEDRADMGITALYSWYGEYRVMDFSAAFVRTAITCIAPAARVLSSWDLPFLPFARLMWVCLIFTFFYASFALFIAQRSTDKIFMSTFRMMITQARDDTADSWRIRSIAGWMLVTGLIIDNAYGGGLASSFTVPKYEASIDTVQDIVDRKMEWGATHDAWLFSISLSEEPLVKELISQFKTYPEDELKRKSFTRSMAFSVEHLPAGSFAIGEYITKEAAEDLELMLENFYYEQCVVMLRKSSPYTAKLSELVGRLHQSGLLLAWESQVALKYLDFKVQLEVKLSRSRRDVDEVKPLNLKQLVGIFIIYFGGLSICLICFLVELLTRCGKASIVI

>EposIR68

MWITVLLVLLITGAIFYCLARFYTNLLQYQSDHSHVVVTAKDQDEKPVGMYLFGDIINSILYTYGMLLVVSLPKLPTGWAIRLLTGWYWLYCILLVVSYRASMTAILANPAPRVTIDTLQELVDSKVTCGGWGTETKHFFEESIDDIGQKIGERFEMIDDPNEAATKVAQGVYAYYENEYFLKYLSVKRKNSDDKMSIETQNNSTNATVQVKLDSERNLHIMTDCVVNIPISLGFHKNSPLKPLADIYMRRTVEVGLVEKWMNDVMYPIRALDTTDNEIKALMNLKKLYGAFIALAIGYFLSLVCLLGEVIYWNCIVKRDPRFDKYAMDLYYEKKK

>EposIR75q.1

MKVVLIASVVLLCLSFVSMNDAKILKMVVNVIQSFNKPCHVVANLCWGSFEKKQLMDGLGSVDNPKTVKFVQNMDLDHFVLREKTVFLVDLSCPSVTSFFNKANSSRIFNKPYRWLIVGSSVDEGFPSELDNLQILPDSEVYLSQSNGNDSFIINLIYKIKLGRDWISEYYGTWTNNGVIKSRISETSVAMRRKNLAQETIVTSMVVTDDSTITDLFELRYPLIDSVSKSLTQQLMPLYYFMNATARVIHSDTWGYFVNGSWSGMLGDMVEGRAELAGTLLFITEQRIEVQEFLTYPSSFPVRFIFHEPPLSYQNNLYLLPFNTSVWYCCGSFVGVMIVVLLINAQWETKKLKKESANTVLFPSVSEVTVFVVSAITQQGSTVELKGSLGRVVIFILFLAFLFLYTAYSASIVVLLQSSSNQIRTLSDLLQSKLEIGAEDTPYNRFWFAAAKDPVRKAIYEKKLAPKGSIPKFYGMVEGIKMMQNKPFAFHANLGVGYQIIQQYFQEHEKCGFQEITFLQDSTPWSSCYKLSPYKEIFKIGQIRIQEHGIANRINHLIFAKRPVCSVRGGRFVSVSIVDCYPSMLVLLYGLVLAVMILFVEILYDKRCSARKRVSASIVSSSRGTVSSSSGLPSVSIDF

>EposIR75b

MKLLFLLNFALCLSSIFSLDSDDISMIVSFSQQDGRSTSVLAPYVCWSSYEVATLAKSLHEVGVNAARSLQPRRTENYLQNLLILADLGCAGTDEFLIKANDEGFFKSPYRWLLITKDPEDLGILDRIVMLIDSDVVLAQKTSDGYKLIEVYKVIANSEVIFTTRAKWYPNGGTLKNTTTDGNNTALTMDDKITSITVVEDKYGVLEDHRPSKILSTRRMDIRKHTLTMVNVITDSNETRKHMDDRLQLHQDSITKMSYMVVKICFEMMNSTEKLIFTNTWGYVDKNGSWNGIIERLIKKEGDIGTLTIFTQERMKNIDYIAMVGTTAVRFVFREPPLAYVSNIFALPFTGAVWLAILVCVLACALFLYITSKWEATMGMHPLQLDGSWADVLILIIGAVLQQGCTLEPRHAAGRVVTLLLFVALTILYAAYSANIVVLLRAPSSSVRSLQDILNSPLKLGASDFAYNRYFFKKLNEPLRKTIYNKKIAPKGKKANFYTMKEGVEKIRRGLFAFHMELNPGYRLIQETYQEDEKCDLVEIDYINEIDPWVPGQKRSPYKDLFKINFIKIRESGIQACIHQRLHVGKPRCLGAVNTFSSVGITDMYPAMLATLYGMLLAPAVLLLEIAYKRLMEMREKRKAILQSDHDAT

>EposIR75q.2

MKTYCLMVSLIFFSGGCYAENETKLFMVSDVIQSMQRPSAVIAMLCWSSRMKMQLYSALGENDTEIKMMQFFKAGKVPQLDAQAQHVVFLADLDCPGISSYLTMSYLEKHFRSPFRWLLIGTGSKNTGNEISIPEALASVDLLVDSEVILAQHLEHGSYELHLIYKVGPNTEWKKELYGTWDKNKRLQTSLMEGELILRRLNLENYEIPISYVLTDNDSINHLFDNVNDHIDTITKVNFPTTNHLLDFLNASRKYVFTNTWGYRVNGTWNGMTGYLVREEVEVGGSPMFFTSERVSIVDYISSPTPTRSKFVFRQPKLSYENNLFLLSFRAAVWYCTIALLNLLVLAVFIAAVWEWKKTHGHENEKSETDASILRPSLMDVTLLILGAACQQGSTVELKGSLGRVVMLVLFLALMFLYTSYSANIVALLQSSSSQIKTLDDLLHSRIKFGVHETVFNKYYFSTATEPVRKAIYETKVAPRGSKPRFMSMEEGVKKMQKGLFAFHMETGVGYKFVGKYFQESEKCGLKEIQYLQVIDPWLAVRKNTPFKEMFKIGTKRIQEHGLQSRETRLLYEKRPKCSGQGGSFVSVSMVDCYPALLVLSYGSIVAIVLLFMEIMSAKKSFIYGKICRTTETED

>EposIR75e

MFLFVLMCICLANTRASHLNGDFVKDFLRREERPATIVSHLQLTKREQVHLSKTFFANNKQFQIALETPITNNEHVVHLADCRNNYTWEILQNAINDKSIRSPARWLLLLEDDLGNDLDSVQINDYKSDIERRLLDSYVFMDTEVYVACQRENHVELFSVYKTKPHLPLIWEEHGVWSQAGFSKPRPAPLAMRRRSLGGAPVVAATVILDNRTLEHMPDYLHREVDTLTKLMYYMTMHLVEWVNGTRVINRTGSWGYRQPDGRYDGVVREMQDGRADLSGTVMIPTIERSKYMNFVLPPAPVEAKFIFKKPSLASVTNIYVLPFSIGVWLSILVLIFVSSLTLFLSYYREKVVNPRYNMSWPQKVSEGLFETLCLVFQQGTAGDPISIAGRQILLLGLMAFMFLYTAYSANVVALLQSPTNDISSAETLLTSPLACGAQDVLYNRNMFLHETRPIHRALSSRKILPQGEKAFLSVDEGIRKVREGMYAFHVELTAGYDQIQKTFLEDEKCNLGAIKFMSITYPYLALAEGSHIKEQLRIGANRIMEGGVQRRTARRMLPAAPRCGAGAATFAAVRLRDVAPALRALLALYALALPVCALEVLARRRELRNAEKKADEEDTPEDISENFIS

>EposIR75d

MELASFITVYFAAKQLSLLTAFICWKPEELLVLQRETARAGLRLRTPRGAXALAPDGDRREAMLLDLXCPGAQLILDQASASRAFNLRHSWLLLDXGPFNASXVQXTLAXTLVLPDADVALAADDALVDVYRXRADQPLALAPLGAGPAEWAAXPAAPTRRKDLNNVYLKAATIISQPQHFKGWADLTMRHIDTFPKLTYPLMLLLAEDLHFRYNLKQVDLYGEERXGAFDGLAGQLQRAEIEVGITSMFMRXDRTRVLHYCSETVELRGAFIFRQPSKSSVSNVFLLPFSRGVWAATAAVLAAATALLAALARQARLRAVDPMLEQLSLGETVIFTVGTVCQQGFNLVPAIASXRVVMFCALMTSLFAFTSYSAKIVAILQTPSDAIRSIDDLTRSPMTLGVQETTYKRVYFAESTLPATQQLYRXKLLPLGERAYLSVVDGVERLRTGLFAFQVEEPSGYDIISKTFTEREKCGLQQIQAFKLPMVAVPVRRHSGYKELFATRLRWQREVGLMERSRXMWLAARPRCDAGAGGFVSVGLIDIISALHVLAAGMALSLVMLAAERAAQRCGPPRLRARRXEELVT

>EposIR75p MSGRIIQWVMFASLMALYAAYAANIVVLLQAPSNSINNLAQLAKSKMTLAGYDADYNNFLFKGTTDPVRRAIAKRVDPERGPKPFYSLEEGVERIRKGLFAFHVVSDPTYRQVEKTFFESEKCDLVEIDYAGFKKFYMPAYKHSPYLELLRVVFKHIREVGIKSAINFRLETGKPSCKNAAAMFVSVGVMEMRSVMLFMVYGVGISXAILLVEILVFHLDRYRQNRSAVRVGQVNQVCVGITWQESPKLGGKGAIFGRNRRSMDTEIQPCSTLVEG

>EposIR76b

MAGMELIISSFCNATFCDVIYDSPSPLTEHQAEVIATKNELNGKHLKVATYDNYPLSWVATTENSTLNGRGVAFVIFDILREQFNFTFDVITPSQNFEIGGSEPEQSLIGLVNSTKVDMAVAFVPILYKYQSMVDFSSILDEGVWNMMLRRPKESAAGSGLLAPFEVHVWYLILAAVLSYGPCITFLTYVRSKMVKDDESHIPLSPSFWFVYGAFIKQGTTLAPEANTTRVLFTTWWLFIILLSAFYTANLTAFLTLSKFTLDIENPQDILKKNYRWVAPQGSTVQYTVGDVDSDLYYLNKMVANGRAQFPSVNADKQFLPLVAGGAVLVKEQTAIDHLMFADYVRKTKDGVAEADRCTYVVAPHPFMEKLRGFAFPRGSKLKPLFDPVLTYLLQSGIVKFLEHRDLPSTKICPLDLQSKDRKLRNSDLTMTYMIMVTGLAAAIGVFVGEMMIRRYVRVKMKNMRGDTKTKLPKNAAMTRRFKLDDSRPPPYDSLFGNNSRYKMTGKSKRKIINGREYWVVGTVNGESRLIPVRTPSAFLYQRDK

>EposIR87a

MRSRLFLLYLCFIHFAAAKTNPLLMPSEDSGKLVKAAECVVKMSAKYFVEHKALSGSIIIISMNSMVSIAQRAMIDTIHRGIKSTVMVKDSLFPHANASHFREIAKNYMFILESQQELTRDVIQLNKLPTWNPLAKAVVFYQLQPGEDGEQISIDFINLMRGYKLFKSIIFMFSPENEEVISYSWAPYSDTNCGGKCESVYILDKCTNGKVRQVHSQIDMFPLNMKQCPLVTYAVVSEPYVMPPVRKLTDTAYDDAYEFEKGGEIKLVKLISEFTNMSLIVRMSDVTENWGLIDANGTATGAFGVLRNDSVDLVIGDIEVTRTIRKWFHPTISYTQDEMTWCVPKSAQASTWNNLVIIFQWSTWVATFLSIVIMGLIFHYFYYRENNRKVTKWPTNSWLYTLSMLLGWGASFNPKSATFRILIFAWLFFGLIMGISYESFLRTFLMHPRYEKQISTASDLIRSEIPLGGRGIYRSYFETNNESSFYLYRKYVETSFSDGIRRAALGRNFAAVSSRRQAEYQDQKLGKGRPLLYCFKEGNNLYKYGVVLVAKRWYPMLDRLNNMIRRVSENGLIEKWNQELFIHSVSADSSGVVESLKIQHLLGAFMFIGLMYAFSVLVLIGELAMGSLDKRRNNKTKEKAVYRVKLI

>EposIR93a

MRIWLLVFCIAGVRSEEFPSLITANASIAVVLDRQYLGEQYQALLEGLKDFIKELTRVDLKHGGVVVYYYSWSTISLKKGFIAVFSIASCEDTWSLFSRIEEEELLLFALTEVDCPRLPPDSAITVTFTDPGQELPQLLLDLRTNKAFNWKSAIILHDDTLNRDMVSRVVQSLTSQIDDENIPTISLTVFKMKHEINEYLRRKEMHRVLSKLPVKHIGENFIAIVTSDVMSTMAETARDLGMSHTQAQWLYVISDTDNRRANLSMLINALYEGENIAYIYNITEDREDCRNGLMCYAEEMMTAFVSALDAAVQEEVDVAAQVSDEEWEAIRPSKLQRRDMLLKHMMQHITVNSKCGNCSTWRAIAADTWGSTYSGYVDTSDTGDNETTGVIEQINLLQVGYWRPIDGPRFTDYLFPHIAHGFRGKVLPVITYHNPPWTILKANESGSISSYTGLIFDIVNQLAKNKNFTLKLLFPGDIKNALSNKTSAEGTYSQSAMLTMMAVAKRQAAFAAASFTVLPDKNPGINYTVPVSTQPYAFMIARPRELSRAMLFLLPFTTDTWLCLGFAVVLMGPTLYIVHRLSPYYEAMGVTRQGGLATIHNCLWYIYGALLQQGGMYLPRADSGRLVVGTWWLVVLVVVTTYSGNLVAFLTFPKQEMPVTTVAELLDNRALYTWSISKGSYLEMELQNSDEPKYVSLLKGAELTSESSGMEGHLQTRSPLLMRVRSQRHVIIDWKLRLSYLMRAEHLATDTCGFALSTEEFLNEQVAMIVPAGSPYLPVFNKEINRMQKAGLIAKWLSAYLPKRDRCWKTSSIAQEVDNHTVNLSDMQGSFFVLFLGFVTATSVLLIEWFYHRRKSQKEDVAIKPYVE

>DmelIR25a

MILMNPKTSKILWLLGFLSLLSSFSLEIAAQTTQNINVLFINEVDNEPAAKAVEVVLTYLKKNIRYGLSVQLDSIEANKSDAKVLLEAICNKYATSIEKKQTPHLILDTTKSGIASETVKSFTQALGLPTISASYGQQGDLRQWRDLDEAKQKYLLQVMPPADIIPEAIRSIVIHMNITNAAILYDDSFVMDHKYKSLLQNIQTRHVITAIAKDGKREREEQIEKLRNLDINNFFILGTLQSIRMVLESVKPAYFERNFAWHAITQNEGEISSQRDNATIMFMKPMAYTQYRDRLGLLRTTYNLNEEPQLSSAFYFDLALRSFLTIKEMLQSGAWPKDMEYLNCDDFQGGNTPQRNLDLRDYFTKITEPTSYGTFDLVTQSTQPFNGHSFMKFEMDINVLQIRGGSSVNSKSIGKWISGLNSELIVKDEEQMKNLTADTVYRIFTVVQAPFIMRDETAPKGYKGYCIDLINEIAAIVHFDYTIQEVEDGKFGNMDENGQWNGIVKKLMDKQADIGLGSMSVMAEREIVIDFTVPYYDLVGITIMMQRPSSPSSLFKFLTVLETNVWLCILAAYFFTSFLMWIFDRWSPYSYQNNREKYKDDEEKREFNLKECLWFCMTSLTPQGGGEAPKNLSGRLVAATWWLFGFIIIASYTANLAAFLTVSRLDTPVESLDDLAKQYKILYAPLNGSSAMTYFERMSNIEQMFYEIWKDLSLNDSLTAVERSKLAVWDYPVSDKYTKMWQAMQEAKLPATLDEAVARVRNSTAATGFAFLGDATDIRYLQLTNCDLQVVGEEFSRKPYAIAVQQGSHLKDQFNNAILTLLNKRQLEKLKEKWWKNDEALAKCDKPEDQSDGISIQNIGGVFIVIFVGIGMACITLVFEYWWYRYRKNPRIIDVAEANAERSNAADHPGKLVDGVILGHSGEKFEKSKAALRPRFNQYPATFKPRF

>DmelIR10a

MAVLGTVFLLFMLDLKTLNLTRLNGLLVEPTRDLPQLELWLRAGSDHQDAENPYVQWFLLRTEIPLSIVTYQENRYWMDDPFGRRNLVLVMSLDQLLTNRGAAAPIQKASTFFYILADQDKDLSADEQLRLEGSCRQLWTQHKVYNRFFLTRDGVWIYDPFKRRDSAFGRLVRYYGSETLDKLLFRDMAGYPLRIQMFRSVYTRPEFDKETGLLTRVTGVDFLVAQMLRERLNFTMLLQQPEKKYFGERSANGSYNGAIGSIIKDGLDICLTGFFVKDYLVQQYMDFTVAVYDDELCIYVPKASRIPQSILPIFAVGYDIWLGFVLTAFACALIWLTLRVINLKLRIVSLGNQHIVGQALGIMVDTWVVWVRLNLSHLPASYAERMFIGTLCLVSVIFGAIFESSLATVYIHPLYYKDINTMQELDESGLKVVYKYSSMADDLFFSETSPLFASLNKKLSWNRDLRADVIDEVARFRNKAGVSRYTSLILESSHFTLLRKIWVVPECPKYYTISYVMPRDSPWEDAVNALLLRFLNAGLIVKWIQDEKSWVDIKMRSNILEADAESELVRVLTIGDLQLAFYVVIGGNLLAFLGFLAEHFRWKLQKKGV

>DmelIR52c

MVWLIIILFCLGNSSSQILDVTNNSHLDFDYRLFGLLQRLQVEKSYDTLLVYGEDCAIPSLFERLQVPAVLVSSGSTNFDWNFSSLTLILSCNFQDEREENYRTLMKLQTSRRLILLKGHIKPESVCDFYSKKEQHNVAMVKENFYQLEVVYSCRLFQDQNYEKLNLFDGKSIYKDQFRNMHGAPIRTLSDKEPPRTIPYIDSKTGEEKFKGYVGMLISQFVKKVNATMQIREDLIKDDEEVSFVDITNFTSNDILDIGICEARTLEMSNYDAISYPYLMSSYCFMAPLPDSLPFSDVYMAIVAPSILIMFLIIFCICSVLIIYIQERSYRSLTIRSVLMNDICLRGFLAQPFPFPRQYNRKLKLIFMLVCFSSLISTTMYTAYLQAFLWGPPIEPRLTSFDDVKKSRYTMAINIYEREFLEALNVSLEDVEIYDYGKFSKLRSTFNTNYLFPVTALQWFTINEEQKLFKYKIFYYCDAFCLNQFDILSIPLRRHLPYRDIFEEHMLLQKEFGLTKYWIDQSYRDMIRANLTTFKDFSPLLENDYIEVHNLYWVFTMYFVGMGMGLCFFILEILRPLRYWRNCKIKCEYCYAFLKNFAK

>DmelIR56a

MGSRFFIRNLILFGLLASSNMQIPFGELEKKFELDVDFLLGVTELVGHIQGLYSITVYADCIDIHPSIQQRIMDKFMVPVNTIGSNLSRPNYHKLDNSRIRIVLFTGLNDTILVNLNKTDVPYSDNFYMLAYASAIKNKCIELDFIEEVFTLLWKMSIQNAILLIRGEFMMEMWSYLYMGKIHKIKLTKPNSYLESLRKYNYRFSLEVINDPPAIFWYNSSEQADVTGGGNLSVSGPLGLIIINFLRHLNVTIDIVPIPGKQTSQYELFQQPDNLRAENGVNMVGSALLKYSPMVTQSRMCLLVSNRRMIPFSRFLDRLVSPGVHKLTFVSSIGIFVIKYFSHRPRSFVDAIFCTIRFFFAIPLPSIILNRLPVVDRFIEVFIIIFVQILLSSNISITTSALTTGFWEPPIINVETMRASGLHILTEDPTILQAFKENILPSSLADLVILVDEDTYFHHVTTLNNSYVYVVQAHNWQIFRLYQQQMTNEPFEIASEELCSKWRILGIPLNPKSPLRFMFKDYFYRILESGLREQWVHSGFKKFCEFNNLKKLPVDSVDSWQPLSIEFYSNVIRAYIIGLVIATLAFVAELLHNGYRRKNVKKT

>DmelIR11a

MRFAILWLFSGCLLPGIQVGIWVVVRAQPTGRDVLLSRLGNQQNELNTRRLANASSYLTRNYIANRINTLVVREICVECPYELSERQRQLVDQILASLAPELSVLLHKGTAEETTWEYTLFVVNDHTAFTGQVFIFPDELLEREFFCIVVVSEIQSRQFVRQTVGSIVKSNLQMHFVNVVVVAQLEDGTVGTYSYKLFKANCTPGITVRQINHFDRITGKPQQSMPDLYPVRNGHLGDCPFNVGAAHMPPHLIYKRHKDPPPASNVSIPAEDLAGIDWDLLQLLAKALKFRIQLYMPQEPSQIFGEGNVSGCFRQLADGTVSIAIGGLSGSDKRRSLFSKSTVYHQSNFVMVVRRDRYLGRLGPLILPFRGKLWGVIIVILLLAVLSTCWLRSRLGLSHPIEDLLTVIVGNPIPDHRLPGKGFLRYLLASWMLLTLVLRCAYQARLFDVLRLSRHRPLPKDLSGLIKDNYTMVANGYHDFYPLELTCRQPLDFSARFERVQRAAPDERLTTIALISNLAYWNHKHPNISRLTFVRQPIYMYHLVIYFPRRFFLRPAIDRKIKQLLSAGVMAHIERRYMQYENKRKVASNDPVLLRRITKSIMNGAYRIHGLVIVLATGMFILELLAGRSNGRLRRWMEWVHQ

>DmelIR7b

MKYWLYILSCCSLVASTMESSSDWDLAEALAQVVANSEMGRFKTLYIYTHTNSQSTGGHLEELLDQVLMIVPNNLQARRLLLQQSMEYKPYVHAVLALVDGLPSLSAIYARIRATQDLSHTLIYMSMPTDAYGEEMQATLRFLWRLSVLNVGVVLRPPGDHILMVSYFPFSALHGCQVISANVVNRYQVGTKRWASQDYFPSKLGNFYGCLLTCATWEDMPYLVWRPDGSGSFVGIEGALLQFMAENLNFTVGLYWMNKEEVLATFDESGRIFDEIFGHHADFSLGGFHFKPSAGSEIPYSQSTYYFMSHIMLVTNLQSAYSAYEKLSFPFTPLLWRAIGLVLILACLLLMLLVRWRHHHELPRNPYYELLVLTMGGNLEDRWVPQRFPSRLVLLTWLFATLVLRSGYQSGMYQLLRQDTQRNPPQTISEVLAQHFTIQLAEVNEARILASLPELRPEQLVYLEGSELQSFPALAQQSGSSARVAILTPYEYFGYFRKVHPMSRRLHLVRERIYTQQLAFYVRRHSHLVGVLNKQIQHAHTHGFLEHWTRQYVSAVDEKDESVARIASTSYSTLDGIDGDPSLSESEEDQQVAPVRQNVLSMRELAALFWLILWANLGAVVVFVLELLLPRIKLRKILRKMKSDIKKQISKLVRK

>DmelIR21a

MSYYWVALVLFTAQAFSIEGDRSASYQEKCISRRLINHYQLNKEIFGVGMCDGNNENEFRQKRRIVPTFQGNPRPRGELLASKFHVNSYNFEQTNSLVGLVNKIAQEYLNKCPPVIYYDSFVEKSDGLILENLFKTIPITFYHGEINADYEAKNKRFTSHIDCNCKSYILFLSDPLMTRKILGPQTESRVVLVSRSTQWRLRDFLSSELSSNIVNLLVIGESLMADPMRERPYVLYTHKLYADGLGSNTPVVLTSWIKGALSRPHINLFPSKFQFGFAGHRFQISAANQPPFIFRIRTLDSSGMGQLRWDGVEFRLLTMISKRLNFSIDITETPTRSNTRGVVDTIQEQIIERTVDIGMSGIYITQERLMDSAMSVGHSPDCAAFITLASKALPKYRAIMGPFQWPVWVALICVYLGGIFPIVFTDRLTLSHLMGNWGEVENMFWYVFGMFTNAFSFTGKYSWSNTRKNSTRLLIGAYWLFTIIITSCYTGSIIAFVTLPAFPDTVDSVLDLLGLFFRVGTLNNGGWETWFQNSTHIPTSRLYKKMEFVGSVDEGIGNVTQSFFWNYAFLGSKAQLEYLVQSNFSDENISRRSALHLSEECFALFQIGFLFPRESVYKIKIDSMILLAQQSGLIAKINNEVSWVMQRSSSGRLLQASSSNSLREIIQEERQLTTADTEGMFLLMALGYFLGATALVSEIVGGITNKCRQIIKRSRKSAASSWSSASSGSMLRTNAEQLSHDKRKANRREAAEVAQKMSFGMRELNLTRATLREIYGSYGAPETDHGQLDIVHTEFPNSSAKLNNIEDEESREALESLQRLDEFMDQMDNDGNPSSHTFRIDN

>DmelIR60a

MWCNNPGLIIIIFLGQILNLCQGIVNLSNETANTVIFMLPEKDLGPDVWKAGVGCLDSFAQIFFFRNPKERFTRAYNLMLVHAFHLSSPADQIQEGFSKLINEAVTNPGPPDREELFQMRVASDYNITNGTEDKGELILADNYVIVVDSVDRLKELMKKKIVEMRSWNPGARFLVLFHNATCRNRPLGVASNIFKDLMEMFYVHRVALLYANSTMNYNLLVNDYYSNVNCRILNVQSVGQCHDGKLYPNNAVVKASMQDYVSGFSPRNCTFFACSSISAPFVEADCILGLEMRILGFMKNRLKFDVNQTCSLESRGEMDGPANWTGLLGKVQNNECDFVFGGYYPDNEVADHFWGSDTYLQDAHTWYIKMADRRPAWQALVGIFEAYTWIGFILILIISWLFWFTLVMILPEPKYYQQLSLTAINALAVTISIAVQERPICETTRLFFMALTLYGLNVVATYTSKMIATFQDPGYLHQLDELTEVVAAGIPFGGHEESRDWFENDDDMWIFNGYNISPEFIPQSKNLEAVKWGQRCILSNRMYTMQSPLADVIYAFPNNVFSSPVQMIMKAGFPFLFEMNSIIRLMRDVGIFQKIDADFRYNNTYLNRINKMRPQFPETAIVLTTEHLKGPFFILVVGSCWAALTFIGELIIHRWRTQLVSTSEQQDRRSDKRRRRRRRRKPEKDNRWQRQVQVAPVVRFTPVKRRKVFQGQTSQK

>DmelIR68b

MKFLVGLLLQWYLPGIYALAEIACRIAVEQNVQVTYLYRCASCPASFDADYSALELDLYRCVGSRLPVITRNMEAHELEPFRRTDSLSIFQIPAAEKGDSLVRRILDMLNPHQRRKHMHKYLFVWPNAGRHQLLRLFRGSWAKKLLYGLAITGRENGTFDFDPFAWGGLQVIQRLDGEVPYARKVKDLRGYPLRFSMFTDPLMAMPRSPVETAGYQAVDGVAARVVGEMLNASVTYVFPEDNESYGRCLPNGNYTGVVSDIVGGHTHFAPNSRFVLDCIWPAVEVLYPYTRRNLHLVVPASAIQPEYLIFVRVFRRTVWYLLLVTLLVVVLVFWVMQRLQRRIPRRGVIQFQATWYEILEMFGKTHVGEPAGRLSSFSSMRTFLMGWILFSYVLSTIYFAKLESGFVRPSYEEQVDRVDDLVHLDVHIYAVTTMYDAVRSALTEHQYGLLENRSRQLPLGIATSYYQPVVRRRDRRAAFIMRDFHARDFLAITYDSQAERPAYHIAREYLRSMICTYILPRGSPFLHRLESLYSGFLEHGFFEHWRQMDLITRVGASPDAEEFLEDLGDQTDTDSGSNELAIRNKKVVLTLDILQGAFYLWSVGIGISCLGFAVEHAHWFWRRQTLRNAVEARTS

>DmelIR56d

MDNRAAELILRERNIFPTNGSDNITLLNNMFVLEMFYRITQLYHFKNFIFYISERLDLNNKDSQEFFHNFWTYFPMAPNLIITREHHLGIPMMQFISTPSLVMVFTTGKDDPIMELASHNQQGIHWLKTIFVLFPSLQSRDFETNPESLAQFTAEIKDVYDWVWRKQFINTFLITIKDNVFILDPYPTPSIVNKTGVWQAEEFFHKYAKNMKGYLVRTPILYDMPRVFKSDRPTNRYEKNFIHGTSGNLFLGFLEFVNATLMDTSANVTADYLNMTNLLDLVSQGVYETLIHSFTEITTKFVVSYSYPIGINDCCIMVPYRNQSPADQYMHEALQENVWVLISLFTLYITVAIYLCSPLRPRDLSAAFLQSICTLTYSVPTFIIRTPTLRMRYLYILLAIWGIVTSNLYISRMTSYFTTAPPVRQINTVQDVVEANLRIKMLAIEYERMAKSPLQYPESYLNQVDLVDKHMLDLHRDPFNTSFGYTVSSDRWRFLNLQQLHLRKPIFRLTEICEGPFYHVFPLHKDSHMRSVMTEYIMIAQQAGLMNHWERETFWEAVHLHRIHVHLFDDEPMALSLDFFSSLLRTWTLGLILAGLAFAAEMKWHEHVTFKRRPVIRITRKPRSFLRRFMKL

>DmelIR51b

MCKVLTLLVVILLLALTNAAYNVTLLKSVLSLISTREPWINTPIFVGHNTQGGDLNDLIIWLHQTMGVTSLTMNLFLQPEHIRPLGHFKITRYNGIALFFCHDKHDIMWLTLDRNLRKLRRIRLIIILRNQRSGSQGAIKSIFNALWQYQFLNVLVLQRDQLYSYTPYPAMRFFKLDIHTEPLFPHAARNFHGYVVSTPAENDIPRVFHVHDPLTKSRKVLGYAYRTFVEYLDHYNASLRLTNPDENLDPTTSVNMNHIVQLIIDGQLEISLHPYVFTPPTATKSYPLLIYPNCLIVPMRNEIPRHMYLLRPFQLYSWYILLFAVFYITGILYCISPKLNKSSWPQRLGLNFLDAISKILFISPPITIYRPTWRHLIIFLQLSVLGFMSTSWYNIELDSFFTTIVVGEQVNSMDQLVHQQQRVLVKEYEINTFLRHVEPRLVEKVSRLLVPVNASEQVSALLSFNRSFAYPFTEERWQFFAMQQQYAFKPIFRFSSACLGSPHIGYPMRVDSHLETSLNHFILKIQDTGLLNHWVVSDFNDAMRAGYVRFVDNVLGYQSIDVDTLRLGWCVLGIGWILSALVFSCEYWHLYPWRFIA

>DmelIR48b

MILQQSSNLLKLLLLLAISSVRTQGLNDIIIELNQRLLISNNFLYCNQSDKLNEYEIKYLQHMPPISLMIFTSIESMNFTQVEYNLGADNKLFLIMGNEEPPYDFLHALNLHFQFAEYIIVIDEPVDLKKSTKWLDFVNHLWQQGYVQLLIYTSYDEKLYHKIIFPETVIEETLVEQYISIRGSFNNLYGYPVRVAAYNNAPRSMLYVNRWGKHIFAGFYMRFLRAFIDARNGSFVPVLTPSNSPGNCTLNLVNETVDVCADALAANPAAFSLTHGFRIASANVLVTHAKPLHSYRYLTAPFQWSVWACLVIYVLLVVNFLSFIGWLRSGKWEFSKYLLEVFSSLLFSGFYLKEIRGRERYILFGVLFIAGFVYSTEYLGLLKSMLISEVFEKQIDTFEALVESNITLMVDPYDKILFAKYNMPEILSPIMELVSFETLLKHRNRFDQDYAYILFSDRMALYDYAQQFLKHPKLLRIPIDFSFLYTGIPMRKRWFLKHHLGRAWYWAFESGLTRKLALDADFEAVRVGYLSFLITEHVEAQPLNVDYFVMPAIALAIGYILALLSFVIEMTAWRIREFLGCRKATMTSTGCSEGGHVDVD

>DmelIR7a

MFHHLWLLMGLRSLAMGALHPPQPEAMTPLVAAALEILAEQVSPSQSTLAVMDLTQDAEHRDERQEQLMTIILRSVGSEMALRTFQKPPAEVPASFVVFLVNSAQAFNTLGFHFTDIHSTREFNFLILLTHRMSSRAERLQVLRDISRTCVRFHTSNVILLTEKRDGVVLVYAYRLLNMDCDLSVNLELIDIYKNGLFRHGHEARSFNRVLSLSGCPLQVSWYPLPPFVSFIGNSSDPEERAQIWRLTGIDGELIKLLASIFDFRILLEEPCNKCLSPDIKDDCSGCFDQVIISNSSILIGAMSGSHQHRSHFSFTSSYHQSSLVFIMHMSSQFGAVAQLAVPFTVIVWLALVVSSLLLVLVLWMRNRLVCGRSDLASHALQVLTTLMGNPLEARSLPRSSRLRILYAGWLLLVLVLRVVYQGKLFDSFRLPYHKPLPTEISELIRSNYTLINQEYLDYYPRELTVLTRNGSKDRFDYIQGLGKEGKFTTTSLIATMEYYNMMHWSTSRLTHIKEHIFLYQMVIYLRRHSLLKFAFDRKIKQLLSAGIIGYFVREFDACQYRKPFEEDYEVTPIPLDSFCGLYYISLIWLSAAVVAFILELLSQRIVWLRRIFE

>DmelIR64a

MHWWLLVFLPLSCQGLPEHELLELELDYGLAEPQRTSLLQSSLILQFSQDYKHIPRITYFTCQKPHLQTPNQIPNAAEHRDAFAAKNFQLIKSLYESELFVRIVLLDVLAQSPSSGRPNRPGSGPTGGFSQTPSQAQSNSEWLEGVLRMEALRQIAVVDLACGAVSRRFLELASAKMLYSEKFHWLLIEDFAWHGRTQTAEGSGKRDDGEMEEEEPPGQQIQATDDEDLPSIESFLGGMNLYMNTELTLAKRMSEAAHYTLFDVWNPGLNYGGHVNLTEIGSFTPTEGIQLHTWFRTTSTVRRRMDMQHARVRCMVVVTNKNMTGTLMYYLTHTVSGHIDTMNRFNFNLLMAVRDMFNWTFVLSRTTSWGYVKNGRFDGMIGALIRNETDIGGAPIFYWLERHKWIDVAGRSWLSRPCFIFRHPRSTQKDRIVFLQPFTNDVWILIVGCGVLTVFILWFLTTIEWKLVPHDGSALIKPKGGAPPRHHYQQQQQQEQVEAPVRPITAVSVVVSKEKVEEKQEEYEDSTPIDAGTLWQRCYQKLNKYIKDRKAKQKKAPERVGLFLESVLFFVGIICQQGLGFSTSFVSGRCIVITSLLFSFCIYQFYSASIVGTLLMEKPKTIKTLSDLVHSSLKVGMEDILYNRDYFLHTKDPVSMELYAKKITSVPTTKENEADEDEPVDPNPASTDPAKSYRDIVHSHETGAHAKDNAASNWLDPETGLLRVKHERFAFHVDVAAAYKIIAETFSEQDICDLTEVSMFPPQKTVSIMQKNSPMRKVISYGLRRVTETGILTYHFNVWHSRKPPCVKKIETSDLHVDMDTVSSALLILLFSYAITLMILGTEILYSKWHNRIQLKWVGAT

>DmelIR93a

MNPGEMRPSACLLLLAGLQLSILVPTEANDFSSFLSANASLAVVVDHEYMTVHGENILAHFEKILSDVIRENLRNGGINVKYFSWNAVRLKKDFLAAITVTDCENTWNFYKNTQETSILLIAITDSDCPRLPLNRALMVPIVENGDEFPQLILDAKVQQILNWKTAVVFVDQTILEENALLVKSIVHESITNHITPISLILYEINDSLRGQQKRVALRQALSQFAPKKHEEMRQQFLVISAFHEDIIEIAETLNMFHVGNQWMIFVLDMVARDFDAGTVTINLDEGANIAFALNETDPNCQDSLNCTISEISLALVNAISKITVEEESIYGEISDEEWEAIRFTKQEKQAEILEYMKEFLKTNAKCSSCARWRVETAITWGKSQENRKFRSTPQRDAKNRNFEFINIGYWTPVLGFVCQELAFPHIEHHFRNITMDILTVHNPPWQILTKNSNGVIVEHKGIVMEIVKELSRALNFSYYLHEASAWKEEDSLSTSAGGNESDELVGSMTFRIPYRVVEMVQGNQFFIAAVAATVEDPDQKPFNYTQPISVQKYSFITRKPDEVSRIYLFTAPFTVETWFCLMGIILLTAPTLYAINRLAPLKEMRIVGLSTVKSCFWYIFGALLQQGGMYLPTADSGRLVVGFWWIVVIVLVTTYCGNLVAFLTFPKFQPGVDYLNQLEDHKDIVQYGLRNGTFFERYVQSTTREDFKHYLERAKIYGSAQEEDIEAVKRGERINIDWRINLQLIVQRHFEREKECHFALGRESFVDEQIAMIVPAQSAYLHLVNRHIKSMFRMGFIERWHQMNLPSAGKCNGKSAQRQVTNHKVNMDDMQGCFLVLLLGFTLALLIVCGEFWYRRFRASRKRRQFTN

>DmelIR87a

MSTPEQRFWLAALLFLLSQHSEVRGFGINLMKVQTEDKGQEACILALLRKYFDSGDGLSGSVLCINRNYQLPNIEEQLLRGVNNYENYPWSLLITNSREGPSPAKFLMNEKPQCYFLIVDNLEDEDLDEVFEHWKGMVNWNPLAQFVVYLASLEETDEEMNDLMVELLLTFINKKIFNVNVIGQSEENQFYYGKTVFPYHPDNNCGNRVISVELLDACDYPSEETDSEDENDEDEGDGAQEEDDGPQEEGDGEQEEEDGPQEQEDGDQAKGDEGQENDDGGLENKVENEFRIGASDDDELENDLSSNSSEPEAIIEEFFRAKFEDKFPRDLSGCPLTASFRPWEPYIFRNSEEQPVDDYYYGLQGDEDDYNDTSPNYGESDDESYADPGEDGDGAIPDTETQSGGKLKLSGIEYEMVQTIAERLHVSIEMQGENSNLYHLFQQLIDGEIEMIVGGIDEDPSISQFVSSSIPYHQDELTWCVARAKRRHGFFNFVATFNADAGFLIGIFVVTCSLVVWLAQRVSGFQLRNLNGYFPTCLRVLGILLNQAIPAQDFPITLRQLFALSFLMGFFFSNTYQSFLISTLTTPRSSYQIHTLQEIYSNKMTVMGTSEHVRHLNKDGEIFKYIREKFQMCYNLVDCLNDAAQNEHIAVAVSRQHSFYNPRIQRDRLYCFDRRESLYVYLVTMLLPKKYHLLHQINPVIQHIIESGHMQKWARDLDMRRMIHEEITRVREDPFKALTFDQFRGAIAFSGGLLLVASCVFAFELCYVKYVYRTEKRERKTKKITKKVHNIKIQHD

>DmelIR7e

MNISALLNSYYDLSGEQMNHINEFVARAVLHVVHHYILSVTPSLVLTLCCRSNHTCNFYNKMMSTLFREWGLAPLQIVNVLRGVPWHPVPGRRHFNVIFTDSFAAFEEIRMEYYSREYNYNEHYFIFLQARDRLLQGEMRLIFDYCWRYRLIHCSIQVQKSNGDILFYSYYPFGEHGCSDMEPQLINRYNGSMLVEPDLFPRKLRNFFGCPLRCALWDVPPFLTLDEDQEEVLRVNGGYEGRLLLALAEKMNFTIAVRKVHVNMRDEALEMLRRDEVDLTLGGIRQTVARGMVATSSHNYHQTREVFGVLASSYELSSFDILFYPYRLQIWMGILGVVALSALIQLIVGRMLRERMGSRFWLNLELVFVGMPLLECPRSHTARLYCVMLMMYTLIIRTIYQGLLYHLIRTHQLNRWPQTIESLVQKNFTVVLTPIVQEVLDEIPSVQHMRFRLLEANSELDPLYFLEANHQLRQHVTASALDIFIHFNRLSADKVHQRGEQGSGAHFEIVPEDIISMQLTMYLAKHSFLIDQLNEEIMWMRSVGLLSVWSRWELSESYLRNEQSFQVLGTMELYAIFLMVLVGLIVGLLVFILELVSMRSIYLRKLFT

>DmelIR7f

MNTTSDSNAGSSLSSGSGYSIYKSYLENSRIDMQGEDANLYVARALRLVIENVLAQLSTTLVVTISTRHLGTAHWFEYMMNILMDSWRMVAVQLLRIRPDLVVNPVPGRKRVSLLMVDSYQGLLDTNITASNANFDDPDYYFIFLQARDHLIPKELQLILDHCLAHFWLHCNVMIQTAQVEVLVYTYYPYTADACQKAYPIPVNTFDGRKWKASQMFPDKLSQMHGCPLTVLTWHQPPFVELVWDPKHNRSRGSGFEIQLVEHLARRMNFSLELVNIALLRPNAYRLAEGSSEGPIEKLLQRNVNISMGYFRKTARRNQLLTTPMSYYSANLVAVLQLERYRIGSLALLVFPFELSVWMLLLLALLIHLGIHLPSARRGNEEDGGGGLQVVALLLGAALARLPRSWRHRFIAAHWLWASIPLRISYQSLLFHLIRLQLYNTPSFSLDQLLAEGFQGICTANTQRLLLEMPQLARDPDSIQSVDTPFDWDVLNVLTRNRNRKIFAVANQDVTLSFLHSSAHPNAFHVVKQPVNVEYAGMYMPKHSFLYEKMDDDIRRLDASGFIHAWRRASFASVHRKEQVHMTSRRYINHAKLSGIYMVMAGLYLLAGLLFAGEVLLRQRN

>DmelIR7cMLHSAVHNVSLVYALVWAIDNYYGMATSTPLAVVQFPTSRESRRLHNDLIDAALGRSSGTGRIQFLLEDDRVEMTETDTDPPPPSGLTGRPIAIWFLDSLRSYFRLEMYLNQLGSPYKRNGFFLVIYTGLEDQPMESLKIMFRRLLNMYVLNVNVFLQRDGTVHLYTYYPYGPHHCQSSLPVYYTAFQDLAAPANGFGLTKPLFPRKLTNMHGCEMVVATFEHRPYVIIEDDPKTPGGRSIHGIEGLIFRSLAERMNFTIKLVEQKDKNRGEILPDGNFTGILKMMVDGEVNLTFVCFMYSKARSDLMLPSTSYTSFPIVLVVPSGGSISPMGRLTRPFRYIIWSCILVSLIFGFVLICLLKITALPGLRNLVLGRRNRLPFMGMWASLLGGLALYNPQRNFARYILVMWLLQTLILRAAYTGQLYLLLQDVEMRSPIKSLSEVLAKDYEFRILPALRTIFKDSMPTTNFHAVLSLEESLYRLRDEDDPGITVALLQPTVNQFDFRSGPNKRHLTVLPDPLMTAPLTFYMRPHSYFKRRIDRLIMAMMSSGIVARYRKMYMDRIKRVSKRRNLEPKPLSIWRLSGIFVCCAGLYLVALIVFILEILTTNHRRLRRAFNVINRYAA

>DmelIR8a

MELPLLVLLLALRFAGSEVLKITFWIEPVQRAEFDTDIAMVLKELDALRLDVKVDDTTLTLTRSEDGLDMQRFCEILSTVGASAVIDLTYSHWEEGYNLVRSLGIGYVRLERIMRPFLDMFGDFMRQKRANNVAMVFMNARDAVEAMQQMLVGYPFRTLIMDASQTDPGQHFLERIRSLRPAPTYIALFARAAAMNGIFEKVQKADLFQRPLEWHFVFLDTRDRVFKYRRQAELCTRFTLNPRAICRSMPMPDLYCGSGFTMQRAMLLNVLRSLINAAQVSPGYPLAIYQDCNATASSSEVSDPLEKDDYNWLDMVHWSNFLAYAPPLPHIQDQFQSPVPGLTFAVNISAGYYSSEHEAKTDLAAWSSVGEMRLLNETISPARRFFRIGTAESIPWSYLRREEGTGELIRDRSGLPIWEGYCIDFIIRLSQKLNFEFEIVAPEVGHMGELNELGEWDGVVGDLVRGETDFAIAALKMYSEREEVIDFLPPYYEQTGISIAIRKPVRRTSLFKFMTVLRLEVWLSIVAALVGTAIMIWFMDKYSPYSSRNNRQAYPYACREFTLRESFWFALTSFTPQGGGEAPKAISGRMLVAAYWLFVVLMLATFTANLAAFLTVERMQTPVQSLEQLARQSRINYTVVKDSDTHQYFVNMKFAEDTLYRMWKELALNASKDFKKFRIWDYPIKEQYGHILLAINSSQPVADAKEGFANVDAHENADYAFIHDSAEIKYEITRNCNLTEVGEVFAEQPYAVAVQQGSHLGDELSYAILELQKDRFFEELKAKYWNQSNLPNCPLSEDQEGITLESLGGVFIATLFGLVLAMMTLGMEVLYYKKKQNALEITQVRPVNDSSGSGGNSSTAPPTATSTTKQAWHIPVLEAEEKPAKVSPPPSFETATFRGKKLPARITLGDGKFKPRHGLYARRNLGASDSHSGYME

>DmelIR20a

MLASLNRSTGLSAELLDLYGLVVHFLLSGEHTTLVYFNPAGLDCSWGVLWQRNLTAHPQIVWQRNYSYPDLYYQFNAKLLVLACLPMDSRAAIQLEILANSLSHLRTVVRLLIEVAGPDQVTLARQYLSFCLRRSMLHVELYFRDYHHSLILYSFRAFPSFELVMRWISVGQGVKLFLHKLDDLRGHRLRVIPDLSPPNTFFYRDARGDNQVTGYLWDFLATFAGRLNAGLEVVRPSWRAGSASDSSYMLEYSAKGLIDVGLTTTLITKWNLWAIHQYTYPLLVSSWCTMLPVEKPLATPDLFGRIVCPTLAMTLLLIILVTWLVFRQLRCLTRLKNSRPARIVPHLLTLLLLTTCSAQLLSLLIFPPYHVRIASFEDLLRGDQKILGMRNEFYNFDGAFRARYAGVFYLIDDPNELYDLRNHFNTTWAYTMPYIKWLVIKTQQRHFSKPLFRWSKDLCFFDFMPTSVIVAPDSIYWESIKDFTFRIHQAGLMKHWIRKSFYDMIKAGKMSIKDYSDLETLKPLNIGDLEIVWRVCGAAIAVASAIFIMELLYFYINVFFNSL

>DmelIR31aMNLLISMFILILAAGEGEIIPSMEESVVTNFVKSLVKTKQAIVFSCLFKDFKEISLALMRINQFVSVVNLNQSYSLTSILTRENYARTSVMVNARCSGSSELLFEASENRYFNKTYQWFLWGVDLEVQSLFPLNLNYVGPNAQITYVNETADGYAYWDIHSKGRHLKSNLEINLIATLINDTLNIARDIFHLQSIDFRGQFNGLTLRGASVIDKEDIISNEQIESILSRPTKDAGVAAFIKYHYELLGLLRERFNFTVNFRNSRGWAGRLGNTTFRLGLLGIVMRNEADIAASGAFNRINRFAEFDTIHQSWKFETAFLYRYTSDLDTHGKSGNFLSPFSDRVWLFCLLTLGAFSIIWVLFEIIDYKILRIRVNSQKLEHLNQKSSVICIKTTCIERILQTFGACCQQGLDPNPVDRSVRFLVMTLFLFSLVMYNYYTSSVVGGLLSSSDQGPSTVDEITASPLKISFEDIGYYKVLFRESQNRSITRLIEKKLSSSRSLNELPIFSHIEDAVPYLKAGGFAFHCEVVDAYPVISEYFDANEICDLREVSGLMEVEILNWILHKNSQYTEIFKTAMCNAQEKGFVERILRRRQIKKPACQSLYTVYPVSLSGVLPGFVILICKSINKFS

>DmelIR41a

MFIDLSWSLVLSAIVGKYLNESTICIFWNDKFEFQLLHKSDYISFVGINIKSFDDNGGHYIIDTGLKKKELQNKHLFLDELVIKIIISIEVTHCETFVVFDKDIDRFVNAFNKASVYSIWRSLHNKFVFAHIANESPESRNHFFEDQPNILFVVRDHSSASSFDIKTNKFVGRKAENPSQMILVDRYLASEQRFQFGKSLFADKLNNLQGREVIIAGFDYPPYTVIKHNMSTNAQDMGVSGESDFKNVYIDGTETRIVLNFCEQFNCTIQIDSSAANDWGKVYPNMSGDGALGMLINRKADICIGAMYSWYEDYTYLDLSMYLVRSGITCLVPAPLRLTSWYLPLEPFKETLWAAILLCLCAEATGLVLAYKSEQALYVLPGYREGWWTCTSFGVCTTFKLFISQSGNSKAYSLTVRVLLFACFLNDLIITSIYGGGLASILTIPSMDEAADTVTRLRFHRLQWAANSEAWVSAIRASDEALVKDILYNFHIYSDDELLRLAQDQHMRIGFTVERLPFGHFAIGNYLGPQAIDQLVIMKDDIYFQYTVAFVPRLWPLLDKLNTLIYSWHSSGFDKYWEYRVVADNLNLKIQQQVQETMTGTKDIGPVPLGMSNFAGFIIVWILGSAIATLTFLLELSLTYILKQSNLK

>DmelIR47a

MRQIKLLVWLLVVGVVSSTEQLQFLKNFLEAVHKERSISTILLIQRKVHKNDFLHGLYPIFWPIICLDETKRVELVNNFNKDFLALVYMESEADTLLLSALAADLNHIRDARIMIWLQMSPSENFLDRIVFQASKQKFLNLVVIENTLKTRRFYPFPQPKVQVIDKPFEEKEIYPALWRNFMGKNAIAVPDLVPPRSFNSFDPKTGHRRESGSIYNVFKAFTQRYNITMLLKWPLIRNTTQEEIIGKSVRGEIDLPITGQLISFRHPNGSRSQPLLGMTALSIAVPCGPELPMFDRFFLFYGLATPITITGYYVLLNTIEIILGTLSDRIKRHPRRKKILNLVLNLRVFSCILSLPTPQGNRLRSVKGQLTMVMSITGLILSCIVAAQTSTILTMKPQYRHIKNFQELSDSNITVVCNHLNYLTIKQQMDPKFMAKFMQNIWIVNSIEQMKMIFDLNTSYAYQTFSYKKDPFTLLQMHTTRKAFCRTPGLDLVSGLAYTAVLEKNSIYALALQDYTLKAFSAGLVYYWAEESIRDLISTVGRTQFEKLPIVIGYQSLKLQDYNVCWKILLIGGALAFCVFIVEVVVGLINRRI

>DmelIR48c

MSLLRIILIIIFLRIVSSIPDTIISHLSAELQIKIQIYFGLGNDLYDFSRLDGNYQKIIISHNISEEFKTYHDEPVLIIIRLERDLNLNLATLDVLRSYLTDRQYNDILLIDNDEENLNSYVDIRKAYWNAGFSQVLIYNSQQRTWSIKPYPYLQIRPTSLKEYIENRNTRNLMGYPLRVLVTNDPPHCFVDKDELPGSPNRYKGSIVTMLKIFADQLNATFQANPFREFRRYSTADCVQMVSDDEIDACGSIFIRTYTYATSQPVRLNRVVIMAPFGNPIEKFYYFFRPFDLYVWIGTGIIVVYIAVMGSLLHRWHFKEWNVGQYLLLAVQTLLNRELSLPQSSSGSKFMLLLLLFAIGFILSNLYVALLSMMLTTKLYQRPIENLADLKAANVNILLQTHNIRPNSVYGSSEELRERFLLVEESQHLEKRNGLDPSYAYVDSEDRMDFYLYQQKFLRRRRMKKLSNPVGYTWAVQVIKQNWVLEKHYNDHVQRFFETGLQNKLVDDVHELAVKAGFLHFFPTQTQTIEPLRLEDIVMAAMVLGGGHALAVICFLVELFA

>DmelIR52a

MALGWSVIILGFIGQLSAQILNYTQSRDLELLEGSLFRVLSRLNLEEEYNTLLIYGKECVFHSLLRKLEISAVTVPSGSTDYDWSFSTAILILSCGYDAENEENSYTLMKLQRTRRLIYLEDNSEPESVCMRYSLKEQHNIAMVKSDFDQSDTFYSCRLFQTPNYVEGHFFKDQPIYIENFQNMRGATIRTVADSLVPRTILYRDEKSGETKMMGYLGHMINTYAQKLNAKLHFIDTSKLGAKKPSVLDIMNWVNEDIVDIGTALASSLQFKNMDSVWYPYLLTGYCLMVPVPAKMPYNLVYSMIVDPLVLSIIFVMLCLFSVLIIYTQHLSWKNLTLANILLNDKSLRGLLGQSFPFPPNPSKHLKLIIFVLCFASVMITTMYEAYLQSYFTQPPSEPYIRSFRDIGNSSLKMAISRLEVNVLTSLNNSHFREISEDHLLIFDDLSEYLVLRDSFNTSFIFPVSVDRWNGYEEQQKLFAEPAFYLATNLCFNQFMLFSPPLRRYLPHRHLFEDHMMRQHEFGLVTFWKSQSFIEMVRLGLASMEDLSRKRNEEVSLLLDDISWILKLYLGAMFISSFCFILEILRCGERCKRLWRCRW

>DmelIR52d

MVRIIIILLCLGYTKARILDATNTNHTDLEERLLSLLLRLQQEQFFNTLLIYGEDCAFSSLSRRLQVPTILVSSGSTSFEWNYSSLALILTCEFKAEREENYQTLKKLQMNRRLILLNGNIKPDSVCDFYSKKDQYNIAMVNNNFHQVGIIYACRLFQERNYEKVYLSEGNPIYVDQFRNMQGALLKSITFNLIPGSMAYRDPKTGQEKHIGYVANLLNNFVEKVNATLDMQVKLHKAGKKTSFYNITKWASEDLVDIGMSYAAYFEMTNFDTISYPYLMTSTCFMVPLPDMMPNSEIYMGIVDPPVLVVLIAIFCIFSVMLNYIKQRSWRSLSLVNVLLNDICLRGFLAQPFPFPRQSNRKLKLISMLVCFFSVITTTMYTSYLQSFMWGPPIDPKMCSFADLENSRYKLAIRRYDIEMLRPFNVSMDHVVVFDESSQLEYLRDSFDDNYMYPMSALSWSAFKEQQKLFAFPLFYYSEKLCLKPISFFSFPIRRHLPYRDLFEEHMLQQNEFGLSTYWIDRSFSDMVRLKLATMNDFSPPRLEDYIEVSDLSWVFGMYFTGLGISCCCFGLELLGLPSWTRRLRLTNWLRVRN

>DmelIR54a

MWTVITGIVLWAPVLVAGSAVDFIFRAAAEHSLSVIMIRIDYCPYNWAKDIFENQTIPVVVLSDSETFINIRMFSRPLHVACLPGHELQKDLALLENFTSSLMDFPSQKKIVYISNNFSDPTRMDYIFETCYHRRIWNIVGLLASDEHRYFYRYHLYPSFRTEYRSLESSTIFDKDFPNMHGHPLTVMPDQWLPRSVLYVDRRTGKQILAGSVGRFFHVLSWKLNATLQLSKKVTTGRFLNATALKELSESFSVDVPASLTIMERVEQLASTSYPMEVTHVCLMVPVARRIPIKDIYFILSSASNMFLAIVIVSSYGLALNLLRNMTHRDVRLVDFVLNDKALRGILGQSFNLPLSRSFSTRLIFLMLGIVGLNVSSIFGAGLDTLMAHPPRQFQARSFAGLRRTKIPLVTTEEDFPTWMKLRVPMLVVNVSEYNHLRNGRNTSNAYFASRLYWNLFSEQQKRFTRELFIYSTDDCLWSLALLSFQWPQNSLFTEPVSQLILEVNANGLYDFWVGMHYYDMTAAGLSGLEDPSLQLKEREHPTSLRIVDFQWMWQAYGTFMVIAILVFLLEVSWHRITSLFVSLVY

>DmelIR60d

MRLAIYVAFLSSIGNRSGFLSSLLMSLGKELHYKTILLVGGSSTCWSLEPFETGVPILNLRGENNAYPQDTFNSQMLALACLQTESEDAVKLLYRSLKDMRDTPTLLFASSEEHIHDTLFLGCFRENMLNVLALTASSKEFIYSYQAFPTFRVIKRKLVEIHRYFEPQLKDLGGHIVSALPGNIMPRTMCYRNAEGERQLAGYLNTFIRNYVESINGTLRISWGLVPEDDMRHLTISRLSKIQHVDFPLGIIPLYNKTDKQHVYMEISSWFLMLPMETSVPRAHLFVKLGLERLLPIIVVVGAVLGNAHRIEVGLGPSWRCYYLADKVLRGALAQPIVLPRRLSPKLMLIYSLLLLSGFFLSNYYMASLTTWLVHPPASDRILEWDQLRYLHLKVLTIPEEFKYMSLILGTDFMTAYGSIFQLTNSTDFQRRRISMDPSYAYPVTTSLWPFLELSQVRLRRPLFRRSYDMVLQPFQVMSLPLPRNSIFHKSLLRYAALTRETGLYYYWFRRSYYELVALGKISYKEEEGNPYCDLKWNDFRIVWLAFLGGTIISCLALLLEVAHYRWHLGNSSL

>DmelIR60e

MVIKMISFLLVSVLLCLVGASDSESMQVQVLQDLNLALQTELNVFIDFECCATSEILHKLDSPRILLSSNSREARDLRIRGNFTESTLIIVSVMDSDLNPLVASLLPRLLDELHELHIVFLSNEEPGFPKQDLYTYCFKEGFVNVILMSGKGLYSYLPYPSIQPISLSNVSEYFDRARIIRNFQGFPVRILRSTLAPRDFEYSNEQGGLVRAGYLFTAVKELTYRYNATIESVPIPDLPEYDVYLAVAEMLHTKKIDIVCYFKDFSLEVAYTAPLSIIREYFMAPHARPISSYLYYSKPFGWTLWAVVISTVLYGTVMLHLAARGARVEIGKCLLYSLSHILYNCHQKIRVAGWRDVAIHGILTIGGFILTNVYLATLSSILTSGLYDEEYNTLEDLARAPYPSLHDEYYRSQMKAKTFLPERLRRNSLSLNATLLKAYRDGLNQSYIYILYEDRLELILMQQYLLKTPRFNMIRQAVGFTLESYCVSNSLPYLAMTSEFMRRLQEHGISIKMKADTFRELIHQGIYTLMRDDEPPAKAFDLDYYFFAFVLWTVGLISSLLVFFAELVSGHL

>DmelIR67a

MLPILVPVLLLFNETSWINPILTSIYKDRHHETVLLLQHSQHGNASGLERFPWPVFSFNEQMDFYVRGKYNSEMLVLIWQTGNSDWDLDLWQALDRSLLNMRKVRVLLLRKWEKIPTADVAATAEHLLFLHVAVIGQGNRIYRLQPYAPQSWLQVDPIESPIFIKIRNYFGRYIVTLPDQFPPRSIVYRNPKTDEIQMTGYVYKFLLEFIRIYNFTFRWQRPIVQGERMNLILLRNMTLNGTINLAISLCGFETPSELGVFSDVYDMEEWYIMVPRAQEISIADVYVVMVSGNFLIVLIIFYFIFTILDTCFGPLLLKERVDWSNLMLNERMISGIMGQSFNMSARNTISSKVTNATLFLLGLVLSTLYAAHLKTLLTKRPTSQQISNFKQLRDSPVTVFFEEAERFYLKHAWDRPIRYIKDQLNFRETIEYNALRMGLNRSNAFSALTSEWMIVAKRQELFKQPIFTVQPELRVIQTSVLLSLVMQSNSIYEDHINDLIHRVQSAGIVEYWKHQTLREMITMGMISQKDPFPYVAFREFKVGDLFWIWLLWVSFLFMSFVIFLCELLVDCFISKTLIRNKRPH

>DmelIR75a

MQLVQLANFVLDNLVQSRIGFIVLFHCWQSDESLKFAQQFMKPIHPILVYHQFVQMRGVLNWSHLELSYMGHTQPTLAIYVDIKCDQTQDLLEEASREQIYNQHYHWLLVGNQSKLEFYDLFGLFNISIDADVSYVKEQIQDNNDSVAYAVHDVYNNGKIIGGQLNVTGSHEMSCDPFVCRRTRHLSSLQKRSKYGNREQLTDVVLRVATVVTQRPLTLSDDELIRFLSQENDTHIDSLARFGFHLTLILRDLLHCKMKFIFSDSWSKSDVVGGSVGAVVDQTADLTATPSLATEGRLKYLSAIIETGFFRSVCIFRTPHNAGLRGDVFLQPFSPLVWYLFGGVLSLIGVLLWITFYMECKRMQKRWRLDYLPSLLSTFLISFGAACIQSSSLIPRSAGGRLIYFALFLISFIMYNYYTSVVVSSLLSSPVKSKIKTMRQLAESSLTVGLEPLPFTKSYLNYSRLPEIHLFIKRKIESQTQNPELWLPAEQGVLRVRDNPGYVYVFETSSGYAYVERYFTAQEICDLNEVLFRPEQLFYTHLHRNSTYKELFRLRFLRILETGVYRKQRSYWVHMKLHCVAQNFVITVGMEYVAPLLLMLICADILVVVILLVELAWKRFFTRHLTFHP

>DmelIR75c

MTSWPLYRLIVFNLLEINLSNLMVFHCWSIKEAFPLVEMLNQNGIFSQYIDVQNPDNLANVHKEYLDSDLVRLGVFLDLGCDKAELVTNQSSRARLYNQNLHWLLYDEAGNFTKLTQLFEGANLSLNADVTYVSREDEERFILHDVYNKGSHLGGKLNITVDQTLQCNRSHCQVKEYLSELHLRPRLQHRMDLSSVTFRLAALVSVLPINSSEEELLEFLNSDRDSHMDSISRIGNRLIMHTQEILGFKLHYIWCGTWSVQDAFGGAIGMLTNESAELCTTPFVPSWNRLHYLHPMTEQAQFRAVCMFRTPHNAGIKAAVFLEPFMPSVWFAFAGLLIFAGVLLWMIFHLERHWMQRCLDFIPSLLSSCLISFGAACIQGSYLMPKSAGGRLAFIAVMLTSFLMYNYYTSIVVSTLLGSPVRSNIRTIQQLADSSLDVGFDTVPFTKTYLVSSPRPDIRSLYKQKVESKRDPNSVWLSPEEGVIRVRDQPGFVYTSEASFMYHFVEKHYLPREISDLNEIILRPESAVYGMVHLNSTYRQLLTQLQVRMLETGITSKQSRFFSKTKLHTFSNSFVIQVGMEYAAPLFISLLVAYFLALLILILEICWARYAKKKFSTIIPQNQ

>DmelIR75d

MKVQVAHWLPLIFFLLVSGTPRVAGSWRSEYSRQDPDPKTRWGNQLPDMLVAYYRHHGVHSLMLVVCHTDIADFRLWKLWQHFNLNNFYVQVSTESSLRDLQHVDALDEHKDAPPPKSFHANNSTHWETSFLLPALPYKMGILLLEFSSECALNLLRWSAASEHNYFTTNRFWLLLTEDPGDIDLLEDPEIFIPPDSELRVLHYENVGNFSCSLIDLYKVAAWKPLKRTLVGHNIRNSRHVIHALQHFGSAITYRQDLEGIVFNSAIVIAFPDLFTNIEDLSLRHIDTISKVNHRLMLELANRLNMSYNTYQTVNYGWRQPNGSFDGLMGRFQRYELDLAQLAIFMRLDRIALVDFVAETYRVRAGIMFRQPPLSAVANIFAMPFENDVWVSILMLLIITTVVLVLELFFSPHNHDMSYMDTLNFVWGAMCQQGFYVEVRNRSARIIVFTTFVAALFLFTSFSANIVALLQSPSDAIQSLSDLGQSPLEIGVQDTQYNKIYFTESTDPVTKNLYHKKIASKGENIYMRPLLGMEKMRTGLFAYQVELQAGYQIVSDTFSEPEKCGLMELEPFQLPMLAIPTRKNFPYKELIRRQLRWQREVSLVNREERKWIPQKPKCEGGVGGFVSIGITECRYALGIFGCGAAVSFVLFLFEFIFRHFKQVYRIIKGYREVQR

>DmelIR76a

MENLLVESYYFSTVLSFFAQQFFADSHATCIFWHPAFDFRLETVHPMPLIIMDWHRWANRSDQDVYDYKIKEDEFEGKGIPYNDWTLRLTVAIERSHCETFIAFQEQIPEFARYFYHASIYSIWRSLRNRFMFVYTKEFEDKKDSYLSGYIFQDQPNILVITSQYLNSSTFEIKTNRFVGPRNFNKNPEPVEFYILQRFDAKGTKATWETQSAMSSKMRNLKGREVVIGIFDYKPFMLLDYEKPPLYYDRFMNTTDVTIDGTDIQLMLIFCELYNCTIQVDTSEPYDWGDIYLNASGYGLVGMILDRRNDYGVGGMYLWYEAYEYMDMTHFLGRSGVTCLVPAPNRLISWTLLLRPFQFVLWMCVMLCLLLESLALGITRRWEHSSVAAGNSWISSLRFGCISTLKLFVNQSTNYVTSSYALRTVLVASYMIDIILTTVYSGGLAAILTLPTLEEAADSRQRLFDHKLIWTGTSQAWITTIDERSADPVLLGLMEHYRVYDANLISAFSHTEQMGFVVERLQFGHLGNTELIENDALKRLKLMVDDIYFAFTVAFVPRLWPHLNAYNDFILAWHSSGFDKFWEWKIAAEYMNAHRQNRIVASEKTNLDIGPVKLGIDNFIGLILLWCFGMICSLLTFLGELWRGQG

>DmelIR94e

MDCPKWILSGLCLISLVSGATVIELLGTLKLELDFEYVLLMKNRNFSLSDQVWNGTSLTKDVMDEVQVPVLQFNENVSYFLHNSISRRLVTLGFMSDANLDEHRGLLTALVANLRHMTTSRVIFLVQSKASTDFLYELFRNCWRKKLLNVIVIFQDFETTSTFYSYSNFPILQIEERIYETSLQTLPIFPDRLRNLHGYEMPVILGGTAPRMIAYRNKKGNVVYDGTVGHFMTAFQQKYNVKFVQPLQAKNPLDFAPSMQTVGAVRNETVEISISLTFPTIPPFGFSYPYEQMNWCVMLPVEADVPPFEYYTRVFELAAFLLTLGTLVLISCLLASALSLHGYATNISEFLLHDSCLRGVLGQSFVEVFRAPTLVRGIYLEICVLGILITAWYNSYFSSYVTSAPKQPPFRTYDDILASKLKVVAWKPEYAELVGRLLEFRKYETMFLVEPDFNRYLALRDTLDTRYGYMITTNRWVLINEQQKVFSRPLFQKRDDFCFFNNIPFGFPLHENSVFMEPVQKLIMELAETGLYYHWITTGFSELIDAGEMHFVDLSPHREFRAMQIQDLQYVWYGYAFMVVLSSLVWLLENLAYTVKSKTIFPTHFMQRNKK

>DmelIR62a

MYLQFLFALFLSRYQIVATENFDRAFELALFLDRIGRVHRLHAITIVNSLGSVDPSYLDDLHRGLMCNSSNHFYMLPQMTATDKDSSHVHFSSLQDEETIYLVFARDSKDAVIYLQAERARGRRYTRTMFLLRKQESQKDIKYFFELLWKLQFRSALVVVAARNFYQMDPYPTVRVIRMRRLSSYDPHHVFPPANRKNFRGYRMRLPVQQDVPNTFWYKNRRTKAWELAGLGGILINQLMMHLNVTMDLFRFEVNGSSLLNMAALTDLIVKGKVELSPHLYDTLQSNTSVDYSYPTQVAPRCFMIPLDNEISRSLYVFLPFSLTMWLCLLFVLLVVHFVYVRRLIPDGHFWAILGVPGAGQVRYGNRKPVRRFSTFLILFGIFILGQTYSTKLTSSLTVTLIRRPDNSLEELFLLPYRILVLPTDVYAIVDSLGHAEQFSTKFSCTDAENFSQKRISMHPEYIYPISTIRWRFFDMQQRFLRKKRFYFSKICHGSFPYQYQLRVDSHLKDALHRFLLHVQQAGLHDLWLDTCYRKAHRMGYLKDFSTLAELEEKLRLRPLALNLLVPAFSLFLCGMLGSGIAFLVEIRHSFGCRQKPPSINRNPGD

>DmelIR76b

MATGIELLVAAALCVACPPLNDSPPTNLIQMGENGTLSPVTELPMDVDASEAGFDADAPVETLETINRKKPKLREMLDWIGGKHLRIATLEDFPLSYTEVLENGTRVGHGVSFQIIDFLKKKFNFTYEVVVPQDNIIGSPSDFDRSLIEMVNSSTVDLAAAFIPSLSDQRSFVYYSTTTLDEGEWIMVMQRPRESASGSGLLAPFEFWVWILILVSLLAVGPIIYALIILRNRLTGDGQQTPYSLGHCAWFVYGALMKQGSTLSPIADSTRLLFATWWIFITILTSFYTANLTAFLTLSKFTLPYNTVNDILTKNKHFVSMRGGGVEYAIRTTNESLSMLNRMIQNNYAVFSDETNDTYNLQNYVEKNGYVFVRDRPAINIMLYRDYLYRKTVSFSDEKVHCPFAMAKEPFLKKKRTFAYPIGSNLSQLFDPELLHLVESGIVKHLSKRNLPSAEICPQDLGGTERQLRNGDLMMTYYIMLAGFATALAVFSTELMFRYVNSRQEANKWARHGIGRTPNGQSVAPSRWLRGWRRLNSGHGQLLGASTHGQNVTPPPPYQSIFNGGSHGDPLNRWRRPLANGNALGNGVLLGGDSEGGVRRLINGRDYMVFRNPNGQSQLVPVRSPSAALFQYSYTE

>DmelIR85a

MSIQWLKHILLLAILVNLAGTRENHIPLDLKKSSIVMVKMSQILCKARIKVLFVYFENQTSHEHTGQILKEVTKCDISNQNTPLEAVKDDGILMYMVMITTNISQPLELSLIRKKSAAKHRSHVFLLVRDADTVSDAWMRASFRQFWKIWLLNIVILYWRDGRLNAYRYNPFMDNYLIPVDNKPNEVPTLEQLFPKTIPNMQRKPLRMCIYKDDVRAIFWRQGTILGTDGLLAAYVAERLNATMMITRPHSYNNHNLSSDICFLEVAKEYVDVAMNIRFLVPDTFRKQAESTVSHTRDDLCVIVPKAKTAPTFWNIFRSFGSLVWALILVSVLVANVFCYILKSEVGRVPMQLFAGALTMPMTQIPPNHSIRLFLIFWLYFGLLICSAFKGNLTSMMVFQPYLPDINQLGALARSHYHIIIRPRHVKHIQHFLTLGHKHESRIREQMLEVSDTQMYEMMRNNDIRFAYLEKYHIARFQVNSRVHMHLGRPLFHLMNSCLVPFHAVYIVPYGSPYLGFLDSLIRSSHEFGFERYWDRIMNSAFIKSGVKVVNRRRGSGNDEPVVLKLQHFHAVFALWLVGIGMACIVLAWEHLTHNYNLAVTKRRD

>DmelIR56c

MQHLLNLLAPFGRMNVFQEIVWFVSPHQRLDQLDEFIMRIDEAFGKSATQTVVNNNTEMRMIYSSARRNHMSFVFTTGAEDPIMKVFSKVLLGRHFYVSMVIYVDKVGDMHPIYDLLTFAYNQQFFNSMVHFESMEGVNQLFGVSKFPVMSFENRTDFLKYMGKIWKQVQNARSDVGGFGFTTPLRQDLPHLFQSQGHYDGSTYRIIETFVRFINGSFKELIMPPDSLGGQVINMKDALQLIRERKMEFCAHAYALFMSDEELEKSYPLLVVQWCLMVPLYNSVSTYFYPLQPFDWNVWFFALGALLALVLLELMWLRMFGGWSGYRGAVLNSFCYIINVPIEGQLQQPCLLRFLLLATVFFHGFFLSAYYTSNLGSILTVNLFHAQINTMNDIVSAQLPVMIIDYEMEFLLNLNKELPQEFLELLRPVDSAVFSEHQTSFNSSFAYFVTEDHWEFLDEQQKHLKQRLFKLSSICFGSYHLAFPLQMDSSLWRDIEYFTFRIHSSGLLNFYARSSFGSALHAGLVQRMPDTQEYTSAGLQHLAIAFILLLVMSFLAGIVFVLETLSR

>DmelIR75b

MLQLHNLILHNLIHMAKLSHVLILHCSLSHLALLAQSKNIFTQFQPLHSDIQLNDDFLNHNILKLGVFLDINCDKSGTVLDMASAKRFFSHRYHWLIYDRSMNFSVLESHFKEAQIFVDADVTYVTHDPFSKNFLLYDVYNKGRQLGGELNITADREIFCNKTNCRVERYLSELYTRSALQHRKSFTGLTMRATAVVTALPLNVSIKEIFDFMNSKYRIQLDTYARLGYQARQPLRDMLDCKFKYIFRDRWSDGNATGGMIGDLILDKADLAIAPFIYSFDRALFLQPITKFSVFREICMFRNPRSVSAGLSATEFLQPFSGGVWLTFALLLLLAGCLLWVTFILERRKQWKPSLLTSCLLSFGAGCIQGAWLTPRSMGGRMAFFALMVTSYLMYNYYTSIVVSKLLGQPIKSNIRTLQQLADSNLDVGIEPTVYTRIYVETSEEPDVRDLYRKKVLGSKRSPDKIWIPTEAGVLSVRDQEGFVYITGVATGYEFVRKHFLAHQICELNEIPLRDASHTHTVLAKRSPYAELIKLSELRMLETGVHFKHERSWMETKLHCYQHNHTVAVGLEYAAPLFIILLGAIILCMGILGLEVIWHRHCTLH

>DmelIR84a

MIKLQVKVISWPLIILTAFLRVLQIESINTNFLELAAFEDFLRSEHLSHVLVVRGDDADGDWKIECHQKLLANYRVQFYRPEMSANFEDLMFYGSPRTAVLVLNSEHVLVRRQVFGVASEAGYFNNSLAWFILGSGRESLPVEQLIDQLLSGYRMGIDADITVALRGPDNASMLFYDVYRISRQANTPLIIEKKGLWTHSGGYQKFGNFKNTWVIRRRNFLNVTLIGSTVLTEKPPGFGDMEYLADDKQLQQLDPMQRKTYQLFQLVERMFNLSLAISLTDKWGELLDNGSWSGVMGQVTSREADFAVCPIRFVLDRQPYVQYSAVLHTQNIHFLFRHPRRSHIKNIFFEPLSNQVWWCVLALVTGSTILLLFHVRLERMLSNMENRFSFVWFTMLETYLQQGPANEIFRLFSTRLLISLSCIFSFMLMQFYGAFIVGSLLSESARSIVNLQALYDSNLAIGMENISYNFPIFTNTSNQLVRDVYVKKICKSGEHNIMSLQQGAERIIQGRFAFHTAIDRMYRLLLELQMDEAEFCDLQEVMFNLPYDSGSVMPKGSPWREHLAHALLHFRATGLLQYNDKKWMVRRPDCSLFKTSQAEVDLEHFAPALFALALAMVASALVFLLELFLHWLPDFRRRLGTMST

>DmelIR92a

MLLQPLVMHLSQLLRIIVGQYFAEFPSILIVYNNSASTTPLQLEYLSALELVLRELSKPIRLQWINVAFLKDLNDLEDQVMGALNSSVTEGFITILSQTHHFIHARYYATRNANVRLKDKRYLFLCEDESPAELLCMDILQFYPHHLMVRPGTETAPTGPTGPHPDPRRGGGASVSTKNKDDGEGGAGNKTTSPYRDINFELWTQKFVGAVGNLDALLLDAFLPNETFANRVELYPNKLLNLQRRSLLVGSITYVPYTITNYVPAGQGDVDPIHPQWPNRSLTFDGAEANVMKTFCQVHNCHLRVEAYGADNWGGIYDNESSDGMLGDIYEQRVEMAIGCIYNWYDGITETSHTIARSSVTILGPAPAPLPSWRTNIMPFNNRAWLVLISTLVICGTFLYFMKYVSYRLRYSGTQVKFHHSRKLEKSMLDIFALFIQQPSAPLSFDRFAPRFFLATILCATITLENIYSGQLKSMLTFPFYSAPVDTIEKWAQSGWKWSAPSIIWVHTVQSSDLETEQILARNFEVHDYSYLSNVSFMPNYGFGIERLSSGSLSVGDYVSTEALENRIVLHDDLYFDYTRAVSIRGWILMPELNKHIRTCQETGLYFHWELEFIDKYMDKKKQEVLMDLANGHKVKGAPQALDVRNIAGALFVLAFGVAFAGCALVAELLIHRMDLSK

>DmelIR94b

MSLIFNLLFILILSQAVSQETEFLQLKYLNNIVRSMIKLHKMETLVIVKHHLDNNCSLQNWNAHGMGIIRTNDQGKLIMKDTFNSRTLAIICIGQNSHITLLRNVFETFGKVQQKKIILWTQMELKEKFFQEISKKSRDLKLLNLLVLKAVTKDKLLIYRLNPFPSPHFKRIENIWTPNDTLFMDTKFNFHGMTAVVKHDYNWTIQMGNIRKFPISRIEDKEVIEFALKYNLTLQFFNDVERFDIELRKRIILKSNSTQPIDSGIPMVFSSLLIVVPCGNYLSIQDVIKVSGIEKWIFYIILVYVIFVLIEITFLGVTILISRQSRHQMIPNTLVNLCAFRAILGLPFPETRRTSLSLRQLFLAIALFGMIFSIFINCKLSSMLTNPCPRPQVNNFEELKTSGLTVVMDHDAENFIEKEIGVDFFNQYMPRKVTLTFTERAKLLFSLKGNHAFTLFSESFAIIESYQRSKGLRAHCTSEDLIVAERVPRIYILENNSILDRPLRRFIRQMQESGITNHWLKNIPSSLEKNLMQITIPYDRERVHPLSIEHLTWLWCILILGYSISMIVFFVEMSLKRRKKNLENRAPNICIC

>DmelIR94c

MSKVFKLLVLPLIYLSLTKGSKNPQLKFLRELINVIEEGREIRTIMVIKHSRDEYCHLDQWNPRGSPILRTNEMGSIRISGYFNDQAVILACMGENSDYGLLKSLANAMDNMRQERIILWSEREPTKMLMDYISQQADRYNFAQIIIVTMNEDVDAVPSLHQLNPYPTPRFRQITNISNIRRTSFFGCGLSFQGKTAILKESVVSNIRFKVWSPSGPIPLSELKDYEIVQFAVKYNLSLKLYDQNESKSDHFDIQLGPLFITKDFPTQMAFVSPNTACSLIVIVPCSPKWRFMDVLHKLGVLKLIGCLLIAYAVFVLIETLILWLTHRISGREVRLTSLNQLLNPRAFRGILGLPFPEFRRSSISLRQLFLVISVFGLVYSNFVSCTLSALLTKPAQNPQVRNFKELRDSGLITIMDKYTHSFIEKHIDPEFFDHVLPHYLILQKKEALRMIWNFNDSYSYVMYTTTWKSLNTVQKSFDERVFCESESLTIAWNLPRMYVLGNNSVLKWMLSRYITYMPQTGIPDSWTEQLPKVLKLLYNVTSPRRIKEGAVPLSIQHLSWIWHLLFIGESIATLVFIVEILLQKSNQHTSNMRERSSEDDDFV

>DmelIR94h

MLSNISFSSAPELVDLYGLVLKFLVSSETTLFYFNPTGQKCSWETLPRTILSNHPQIIWFREETYPGLYKRHSSNLFVMACLSSTSYDGQLQLLAESLTRYRSVRVLIEVQDKEGSFLASQILLLCQQHSMLNVVLYFSRWTRTLNVFSYLAFPYFKLLKQRLSGSLRPKIFINQLKDLQGYKIRVQPDLSPPNSFSYRDRHGECQVGGFLWRIVENFSKSLKGDTQVLYPTWAKAKVSAAEYMIQFTRNGSSDIGVTTTMITFKHEERYRDYSYPMYDISWCTMLPVEKPLSVEILFSHVLSPGSALLLILAFILFFLIVPQLIKCLGITFRGRLIGMASRIFALVMLCSSSAQLLSLLMSPPLHTRIKSFDDLLTSGLKIFGIRSELYFLDGGFRAKYASAFHLTENPNELYDNRNYFNTSWAYTITSVKWNVIEAQQRHFAHPVFRYSTDLCFSSETPWGLLIAPESFYREPLQHFTLKINQAGLITQWMTQSFHEMVRAGRMTIKDYSRTNLMKPLRIQDLRKCWVIFAVGLGTSTVVFTIELLLIYTNVFLNSL

>DmelIR94g

MSTAVNSVHSKLVSLISRGQELTSIFFYAPAKEKCHLEDTISSATWGLPLVIWRTDRTVILNGFIGEGLLVLACLPGFHWRALLGSLARSLKYLRQARILIELMQDRDEFLVSEVLQFCLSQDMINVNAIFDDFPETENLSSFEAYPSFEVVNQTFTPDTQVSDLYPNKMLNLRGGVIRTMPDYSEPNTILYQDKEGNKEILGYLWDLLEAYAHKHNAQLQVVNKYADDRPLNFIELLDAAQSGIIDVGASIQPMSMGSLSRMHEMSYPVNQASWCTMLPVERQLHVSELLTRVIPYPTLALLLLLWIFYEVLRGRWRRHSRLQSIGWLVLATLVSSNYVGKLLNLFTDPPSLPPVNSLAALMESPVRIISIRSEYSAIEFTQRTKYSAAFHLALHASILIGLRNAFNTSYGYTITSEKWKIYEEQQKRSSKPVFRYSKDLCFYEMIPFGLVIPENSPHRAPLHSYTLLLRQAGLHDFWVNRGFSYMVKAGKINFTAVGERYEAKTLTITDLRNVFIIYVSVLLISLILFTCELFVSWVNYWLGF

>DmelIR94f

MWQQVLLAETSNWFRSDVLQRFWTHLRVEIRFRTMLNYRLESCDCWFDNVLGSDNSTALLWNDQTYPHYLRRRQDTDILVVSCLRFHQYQEVLLALSLMLDQMRSMPVVLQLCGDEDSMQELNSARLLLKHSQDLKMPNVVLLSSTFFTSATLYSYEMFPEFNVQKLVYQAYLTLFPYKLGNLKGHPIRTVPDNSEPLTIVRKTLNGSIAIDGLVWQFMIEFAKHINATLQLPIEPHPEKSIKLVQILDLVRNQTVDIAASLRPYSLNVQRSSTHIYGSPMMVGNWCMMLPTERVIGSHEALTRLMKSPWTWLILLLFYSVHRFLAQKTRLRSSLIHLIKLLINLSLICFLQAQLSAYFIGPQKVNHISNMQQVEESGLKIRGMRGEFMEYPIDMRSRYASSFLLHDLFFDLAQYRNSLNTSYGYTVTSVKWELYKEAQRHFRRPLFRYSEEICVQKLSLFSLIQQSNCIYCYRSRIFILRMHEAGLIRLWYRRSYYVMVTAGRFPIGDLSTVHRAQPIRWTEWQNVVLLHGVGLLFSVVVFVIELTVHYANVCLNNL

>DmelIR100a

MATTLQLIMLALVGGTLGQANNTDHKQVLTSIVKQLEGGLELHLRTSEDGGNDLVQFLMQEKSSIIISAKQEEVPSRAKIMRHHFFIFDGVHQMQEIRTSLFNTDGFYILALENNTIEDDVLLMEFAADVWLQHGHSRIYYVQLSKKSVLLFNPFLQRLVVVQDSKTYSRIYKDLEGYHLRIYIFDSVYSSVIGDGENKVLSVTGADAKLAKTVARQLNFTADFVWPDDEFFGGRLANGEYSGGVGRAHRGEVDIIFAGFFIKDYLTTHIQFSAAVYMDELCLYVKKAQRIPQSILPLFAVHMDVWLCFLLVGLLGALVWLILRAVNLILGIEGVPDGSRATRISYFGAARRIFVDTWVIWVRVNVGRFPPFHSERIFVASLCLVSVIFGALLESSLATVYIRPLYYRDVNTLRELDESGQPIYIKHPAFKDDLFYGHNSEVYRRLDAKMMLVAEGEERLIEMVSKRGGFAGVTRSASLQLSDIRYVMTKKVHKIPECPKNYHIAYVLPRPSPYLEEVNRIVLRLVAGGIVGLWTGEAKERAKWSIQRFPEYLAELDVGRWKVLTLSDVQLAFYALTIGCLLSAIVCMAEILLGRQRRLHSPK**GR**

>CpomGR1

MGVMPIMRVPRDAQTTKRTTYNWISKATFWAYLVWSLESIIVVKVGRERYENFQKSSNKRFDEVIYNIIFLSILIPHFLLPIASWRHGPQVAIFKNMWTHYQLKYLKITGTPIVFPNLYSLTWGLCFFSWGLSFAVILSQHYLQDDFELWHSLAYYHIIAMLDGFCSLWYINCNAFGTASKGLAQNLHKALEADHPALMLAQYRHLWVDLSHMMQQLGRAYSNMYGIYCMVIFFTTTISLYGALSEILEHGLSYKEMGLFVIVGYCMTLLFIICNEAYHASRKVGHEFQVRLLNVNLGAIDHSTQREVEMFLVAIAKNPPIMNLDGFTNINRELFTANISFMSTYLIVLMQFKLTLLRQGARKAVRAIVKAIFNTTTMLPDEEYEDEE*

>CpomGR2

MIPDHYFDEGLNGSLYPDDMKQLNTVKLVYEKTQADYEQEQRDMLSSQDGDTCETHDQFYRDHKLLLVLFRALAVMPITRSRPGTITFSWKSRATMYAISFYIVATVVVLMVGYERVMILRSIKKFDEYIYAVLFVAFLVPHFWIPFVGWGVAHQVAIYKTSWGKFQVRYYRVTGENLQFPNLQTQIVIISVGCLLLAVCFLLSLCALMDGFLLRHTTAYYHIIIMINMNCALWFINCKGIKIASQSLSECFRRDVNVEISAKLISRYRFLWLNLSELLQSLGNAYARTYSTYCLFMFFNITIAVYGALSEIVDHGFGFSFKEMGLFVDAAYCSTLLFIFADCSHKSTLKVAAGVQDTLLGIDVLAIDRPAQKEIDHFIQAIEMNPAVVSLKGYAHVNRELLTSAISMIAIYLIVLLQFKISLPKADS*

>CpomGR3

MAFYTNNSLFPNQPPIPNGIAAQMDEKSKNKIIFLDVTPNRTPRLPTPNNAIAPIQDNLINPDITRDIIYENIKPVFTLLKIMGVLPLSRPVPGVTQFQPTSPSMLYSVVVYCSLIGYLLYLSLNKVQIVRTGAQEGKFEEAVIEYLFTVYLFPMIAVPILWYETRKIAEVLNGWVEYEIAYKKLSNRVLPVGLYKKALAMSIVIPALSTASVIITHVTMVHFKLLQIIPYVFLEILTYMLGGYWYLLCETLSICAHILAEDFQQALRNIGPAGKVAEYRALWLRLSKLARDTGIANCYTFTFMSLYLFLIITLSIYGLLSKISEGFGVKDIGLALTAFCSIMLLFFICDEAHYASHNVRLNFQKKLLMIELSWMNADALTEVNMFLRATEMNPSQISLGGFFDVNRTLFKSLLATMVTYLVVLLQFQISIPDDSRVQEADDDDDFVNATASVTEAPTTLTTITTLLTTLAKKKKKH*

>CpomGR4

MNASMTIFFQIMAKIFRFSRWFGVAGSGNVLWKTFGLCILLLLGVIEGVAIWRVVKALAGLAIDIEGHRSVTARLAGATFYASSITTLILSWKLSSSWETIASYWASIDRSIAINVSSDKKIKTRMITVTSVMVTCVVVEHAMSMMSQVGFECPPSLILKRYTLMSHGFLLLRTDYSIWFAVPLLFMSKIATILWNYQDILIVLISMGLTSRYNTLNQYVAKFSTLSKDPWNPHGECSKGHTWRRIREAYVKQAQLVRQLDQSLGGLILLSNLVNFYFICLQLFLGITQGLSGDLIKRLYYVVSLVWLCVRVSCVVLAAADINVHSTKALRHLHASDRHYYNVEIVRLQNQLSKDYVALTGLGFFSLNRTVLLQMAGAIITYELVLIQFDDHGFTEHKSALNSTTF*

>CpomGR6

MRISYPWVFSFMPYSIPLGIITQFLHFQATFIWNFSDLFVICTSYYLTSRLDNVNRKLLIAQGKYLPASFWRSAREEYSRVTQLIRKVDQVISGIVFISFANNLFFVCLQLFNTLENGIKGTGACRASRLKGTTLFAGYEGPAYFIFSLVYLISRSVAVSLIASQVNSASLLPAPVLYDVPSPVYCIEVQRFIDQVNADNVALTGLQFFTVTRELLLTVAGTIVTYELVMLQLTPSAQVPTANGTMT

>CpomGR8

MKSTQSYNFLEIKKRNNYVEDEELHAEGALALLLRLCCWARVAPRRVHTRSGWRFETFASLASLQAFGMTLLNAAIITSIILDFYQEPEKRLRVGATFLKTVIWLTEIALVMGIASLAVYTGPAQVECLKRVLKQLQKINSDLNLNNPSAKTEKIKSIIMVILLTWVVFIMIMDVVFYYPHSLEEGTVCILCLQLPFYVAHLLWWQAVLRWALTVEAVHGAAATVNHRLQSFRLATMKPVSMTLDEFLSKPRSIGTLLNCIRDPALTAKTTELGIQNFTYPAQVKTLIRRLALSYERIGDIMRQMNETNGLLLMIILTTTFMKLVVTPYYMLIYALDDESNVVFDMMLSLNWSLAILAILVLTIEPCHRVHSQRERTEVLLRQLTTHLAPSRQLSKELEQFTKLIVLNKPRFTALGIYTLDRPLMAMMLSGITTYLVIIIQFQKFSHKLEYD*

>CpomGR9

EGPRICIDVPPVCVVSGALALLLRASSYAGVCPLRFTQTHDGWRPSPSAPLAAVQRLIMTIFNALMLAAFILDICQEPGQYIRIGETTLKMFVWCSDMLLMMMIASVAVYMAPKRMNHLVHMLDQLRQVSTELKMNPSARNEKIKSIAIVFIPLWAASILIADFYSFLGPLMNGKMWYIMCMYGPYYVGNFMGILVLLQWSCAVLAVHATVVAVNDELATLRRAKFDLGPTTTLEDLLRPPKPERNTLVGCFTKPAKSPGNSMTLPQAQATIRRLAFSHERISELMRQLNASNGVFLMFVLMSTFIRLVLTPYYLLQRFDHDERILYELLLQINWTLFHVITLLLTIEPCHWTQEQRERTQILLSHLIVHLAPKCERLSKELDQFAKQILLSGAKYMPLGVYTLARPLMATILGGVTTYLVIIIQFQKISDQL*

>CpomGR10

AALILDFQEDPSKRIHVGESAVTACVWISDLGLVLAIASLAVYRGSARMKKFIKLLRELHKINDDLHNTKCVKMEKIGVIAVTSFLMSAMIIQVAQIYLLTKLFINRGCNWSIMLMYSSYYVANCLGLLALLQWGFVVLAVYSAAATVNQHLLRLHHVKLKAKETAMSEVYLSPPKPRVDCFIEGHYDIAPPDFAAYPLQLQSMVRRLASSYGHIGELMRQMNETNGTIIIAILMAVFLHLLVTPYYSLRALNSNANVLEVVLVPLCWTFLQIAILLLTVEPCHWTHEQRETTKFLLSRVTVRLAPKSKLLARELDHFAKQICLSNIKFSPLGVLTLGRPLVASMFGGVATYLIILVQFYSHTDD*

>CpomGR29

MGYTSDLVLRFLRNYFPLKGILLVRAFFGHYFSFKCPKIYLKLHKVYCVVVTLVCVIIIFIMTNEWRKWVIFELVIMTLASILIEGDCCGKFLSFVECTDHSFGLGRRNLASPRLYAAFFIITSIRLYIDYINIHAFFVNPVLYSAFTFLYTGLDLNHLLRIIVFDILYERAKHLRNHFESVFSRVNGDDNMISEVKRGILLYKELIGSVTMLEKIQVTYLLALVARFAANVADIHLILCVEKRDEMLLGKMCRLGSESLYLAALVCAPAVIMELVHNEVDKITSILTIQHAIATDRELGA

>CpomGR30

RYFTYTIRLFNLKCTNFYKVFFPIRLLFNRDVIFFNNFAMEGILNFLKIYLPLNRILFIRAIFGHHFSFDCRMLYLNIHKLYCIFVSFIFPLIIYSFSDVLESTRYAIFMEFFVCIWITLIVEDNCFQEYLTSIKRTDQLITHGRFNLASYRLYMVYFFITLLRMIIHFMSVKFFSISLYKFLTYAFVYMTLDLSNIIRVLIFEALYQRMIFLRNHFESIFDRPTNDCRNIISEVRRGLLIYGQLLDSVKLVNKIQVTLFITLALRFVGFALKLNMMLFKVNSIWDHTRMFMYTFESAFILALDLTPAVFSELTHNEVEKIETMMSHKHSMCTHRSLRAALSKGILYFKLRPFEFKIWRVIPVDSTLIFSFLSVFLTVSLLIFQFHRMM*

>CpomGR55

VNYKNRDVEALLKQQMKRFRIQLALSISIVNSITLMYFIEQYKSGKFSEMISTLIVIVFDLTLEYRFYFENIVFFVLIDILVELLKYLNQSILSSIEKLNKDDIDEVGDTGRIANELEVWSEIIRLLAIACHRLQVCFGGQVLLSFFTTILYYIMYFYQGILYSIKQELLWDSSISSVIMALSMYIAMKFVIVWSGQRAQNEAETLEANLTKLQTLLVKKRNLSRVLK

>CpomGR58

MNKNYKNNNGHVGCLLENYIEKEILDANWPLFFCQSVLLMPPFCVTNGYVTPVDKKYYIKILLGVCLHIAARVYYYICLYESFLQISMNPFVIFMATANSATYCIAIIVIYFVNVIQSHNNLQVMLKLRQALFTINLEKQEILKDHKIWNFIYIIGLVSIEVVVSICYCRMEQRMSFIFSKITFLVCDINLVCLYRTVSFGASLLASWNRKMMIYTNQIVTKKEIESMFNAYMHIIDALGLCKK

>CpomGR60

MILDNVIDREFQMMLLPLNILEILYCQPKFRITETFITPNGIRENLLCTLGVLLMILANVGYVSINSYIPGNDEISDIIHSFICTDAAFYIVYCLLMYAMNIIYKNQIVQLIIKMQKAYRVLQNENGLRRFQKSNWIFVVAVFLFYFTYNLSYTIFNIYRVTHLLYDVVLFYFDVNIIVAIRIVKFLEYELILWKKELNKFLKTCSTTNHNQLVKYL

>CpomGR61

MENSRNIIDEKILDALSPLIFAQNFFLFPKFMITERCIAPIAPRSYTSSFVGAVLMLLIRIYRLVTVCFYNYFGENSDALLLANFVVGCFGTIFSYVINVVQSANAVYMVIELQEALWCLSSNIKQSLSDYKFWNIVNIACIFGGYILYTGLFGVANQETHGEASFLVSHLVSITYDLNIILATRTVILTASILEAWNSKMSEILSEETEVRENCSQDMFSAYEKIINAFNLCKKAYQFGIFYHTFQTFHSILYSMQLFLEYAKSASHEELKVFGLLRGVTYFAWNSKNFLLLVNVSVACERFYAALRDAE

>CpomGR63

MRVSGMRVISADRRSTELQNTFKPIKTLTSIVSLNCSGPNKSWQLFWIVLKALASASVLGCLTFYCLYIKIRYHYNDVILSIKLTDVIQMSYDYSQYLVDLFFVFKYGRDTYAEYDKQLINIDQILISTNYSAIKRRHINLIVYFIAIWIFSSVCDFTAWAVSYGSLLPTLYSTSYIYLLIKMISTLDLMSHVMHVEYRLKGIVNQLQECYCDTKPFPGDFSDPIGKKFWFYCESPSKPGNTNETPPDRTLVCNSPQAVRWLSRCYLLLCEQCVFINSMFGTRILLNSLSLLIDMIRFTNIAVRLVIGSQPTMYASGNYPAAANVLRMV

>CpomGR68.1

MIQFTVISFYYVLVLMVVGVLKNINEQMKSIYCSNRVNAQFIKVEKIITLNQIEVVYVHMLEMKREINRAFQASILATAIQCFHSIVSESHILYHGLVVEHTLTTHDVCNCSIWIVYQLIKIYIISCSGSMLKEQVSKIGRSLHNILPGKDDARLYLEVQHFSSMILYQNAEMTVYDFFPLDATFTFNVISAAVMYIVMLVQFDATKKS

>CpomGR68.2

MFTSLQKYFSPVVNEDEELCFLQIFKPLYIVLSALGLFPQAVRFPDGIQNTTLNIKNSVINSMCTLFMIVIVHAFLVFHLQELNISSKDNSMTEDNMTLMNYIIGLVLEILFCTVSYFCVIRDRNLYITMLNDMAVCWDKLAMGKRRLILGRLRVHINCVVLTTVLAMILVLAVATYTSYLGVWKMILITLTFVLPDLIQFTMIAFYLVLMLMVVALFKNIEEEFKVISLVKNNAPNDLVEAHLVVSIREIREIYVKTMEIKRRINEAFQAPILVAMMVCFLELVSMPHMIYHGLSFQANFTMHDAVECTIWVLNQLLKMYALAKSGALLNSQVNEIGRTIHNIPISGDKDLKLYLDVLHFSSLMTYQDTAITIYGYFPLDSTLVFNIVASAAMYLVILVQFDKPE

>CpomGR68.3

MSSALTKYFSPVVNHEEELCLLQIFKPLYVLLSALGLFPLSIKFPDGIYKTNVDLKNSTINSAFTIFMIIVIHGSFVFHLQELNISSKDNAMTESKMTLINFTVGLIIQVLFCTVSFFRVMYDRKIYITILNDMADCWERMAMGKRRLILGRLRVQVNCVVLPSVLLSFLLILISQYTEIDLNIWKLILISLTFDLPELIQIAMLTFYFVLVLIIVALLKNIEEELILILHVAKNNRNYPVEADMRMDMGEIMKVYVKTLGLKRQVNAAFQTSILVALMSTFHLLVSLPHLMYHGLTFQTNFSTHAIIECSAWAVNQLIKLYFLSRSGDLMTSQVNEIGRTIHNIPISGDLDWKVILEVQHFSSLMTYQDAKMTVYGFFPLDATLSFNMFASAAMYLVILVQFDKPE

>CpomGR68.4

MFSSLRLYFSPFVKKNEELRLLQIFKPLYLVLSFLGLIPCSLDLPQGNVDCIILHKSAFKHSCSAFLTLLIVYVFFGLHVYEVLTSHEENILADDKMAKANYIIELVTQFTFCNATYFCAFRYKEIYVSILKEITRSWDDLPYVNRGIILGHLRVKVNCGVIGTICLILLTLTAVTYAGSSSLWKRILITMSFNLPEMIQFILVAFYYVFVLMVVALLKNIEDHCRMFMKARRSIKNCSKVELGRIPITLSQMQCVYVKALRVKRQINVVFQAPIMFSLLQCFHTMVSESYDICQGLLYQDNFTTHNLVECSYWVLLQMLKIYALARSGSLLKLEALKIGRTIHNIRSDDEEIKLLVEIQHFSTLMAFQSTEITIFGYFPLEAPLMFNMVAAAAMYLIILVQFAKTH

>CpomGR68.5

LKIHKYLFLIIYFSISSSCTKHARNMFAALKKYFSPVVHQNEKLSLLQIFKPIYVLLSTLGLFPQAILFSDDGQDATFAWCRALFMIILIHSFYIFHLHELYIFNKDNSITQGNMTLTNYIIDLSLQVLSCTVSYFHVIRDRNLYSRMLKDMAGLWDRLAKGRRRQILGQLRVQMCALLCPGTVIIPLLLAITYRGSLRVWKKILFTVTFILPELIQFLMISFYLVMILMIVALLKNIEEEIKILALRNNICYNLLEADELGMSILKIKNVYVKTLKIKRQVNAAFEALILVALTVCFHELVGLPHMIYHGTVYVPNFSINNTIGLSLWVFTQLLKMSALAISGALLKSQVRSLRS

>EposGR1

MKSCSQPRSLASFLDTILTANLTLRPDSEPGCVVSGPTAVVLRASRVFGAAPLSVSRARAAWQIGPSSSQATLQRVLITACNIIMLAGLGWDYSQEPDRRIRAGETTLKGLIWVFDLFLVMLIANITVYTGPCRMANLIQILKQLQQVLRSDDLCTIIISIDKLSPQTNVFHIHSQILFFILFYINFNIHDQRANTLAKSINSDYIRRANNVHVLHILLRPCCGRDDGAAVALHGSRRFDAISIVSSYLWLRITLPDRAPMQASQAQATIRRLALAYDRISEVVTMMNAGNGLILLVLILSIFYRLVLTPYYLLLGLLGDDEIVLSWVVLQINWGVTLMINLMAIVEPCHWTQEERDDTEHLLGQLTVYLAPKFERISKEVDQFAKQVKLDNTKFTLLGIYTLGRPLMATILGGVTTYLVIIIQFEKISDLC

>EposGR2

MGVMPIMRVPKDAQTTQRTTFNWISKATLWAYLVWGFECIIVIKVGKERLDNFQNSSNKRFDEVIYNIIFLSILIPHFLLPIASWRHGAQVAIFKNMWTHYQLKYLKITGTPIVFPNLYSLTWGLCFFSWGLSIAVIMSQHYLQDDFELWHTFAYYHIIAMLDGFCSLWYINCNAFGTASKGLAQNLHKALEAEHPALMLAQYRHLWVDLSHMMQQLGRAYSNMYGIYCMVIFFTTTISLYGVLSEILEHGLSYKEMGLFVIVGYCMTLLFIICNEAYHASRKVGHEFQVRLLNVNLGAIDHSTQREVQMFLVAIAKNPPIMNLDGFTNINRELFTANISFMSTYLIVLMQFKLTLLRQGARKGVKTIMKAVFNTTTTLADEEYEDEE

>EposGR3

MNVNEGVHSTWTDRTSAEVQETLKPLHHVTKFFSLNCCPKSKTRWQKTSSVAKATACAAVLGFVNFYTLYIKSRDVFDSINMSIKLTDAIQMCYDYCQYLVDLYFVHKYGSGVSLEYFKRYADIDQVLGITVYATIRSRLVKLIVYFIVIWLVSSVIDFTAWTLSYGPYTPTLYAISYIFFLIKMLNSLDITSHVMHIEFRLTYIKDQLQDCYCSTRSLTGNLNDATCNQKWFYCENTTRISKSSIYPESLVAHRNNHQVIKWLSKCYLNLLEQCQFINKMFGIRILLNSLSLLIDMIRFTNIAVRLIIGSQVTRYDPGYFPAVANLLRMLTCALVIGSLVAHCERVYRQADATLSVIDHTLINKDPDGDVRAALTELRGLMQSRRVDFHMAYFFRLDYSLLVSIASVVVTYTIILLQNVPN

>EposGR4

MCTVLRKYFSPLLHRNEDLRLLEVFKPLYYILSIFGLFPYSIDFLYGKKYLNVAFKSASFTLACNFVVPTVICVFFALHMLELDVASRDNAFTEDEMTKTNFIIEMVCQFLVCMTAYICAFKNRSLYINVLNEMSGCWTNLPKNVGSKILGQLQIKAHCVLFGSILLVPLLESPMTYLSSATAWKKILVLVTFVLPELIQFVLIAFYFVLIMMVVALLENIEEHVKILYHSKSCWTSSNIVEDAKPLPASLRQLRDMYARALAVKRQVNAAFQAPLMLLLAQSFHTLISEAHFLFHGLTFQNDFNVLGVFDCCVWIVLQIIKIYILGHSGAILNQQATEIGRTLHNIPADIDEDISLYLEIQHFTTLMKFQAANITVFGYFSLESSLIFNVTASATMYLVILVQFDKST

>EposGR5

MTKIFRYSRWFGVAGSGNIVWKIFGVFILLLLGVIEGVAIWRVIKALAGWAVDIVGHRSVTARLAGTMFYASSIITLILSWKLSSSWKDLAVFWASVDRNMAINVPPDKSLKSRMISVTSVMMACVILEHTMSMMSQIGFDCPASLILERYTLMSHGFLLLRTDYSIWYAIPLLFMSKVATILWNYQDTLIVAVSMGLTSRYYRLNHFVAKFSAAVNKHVPWNSQSNRRNEYTWRKIREAYVKQAMLVRRVDAWLGSLILLSCLVNFYFICLQLFLGITQGLSGSFIKRLYYLVSLAWLCTRVSCVALAAADINVHSKRALRYLHACDAHCYNIEVERLQNQLSKEYIALTGMGFFSLNRTILLKMAGAVITYELVLIQFDDNGSTAHKPYQYNATF

>EposGR6

MRSGITATCVSVIILVMLLGLELTAIWKLIRAFGGWAVVKGSITARLSGAIFYGNALFSHLLSWKFISSWEDLSLHWTQIERAEVVRLPRDNKIKNRMTIVTSFVAACALVEHVLSMMAATGLNCPPTEYFQQYILRSHGFLVHAWEYSIWIAVPIFCLSKIATILWNFQDLIIILISMGLTSRYQRLNSCVEYILKSEKSKGKLERVGTDKYAEVQTWRRLREAYVRQAALVRRVDGSLGALILLSNFNNLYFICLQLFLGINNEQGPLINKIYYFLSLSWLILRACSVVLAAADIHKHSRSALPFLHACPCRVYNVEISRLKTQLTHDFVAIKGMGLFALDRKLLLEVAAVILKYELVLIQFDK

>EposGR7

MFLFVRKYFSPFVHQDEELSFLQIFKPMYYLVSAVGLFPSSIKFPTGKTLTVVYKSTTINLACTLLMATTVCVSFSLHMLELSINGRQELNNFEEDDITLTNYITNLVLMLIFSVIAFISALKNRSLYIKILNEMAECWAVLPNSVGSNGILGPLRVQVNCVVLGSLLLMLIVQLVVTCTADLPKSKIALIAMTFNLPEMLQFTVLAFYFVMIVMPVAILKNIEEHFGMIFDVRRVCDVENDFVGLPLGSSLASLRQLRGVYARALAVKRQVNAAFQAPLLLITALSFHTIVTVAHGIYHVLTYQNNFTTHDVVEECFWVVYQLIKFHTLGYSSALLELQANKIGSTLYKISTHINEQITQYKSSADVNKEIKFNLEVQHFASMMKYQDTKITIYGLFPLKSSLLFTAVASAASFIVILVQFDDGK

>EposGR8

MFSFLRKYFSPFVVQNENLSLLQVFKPLHFCLLVVGLFPSSIEFPSGKRDFTIVFKSSSISLACTLLMTVLISVSFGLHMLELSVSSKENAFTIDQFTLTNYISNLVLMLLFAGMAYICALKNRNIYIKILNEMAGCWADLPNSASDSILGQLRVQVNCLVLGSLLLMLIMQLVVTGTSDVPTSKIVLIAVTFNLPEMVQFTLLAFYFVMIVMIIAIMKNIEENFALILPMRRVNGVDNHYVRLESGSALASLRQLRGVYARALAVKRQVNAAFQAPLLFLLVQAFHSLICDAHGVYHGLTYYRDTFSTHDVVEEFFWVFYQLIKFHILGFSSAILKLQADKIGRALYNISAHVNKEITQYKSWADINDEITLHLEIQHFASLMKFQDILQYMAFFH

>EposGR9

MCIYSYILVTVLVICTIFGLASEINVGVELSVRMTSRMSQVVSTCDVLVVVATAGAGVYGAPMRMRNMLKFMDSVASVDNSIGAQYSIMTERKLIAVLLAILLFFTVLLADDFCFYALQAKKVDRQWDVVTNYIGFYLLWFVVMVLELQFAFTALSVRTRFRAVNDAIALTARHVAVPLEKLEHPTPVNMFAIRVAPAEARRSASNVSLLVDSLPGAQLEHPVIIRKTVNGEPRLIVPPCEAIRRLAALHGALCEVVQRIDCSYGLPLIVILLSTLLHLIVTPYFLIMEIIVSTNRIHFLVLQFLWCVTHMLRMFVVVEPCHYTVMEGKMTEELVCRLMTYAPSGGALPSRLELFSRQLMLRSVTYSPLGMCTLGRPLIASVIGAVTTYLVILIQFQRYDN

>HarmGR1

MNKEEHGFRVYNTNTVHKNETRKREMFQRIDEKDGIKEYDAKDLYGPEITDKDGALLDAHDSFYITTKSLLVLFQIMGVMPIMRVPKNAQTTKRTTFNWISKATLWAYLVWSLECIIVVKVGRERLANFQSSANKRFDEVIYNIIFLSILIPHFLLPIASWRHGPQVAIFKNMWTHYQLKYLKITGTPIVFPNLYSLTWGLCVFSWGLSFAVILSQHYLQDDFELWHSFAYYHIIAMLDGFCSLWYINCNAFGTASRGLAMNLHKALEAEHPALKVAQYRHLWVDLSHMMQQLGRAYSNMYGIYCMVIFFTTTISLYGALSEILEHGLSYKEMGLFVIVGYCMTLLFIICNEAYHASRKVGLEFQVRLLNVNLGAVDRSTQREVEMFLVAISKNPPIMNLDGFTNINRELFTANVSFMSTYLIVLMQFKLTLLRQSARKTLKTIVRAVFNTTTTILDDDFTDDVDEE

>HarmGR2

MTIPDHLFDEGINNTLLQHDMRHVQQNRIVYEKTQREYEQEQRDMLSSQDGDTCEIHDQFYRDHKLLLVLFRALAVMPITRSRPGTITFSWRSTATMYAVCFYIAATAVVMIVGYERIMILRSIRRFDEYIYAILFVIFLVPHFWIPFVGWGVAHQVAIYKTNWGKFQVRYYRVTGENLKFPNLKTTIVMISVGCLLLAVCFLLSLCILMDGFLLRHTTAYYHIITMINMNCALWYINCKGIKIASQSLSECFRRDVEAECSAKLISRYRYLWLNLSELLQSLGNAYARTYSTYCLFMFANITIAVYGALSEIVDHGFGFSFKEMGLFVDAAYCSTLLFIFVDCSHNSTLTVAAGVQETLLSIDVLSVDRPTQKEIDHFIQAIEMNPAVVSLKGYAHVNRELLTSAISMIAIYLIVLLQFKISLPRDPQIVAT

>HarmGR3

MTVPIPNGFPVQINSKPKNKIIFLDVTPVSTPIKPHSPNVVAPMRNNLVAPHISNDIIYENIKPVFTLLRIMGVLPITRPSACVNQFQIASSSMLYAILVFLSLVSYVLYLSLHKVQILRTAEGKFEEAVIEYLFTVYLFPMIAVPLLWYETRKIANVLNGWVDFEMVYKQLSGRTLPVKLYKKALAMAVIIPILSTTTVIVTHVTMVHFKPMQLVPYVFLEILTYMLGGYWYLLCETLSICANILAEDFQNALRHIGPAGKVAEYRALWLRLSKLSRDTGIANCYTFTFVNLYLFLIITLSIYGLLSQISDGFGIKDIGLALTAFCSISLLFFICDEAHYASHNVRTNFQKKLLMVELSWMNTDAQTEVNMFLRATEMNPSQISLGGFFNVNRTLFKSLLATMVTYLVVLLQFQISIPDESQNRDEEEEVPYNITSATTEAMTTSTTTIMTTVLTTLAKKKKKN

>HarmGR4

MEIKLCKLFVVLTEGIEYMKGNSVKNLNEKKRDDFLPTLNNVFLKARFFGISGYGLTISFFWSLILFSMLVVMESVAIWRVVTLLGEWLVSASNNGLIGRLSGAIFYGNALISLFLSSKFVHSWRSLSNYWLRMETSTALDFPADVRIRKRTIYITAFVVSVAVVEHILSMISATGVGFPPEEFLYRYVTLSHGFILKAQDYTIWKAIPIFVLSKLATALWNFQDLIIILISMGLSSRYNRLNLYVRHVVSVEKQFESKQRFGTELYLQIQVWRRLREAYVRQSTLVRMVDRNLGSLVLLSNINNLYFICLQIYLGIHKSSGSTISRCYFLFSLGWLIFRACSVVLAASDVHLHSQRALKSLHACPSAAYNVEIKRLQYQLAHDFVALTGMGFFSLRRELLLEVAAAILKYELVLIQYDK

>HarmGR5

MQNGWNNVISNISVGSVNTVNYLFRTWERLAPNRNMDLYSLEKFKKYKNDWNYPVHVRYQDQVMAEKEKPRMTFQTAMKVTLTIGQCFGLNPVQGIREKDASKLRFKLLSGRCLFTFFSLIGQFIMAFVLFLSLFKETSSTVDTASNFGFTTTILFFRIATNWPKLCMHIAKVESVDPNTDNKLGKKFNIACISILFLALMEHLFSELHGISIALDCFPDTPVYESFMKLSFQWLFGFIPYSDFAGGMAHFSNLQCTFNWNFADVFVICMSMYLTARLEQVNQRIIAAKDKNSPSSFWRTMREDYNRSVHLVRQVDKIIGGVVFMSFASNLFFVCSQLLHTLAGGIKASQRCKPEIGADRRFFYGYEHSIYFVFSFSFLVIRSLAVSLTASKVHAASLEPAYSLYDVSSANYCVEVERFLDQIHGDTVALSGLQFFHVKRGLVLTIAGTIVTYELVLMQFTGITPTTSPESVSGVIK

>HarmGR6

MGQTSFRRNMSFWIPVKKNKVDVAKPKVKNITSFQDALRVTVIIGQVFSLLPFVGVFTNVASNVKFVKTSWKCVYSLLSLVGQMFMAVLCINKLAKTTVSLNGTSPVIFYVTTCVTMMLFFQVARRWPALVQHISKAEDMDPNFDCSLTRKCNITCAVVLILALCEHILSLLSAFAGASACYSGMDTYEGFVTHFYPWVFSYLPYSIVLGVITQFLHFQSTFIWNFSDLFVICMSYYLTSRLEQVNRKLLAAQGKYLPEIFWRATREDYCRATQIVRKVDEVISGVVFISFANNLFFICLQLFNTLEDGLKGTGECTPKLKKIVVSKSGPLGGHEAAAYFLFSLVYLLSRSVAVSLIASQVNSASSVPAPVLYDVPSPVYCVEVQRFLDQVNGDKVALSGLQFFSVTRGLLLTVAGTIVTYELVMFQFNSSTPTLNITSPTVVTHTITTLAT

>HarmGR7

MSSRGFGQFLRDGNLILPEQPNHDDFLTVMEKVFKWSCLIGVLGSKRHINYAWSGFILLVLLFMESQAIWKVIKALAGWAIDTAGQRSVTARLAGTIFYTIAILSLVLSSRLYRSWGQLSALWARVERIMAVKAPPDKTLKRRMYFFLGFMTVCSLLEHIMSVVSAIGLDCPPALIIKRYVLISHGFMILRHEYSDWYALPLIFMSTLASLLWNFQDVLIVLISMGLTSRYSRLNQCLAKICALERKQMDSDKKNEATKVYAWRKLREAYVKQAMLVRKVDDAIGSIIILSCFCNFYFICLQLFLGITQSKASEPIKTAYYFLSLGWICFRVLCVVLAASDINVHSRLGLKYIYTHDSHSYNIEMGRLQDQLSKDYVALSGKGFFYLSKSILLQMAGAIITYELMLIQFDDQGTDDVQLNLTKNAIGV

>HarmGR8

MSSKEFKQFLRQNKLLLPQQPFHDDFLDVIEKVFHWSCFYGVFGSKRSISLIWSTLILGSLVIIEVLAIWKVIRALAGVARDMSGHRSVTARLAGTIFYSISILSLVLISKLYYNWRTNIAGLWGKVERSVGVKIPVDKTLKCRMSFVAGLMTFCSFFEHALSILASVGFDCPPSLILKRYVLVSHGFIFMGQDYSEWFAMPLVIISTIATLLWNFQDQLIVLISMGLTSRYRRLNECLAKFCELEKQHMDSDKKVEAVKVYTWRKIREAYVKQAMLVRKIDVALGGIIILSCSCNFYFICLQMFLGITQGMSTDFLTGVYYMVSLAWLCIRVLSVVLAASGVNTHSKLALNHLYTYETHCYNVEVERLQDQLTKDYIALSGMGFFYLNKTILLQMAGAIITYELVLIQFDDQGNDGIALNATNI

>HarmGR9

MGVESAKVEEVTAAPVPSESGARPSRPTHCVVGGAHAFILRISSFFGLAPLRFESRSNGFTVSISGAMCVYSYILVTVLVICTIFGLVAEINVGVELSVRMSSRMSQVVSTCDVLVVVATAGAGVYGAPRRMRNMLKFMENIASVDTSIGGQYSLVTERKLCGIILAILIFFSILIADDFTFYALQAKKLDREWDVVTNYLGFYLLWFVVLILELQFAFTALSVRARFSAVNDALALTARQVSIPVEKPKSSSPLNIYAIRVAPVDSQRSANVSLLVDTMTGREHVVIIKRTASGEPRLVVSPCDAVRRLAALHGTLCDVVNSIDDSYGLPLVVILISTLLHLIVTPYFLIMEIIVSTNRIHFLVLQFLWCVTHMLRMIVVVEPGHYTIAEGKRTEGLVCRLMTSAPSTGVLPSRLEIFSRQLMLQSVSYAPMGMCTLHRPLIASVIGAVTTYLVILIQFQRYDN

>HarmGR10

MEYGLDAKISKELESINFRVLQETQLDDEAKKRSTKPWIEEDNKLVGKRRIDVKDQFTAFQKAMKVLLVWGQTIGLNPVTGILQKDPSKMRFTVYTWKFLFSLTVAVAQTIGTTLCIYKLFREPTSISALGFVTFFTSTCFTTFLFILIASKWPTLMQDIVRSKLDEYVDKKIITKCRITCCIFIGMALMEHFLSILSRVARVIECSQNETDHGEVFVKVTSPWLYDLNVPYVVAVAVMVQYVNLITTANWNYSYIFIVCVSMYLSSILNQINKRIALEAQKTHVPAKIWINLREDYTRATHLVKRFDDVISGIVLVTYANDLFFICLQLYNVLSNMSKAAQLVNKLCPDQDGTFRAYSYPAYLIYSVLYLLVRFLTVSIVASGVNTASLLPAPILYGIPTTAYTKEVERFQNQVNGDVVALSGLHFFYITRDLVLTLQN

>HarmGR11

MPSKLFLKTFNMATRHRLDKREICGLHSTVRGTLFCSRVMGLLPVSGLTCPTSRRLRFTFRSPYTVLYVASLFGQLLMFVMTLCWLMMNGISLANITNAVFYTSSLISSLILLHIGRCWPALVGSVETLERELPPFHRNVASISNVTTIFILTAAIVEHLLSVFYGLKVACACDSNNVAENYFRFNMPWIFDYTPFTIWKGALSELFNIQSTFVWSLNDLLIMVISIYLTEHLLIHNELLKKAAEQEHFSCLEFRTQYLKIVRLVKLINGQFGIYILTSFGSNLYWICTQLFYSLSRTQTGHFITCTFKDSPTKVPPANEYENPLCPWMLGEKGALNGVEHSIYFTYSFSFLLLRTLLVLLLAARIHSNSVAPLYVLYGIPSSRFHIEVERFIAQINNLKVAMSGLDFFYVTRTMILTLLGTIVTYELVLLQFNR

>HarmGR12

MKVHQLTMRRSNKRCGLHVCLRHAMRLARWTGFFPLQGLGQAYADGTRYKILSLYFIYNFTTLLGQLVMSCFAILLFFQTEVTLNSISNVIFYVTSLISAVLFLKLAKQWPRLMARATETEQGLTELKLPNKVIVKCCVIAYVAMALALVEHILCTSFNLTFVMHCLKEAGITTNVMENYVVHRMPYVFNYVPYSLFWAFLFEYLCLQSTFVWSFNDVLITCFSIYITAYFRSLNQVVTANSKKDKDNMIPWSTLRVHYSKLVRLVKEIDNHISSFILLAFFTDLFYICLQLFNSLHRNYASFKFCNELQTKQALTSPSYLLYYLYSFIFLVLRATMLSLFASNVHCAALEPVHAVYDVPSTLYDNEVRRFQLQLHHTKVGLTGKFFYVTRNMVLKVIGTIITYEIVLLQYTITPNPYYNGTKVILNISSHSYS

>HarmGR13

MSAFRDTECVVTGTLSMMLRMSQIAGVAPLSFRRTHGGWYIRTSRAANCYGKALSFCLWFLSSFTIAIDILIQPERSFRTRTNSTRIVWLADVATVAIVVCAAAFTGISRMRCLTVYALKLEEINLRLSLFHEEPSNEANRRLIAVSSMIFVVSTILVDYSIFIYQVITEHGKIVTSCMYIFYNISTIVQQVILVTFSETVTSVLTSLQMLNNCLKNLLQEILDSSELTNCALNYDSYINMNPNRSAIPNKSINSVVDTMAVYKGYRKNSIKAVPSTIRRLALLYCSICDVIRLVNDSHGLILVALMLCLLLHLVITPYHVITNIFNKERSDRSSPLLQLNWAVLHFVNLLLIVEPCHRTHEEMEQTRHLISQMIRYTPSEHGVLLTELQMFYQHLILNEVSYAPLKMFSLNRSLIVTVI

>HarmGR14

MRKLNLTPLKYLLIIENLTCVFRNYLCLRKCTRCLVTIWVVFETGHMFFNVYYNIVHGQNAELKAQRIYFFSSTAFSIYVMTSSLYFSKRFYKLLSNFDEFYNIFEDDVYNKKMKRAQKVMIWMVVCFIGIKFIVFYIMQVKTEDLENGLVSNTVSEYSVTVNDFRYIFQYFILDCILLIVAEQLRAISRSIDSELSAMMENQRNVGHVEGLPRVVLNYEKINKWAQAYESINESTHLCNSMFSVQLTIMLLIVTAYYIILLYSIAIITVEGVHTVKTLVMNLFSMSVFLLALLVISRAGQKIQNSSQQLRQRLCELCVHTLGNEEYYKLAKDLLRCVRTRPVRIHVFGTLDVNMSMLPSIVVLFTSYTVIALQFNNVL

>HarmGR15

MELVKLEMFIKAIMIFRIVCGLYCKITSNKIIAAIIKIYCALVIVIVFVISLSYLRALKNSASSAFAFGMPASKYFVNSVVHFCFDGDNFYEFFHSLKNLQITQDSELKYDIPITIFLFISITGARLKNYSRYYWLGMSDGHFRFDRFMSFCFGVLISYIGSITCVMRFMMFELLWRRMAMLRKRLEQDLLNARRFENEEGVLRKQLRACLSIYKGILDSTRKNDAPMKLLVTHLYIIFLNNVFQY

>HarmGR16

MAFVKSETFIKVILIYRILSGYYWKISSNKLVSVLLRIYCLFIATAFLCSMYQFYLFVSIPGKIHMCCICYLFSINVATNLIFNGDNFVYFLQEIRNVNIPRHIGCGDKIPITTVLIILTIFLRIGSRIQFDDHTHIDIMQGVFSIQNFSLTFDFLYYSSYLTRIMMFELLWQRITMLRKCLEQDLSIARRFEDGEQLLRNNLRACLKIFRSLVNTTRVCDAPMKLLVRDSVLY

>HarmGR17

MALVKPVALIKLVMFIRLISGFYCKISSNSKINFLVKTYCILIATLIIFLTITVAFVRVNIESKCHIGFMSTLYIISVVTSICLNGDNFEDFLTKIRGITTLPNVESADKITFSIFLFFLSLCSRIVVNVKFTIDNVQSISDPLFYVSLVSLTLATLQYSASFTRIMMFELLCRRMVILRKRVESDLSIPMTYQAKEMVREKIRKCLHTYKSLLDTIRGTDMPMKFLVSFLLKNLIEINRMQRVYKPGVHAPQIPSV

>HarmGR18

MFIKAVMIIQIFCGLYNKISSNRIICAITKIYCALVIAAIVIIAVIHIFFFNIFIVAKSSVVISVTTYVLYAMVHFYFNGDHFNEFFYSLKHLQTTHGSESKYDISITIFLLISISSARLYGYLKFTFMHISILPIDEFTGLFLDLLVCNISHTTCVIRYLMFELLWRRMAILRKRLEQDLLNARRFENEEGVLRRQLRSCLSIYNEILDTTRKNGGPMKLLVIHLYIIFLKIVPLKLTNITVVLLVPQTLNHMFGFLLKNAIGSELTVVDMYILFFETLSPALLAEMVQSEIECMKLSIVKQLLVCKDERTLNAIQDATTYLEQNPFKYTIWRVFAVDMSLILNMIALLTTYTVAMVQFAHFYD

>HarmGR19

MASVKLETFIKSVIFLRLFCGLYYKLSSNKITVALTKIYCTFVAKIIITIFVLFIKNLKVPFQIYLSFGLMCSGYLTNVVFSAYFDGDNFMKYFSALKEIRNPQDFPSFSDIKISIILLFFFASSRIFNYAAYSVSAMFTFQNPFPYNIYTTTAFIGSIAVLHSLVNSTRMMTFELLWRRMAILRKRLEQDLLKARRFENEGDILKNNLKQFLNTYKSILDTIRISEKPMKLGVTVVTNNKSSYLNTNHIAVNILR

>HarmGR20

MVFLKQEILIQILLVYRLICGFYFRISANKIVAPLFKVYCIFLASIVIAKTVWLIVNNKLDTLGTFHMCSACSLYVSHVAINLFFNGDHFIDFLEKIKNINEEDTCAPRDKILISAFILFITTLVRFYSYVQLEYDFFKRFFGSLDSVFTFSLLVDVFQYSSTITRTMMFELLWRRMGNLRKRLEQDLSIARRFDEGDEVMKEKLRLSLHAYKNLMKITFETCTPMKLEVIYMRFVIKNLKLSFILKRLYRRF

>HarmGR21

MEATSVFGFHRSVLLSANSITMAFVKPETLFQVIMVFRAILGIYHKISPNKLINAFLRVYCVIVAIVVHLGTIYIMQFFNLTEVHLIILNLFYSVDAVMGLCLDIDYLKGFLQKVRNMDTEHFEMKLKAPVTVFFIFYILIARFYCHTKFRYDNGESFFSPLFSEASLGLIFDLVRYSSIWIRILMFELLWHQMAMLRKHFKTELSVARRFEEGDHMREKLRSCMTSYKNLLNASHKFDPVVKTQVRV

>HarmGR22

MADIKPNQMIKAVMILKIICGFFCKISSNKIVITLARVYCLFFGLVCMSMLIILANHAKSFSALLQVFFGVYVYLIYFLLNFYFEGENFMGFLHKIRKVVTSQHMKPCEGVQISIRLFTISLAANILLRLKITLDQSHYTFSSTYSFLAFVSTINLLEFSPILTRLMTFELLWRRMAMVRRNLERELSTHQGYENSECILKEKLRRCLNVYKCLLEVTRVNETPMKIAVYNQKLKYARRSQRLSLIS

>HarmGR23

MVARLMVGFYWKTSSNKLVNALVKTYCVTIATVFNIMSITYITNEDEHIPTANTIYMAFVICLYDIIVVVNLFYTGEYLEDFFDKMENPAIAQDMEAGDKLIVTTAVIIFSLTTKFITGSIYIYEKFTLTNTSKNFILLIMQIINPISNFTRIMVFELLWRRMAMLRKSLQQDLTSARMFEGEEIFKTKLRTSLIKYQLILDTLKKIKHPIQFLVIVSFMNYVKSSYHMPDKKKLFSVVILFSDFVKYSSLYSTCTASYVWFYCIWRS

>HarmGR24

MVARLMVGFYWKTSSNKLVNASVKTYCVIIAIVLNILSINFITSEDEHIPTINLINMAFVICLYDIIVIVNLCYAGEYLEDFFDKLENPAVPQDMEAGDKLIVTTAVIIFCLTTRFISCSKYIYETYIALNNTAIDIHCLLLIMQIINPISNFTRIMVFELLWRRMAMLRKSLQQDLTTARIFREEEILKMKLRTFLMKYQLILDTVNKIKHPIQFLVIVSLMNYVKFSIHMRDKKKLLSVVYLIVFRFFQVQ

>HarmGR25

MVARLMAGFYWKTSSNKLVTALVKTYCVTIATAFNIMSITFITSEDEHIPLLNQFYLAFVLCLYDIIVVVNLRYAGEYIEDFLDKMENPAVPQDTEAGDKFVVSIAVIMFCLTTRFMFYSKLLYKSLIDPSDLSISRNFILLIMQMIHPVSNFTQIMMFELIFRRMAMLRQSLQQDVTNTRIFGGEEILKMKLRTYLMKYQLILDTINKTKQPVKYLVIV

>HarmGR26

MDVQATPASIKTIMFIRLLCGFYCDISANKIVTVLVRVYCVAIITLVMAFGIYLWNGIIGISSKIHFLFITTPYITSMVTNICFHGEYFSEFLNKMENFNLTHGFLSSIKIPISSLFFVFVFLQRFLFQMKFTFDAIGLPFRGVLTHASFILILCLMNYTAEFSIHIMFELLWHRMGMLRKRLEQDISTARILRDGEESIRENIRTCMRRYQHLLETARVTDGPVKFLVDTYIFITAR

>HarmGR27

MYSVNAIIIYRFIMGFYTEFSSNKIINFISKIVCVLLNIFITTKLFSLIDYNIVSSFIAFAGYSIIYLTNALASLFNSKWIQTYVNDLKSIGNAMGTGADIDIPIIRIILLCLHGLSSTTQIVTCEHEFCSGFYQKINGFVYSLATAMATILHMVVFELLFHKVRANRKYLENQLTIFKRHQNIMDLKNNLRLCMSNHVKLIDSLDKTDKSLKTTFFVADLAVIPIIMSRVFEIVKSPDRSALVNILLIGKGVVLRSMPGILADLTSREIDKMKHIIGQNLLVYEDDDVKYLLKDTHLFFEHQPFQYTVWRLFSINTAMNVVAFKMVVSYTLAMIQFAHFFG

>HarmGR28

MPSFRESGLESFFTLKGILIMRSIFGYYYKFSNSVPISVLLKLYCIACCIIIWCHIFVWSDVNSRAAFYDVTLLIEITINMLISLFVEDEFSTLNDLIASLPTKQNLRLTYAVIICTIIEQVTAFTYGRSLFTAEDAALYMFQYISCTYGRLSLIYQAHNNEKAIKTLCDSLKDKIEDMNMDAAEKRGHVEKFIDTFKQIINFSFDAKCQVMKFKVAVLGYVCDFFRVQFMVYYIYEFHLKIQVTLMLPWYCGVAYSIFIICLPSMLGELANYHLDEIKVVLVDEVIRNEGTYYFVIILGLRFD

>HarmGR29

MPSFRESRLESFFTLKGILIMRSIFGYYYKFSNSVPISLLLKLYCIACCIIIWCHIFVWSEVNSRAVFYDVTLLIEITINMLISLFVEDEFSTLNDLIASLPTKQNLRLTYAVIICTIIEQVTAFTYGRSLFTAEDAALYMFQYISCTYSRLSLIYQAHNNEKAIKTLCDSLKDKIEDMNMDAAEKRGHVEKFIDTFKQIINFSFDAKCQVMKFKVIQFISTSHIFVKFKIMQ

>HarmGR30

MAYLQKNLHIVKFLEPLMSVKTIMLIRISGGFYHKISSNKLISCITALYCLAITAFLSVNLLTLNYPSYFILVKNYVGFLIYYLLVGICLLTDSGYFQNFLNEIKKIDHMLGASSNIKVPISSFLLLGIITGVTGMDVLYVIYEVENNRQEKGFFITLFCMSLLVQTCAIANYTNVMAFELLWYRMRIFKQFLENTLKREFRSQDEEIKINAVRNCMVLYQRILDTVNKNNLPMKLLTFVLVTKFIPKTVATLYDIVTNAIPEVSYMLIHEFFVDFMVLCAPAIFADLICGEISSIKSMFKKQLLTCQGTFTNCYLVLAKKRSQ

>HarmGR31

MDKSLYKLLLFRFFFGHYFKLSSSKRICFIAKIFCLFTVIYISVIFVKFFLLFGSVISSAINLWILLIEAILSIFLSLHTEEAYVLKFSAKIKNYVSSSSTCRVTNVLAFLIIPINLFYLLAAYQYEFSITNNLLYNITFTVCYCSYLTSLYITEMFASAINNLTSDTVIKLKDVDITDEEKRFCIENFLDNYLKLMNIYNVTATVSRIKVSV

>HarmGR32

MPWLKGTEIESFFTVKGILLFRLIFGFYFKLSNSIIIDLLIKSYCFLCMILMWLHIIFVPNPENPAARVYDTLLAIEITLHIFLSLSKNEFGLDLIHSFDSAILQTKPRCLISYSMIFPVVFLEIYVAAFIINWNETKFINITMFYYQFVACYASRLAAIFQSENYAHATKTLYVQMKENFESNMNSAGKCEHVQQFNDKITITVDFFDKHRNVTRFKVKHLCISIFFFYIKK

>HarmGR33

MTDDTLVCDIKNVLFIRFALGFLQNFNGTSKTRWLSYLYTICFLLLFAVLSLFPNEVIYVSYRILALIEYFFLFMISFLSKEEYIYESYKLIYGLDTIPGAKLIFQNLEYCLKVYFFVSVFTGSFFTGISICFRIQEACSITNSFAVILTILDRTARDIGAYSLIMFIGLLYSRVKLLRNYLDTKSANTAWDRYSVKQYINMYESLTNTIDDSAVPVKVTVCFSCTLLL

>HarmGR34

MTDDTLVCDIKNVLFIRFAFGFRQNFNGSSKIKRLSYLYTIFFSLLFTALTLFSNDLSYHSLSYLILALTEYFVLFTVSFLTKDEYIQRNFKLIYGLDTLPGAKKIFQNLEYFLKVSFVLGLANILFFATMICFRISGLCSIANLLSFFYILLHRLACDLGDYVLIMFIGLLYSRVKLLRNYLVTKSANTAWDRYSVKQFINMYESLANTIHDSAAPVKVTVCFSMYSSSLELSIN

>HarmGR35

MMIDSLVSSLQYIMFLRFIFGFRLDCNSSPQMRLFTKLYPVLFFIVLNFAVWSSENVLNPTLRYSTVIEYCAQFFVSFLAKQEFVNKNYQFIYGIDSLPGAHKNFKRLRVFIKFFCLYYILLRLLAILQLVVYVGPLLKNVTADIIYFHVCDIGRLNIFLVFCILFCRVKTFEMNFQTITDVVVLDKYSVKKYINMYQTLIDYVESMDMLFKLMVIFFIRYNDRFR

>HarmGR36

MIGDSLVCNIKYVIWLRFAFGYLPNFHGSPKMRAFSYFYTIFLFISFTTIVIAPFYKFPWFFRVLALLEYTTHFLLAFVTKDDYLYQSFRFIYGIDTNANVRKLYRNLEVFFKFIILYFLANKILVVMMLCYRLPSICLFSNTLDFSVNIIIRLACDMGRFTVILSIGLLYVRSKILKMNFLTQSPNTICGRHSVRNFINMYESLINTFDKIKTPTNITVCFVITY

>HarmGR37

MIKNNLVVNIKPIMIFRSLFGYRQKFNSPLKSNFFDVFSLISFFMWSLSFFFKTYYFPLVYVMSDNIEYCICFFVAFFTKEEYIYFFYKRQSSIDNLPGAEKLFSRLNLILRAFVVYCFLTKIVVIIILRVFAPEIFQLGLYLDIVMGLSQSMSCDMGRFTIILMVGLMYCRMKIIKDNMDMIGSDLRNRFVARNFIQMYQSLVCSLQRIDVPLKVSVSPLFSINQK

>HarmGR38

MTDDTLVCDIKIVLFIRFAFGFLQNFNGSSKIRRLSYIYSIFFLLLLTALLLAHNELVALSYRIMALIEYLILFMISFLTKEEYIHQYYKLIHGLDTYPGAKKIFQNLENFLKVSFVLGLTNNLLCASFICFRYPKTCSIATPFFFVPIILHRLACDVGGYTLIMFISLLYSRVKLLRTYFDTKPANTAWDRYSVKQYINMYESLTNTIDISAVPVKVTVCFSMCSPSLVLSIN

>HarmGR39

MNSNKKVCNLHHILYFRLIFGYYFKQPTLKLRILTKLYIALFISGLTWLFITKVNGMLYYLQYCDILEYITFIIYSLITEDSSLLRSYEEKIKIDSLPVAKQYFRQLEKFLYLLLFLICGLRLFASSLFCIYAFEICKMAPFGVSVTNTFLTAMDWRHLNTVIWFSLLQTRVKILKNTLEIQGFDRGPMQRFSPRMYIKMYEELVGFSEFNGYAMKSIVRITSH

>HarmGR40

MTDDALVCDIKNVLFIRFAFGFLQNFNGSSKTRRLSYLYTICFLVFFAVLSLFPNEVIYLSYRVLALIEYFILFMISFLTKEEYIHQSYKLIYGLDTIPGAKKIFQNLEYFLKVYFFVSVLTGSFVIGIVIYFRIQDTSSITNSLIITILDRMTRDIGAYSLIMFIGLLYSRVKLLRTYLDTKSTNTAWDRYSIKQYINMYERLSNTIDSAVPVKVTVCFLMHYPSLELSIN

>HarmGR41

MILKYLYKINSLLKIIYSVNGIIITRFTLGFYTEFCSNKKIILLSKIYVVLTILNVSVNHIYTKEYQNISATLSLSFFAIIYFMNSTASLIIDSKWIQKYVNDLKSIRQSMVADKCFDIPISRIIMIIYQAFTLMMILVYCHYDICRHHIYLRVIEGYYAVTSVTTIVPTLVVLELMFYAIRENRKCLEKNLSKFNTSGSVDDLKKNFRNCMSNYTKLRDCLKSTDAPVKTMVSMNEV

>HarmGR42

MTDDTLVCDIKNVLFIRFAFGFLQNFNGSKIKRLSYLYTICFFILFAVFSLFPNELIFLSFRILALIEYFILFMISFVTKEEYIRQSYKVIYGLDTIPGAKKIFQNLEHFLKVSFVLSWTNNLCFSSLICFKIPEACSISYLYSVSPLLLHRLTCDAGRYTLIMFLGLLHSRAKLLRNYLDTKSANTAWDRYSVKQYINMYESFTNTIDISAVPVKLTVSYSMFSPSLALSITLSR

>HarmGR43

MCILYGFSYIESEDPLYTIIFTFVDYMSCDLGRFSLFIIFGLFYCRTKGFRLNMEGEGTDIPWDSYTVKKYMNSYQALYNSLFIAYKIIITVVRFKAYLFVARLFLQVG

>HarmGR46

MVKQFINKLKTQFQYYFGLNAVLFFKLIFGSYYDFSSSVILRRFAKAYCIFVIIAYWFVIYFIWTVNSKISLAFFLIVMTVDAAANILFSFITEEKYVIEFSSMVLFESGLNDRNIYFYLQIFHLVIVISSYLKRYYSIYAYAVAFVNISAYNNRIISFYVMNTFKDAVRSLRLSLSKHFKNKNLTSDQKLLQINKFLNAYMKLLRIIDKVFKIIRFKVSFCTICDIIFLPASNFLKCFVCPVLDCLCIDSRLLQNIRYIIPWISICISGKCYFALFIYLYAYR

>HarmGR47

MFQQIMIKIKNKIRSFYSLNSILFVRLLFGLYYKLSESIIIRLFAKIYCILYLILYLIFVKIYNTTDTDNFTFEFYGSVILIETVTNILFSLFSGELYVMKFLSSLPESDLYSFGVPLNLIICHSIKVILEYLFVPIPVFIGWVYLSVHMTIYNSRMTTVCVMDMLRQAYKSVSGTLIRNIMRRNVTDEEKITEIKIFVKGVMKLGHNMSIDINITRFKVRF

>HarmGR48

MLQQLLINLKSQFRSYPGLNAILIYRFIFGLYYDLSSSAILCLFARLYCILILIATVYTINFVKAISPLMSVTFYAMTKIINAIVNMLLSLITAEKHEIEFSSKVLSETGLRHRNFYRNLIFCYLAVNVSVCIEHSEAPTTYPISFLNITALNSRATSGYILQIFKDTVRSYRLSLINNFKNKQLTSQEKKYELNKFLNSYMKLIINLERVLKIARFKVRQLNGNFFITGLGK

>HarmGR49

MIAKIKYILKQRSPWILKLVTFLCLFYGQYLKISESKIICGFLRVYCIFISSVIIWGFYISYTKNVIYYGFAIIEYVFTIVIYFILYNDSIFKFYNNLEIYDRIMGFKEIPHFACYMIMFLIVNAIFRPITGFFRSTYIFSTWIQRISVGGALCIIEMNMFGIAHFFSLFHQRMQLMRKFLESNSVPVNITGMDEVAVSVRNVKKSLYYYDKLLDCLQSLDIQVQIMVIVFIIFVF

>HarmGR50

MEMLSKLKLLLSSCVDHFLPILRLGFRCYSRISDSKHVCYIAKLYTVIIFIFSTSGFLYWHRSTVISILLATQYCVHAIYSFVTSDKQAFRFHRSVKTSDAIMGFKNLPYISKPVLVPIYITVLLWMFYVYYFWIDDLHLSTYIMTICSDLNTVTVGILLIGVFSRMPIMKIALENNFVPVNIVGKDQLQKNVKIVRKCVGYYSNLLDSFDVIHTQFQFTVSDFMKCASCHYIVQ

>HarmGR51

MQEVKKLTKTQISAMWLVIFFTSARAFLGCYKKISESKLICFLFRIYCIFISFTLVAYYFRDNIDTGSSSHMLVIIEYIIIVIFHMFTGDTYMRTFVNAIKMNDRIMGFKGIPTFTKYVYLLMFLGTVSKTVAGSFRYMYSLATLLRCLTIGAILVSVDFNQLTIIISFSMLHYRMKVLRRFIDSNSVPVNITGRDKVAISIRNTKMSLFYYNNLLDSLQLINKELQFLVKNKFCY

>HarmGR52

MITKIKYILKQRSTSKWIIKLATYLCLLFGLYSEISESKIICGFLRAYCIFISSLTVWAFCVTNTTNFISYVFVIVKYVSAVVVFIILHNDSFIKFNNNLEINDRIMGFKEMPYFSIHGFMFFMCNSIIRTATGFSRNTYIFSSWFQKLSIGGALFIIEINMFGFYYFFSIFYERIKLLRKFLGSNVVSVNITGRDEVAVSIRNVKKGLYYYDKLLDGLQCINMQIQMMVNAFNIFVFDCTLCYYLYYTIAIIAT

>HarmGR53

MISAIKKYVKNDKLFMWLTKSYIFMRLLLGVYVKISESKLICVFIRFYCICIFFVFASIFTYNPENYSVLFEAAEYVFLVIYHFICENNSFVFIDVIKINDRVMGFKETPLVNKYLSFYMVLNSVLRYTTGQLRYTYHFVSWNEHISLGIALISVDISMWGSIAILTMIHDRIKILRKFIQSNKVSVNITGRDEVFISLRNIKKSLHYYDKLLDSLDLVNNQLQFQVNVLFRIVHIKYIC

>HarmGR54

MPQYSPTAILITIQYSVTVIYGYITSEKHAIRFFTHVITSDAIMGFKNLPFVRNAVLVPLYIFYLIWGFALYYRWVDLIRLPTIIMLLATDFNALCTGILFFGLYERMQLIQRTLENNFVPVNIVGQDQLQKNVKIVRKCVGYYSNLLDSFDAVETQLQVMVSGLMKVIEQIRCSSLHKIATYEGVVLFLKIYSVIFLGSICVALPRAVLVAGI

>HarmGR55

MDASKKLKFILEVSIDIFLPLTRNLLGYYTKLVDSKYVCYFCKFYCVFVNFYLVTQTYYLHGFFSVRDLIVFQYSVGAIISFLKADVCGVVFFKHLKTSDAIMGFENVSFGKRSVIAPLYFLYIARQIMVYIIWFDAMSFPVYFMCAASDLNSLLISIYLISIFGRMKLLKKTLENNLVPVNIVGKEQLDKNVNIVRKCVGYYTNMIDAWNIIDNDMQIMLAVALLTNTPIWIMNFYAVVMLFLQPTGQQEAVKLVQGNILSLLMATSPTIVSELISNEIDAIKATLVTQLVRCSDPSLSSELEVALHYIHIPPFKFVICRAVPVDINMPITIIGFCITYVIVFMQFLHFSNL

>HarmGR56

MIVKRNNFIQNRQPSLWLYKFITILRCLLGNYREVSKWKPICYLFKLYCIFICSVIVVSFYLSHYSSNFTNYIVILEYVAIVFFHFVCGDNYIMSFFSDVKINDRIMGFEEIRLPNYVCYVGFAGLLLRSTVSIIRIPIVGNSLRYYSITSAILSTDINQLTVIVIFSIIQDRIKTVSTFIASNSVPVNITGRDEVAISMRNVKKSMLYYNNLLDSLQHINKQLQFLVTTFLTILKIFGNLVGFIPCN

>HarmGR58

MKVNPATVKKQESKPNMEQLEESEYNDKIAKVMRSMKTITILEYSYGLFKFQFTNGQLQPINILIKMTAFLSIAAYIFMLYINVCLTNGTFFLGSYTVIQFVPSMVVLLQYIISTFKNFSISESLLNIRIFTTIAKLDSLLRVEVLNDFYEILRSKTNLIMLIFVILHAANFVLEFLTSNKTLWLIFISYHLHMTQEIELLAFFNCVSMITYRIQLINKFLTFFNNGQEQRDLTVFIVREKNNEAQEKLNFVGRVSETNVKLRDLATMYDIIGKICLMINKAYNLKLFMILVTAFTFILVTIWQALSFYQSPQYCTQDFVKLSLWCFSTVCNLSALAFVCERLLRARNKTRILVNKIIMNYDLPKTMRVQAKAFMELVEAWPLRIYIYDMFSLDITLLLKFISVATTYLIVIIQIFHFI

>HarmGR59

MEAEYDNKNKYFTRMTLTKSHEKQIIETLNIFIIFEYFFGIFRFERVNEELREPNWKKKVLSFCITSTCAISFVAYSVITLNIVMRATMREAIYINLTCITTMLLQFCSSALSSTFFFNFTNIRIITTLANLDVMLQIEDYKNFYKKCLFTTYKYVTVVFITQVTLSIIDGFTMYLGWAVPAAFLDFSQRLTILTFCKYVDLLRRRLKIINNYLKAFTDECEKETITVFTLRSRTNKTTQAINFIGHASDNNTKIRDLSRMYGMIGQACSMVNKIFNFLILTILFNSFIFIITIMWISLVMYRNSSDNLGLYINVVLSFFCWISYLLFITITCQRLVSLRNKTKILLNKIVMNYDLPTTMRDQAKAFMQLVEAFPLRIHVYDMFSIDISLMLKFISVATTYLIVVIQIFNFF

>HarmGR60

MKEKIKGKLNSSPEKFNIRSVKRIVDTINVIIKAEYFLGIFRFTIVHGSLREPNWKLKILSALIVLICSTLFLLLSESYYGLSSHLYRNFTDSTVINLSYFVMMAMQYSIHAITITFCFNASNICIINMLANMDTMLKAKILNNFYKKSLFQSYIYVLLVVSTQFSITTLACCTLNTSWSLIAGILDIVQRLEIVIFCRYIDLLRQRLGVINNYIKKFVSEQEKPSALVFTIRNRTIETTETINFIGEASESNDKIRDLAKIYGTIGHTSSMVNKMFNFQILTVIMSTFIFIIAIMWTCLKFYRNNYSNTGYLLNLILSTMFWISYIALMSITCERLILLRKETKSLVNKIVMNYDLPTTMRDQAKAFMQLVEAWPLRIHIYDMFSVDITLILKFISVATTYLIVVIQIIKFF

>HarmGR61

MQRFVMKDNNEVKAKEPSESNMEKQEENKYNDKLAKIMYTKKPVTILEYCFGVIKFYFRNGQMHAPNKIIKGTYVFFVVLYTFVLFEYFYEPIQTNDLNSKIVLAALPGIIVFIQYIASSIKNSSNSYPKLNIEIITTFAKLDTLLQVEDINNYYNISRSKVNLMVFLFIVFHSVNLLLEIMTYTYQIWPPIIWFHLFVTQKMEIVNFLHIVYMSTYRLDVINKMLRTFILENRQKDVTVFIIRKKIKNTQTKNFIGSASEDNEKIRDLSAIYNIIGNNCSLTNKVYNFNLLMSLVTAFVFILVAIWQTLSLYQSSSQYDMKDIMKVTLWCCNTVFNLAALAVVCEKLLRTRNKTRILVNKIIMNYDMPTTMRDQAKAFMELVEAWPLRFFVYDMFSIDITLILNFISVATTYLIVIIQISHFI

>HarmGR62

MENNMTEEKNQENKQNMEYSEENECKIKIQKVMYTVKPVTVLEYCFAMFKYNFCDGQLQPTKTGMKIYSSLCIIVYALVFFRFFFMPAVGYPYITLVPPICAFIHYVISSFIAFFLSGSKAYICIFTTIAKLDQLLQVDIVKDFYKKSRSRTNILVFIAVSIHALNCFLEIIGDLKEYVMTLTSFHLFFTQRIELASFFNCVAMVTDRLNVVNKFLDTFVTEQDKKDITVFIVKERKKESKETLNFIGRASENNVKIRDLAAIYDILGRTCMMINKAYNFSLLMILTNSLAFILITFWQALSFYQSQEMDSSDLIYMAVWCMSTISNLIALAFGCERLLRARNKTRILVNKIVMDYDLPRSMRVQAKAFMELVEVWPLHIYIYDMFSVDITLILKFISVATTYLIVIIQISHLI

>HarmGR63pMEFQLEEKIPSYRNTNKRRGEEQIFNTLKIIIKIEYFVGIFRFTLLNEKLSRPNWRMKSISIFIITISVVWFFSFAAYNLELPSVDDVTSYKFMNLICIIFMFLQFFASASTTFTFNTSNICIISMLAKVDTMLKVEILSNFYKKCSLNTYIYLTFVIATQILISIIDILTVRISWAITAGILDFVQRLEIAAFCSYVDLLKCRLNIINRLLKTFVDDQEKKATAALTIEFRSGIIENFSFIGQFRENNTKIRDLAKIYVMIGQICSKVNEIFNMQILTILIPLYL

>HarmGR64

MEFQLEEKIPSYRNRNKRRGEEQIFNTLKIIIKIEYFVGIFRFALLNEKLGRPNWRMKSISIFIITINVVWFFSFAAYDLELPSVDNVTSYKFMNLICIIFMFLQFFVSASTTFTFNTSNICIISMLAKVDTMLKVENLSNFYKKSSLDTNIYLALVITTQILVNITDFLTVHISWAITAGFLDFVQRLEIAAFCIYVGLLKGRLNMVNHYLKTFVDDQGKKAATALTIEFRRGIIENFTFIGQVRENNTKIRDLAKIYVMIGQICSKVNEIFNMQILTILMNTFITMIDMMWTCLVMYRAPTSPKLGVLINVTLSCCTWISFIAMMSIRCERFLYVRNETKILVNKIIMNYDLPTTMRDQAKAFMQLVEAWPLSIHIYDMFSVDISLTLSFISVATTYLIVLIQVIKFF

>HarmGR65

MEFGKDQDNESNIDQDYSNQRKKIVNIYNNIKRELIFEYFTGIYRFQLIDGELRTPNWKLKALGIVIVSIYTAAFIWFIIPDPSDCMTGLHVFLNNIDDFPCIVVLIQYIASMITCNFLMNSKNIRSITLLGEVDTILQVEKIEDFYKKIESKLNKYLILLILTHFIHGVLDVFSSDDIVWEMTILPLYLNQKIVVLIFCSYVIMLNSRLRLINSYLREFIQEQDKRSVPVFTVRGTKTKNEKTLNYIGRPSIRNTKIRDLATTYDIMGEICFMVNEIFNFQIFISLVTTFTFIVITIWTSLNVYRKPDYQSSQLINVLIWCMNMICNVAAMSFACERLLVLRNETRILVNKIIMNYNLPKTVRVQAKAFMELVEAWPLRIYVYDMFSIDITLMLKFISVATTYLIVLIQISHFI

>HarmGR67

MANVKKVEPNPKVENKKCDQKRTMLAIINTIKPMLFVEYLYGIHRFYFIKGQLRPPNWIMRAYAVIHTGSFLVLFFAFLNFPAVFSGSMKIVEIMDEFPPIVVLIEIMSSTIIATFVVNTVNISIFIKLAAIDAKLQAESLSDFYKKSRFETYVLLLALSVSHFINSVIDFVTADDITIKGMIVLPLYFVQKLETFTFCKYMYMVRRRLTIVNDYLRAFVEEQEKNSANIFTVTNNKVEKKREVNFIGRASDTNTKIRDLATMYDIIGKICHMINKVFNFQIFMTLVSTFTYVVITIWTSLYYYRKPGSNLGELINTAVWCCSAIYTVGIMSVSCERLLLVRNETRVLVNKVIMNYDLPKTMRVQAKAFMELVEAWPLRIYIYDMFSVDITLMLKFISVATTYLIVIIQISHFV

>HarmGR68

MDDDKEQDNESNTVKDDVKTDENPKKNMVEIINGMKTELIFEYCCGIYRYQLVDGELRRANWKRKALGTLIILIYTIVFFWFLFMDPDDDDLHFFMSTIEELPSVVVLFQYVSSVIANNFIFNSKSIRVITLLAEVDTMLQVEKFADFYKKISFRLNKVVIFLIISHIVNTAMNFCSVQDVAWGITVLPLYFIQRVEIFIFCGAVYMLKCRVKIINNYLKEFIQKQDKKSVTVFTVGKAKPKPDTTLNYIGRPSIRNAKIRDLATAYDNIGEICSMITDVYNFQVFLTLVSTFSYIVITIWTSLNFYRKRDYRIRQLINVMIWCFNMIFNVAAMSFTCERLLVARTETRTLVNKVIMNYDLPKTMRVQAKAFMELVEAWPLRIYVYDMFSIDITLMLKFISVATTYLIVIIQISHFL

>HarmGR69

MEDTTNEDTNYAKEKEKLISESKKQILDTMHLINVIECINGIFRFSFVNNELLPPNRIMKMLTVFCILIYVIIFVFCFISLSPLSDDEFDVVALVIQFSITLDFLQYAACTVTATFLVNSNYIRIIDSLACLDTELEIGKLSNFYKLSRFETYKYIFLVVFTQGLRAIADWFSDASTILYTLNFLLSFIQNVEVMTFCKYNDMLRRRLKVINQYVQVFADEQEQDTATVFIVKPKNEQEKEELHFIGRPSDDNTKIRDLAKMYNAIGQICTMVNEVFNFLILAFLATTFTYIILNMWTCLHYYRIGLNDLGMLINITSSFFFWILYVVVISITCERLLLVRNDTKIQVNKIVMNYDLPKTMREQAKTFMELIEAWPLRIHVFDMFSVDISLMLKFISVATTYLIVVIQMFNLM

>HarmGR70

MEPIDKTEIRNRAAKILHTLKPITIFENVYGIFKFNLVNGDLRPPNWKLKTVAVIYMSIFCYLYVVKHYSDQFVESTTKWSREYIMKQVPAWFVLIQYVVSCLKASFPDKSKSDIHIIKTFAELDCLLHFETLNRFYAKSRSRTNILVLILLIYHLLNFVLDIFSDYDYPRVFLEAHIYLVQKLKIVGFFRLMYMVTHRLDIINGCLNKFILEQERANTTVFSITKRQKRMKDMFNFIGHPSESNVQIRNLALMYNIIGKQCCMINELHNFKFFMILLAAFGYVVVTIWTALSYYQTQQFNATGTVIIAIWCLSTICNLTAMAWACEALRRERRKTKISVNMIVMDYSLPKTMRVQAKAFMELIEAWPLRICIYDIFSIDITLLLKFISVSTTYLIVLIQIYHLI

>HarmGR71

MFVTIKKKPFNILGNNHNIIEALSRTNFFRRFSGISVFTLKLSADNRVIRGFSKFGFTCFLTWLSLYIYCTYRAHAEDQTVLRVLFSTKVQRYGDDYERISSTIYVIFAFWKIPFRLNINNGFMGMVVKVDKALEELGATVNYNFDALLALSMSISQTFLCVTRLLSVWLTLRHLGVPVPSEKMFQVILSDSLALIATAHFSFFLTVLRCRFRHMNKILQDIKNHKSWEHKLFIRGSMFSNPQKAVSLQDKFICEKIKACANIHAMLYQMTDLTNKVFGSILMVTILIYQTYTIWFMFSFMEATAAGLFHDVERYVVFCINVFWEIGYATFITFMVIYVSERAVYEVSVTHRRA

>HarmGR72

MSATMKVPVKNKYFSYPFKLDGCKNIVEALSRSNFFRRLGGISVFVLKVGSDNRVTREISTYGLLFYIVWYAIYVYCTYKAHYEDQTILRIIYSTKLQRYGDDYERISSTAFIIFAYYKIPFGVNINKVFISMTVDLDKALENLGENVDYRLDALLALVISISQIALSTTRFFSIWLTIYQLDVPIPLERMHQVICSDTLALIATAHYCFYVKVIKCRFQHMNKILEDIKNHKSWEYKLFTRGSMVANVQKAKGLEDKYICEKIKACGKIHSMLYKMIEANNKMFGSMIMLTVLVYISYITLYMFYFMEATAAGLFHHPDKYAVLFVYVLWEIGYASVTIYVIVYISEYAAYEVSRK

>HarmGR73

MKFDHNVSLKQLMWLKIIFFQFCNWSSSVYVSVFAKLYCISNYLFIFIYGSIKMSHFNQYSASIRLNYITSIVELNVDTLFSLIHGEEYVRRFVKMLEKDFPGLQKFYKYYVTIYLSLIGLVGFLIFFTMDIRGGDNILKPCLFFNRSVACYFCRISVIYVIEGYRNTVSILRKQLNTQLNMNNITEAQKTVFIKEFTHSFIKLTEHFDSAMTIIRPLTIFRFVIDFCKILNIIYYISFFDISSFVISWTFEVIIEMLSLCCPMLILESAADDLDEIRKIIAKELLTYEDYYLRSVIYESMEFVDGYTTGLNVWNQYPMNKDMVLAFIGLITSYVIALLQFSY

>HarmGR74

MNITESKTICYFMKLYCICFSAVVIANFYFIEVHEKNIYSLIFVSIVEYALISSLSLIYGFNLFLNFFTAIKTNDRIIGFERMSLITRYSYLIIFVNSVMRYVITVSFGSTYIHSLLEHFFVFLAAIALDLYYVTIIILFALIQNRMKCLRLFLASNSVPICITARNNVANSIRNVRKSLVYYNNLLDYLEGLGQQLQYMVHFLFIYFFVLLELVKDTISYLSLFFLLQIFVNWVCYTSRVIILLYSITVFYSTGVS

>HarmGR75

MITFQSFLSKDLTLRMLQIIAFLRLFYGNYVTISESRAICYLVKLYCIGVCSFIALFGFYAITQLYSFTIISFTLIDYFMTALIFVVFSDQPILHFFSGVQTQDRIIGFKKMSYINKYMYFILFCTSFMRLAFFIHRIITSYRSNLEFILVVYILTATDLNYLLLIIIFSVLHNRMNRLRLFLESNSIPINITGQNRIAISIQNVKKGLIYYDRLLDSLQSLDKILQCQLMVYSITQFLRISMMSYRLIQQFMVEYILLRSTINVLELIPTALFVFAPLIFVEATTYEVERIKTILTAQILRSSDECLRFELQTALQYVRLRPFRYTLCRAVPLDINLIFTTASLCITYVIVACQLVYFSK

>HarmGR76

MKPIQNFLFRDNATLSELLLRTIAYLRLFLGNYVHVTKSKGIRYFLKLYCICFFMSVMSIYVYTSVPFQFTVFSFTVFEYILTAIVFVVSDDKHIHDYFINIITNDRIMGFKKMPYMTTYVYLILFSSTITRFCLCIIRIHTNIHTVITFCAILISITAMDLHYLLVIFLFSILHTRMNMLSLYIESNSVSVNIIAHNEIAKSIRAVRKSLSYYDKLLDSFENLAKILQYELIIYWVTEFLRASIITYNGLQGLISSKGEHLVLQHSNSVMTLNLMEVIISVALISFPAFIVEATVYEVNRIKKTLTAQILKCSDECLRFELQTALQYVRLRPFRYTLCRAVPLDINLLFTTAALCITYVIVALQLTHFAA

>HarmGR77

MIPERQLPNQQIVSEWVLKIIAACRLLLGSYMNITESKPICYFMKLYCICFSAVVISNYFTEVYENHTFSVFYVFIVEYICMSFLSLIYDYKLFLDFFTTIKTNDRIIGFERMSLITHYTYMIIFVNSVMRFGITVAHGSTTTKSDLGHLCILLTFTALDLQYLTNIIIFALLQNRMKCLRLFLASNSVPICITARNNVANSIRNVRKSLVYYNNLLDYLEGLGQQIQSMHYTDLFLGTLTIGVVHSKVMIYSPALIVEVTIREVEKIKAILTTQILRTSDECLRFELQTALQYVRLRPFRYTLCRAVPLDINLLFTATAFCITYVIVALQLNHFAE

>HarmGR79

MNPKLLFQFKSPTLCLIQIIALLRLLLGNYINLGHSKLTRFLTKLYCVCVCSSIVSMYILSLSERALFPVYSFIIIDYTCAVILSIIFGYDRYPAFLAANMTNDRIIGFKINLSLTNYAILSIYGSAFLRICLIIAHFLSHPSSILAFCSITCAIAATDVNYIAVIIIFTMLHRRMKNLRTFLETNSIPINITGQDEIAISITNVRKSLLYYNNLLDNFQSLDELLQYAVSKYTIWRMFQITINWITNFSRTCVVVYFFVQHIIAQLYPSLVLISSPAIIVETIMYEVDRIKETLTTQILRCSDPKCYTSIGISLSSVCHQTSVSGSSCRRRCSTCACVRSDTRSAARCRSTSTCCSPPPHFALLMSL

>HarmGR80

MDRASTFRSKLNVKRGTKLNPIFTKNLHSEVLLDNFIEKDLQSLLKPLNIMYSVFICAKYSIRDNFITCNNFLYNFIGVFTTGLFLCISVYRICSTLSSKRIQYVIFEWNTIFNFMSYSLGLIMNYIINIRFSDKNIQLVVKIQSVLRVLKMNRKDLTCLLVYNWSGVLAINALIVGSIAYMMYIFFPHVDILDVIYFYSTIVFDVNIMYSLFVANFSRKALCSWINDVLQSGDDSDFYWNRMFNVYLNMLDIYTTLEVVFQYSVRFHYLEIFLNY

>HarmGR81

MHNLQPKKMLKMKKIIQKREKSFDQPNRIGFDYQLDKELHQIVNSFNFALNLCFSSKYYVQSNHIEARGMKYRLLTSCYTIIMGLLCIYRIVTADIRDALMSYSENCFLRFLSGLYYTDYLLGFILCYILDIVHSHNHIILVAIFKVIHRSIDCSKIVSRFIIWNWITLCTTICIDLFIYVMYYGFFARFSLIENISDCLCDVMFITFYINYIIAIRVIILLKVYLDEWILNIRNLSNGLDRDEVCLKLIRVYNDIMKAYDLYKSIYQLLVSSLMNHLFQKFSVAFLRECFIFADSNSRTGHIFAKFNVLLSDHHKPQEECTGRR

>HarmGR82

MSSITRYDVGTGNSCHSVNSRCPVNKVDKDLQSIFLPLNLLQILVLNPKFYIRKNLVKPNDCLQKLILMCGLVIFLSGYVYRVTEIILDDNLKRYGSINFLYYASYFDFVFYSSGFIMNVIIHCKQSSKMVTFVLIFQKIHVFLNTGSIKRSVIRNWTNVTVIFVFYILVMLFSSLSIYNQASWNFALNLLYLASLDSNIIYAISLMRLLVDKLELWSVRVLMTSTDGNDVTYRYQMFEAYKQILKCYDLCKDVFQQQVSQVVNLNAYSETYIDSVES

>HarmGR83

MYVRSVEDIPTLPINRLHINNMVDNDLQALLRPLNLVPRLLFCASYRIRHNFICPNSFLYNVLVVFYFVSFRCLALYTVIYLCIYIVDFHGGSKIFFTLFDCLDFTIFSIGFLINTYVCFKESDNNILLILKIQYVLRNLNVSRLCLKSLIASSWWSVILIHAFFIVSGTYYCYYFSELRITDVLTQYPTILFDVNVIYASLLIKLLEKTLRVWIEAVQKQNIDNSERERHLEMLFDVYFNIQGAYKIIDKTFQIQVNIYFHVLLIIKNVMTMLLIKNYESIKNIKYFVLVFISQTWLLKSIVVFIYLSVECERFYAAMRDVHDTSIMLMNSEQFAELDMRVYKNIHRARRSLFSKLDGCRLFQVDAELPLQLSRLISSYIIVCLQFAFL

>HarmGR84

MKFDHNVSLKQFMWLKIICFQFCNWSSSVYVSVFAKLYCISNYLFILIYGSMLMVYLGQYSASVYINYVTANTELNVDTLFSLIHGEEYVRRFVKMLEKDFPGLQKSYKHYVTIYLTLIMFIEISIFFIMDIGERQFLQSAMFFNKHVACYFCRISVIYIMEGYCNTVSILKKQLTTQLDTKNVTEAQKTVFIKEFTRSFMKLTEHLDSAMKIIRPLTIFRFIIDFCKILNIIYHVCFFDFSRFILSWCFEVIIEMFALCCPMLILEWAANDLDEIKRIIAKELLTYEDYNLRSAIYESMEFVDGYTMGLNVWNQYPMNKDMVLAFIGLITSYVIALLQFSY

>HarmGR86

MPFYTIFNSRIAARRRSLTTANQRHEIETNEGLGRENVVEKYLQRIFQPLDIMQAIFLSTKYKIRDNIITPIGRIYSFISISGEFGLMIVYYFLYIHTDIWTDNINHFIFGVVDFILYFCGVLLNPLVNVIQKLNNVLLVLKLQNIHRVLNMNENYFKHIIISNWIFSFTVSNFQLLWLCGYYYAFYNIGVDNILTVYICIRFDMNVVYATRTIKLLCKSLEKWTEDLWRSGYFEDSDDQYWDRMFEAFLDTIKAFHIIETIYQQTVGFLNSNHSDLKYGFLTFLSLSWLMKNLTLQTLLSVETERLYAAMREVQSSCILIPTLKQPSVYQRRFYKNIQRVQEISFKKMSMCRLVTVDAELPLRVLHTITTFTIVILQFEFL

>HarmGR87

MIFTNNKISAKTREPEVLLDNRLGKDIQRMLYPVNLILSLFLSSKYTIKDDYITPKGKKFYIATFFFILLLYGLGINRVFFEDIEDTMGTDNRDIVTIIFSFAFVFYSIGFTLIFVLNIIHSDCSISLILTLQKVFNSLDFSDKIVAITRWNWFAICIAFGTNVFLYMLYYVTYHDFNPVDLVMDIMFITFDINLVYGILVITWLRKILEKWIEDVLAFEDGDEEFYSEYFQVYRNILNAYNCYKTLFQLLVRIFSIFCFSKNLMYYFFLPVVYFLQVLFHTADTFFRCLCYFAIILQILQMPDSTVYEQMVQYTVVKVVAAVWQIKDVLLVVMQCLECEKFYMAVEDVETTCIQRLKKKHHLAAEERLCSSVLQANRTSYCKMSACGLFDIDATLPLDLIGLLTNYIVIMLQSFFL

>HarmGR88pMSEKRKIVPYPSPMLPNTEIDKEMQGIVKSFNFVLHLFFSSKYCLRRNNVYPRGTKYRFMTVLHTSFLNGLNVFRAYRDSSIGVTVNDFMRIMNCCYDFVQMYAFILLFILDFVHKQNHVLLILAIQTINRSFDLSKSIRSFIIWNWIVLFITLCLESYMHIVYYAIYAHSRFSDVIPDCICDFMFISFNVNYIFATRIIILLKIYLDEWVKFILILNEREENNEYCLKLLEIYENIMQAYNLAKTVFKNLISVSHTLDLFFWVTKTMLLSLVHCAYCERFYISVEEAECACIQLIKNINCPKSHKYLCKAVIRINRSFSKMTACGLFYIDASLAICFFGAVTNYAIVMLQFTF?

>HarmGR89

MLAYFKKKPKSKVNDVIIKTLPTVQYKEVLLNNVIEKDLQSVLKPLDLMQRLFICAKYCIQDNFITSNSRSYNILGITLAITLRSLLFYNLLRIVSSEDQNNYVLAFSNIFDDVFFSIGFILNYYSNIIQCNNHVLLVLKIQEVHRILRVNGKQLKCLIIINWVYVIVLNVVYILGALFYCLIVSFARILDPVSNYCSIAFDINIVYTAFVMSLLRKTLSIWIEDIRTSKHVAIESYWTVMFDVYSNILEAFKIFEK

>HarmGR90

MTESGIPLTNKLSKEVQQIFQPLYLMQMLVLNPRYQLSNNFIHPNMWLNKFILLTSLGLYVSFSLYRIFDIYLYFDLTMFLLGNYLQFASYFNLVFYCVGFIINFTNLFIHSKRYVAFVLIFQRIHTCIGNKASFKSAVNISRIYVSIFFGAYFLMSCFVITIFKQTSWVIVFNTAIMLTIDANMIYAITLIHLLTNEVKLWNSHISMFSKKGSDEDQHKIMFETYLHIAKSHRICRTVFQYMVRYFIILSRKCSNGIGLNSSVFLHVPRSYRIHLHFHGDLCLDSKSRGHTNSAGPQFRSLLPSYRRCTRFLRSHHDMVKQSYARTYCVSTVPVSAG

>HarmGR91

MITKITQMVKQPITEPDSSFRVLKDDRLDKELQHIVRPINFALHLFFSSKFDVRYNHIYPIGTKYRLLTFCYTMIMIIFCIYEIFTFDNLVEAAFDYQKILVKFLHIVYFTLYVVQFLTWFVLDNVQTQNIVSFILMIQVIHRGIGCSTEFRSFVIWNWISLFSVLCVNLIIHIIYYIFLDDLSNMTTFIAFLLDILYISLDVNYAVAVSFIRLIEKYLEIWTKEVLKMNAEKENGEQRRKLLKIYHNIMDVYDLYKTIFQYLVCLK

>HarmGR92

MLHIVRKLQTAIKKVKCASSYMNTDNLAVNVIEKYLQATLLPLNIMQELFFCAKYQIQNDFIYTNGLKYDIVSAIGTILYVSLSFYLISSSFNKIHNLLLDLVVFAIGSILNYFVNIMQKNNNVLLICSIQNAHRMLDINGMVFKRSLIFNWIYVIALNSFHIFWLFYYCYTFDDISIRDIFTSYLHICFDVNVVYAAKLLEINRKTAQIWIERIQQSVGNIVHCDSNNLFKAYLEILKSYQLIEKTFQYLVSLFVACN

>HarmGR93

MTVESCVKKNSITRPDQPVKALPGDFIDNDVKRMLRPLRLIHFFSFCPYYRLKGNLILPNSLCSKLLSFCVTMFFMFLFAYRCYDHRYIRQQRRNVAFHTINSYVLVLISCFGVLSNFISALQSKLNVKFVLKIQDVHAFLNDSKVFKRFIYRNWIFVISIICYEVFGWISVNTLLKLSYMDVLCGAASMSFNVNIVNATRLIVLLQDKLNLWNDRVLQLEGMESNSDTEDYCQKLYQKYIDIMECYDIHKLSFQMKVS

>HarmGR94

MLLENRLDKEVERIAFSFNLPLNLLLYSKYRLKYNRIYPNGIKYDIYALFCTLFLGVLCFYRIFTLDMTNASMSYMERAILTVVPILFFTIYFIGFVIVFVSDIVYKDNNVLLILTIQTIHRSISFSKTNHSFNMWTHISFATVILVNLITRGTFYLTCRYSHVSEEISDIVRDFSFVTTDVNMVIATRIIILLKQYIDLWIKAILTTNVAQATDLYCQKLFDVYMNILKAYKIYRKVFQALVSFDFYIFHLFI

>HarmGR95

MLSHLNKFWKPNKSATTLEHGTRESTENEPNSFVIEDDLQSILKPLNLMLGYFFCSKYSIRDKLITYNSYIYEYIRVLVVIIIYSWNFYNTILLNKKMLDQHWHGLICLGLGSMSGFFLSLIGDTIITWSNIIQKQCNILLVIKIQQILRVLQIYGNELRNVINWSWICVIVLNIFSMSYFICYCVTIEEINVIGIIISFASVTYDINVVFAFLLLKLCEKILRVWLEKIKLLKDNGDEASDEFWNRMLKVYLSLLDIYLMIESTFKHMASIS

>HarmGR96

MLTTKTMRQTQNNAEAVSCQILPKNRLEKEIQKIVCSFNFALRLFLVSNYCIKNNHINPNGKIYHTFAFFWMLFMSVLCVYRMFTLEEAVEHLEAAILVNLLFLYYAVYWLGFTVIFIQNLVNRNRIVSLILKIQTIFRSLGFLKDIQSYIIWNYVSLASIVCANIFVHVTFYISWSHIKFIDQIIDNVTNALFVSIHIYTIIAIRVIILLRKFLEEWMSVVNTMNVEHDNNELCLKLFESYKNILEAFNLFKEVFNLLVSNRNVYCL

>HarmGR97

MSQTQINVATVSFKILKNNRLDNEIQKIAYSFDFAVQLFLFSLYNIKNNHIDPNGKIYHTSGIIFMFLLNSLCFYRMHSIAGLRGKIEQFDLIIFLIFFYFGWYCVVFTMMYIQNILHKNYIVELILKIQAIHRNIGYKQSLHSFVIINWISIATIIISNIMCLITFCASGNYNDVIELVSDNICHFTYVAHHINVIVATRIII

>HarmGR98

MYQPNSRSDISHCKNLYNRLDDEILKIVYPFRCVLYLFFTIKYRIRNNRIYPSGKLYRIFAFCWMLFLNSLCILRISNVEVRNNGKTKQLEYSILLVLCTGFFVTYFIEFTLMFIFDMINEENNILLILRIQNIHRSIGSRKTIQRYITWNWISVAIIIFSDFAIRVLYYISSYYPHFMHSVYDAIIDAMFIALDVNIVITMRILVLLRVYLNEWINNIKTMKADDEEYRKMFQDYKNILLAYDLFKTVYQAFVGSVSTYLFNCCEY

>HarmGR99

MLKSQLVNSLNNFIDKDLQSMLLPLSLMQNFTFCPKFRIKNNRITPISYFHKFVAFVGTVMFIYFYVLRVYIQSFEKIFKNDFFELYTCCYCSFGIAINFIDSIIQTKLYVNFVLLIQKVHRLLNDEHHFRFYVISNWLRIIAVYGFFIIILIEVSVWIQMPYYYIASCYPMVAFDLNLVYAISLITLLKDKIILWDIHVSNLQAMQEENEPKKMYQTYVNILKCYEIYATCFERNVSGFFLVISLISLFLVLLPKLF

>HarmGR100

MLSSIKKTNLNKIALMVVKRPTVSSERRNKIEEDLESILKPLNIMQSLFICAKYSIRNRIITHNSKLYNALRVFCTCIYSCIYLYRMISQDHQTINKSWRKFWFGMAWIAGFILYMVGDLINTISNIKHSHKNILLVLKIQHVLSILRISGTDLRIFVCYSWASVIILNIFSISYVICYCFTTNIIIFEIISAYTSIAHDINIAYAIILMKLNEKMVRVFMEELESSKCDDSKIEEYWNRMLNMYLNILEIYNIIWKTFQQMLVLLPLMAQIWNAKNMVVLMCLSTECERFYTAMKDVETACTKIFKSRNCSVHLTRVCKNIQRGQNTYFKKMNACGFFYIDVHLPVMLSSFITSYTIVLLQFVYLD

>HarmGR101

MSPARFQKISNNRLERDMYRTAYSFNLPFIMLLASKYQFKYDRIYPNGKKYLLFNFVYMLFMNGLCIYQCYSVEMNSDRINFLGKIAIEISINFYIVTYFIGFTMMFITDFVFKNKYVLFILKIQSIYVEIGSRSIITSFITWNWIYLLLTIAINILLHGLFYSNYNERAFDVSIYIIRDFHLIVLDINVVLAIRIIVLLRKFIESWIQYISVKNDEADNALYCRELFGIYVKILNAYSLYEKLFQLMKPIIAILKVLLLVKDLGLIIVHSEQCEKFYMAVTKSESVCIQLIKNGHFTKYQKRLYKNVIRRNRVFSKMSACGLFDIDATLPIRFTEALTHYVIVLLQFNYL

>HarmGR102

MTETNDDSQTNNQLNMRPKYPKSKPCQVLPNNRLDKEVQMIANSYNIALFVFVSSKYYVKDNHIYSRERTFLLFKIFHILFTNTLCIYRMFTININTLGSMGHYEDECMKILNVVFYVTYFICYTFIFINDVVQQNNYVVLILKIQEIHGYIDCSEKIRSYVMFTYFGVIFTICIDSLIIVAFYMFFDTLYWFDIITTCYCDIMILSFNINYVISMRILDLLKLYLEEWTIEVLRNVGKNDDEQCIKLLKMYQTIIEAYNLHATIFEKLVG

>HarmGR104

MIGYHQFYRLRFFIYFRIFFGLGNFMFSPFQNFVLKLYSIVVAIIITAAGAIFIQYYGTDTFHRVVVLVEYFAYSLISFVAKGEDVRSFFQYLPSLDSFPGANREYRKMMNSVIFVLSFSIAWRITVTVIVLYLYSASLNTLVGMEIFFFIVITVAIDLGRASTFIYFSILYFRLKIFKTMLRSTDFNSTRNISVVYKFIQIYELLADKFRKIQKVLKLQVSIARQIV

>HarmGR105

MFEPQTVCNIEPLMYYRFILGFCQNFQHSRLTRILSKIYPIVLVVALIVKTFIFNDTATLYNKYFIGLEYSFNIVVSLVTTDKYINMYFKYYHTIDSISGAKKIYKNLEKLAIISVFYASSFRSWAFIVISRYNEAFFKGFDRSNIIELAVMYYVNDLSKITTLLCFTLLYFRTKVMKMALEATEFNDALRDRFAVNRLIQMYETIIDTFEIVAQPLKFTVRSIIMNRYYLGVQGRFRPRWLFHRKRSASWAGHIIVHEHVRRHKCTPYIPSLS

>HarmGR107

MNADLLKCFAPIHNVLLFLGSSRLKIKNNMIAPSTRYQKMYALCCIFIVTLSFSYIQLYYYLTYYHEDTTIYVCYAIGIMVQNVSYLSHTIFARFLDVESSVKLCQNLQKVDNILRLKQFKRYNEQQYYWNVVVLVFIITSFECGFLVHIWYTVEYPILAFFAGIGLLNVYMELVLAASLIVYLAIRLKFLNKIAHHNFKIKGYYNNTRAACAVDEHLLINSDVKDANIDLGNFLICMKEILKMYQHITQVFSFPVSRCTTRAKIIKVRASWRALSSRLLSF

>HarmGR108

MPRNIEELSEDLFTDDFIRIFQPILFVLRALGLARVSIKYRYPTGTSKWYLLYSNVFWLLNALSAVYFFFHCGESFDSKYADSTLKFGVLNSGINGILVVFRNNLERNNKFGAMYVKLQKIERHLNMEDTKSINKQLRSQSTIVMVVAFFVTIFWIVLFKYLFMKSMCIPLIVNVVTSIGLQTEMAQIYFIIKFIITRVNYINDMLRQVSLLSIEPLTKPMDDGILFVVSNTLKHQDGEVPGELVSGMHCIFETLSDFTGLFQFSLFYFICQILAWNLVTIHYLVTSMKEQGAADTDLLLCVMPVLVALQFIILTLCLKAQDLSTKLEEARKLCIDIISSPLINGKSREHAKQLTLLVEGRRSVSIYNICTFGTRLPLHLLAITASYTVVLLQLALL

>HarmGR109

MKNLKENIKIEYLSKDILDEEFMKSFSLLYYTQRLIGSTRVQIKHRFVTTPSFLQKFHTLISVVLLLGLDYLVIQKYDKILFDRETIYYLSICVTGLQTITFLCNIINVRFINGDANVELLVNLQQIDRRMNINRNKSITTLLVKTNVISLVVVLIMFITLLGVASAKGTAAFWPYTGIAYSQFSFVIELISCSNMFMYFYVRARFINSIIKNYIDQKGTQEILYSKERFLSSYFASKVFMRRLAAGSHNFVSSDTDVYLKQLLEGFFKFQDIYKFQVFMFCCKLVASALLTFEFLLYAVQNDTVGLWDSLTPSFFTVIDLVMAILLGVRCEVFIREVKETKRLVITVMSRHYDGRLREKSKRMLKLVEETPPHFSVYDMWQLDANVLLQMFMLVTGLIVTQMQFAFL

>HarmGR110

MISLKSVITIKTLMAIRLGISGLYFPITSNKIISLLLKIYCAIFTITVMYYILTCTYGLPFRYNLTSYSILTMYFANVIHTVFHNGDGEYLKNFFVAINKIDLAIGERPDDEIKISRLIFIVVFLVMRTTGMVIYCQSEYKKYCSLLRSLLLAGLFVSIAGQWCHTSYIMMFESVYHRMRLLRKRFENRLSASRQFEADEKVMENQLRQCLDIYKNLLGVTGLYGARIKIMVITQLFIYVHIFLAKMYILKKNLLIDLKIELIT

>HarmGR111

MSPARFQKISNNRLERDMYRTAYSFNLPFIMLLASKYQFKYDRIYPNGKKYLLFNFVYMLFMNGLCIYQCYSVEMNSDRINFLGKIAIEISINFYIVTYFIGFTMMFITDFVFKNKYVLFILKIQSIYVEIGSRSIITSFITWNWIYLLLTIAINILLHGLFYSNYNERAFDVSIYIIRDFHLIVLDINVVLAIRIIVLLRKFIESWIQYISVKNDEADNALYCRELFGIYVKILNAYSLYEKLFQLMKPIIAILKVLLLVKDLGLIIVHSEQCEKFYMAVTKSESVCIQLIKNGHFTKYQKRLYKNVIRRNRVFSKMSACGLFDIDATLPIRFTEALTHYVIVLLQFNYL

>HarmGR112

MLFNGLPNTNVSVNRSSKVVPITVKPTQRNNYVPSKTVSAIQTIKYQVCTALVFGINRLYLFKPNTFVLLLSYVYTIFLPILVWDIMFNADDLSATYFVFKYTCCIEYVLLISISVFTSRSKLVNLLRDLDKFDSLLNIRKDLKVIDSGYISVFWFCGCFIYSLCEYICCYFYLTVFIDRSVYCLYVMMLAHDCEQILFFVLLRTIYTRLRVIKAHVLKVFSAENRTNNYRRKLDKVEALSNNAQLDISSLHRVYDLLHKCAEQLNSIMSLSVSFEELFAN

>HarmGR113

MNLFYVLIFCENFMCVYRNYFDINKYQRIVAITRIIVELTLSVVITAHNVLLTKAVNYESTEKDVLLTMLFQILTLFKSIVIIIGGIMNSESFKQFYENLRKLYHCFENDVDYKMFEKKLRIKSLVGFSIFTFMSLVQMFGKIFQYYFLGTYQLTEIIVLVLYELWVDMRYSLEHVVVFCAISCISDFLKCLNISVNKVLMRFSEQPSLEPNSNEENVNLPEKVNNWTEKYQKIMACCKNISLCYQELVSNHHLPSLFMTRLGSASSLTACS

>HarmGR114

MSEIKLLTYLLHIENLLGIYRITNHHKNVKKYLITIQIFLISLLYTSVVVVEIDLNIRRVDGDDHEIDDIYLIFSSSWYINFLTSLASSVYYRSAFESYYESINRVYDWFRREKSNVTSMTKFQWCTLMFSSFTMFFNLFQPVEALFKYDFTYPLFVAYILLTVSFLKITLLFEHFVLFSIIILIVRVLKCLNHLVNAAEKRLRSQISDSECEIATKQIQEWASLYTDLANC

>HarmGR115

MTPIKLLNYLLFVENILGVHRKYNIQNKIKKYLIIFQIVIQTTFHAITLISEIYLLFKEEKWKNYIDINVIKSCYAVTAHINAISAVLTGICYSQSFLSYLDSITRVSDSFQDDRKLAKSLKKMYYLSISLMFLSISFVMYRVKEYLKRFSYMHPLIVVPVVVSQFFIRSTLIVQPLILFTIIMIVAHLFRSFNYFISVVNKRARSVDSLSEENDITREEIQNWVELYRDLENSCENVAIFFGHQYFFTLTMSISNSIMMVYHVGCSVLLKAITPDLRRMVIGLLNYVAWSMLPVISGQVVRNQAVKCHREFARLYNTVVIDPSEEKGKLIKDFIRVIKKKPLDIKLLSKLPIGMYMLPAMLTMGVNYAIMVLQINHII

>HarmGR116

MSSAKLFKYLLVFENILGIYRNYGNKNRKVKCLIIFQILVQTIYHIINVSGELYYLLQKRLSTQTFIELGFVLSANINASVTLVSGFLYSREFQKFHRTISLISERFKHEKSLKRSLKTLFYVTGIITGFLITSVILRAREVYVRHYSFSDALLVNFMFLPQLFTRLTLTYQLIITYVYVMVVVNLVKCFNSLISDGQRKVSRNTSVLVNCGCDVTKEQIQDWVELYQEFSNCCEDVTICHGWQASLFCICY

>HarmGR117

MADHLINLLTKTETIIGIYRNYDSLSKKQKILCKLRIFFEILFVFSIATYNVLVLENFLSTRIFHYMMMYHISNFLGGPIVAISGILCSNTYKNFIDNFMTMDMHYQKKSAYVKCLKKMKILFVVTCIISCLSIVFFLITKITARFFIHHHYVNIGFVLMLVVAVFVQQRFFLEHTLMYIFIRMIQNVLRCLNDCMLDAQVGYNDMTRSGQSDSREWRPLLTAEQVQLWAEHYKCLLICSKNLSICFRSQVI

>HarmGR119

MIEDKVIKTFVISENIMGVCRNFAALTTSQKIFSIIRIVVEISAYIIIYSLFFLDKCNHVLIEGGHFLSLMMIYHPVNFVCGSMILLCPVYNPCGNKLFIKEFTMVQCEFRHTPFYAQSMKRVKSYIITCITFFSIIVAIVLYTKVLLVFEWSTFGPQSLYIGLITIEIIFEIRQTVESVTIFSYITLLQYHLKTINSCIASVVAQYDPLEARSDSQTNNDHLTVDRVQYWADTYEKISNCSKLLSQCFSTQVNFGLNSILLFIY

>HarmGR120

MSLLNNLIKSENYMCIYRNFMLMKNYQKIIVLIRIFLELCATIIIITHKIFFRDLGNVVSSLLFVPYCYQALVLLKNLVIVVGSVLNSKSFLIVNEKTRVLHNRFRNEPSYSKSVKILNLKCSVISIAFLILVFIVIIIRIYIISFNKDRFNKGRVVIIILFEAWVDVRFMLEHLVIYTVITLIYDFLKCLNNYVYEDLKKYNIDTKDEEVHLDEQFNETADKLTVWTEVYQDILSCTKNTSICFNELVIKYEFI

>HarmGR121

MLTKIETILGVNRNYPSTSKTRKILFEIRIVLEVLYIIGFIYTYRTKIKFKDGQFYLMEIFHLANYLSGPIIMINGILTSQQYKRYLENFIPVHAYYIKESKYAEKMKKIKTIFIIVTSISFAISCAGFLVKYYNRYVQNQEITFLVTIFLLSALLVHYRFMLENGVMFTHMAMLRNLLKCLNDCILDAQVGYSYFVQSGRSDRNEWRPLLSEEKVQLWASQYMSLLNCSKNLSVCFRAQVREPQIQ

>HarmGR122

MSSVKLIKNLLFGENMLAIYRTYDCQNRLMKCFIIIHILILILLNTFIVSLELYYLFDLGEPDQMSVVFCGFSIASYVNTLSSIMSGIYFSSGFLSFMKSITFISESFKNDTIVIKSEKRLRWFSLLILTFPFGLFLIRLQEVLKKFKDLNLMILIPIVVSQTFTRMTLLYQPIVFFIVISTVVIYFKCLNRLISIATDSLKICRLRSGLHGECDLGRDQIEGWVELYRDLANCCEKVSICFGRQFSFSLVLTMSNYILLLYQICYMNTYHIPNDFEFKKIILVIISYIIMTMLPIFAGQLICNQELKCHRVLSRLYNTMLIYSNEAEVKLVKDFIRLMKKYPLDIKLMNKLPAGMYMVPAILSFAVNYTIVMLQFHHVI

>HarmGR124

MIKTFLLAENIMCVYRNYVSFSGYKKVLILIRITLEIIWMLITILIFVWQVSEQFSTRNHFLYLMGVYHLTNALCGIALVICGVQYSESYKLFLNNFEVIRHEFQNSPAYTKNIKKLKRNLTILIVFFAFVSIIDFFFKLRANMITWRQNHIKITFLTLLFTLVYIEIRKILVNSIHYANITILRSTLESLTDRVSEIREKLSKPGMSDRSYKLTVAIVDEWAVNYQKVLISSKLLSECAGCQVICTFFLICVLFYLYLCCLKSICFYFGYCLLLFFYPVHNVV

>HarmGR125

MVLYHSSNLLCGSMIILCAICCAGSHKLFINNLKIVQREFWYTPTYIKGMKKLKIYIAVTTTFFSLAVASVLYSKCRNATIWMTPDIILCYIALLVIEIYVEIRQILESIIIFSYISMLQYSLKCLNVDVLGTRKQYNRLGIFLYVELKSSNFLNVQVEEWAAKYQNILVCSKLLSDCFSNQVSWYNSIKITLQTKQLADW

>HarmGR126

MLFTSKFSIAYNCIRPHRFFYYVFSFIGVLTFILYHLQRLFNGNFATYNYLSLAATLNVFFVVIPFPIFYTLNVLQREDNVEIILKIQNALKIINYKRYMIRTYWNWFYIIRHLIAYLIIVCITRNSQIAIYYYTLNYVDVNVIYGVIMIELIRDGVIIWVSEVEHYSKLCLDLNEETYNKTMKKLFHAYISLMEAFDIFKGLFQYSVSFYVISWQICFTRDHLAPVGLHSLSEG

>HarmGR127

MSGAESSVVIRIIIAVRLLCGFYTKVSSNKIVDALVRAYCVIFSMTVFSILCTVFSRVPLIKVTFMITFSLIYACNVILDHCFYGDKFFEFVRKMRHISISQDSRLRHVKLPITMFLTILLFCSRLFSHLKYMLENKYVIHMISMQSLGLAYDLIQLVSVLPRILMFELIWRQMIILRKQVQQDLSSIRRFEAGEELLKNKLKVFSNAYKNLLNSTREIDSASKLLVRNAILIEIAIKFVVIISPALLAEMVNSEIDKMKLCIVKQLLVCHGDSTRDAIEDVMLYLKQNPFKYTIWRLFTLDMTMLYLKQNPFKYTIWRLFTLDMTLILSFIGVFTTYTVALVQFTHFFV

>HarmGR128

MSITKTDIFTKIIIAIRLICGFYYKISTNKVVNALVRAYCVSIATTVIVIIIYEWFISLDRSAVKILVFSSTLYSLNIFVDFCSNRENFMLFVKYIRQPISQDMQLVVNTPITAILLISTLCLRVFSHIKYQYDNGIWFAFISEETLMLVLDILQYVSISQKMMMFELLWRKMAALRNHLERDLSSARRYETREDLLKNKLKACVDIYNNILNSTREIDAQTKFLVIN

>HarmGR129

MIQKNYVDKDLQSMLLPLNLMQNIMFYPKYSIFNNCIVPNSVLSKVVALCSTMAFVLIHLYRSYNLYYNQMIREFVNILYITSYFDIILSCIGFAINFIVSIYQTENNVLFILKFQKVHRFLNDKNLFNRFVFMNWLILVLLFSFVIFIMSSFFLYMEVPLPDFFCGLAAFCFDINIIYALRLIKLLEDKVKLWNFEAQHLLQIYHSNIESHCQRMFEAYFNILECYNLYKYSFQLMVCMYGPESLFLRYFLFHHLRC

>HarmGR130

MLPSLENSLESPENVHTIESISPEASNEETVNNKIDKDLQSVLRPLNLMQAIVICSKYRISDNKIKPHSRLYNWLGFTLVIAFRILSIHKLLTSNYPSNVSRLVAFLYMINIFDFVFNAIGFFLNSYVNIIHRYNNVWLVLKLQHIHRILNINSKNLQMLIIYNWISAISIYIVFIMYMCYISLFFPVFSVFGTIVTFSTISFDINVIHALLLIKLLRQTLRMWILKLLDLKNTDTVSNDESLWTQMFDAYKNILDVYKMIEKLFKLMVSIFL

>HarmGR131

MKFYRKTETKTNVHLVFNNLLDKDVQSIFFPLNLMHYIVFCPKYTIKNNFIIPTSFIVKLISILGTLVFISVTLYRNYYLFFYQESVTISPFMYYSSYYDALFYSFGFSMNCLFGIFKSELIIRSIMTFQNIHRYLNNESNTRRNIILNWTYVIVTFVGYFSIYTYFYSQLSNSYNLTNAFFLVSFDINAVLAIRSLNLLEDKISLWNVSICKNQELENVNDRNYAKKMYQAYVNVLECYETLKTLSRSFVST

>HarmGR132

MRSHTRQTFRQHGIFFPFESILNNQLDKDVQSILFPLNLLQFIVLNPKCHIKNSFINPNNSFNKVILFFGMIIYVSAYIYRVLEITLDVNLRAYGTLSFLYIASYFDFAFYSTGFILNSIINFCKTKDMVNLILMYQDVNRFLKDKSNFRWSVIRSWIYVALIIGFYVFTMLFMSVAPFHIVFNLIILISLDSNIIYTIILLKLLTEKVVVWNSAILKVHKNGCSTSYCTKMFDVYVDIFSCYNLIKDMFQQPVSLLKFFSTTFLIYYYEASKIFRLCYFVFRFYTKL

>HarmGR133

MLLPLNLMQTIALYPKYSISNNVITPNSAISNVLSLCATMAATVTHFYEGFKLCYDADVVFKYIASNIQYFASFFDIFLTCIGFIFYFFICIFHSKNNVLFVLTFQKIHRFLNDEKISKRFICWNWITMAVVFIFDIVVLTYFNLRLHLPLYTFVCCLFAITFDVSIYYALRLMKLLSDTIKVWNMEAQNLVRLRHSNINQPNCQKMFKAYSQILECYNLFERSFQQIVCILMFYFFVLEPSLL

>HarmGR134

MAEKHYVDKDVQSMLLPLNLMQTIALYPKYSIWNNVITPNSAISNVLSLCATMAATIVHIYEVFELCYDADVVFKYIVSSIQYFASFFDIFLTCIGFNFYYFICIFHSKNNVSFVLKFQKVHRFLTDSKRKRFIFWNWITMASLIIFDVAVLIYIHVKLHFPLYNLFCCLMSISFDVSMNYALRLMKLLSDKIEVWNMEAQNLRLLHHSNSDMHCQNMFKAYVQILECYNLFKCSFQQIVCITDVFFCIF

>HarmGR135

MESARQKTTNKTKSCSVSIATIKTIDKDMQRMLLPLNLFHNILLCPKYRIKNNFINPNSFLSIILGLFGLILSIFSFCYRVYKYYRINPKKYVMNVMYVTSYIDFVIFSIGTIINFKINVLETVRNVSLVLKIQDIHRFLPGQNYFKCFTIWNWISIFSGVGFYTYMLIFTVLTFEMHIDGMLFGFTLLFIDVNIIYCIRLIKLINNKVDIWNRRALKMHQMDPIDNEDYCEKMFEAYMNILKCYDFYKDSFQLMVRNNLFHSSARYF

>HarmGR136

MCGKRYEMRNTRKRIICRKIRVHNYKNNQIDQDIKWMLFPLNLMQMITFFPKYSIRSNIIKPNSLILKFVSLTATILFISAFIHRHFTLLSRSNIHSSSLIYTYAATVAFCLGCIINFIYSVIKTRDQIIFVLTIQRIHRFLNNRNVYKHFVIWNWIYVIGLFIFYISAVTYFTIMLNLPIYSTYSSVILICHDVNLICAIRLMRLLQDKAVLWNDKIWFQENENGHHNNVCRRTFKVYVDILKSFNVYRTVFQIPVSKFMLNS

>HarmGR137

MEPKGRIKRIFRRYIDKDIQKMILPLQMMQTICLNPKFSLKNNFIKPNNIANNLLAVVGVIFFVSLLIYRICDMMLDENLRRYQTVNFLYFATCVDSFFYGCGFIMNFILHFVHTMNNVNIILIFQEIHRHINDKASSNMAVFRNYVIVSMVFAFQTAASIYVYIVYMHPPWYVVCYVLVLISLDSNIAYSVCFMKLIADKLVLWNAKLLWSLQHGSHVMRCKKMARAYVQILNCFDVYKNIFELPVSIR

>HarmGR138

MAINIEDISTQNNVVEKEVLSLFKPFRMMHALFVTAKYKIKDDVISANTLLYTCMSGFTSIVILVFYFFSIFQTAFVFKWEGLNLAKQVCNIFIYAIYLMGSMMNFCSDIINKDFNVLLVYKIQSICETLKIKGKSLKNFIMINWIYVITLNVYHMWWIVFFSYAFSSSYLYYEVVTNYFYIIFDMNVLYGMRVMKIIRQPLQIWLEEVRNLNSVIDEDYEYFWNKMFKIYEETLETYQIFAKIFRSVVSKVNWRLLIILAYEICLKLILRNYLSCSLIRTSTIYFRCSL

>HarmGR139

MVTVEVIPVNKNKQIIEKDVQSLFKPFNIMFALFFCSKYRIRNDVIHTNSLFYKVVSGICCLAIFIGYCISVFIKIFTIHLEGINYSKFCYNITVCALFFSGYTLIYYTHVIESNRNVLLMYKIQNIYKIVKTRGVFVKNFIKYNWIGVAVVALYQLLWILFFTIAFASNYEYYEVIANYVYVIFDLTGLYCVRIMRIIREPLRLWLDDVKNVQHVDHEGKASFWNKMLRIYLETLDAYQVAARTIQPGVSLIFNY

>HarmGR140

MKLKVVAMEASIVAKNNIIDEDLQSVLKPLNFMQAVFFLSKYSIRNNLIKPNSLIYDLISVTCLLMFRIVSIYRIIIFSFASKWTPLLQFLYVSQILDSIFYSVGFLLNNYINIVYSKINIGLVLKLQFVHKVLNINRHKLRPLIIYNWIFAISVYSYFIIFNLYMWLKFPNPSYYALILVFSALAFDINIVYALLLIKLLTQMLRIWLVEIQELTNVGMSGSDESYWNKMFDVYKNILEAYKTVEELFKLLVRFYFCE

>HarmGR141

MRSTSRKIWNRIRIILSSEEFSNNKLDKDVQSIFFPLNLMQLVVLGQKCRIKNNRINPNHCFNKVILFCGMVTYLMTYMNRFLEIMLDENFRTYVKNPFLFLSTYFDHFFYMSGFILNFIISVTKTKDVVNLILTYQKVNRILKDESSFKGTVTRSWIYVGAIFAFYLYTLFFSLLASFNVLFNTVVVITLDANLIYTMVVIKLLTEKVKLWNARIITDSENDCNNKMFDVYVDLLKCYDLLKNVFQQSVSQATPF

>HarmGR142

MKIIIINNYKKINFVDEDIRSMLLPLNLAMLCPKYSIKGNLIAPNTFRNNCVSIVITLVLISAMCYRTYGLSFYQDGFSNIVYYYSYYDVCYYSFGYIMNYIISVYQTEQNISLVLTLQKLHRLFNDAAAFKRFIIFNWIFVITALVTHLLLVTSACLDMLYHSKVNLIGYLLVLFDIYIIYCFRLMKFMEDKVHLWKSKLESSEEFDVCKFCEIMFESYVDILKCYDMIKDCFQRFVSMILFFNVSNIENSCWSA

>HarmGR143

MAVTIEEIQNIIDKDLQSLLRPLNLMYILFGCAKYKIHDNKISPNSVIYNTISSITAIFIFCISFYFMIGTFSLNFNGYIYINHLGKIYTYILLIVGCLSDLYTNIFQKSNYISFVMNIQNIYRSLNISGIFRSYIFPNWVSVIALNCFHFTWMFYTFYAFQSLDHSFVFASYYCIVFDMNIVYAIRIMRLINKSLKYWLEDVEMSGRFVTESYWNKMFETYIEILKTYQIIESTFQRTVCLSV?

>HarmGR144

MVKIDSKPSFEKSELSSNNVIEKDVQALLKPFNVIFALFISSKYRIQNDVIYQNTLLYKILSGISYTFLIAGYFYSIFRTAFIYKWEGINFTKQWCNVFIYAIYFLCCVINYHTNIVCSKINVVLVFKIQNISEILKVKGISLNDFIKFNWVYFTILNVYHVFWIVFFTIAFSDTYEYYEFLTNYVYILFDMSVLHGTRFLKVLRQPLKLWIREMRNSDSVLDEDNEYFWNNMFRIYEEILDTYQILTKTIQPVVSY

>HarmGR145

MVLSRRNSMSLIETNQGDVEILSNNFIDKKLERLFFPLNLMQNLVLNPKYIIKQNRIKPNDVFNKFKIFLSMVIFLAVFAYRLCEVIFDENLRRYGSVKFLYFEIYSECFVYCTRSVVNCIVNLVQSKNFVAFVLTYQEIHRILTYEHMIKFYIIRNWVYFSIVFGYYIIVLVLIPLIFERWAFHFDINVFTYIILDANLIYTIALLKHLNDKVKQWNIEVVRSPHRICSERMFQVYVQIFECYEIYKNVVQENVS?

>HarmGR146

MELITNNYLDRGFQNMLLPFRCLNHLVFISRFSIEYNCIRPHSRSYYIISFMGVLCYIIFHSLKFFDANLTAIPNQFIQFFLKVNIIMLLIPYAGFFILNVLHRNKHVQILLKMQKAFRIINYKRYKLAILWNWFGVFRHIGGFIITTAYIRLLSVAEYFYTLIFFDVHITYAISLITLIRDGVITWIAELERHSQNLEVDKDKHDERMKKLFQAYINLMEAYEIFKKLFQVAVRILSF

>HarmGR147

MFPCFKHEGQSDPSSEDVVHEDLQSVLKPLNLMQAPFFLSKYTIRNNCIKPNSIIYNLMAVISMLIFRIVNVYKIVVFPFVTKVNSSVTLFLYVSQILDTVFYTVGFVLNNYLNIVYSRINIGLVLKLQFVHRVLNINRRNLKSLIIYNWIFVVSLYSYFIFIGIFSWITYPFITLYSYILVVSALSFDMNIVYALRLIKLLTQLLRFWLMEIQELRNLGVCRSDESYWMKMFDVFKNIVEAYKTIQDLFSLTV

>HarmGR148

MPKKNRIVEVIVRKDGIKNPLIKDIKRIFLPFNLALNLFLNSKYEIRNNNIYPNGPKYNIFASFFLILMNALCVYRMFTFDVADNSSIEEDLTKAILGFLGTSFYFVTLIGFTITFISNTLHRENVVLLILMIQTIYRSIDISKSINSYIIRNWICLVIVIISDFTERLMYHVTCHYHVLFEQAFDIITDIMPLVLDINIMLFNRILVLLRIYLEEWIKIVETTNDDDEEQWVRFYKIYTNILNAFNLNAKVFEWLVRLIPYLMQNSIIFLSEYLYMS

>HarmGR149

MIDNTNSNYIDKNLQTMLFPLNLMQNMMFFPKYSIYNNNIRSNSLLSNFVSLCTTIAVISLHLYRSYKLYSDNIIREFINILYITSYFDIILTCIGFTINFIVSVYQSKNNVLFILKLQKVHTFLNGEQQFKRFMYRNWMFLVFEFLYFTFGLCFFCVKLNLPKYDYFCSLTALCFDVNLVYAIRMIKLLSDKVELWNIEAQKLLQLNYVDIESHCQKMFDAYVHILECYDLFQCSYQQLVRIFFSLSYLFNKVFFVLKIFTIFYRSCSTAFNSFFTSSFISKLRLTF

>HarmGR150

MNTVDKDVQSMLLPLNLMQYLTFCPKYRIKDNFIIPNSRVSYFISAIASLIFMFILETFYYQILKSPDFDEEPAYLIACTTYDTLFYGFGYTLNIMDSVIRSKKNIQFILTFQRVHRLVNIEKCFKNFVVWNWIIITLFLTLQTLLITVFCLLSDFFDATVGYFYVLAIFDLSIVYAMRVLKLLENTTVLWIQVLNSHQFGNLYDCKKLFQAYVDILQCYDMFKSCFQHFVSFYQAKCFFHVMF

>HarmGR152

MVLTFKTVCDKRNKKIKVKPRINMPTEAKLNNEIDKDLQSVLKPLNLIQGLFIMAKYKISDNRIQKDTLLYNLLSIICLIIYRLVNFYKITISSLNRDWEGTRFFIYMSNINDTIFYTFGFVLNNCINIFYSDSNILLVLKLQQVHRILKINSKHLNDLISFYWRFIISFFISHLLFEMYFIFQFPVYTMYGVLLSFAILTTDINIIYAYFLMKLLNKTLRVWIEEIQKLRNFVTFSINDSYWIEMFNAFEYILKVYNFIRKVFKLMVSYCFIIIMTETFIIIVLYSFFTFTIVEIMSFPCHF

>HarmGR153

MTPDTSVSLRNDKINHSFAIHNFVDKDVQTMLWPLNLIENILLCPKYCIKNNIIKFNSLTCILVSVIGFIICESLRLYRIYNLHFDYFTRNFNNIKYIMAYVDFVLFSFGFFIVYFVNIFHMKYNVLFVLKFQNIHRFLNEKKYFMRYIIFYRISVVIIPIFFTGVILYSFLRHSVTVMDCICAISLICFDTNYVYAIRMMKLLKSKVDLWNIQIGQLQKLDQDEKVICCNKMLEAYKNILDCLDLYKTVFQPLVSLNFFCILYHY

>HarmGR154

MKMKIICNTIKKYFSKPVMKKLIDKDLQSMLLPINLMQNILLSPQYRIKDNLIKTNTLTAILVSFCGVMISIFAFLLRICLTSEAIKQYYSSLYIVSKIELVLYSTGFIINYISVLRSNKNVLFILKVQDIHRFVNDGIYLKRLIVCNWISVILIFSFDFTIIIYAWVKLELRFYNLICGVSVICFDINFIYAIIFLKLLRNQAELWNIRLKNFSGQSNGESVCRSMFEAYDNILKCYEMYKDYFQQNVCNFNHQRVIFVY

>HarmGR155

MSVVEDGISNSNDRNVIGKDAQAWLKPWNLMDALFICSKFKIKDNVISSNSLFYNIMSITSCLVLVLIYFYCIFKDCFHIAWEGLLLAKYVHYCFEYLVYVIGVIIAYFFNIKNRHSNVVFALKIQRICEIFKIHGKSLKSLIVLNWVLVIVLNSYQVFWVFFFYYAFSRYGFPIEELVPNYFNIQFDVNAVHTSRIMKLMWQTLRTWLEGLQNVYIVEDEDVEHYWRKIIAVYKEIIEAYDIFRKSFQVLVRE

>HarmGR156

MSNTLRSKVFQNHLHKDVQKMFYPFYYFFLLLLSPKYCIKDNYITPNSLKRNLVSFLGAFYVFVTSIVYACEEGYKDYYNESNVHSLAMMSFIYSLDISSFALGIMLVFVQNIIYSRKNILIILMFERIRQSIDISKSIRSMVIWNWVFGTLYFSIHAIVFIELHVLSQVNFLMQIGGFICNYMYAIFDVNSVYGLRIMKILTTYLNRWTEMVLKLNEGEENLISCVKLFDIYTNILKAFELFKDVFQVLVRSVDLIGSNYSACLFELERIIYFTGIIYNNQRVYS

>HarmGR157

MLKPCIFLRKTRSYPVEVLLNNRLDKEVESIFYPFHVILTLLCASKYCIRDHFITPNEYKFYTVNFISLSYVVASFAYQMYNNQLVHIHRSDNNIVVSFLSVFLPISRCICHILYFVLNIMHCQNNVFIIVLINIIYKSLNSFQKVRCHIINSWILLALILLIHAWLTITYIVIYEIYFDVIHHVSEVLLYIFDIDFVYNVRVLLMLTNYLNSWIENIKLFDDGQEYDKIHYIKMFQTYLNILRAYDVYKTVSQVLVRWQKSFTQIKCMSNLCFILGLPPSLTKCS

>HarmGR159

MCSVLNMYKYIRNKLRCAREAVLIDGVSPAAYKEVLFDNIIEEDLQSLLKPFNLMTALWIGKKYTIRDNFITFNSNVYNYIGITLCVAFLIARVSYRLIVEGIDLDSFAFSLLRTNAMLDLIIFLFGILLTYSTNIIHSYHNILLVLNVQNALRILKINRKDLRRLTVYNWTTIACIWAFFFVPHVFYSMGTKRFDIFENLGILFKISFDVNMMYIIFLVNLLEKILHEWIRNFEKSMFKTSDELYWKKMLYLYLNVQESYQIVETVFRTHVSLQYHYSKFLHTESV

>HarmGR160

MTPYSREVLLNNRLDKDVQRILFPFNFFLTMFLSSKYCIRDNYITPSKRKYYVFGLFGICIITAANVHQMYGQIANMDLNKRGLLILIFLHVTQIFNFALSIVLNIIDCHKNVLLIVIIQAIHRSFDFSKSIRNLVFYSWMILLIGLCINVYTIAYGYAILQSWHILSFIHDVLMVVLDIDLIYKIRLLILLTTYLNEWIKNICLKKDDWQQDQANCVNLFATYQNILKAYDVSNELSEIIVSYEVYL

>HarmGR161

MRSFKNIVNIYPKIINNKVDNDVQEMLYPLDFMQCLIFISKYHIRNNLIAPIGVITTFISMIATMAFVVVHIYQTLFATSETNSIASITTENITRYFSCVFYCSVFTINFIMCVIQTNKSIKFVLTYQKVHRFLKNSGSYSYNNNYIIWNWVFVITAVIWHTSTIVYCVVSIGYNFFVATCYMYPIMLFDINMVYAMRIIKLLENEINIWNDVIKSRLHTQDENYCRDLLNIYVEILECYEIFKDCFQQSVSISTNRLD

>HarmGR162

MKLKICRYCSVSTKKTSLTMNSVLDKDVQSMLLPLNLMQYLTFCPKYRTKDNFIISTSRVSNVISVIGTLVLILSLELYYYKIVFIDNISEKQYYRFMYSLIFDSIYLSFGFILNFMDGILGSKNNINFVLRLQGVHRFLTKGNNDKKRFKYFIICSWIIVISFVSVCFFSVPLTFIHTEFYIHIIYYLIILIFDINLIYATRIIKLLEHQVATWIQVFSCNELDYLCEETLLKKLFRAYVDILQCYDIYKLCFQHFVSIGRKSDYIF

>HarmGR163

MFQSLNNVKHKQKIPRNILNFPPEVCLNNYLEKEIQNIFRPFNYAFILLLSSKYTMQDNYITPNGKLRTFLSCMSASYVSGVGFYYMISNKYLEYNSSVYSITVVTIVVQLIIIGYCFGVILIMVNNFVFSQKNILLIVTIQTIGKNINLSKVVKNFIVGNWIAILIPSAIYAGIQGSFYIFYYIIDYTLVLATLCTIAFLAFELELVYALRVIVLLRKYLQGWVQMVSKLNYDQDDGYDCVKLFKIYQNILQAFELYKAVSQFLVIGLHLLSSQKSFLKRTLIL

>HarmGR164

MHILFKNILDKDVQCMLWPLNLMQYMMLCPKYQIKNNLITPNSLISNIISIIATVGFISSSFYRTYEIIYYSVLKSSFFMSFVLYYDCIYYVVGFIMNCAMGILQTKNMVKFVLIFQKIHRFLNDGSLFTRYVIMNWIYFIAALGFFFIILMLFVMLFENWIFIIYGYELIFFDLNVVYLIRIIKLLEDKVLLYNKYLLNCQNLTHDESYRQKMVQVYVDLLECYSILKKSFQQFVSNAFIFCFSD

>HarmGR165

MNTVNKNVQSMLLPLNLMQYLKFCPKYCIKNNFITPNSNVSKLISAIATLALILFLELCYYKLVFHDHYDKEKHYYLLASYTFDSIYFSFGLITNCLDGIMCSKNNIQFVLIVQRIHTFLNIKRNFNHFTAYNWLTVISYISLYFTLITIYCIHLNLASSTIFYYMFIIFYFNLIYAIRIIKLLENQVVLWIQGLNCTQLENTYDKNCYKNLFQAYVDILQCYDIFKSCFQHFVSILICKFSTDFNICIIIYFFGLQYLLYVTFQILYYISEVFIYSLINAEEAIILLKIGWVYLHQVRSFLESDMKVFIYNCICRNVKSLSYFCYRPKHGSYHYS

>HarmGR166

MHNIQILKAVKNNNNIVDKDIQSMLLPLNLMHYIMCCPRYHIKNNLIIPNGLISHCVSIIGTIVFIALLCYRTYVLSSEYTAMFDVLVYYYSYYDIVYYTFGLTMGCTLSIIQTKKNVEFVLIFQKVHRFLNDETSFKNLIVFNWIFFVAAIVCHFFIVSGFFSLLTYYSKFVWTAYLLVFLDFYIINVIRAIKLIEDKARLWSLNLLNKNIENMDVQNYCKRMFESYFNILKCYDIIKVCFQQFVSMIITWLYDNLIVAEL

>HarmGR167

MFNSHVAVTSCYPVEVLLNNHLDKEVQAIFYPFNFLLTISMSSKYCIRDNYITPTQRKFHILRFVCTVALLVIYDSVTFKVFAGENIIFYIARFLSVIRNLTFLQNVAVSILCNQDNVLLVVLIQMIHRSIDLSNKIRSFIAWNWIIIATISAFNIMVATAFSSGKDGFNFIGCFTDVIYTSFDVDFVYSIRVLNLLNKYLYEWIKSVRIMNEGKENDKMNCTKLLKTYENILRAYDVYKKVTQYLVSILRKGYIILCFSITKQKTM

>HarmGR168

MHNIFKIFQKKRNQTIILNPVHHDTRLDNEVLRIIYPFNFAFFLLLSSKYSIQDDRIMPIGMMRKCLSLFNVFYAGALSLFFIYFYIVTNDFSKSSIIMSIIHVSGTVTFSMGLTLIIVVNIVFENYNIQLIKMIQFINRGIDFSRSVKSFIIYNWVFVIFVFSIDLFTYIFFMVTYYMDVLGIVTLWARLMFISYDINRVYAIRIITLLRKYLDEWNKNVSQVNNEDGTRFMKLLEVYENILESFKLYKTIFQELVSIFNELNVYLL

>HarmGR169

MRSNCPPAVLLNNHLDEEVQRILQPFHTLLTVFLSSKYRIRDNHINPNGFIFEFSGFSGLCFAFGATVYRLLKNENSGFDSNVMTNIIHYFLPAMRLLGFVTNFVLTIIHRYNNVILVLHIQRIYRSIDFSKSVDSYVLGNRITVAIILVTNGVIFTVFITMYNGFDVLNVLFDIYFVTLDVDFIYAIRILILLVRFLEEWIKSNKLIEEQAIDGEYSSKLRESHRYILLAYEMYKTTTQLMVSQEGPQKVDR

>HarmGR170

MCSYFKEYFESCQELIIIKDISPEADFGNNIVDEDLQSILRPLNLMQSLFLSAKYCIRDKNITSTTRFYTFLRIIFVLVHRCFQAYQIIVWNTNMFNENSTSPNYYSMLYLCIASTVGFFIYFIGDVISIISNIVFRRSNILLVLKIQHVFSFLRLNRNEIRGFIVSSWVLVIVSNVMSLSFVVYYVFTFAEVHFITMFTAYASISFEINVLYAFILLKLTENMLREWIKKFEATRNIDDSEKLFQVFGIYWDTLEIYMIIEKTFQHMVWFDSTC

>HarmGR171

MKHNIIRYTLCITDMYPSLTMYLKQFRNFAKVENISAVTSEDRFQNNKVEEDLQSIFKPFNFMMNLFLCAKYSVRGKCFTPNTRFYNWFRLICVIVNRCFNLRQFIIWHYTIKKTHFVFSYYYGHLCINIGSAIIYILYLTGDLIISVSTITQSDYNIFLVIKMQEVLKSLKINGSEIKGFLSFSWWSVIISNILSIGYIILYCFTLADMVSIVDVISAYASISYEIHVLYALLLLKLTNKMLTVWIKEFRNSRNLGDSTNEEYINRMFFIYWDVQDIYMTIEKTFHHTVRNSIYCFIWIVAVVQYIYIFQILFYIIFTISISLWEIFSSLVFKTAGHQSVSIKVTWLILSGIL

>HarmGR172

MTRRTRIQQNNNNIFQVEMLLNNYVDEDIQSMLLPLNMLHFIRLCPKFIIRDNFITPNSSKFNSVFFIATIVWLFALFYDIHTEFWEELVNFNITDSVSFSVFFSSGLIVNLSLSVFRTRDYVSFVLKFQNVHRFLNNESDFKVFTFINWFIVILFVIIYGGFVIILILITDTLTIQLFCAFVLLSFDADIIYIIRFMKLLKDKFNLWNEQALQVRNMRDGNKEEYCQKLYQAYIDIMECYRLIKTFSRLVVSKKQASLPVSLESFLVNYFIAKTSCYCSISSTSRNYYPQ

>HarmGR173

MWSCFKQYWLPHHRSVIIENTSSFSPDDIIEYNRIEEDLQAILRPLNFMENIFLCAKYSIRRQYITSNSRIYNFFRVFCIILNRCFHTNQIIDWNITVWNERNTTLHFYSTLCVSISGFIGYMLYLTGDSVSTVSNIVLSRYNILLALKIQCALSSLRINRSQVTGIIICSWCCVIISNIYSISWVIFYCCYYGNINIVTIVTAYASISYEISVAYAFILIKLTDKLLQQWINECRALSLEDSENVENVDKLFIVYCDIQEIYMIIEKTFQHLVSFEPS

>HarmGR174

MFSYLKEYFKLYKMSTVENISRVPSDNICENNRIEEDLQLILRPLNFMQGLFFCAKYSIRGKSITCTTRGYHLLRVICVIVAHGYNAYLFIVNSIAFWNNPIASSFFYSSLCLWVSSFIAYILYMIGDSLNSIVNISMSHLNIVLVLKIQHVLSFLRLRRSDVKGSIICSWACVIIANILSFGWIAYFCATAPEINYIPIITSYASITYEINVIYAFTLLNLTTKMLNVCINEFRTSSCLKASENVKYLHELFHTYCNILEIYMIIEKTFQHMVSI

>HarmGR175

MWDSVRNAQIFIPSSQPVALSKNRLNKEVYRIIYPFHLIFLVLCSSKYTVKYNFILKDGVLRKIVSFLSVCFVVIVSSCYMFLEKYTAYINNVQSIKIIFFIFHGVLILYCIANIMLFSMNIALSKQNIELILKIQLVHTNIDFSKSSKNFIIWNWIYLIIFLVIDLAISSSYYVTYYEQDVVDALGYLCNYLFAVFDMNVIYACRLITLLRKYLNKWSKVILKLSDGVNNRNCGQLFEIYDNIIRAFQLYKTVFQVVVSSLLTIAVAIDSFVHTFKQISCWCHVPLQILSTTVNIFSRNLGFIESRLQIITDTAESVNEVIFVLLSLKFLLSLFYL

>HarmGR176

MTTVRKIVSIKPIYNFTIDKDIQSMLLPLNLTQYMMFCHKYRIKNNLITPNGLRTKCITIIGTIIFIFSIAYRTFSLSFNQNSAAFSPLIYYYSYYDTIYYGFGLILSCVLSIRNTKKHVRFILIFQKVHRFLNDKTVFKQSVVFNWLFVITCLVIHFTTVISVALMLIYYIKYVWNGFVLVVFDLNVVHTVRFIKLLEDKVEVWRTRLLNSPDLEITDLPSYSKGMFQAFFFFF

>HarmGR177

METLPLSNAPKSSFSSNVIDKDVQAIFYPLNVMSVLLLHPKYVIKNNKITPLSNVIKIFSACVTTLYLCQHAHKFFSVVLDDNIRRIQPVSYLYYATGSDLLFLTWGFIMNCAGNIIYTKKYVAFILKFQESHRFLGSACFKRFIIVNWLSIISMFGYFISSCIYTYFTFFHPPWGTIFHMMVLANLDADAVYACRLILLISEQFIQWNERALLLKENGEDKDYCRKMFETYGQILKCYKIYRNTMQFMVSRILGFLMIISCL

>HarmGR178

MSIKMKPTAHPKEFTCTYIDRDVQKMYQPLNLMQQLTLNPKYQIKAGFIKPNNVKSILLSFCGLISYVGVSIYRVWELATDENMQRYTPIKFLNFATLIDASFNSTGYIMNFILPIIEVKQNITFILTFQEIHRFIHKTDNCKIIVNNWISVITYFSFYIFACIYIYVYYMPNWYVMYYVFILCTRDTNPIYAIGVIKLLTEKVFLWNSELLMSSKAGHREMRCDRMFRAYGHILSCYDVYKNIFQIPVSIIFGKLRNYNCVYIKSSFLFLN

>HarmGR180

MQVSDIIKPGDFNNKSYNSMIPIIRILKVFAINGNVAPNKTSMLIKSICACSIFGCLSSYCLYYKTKYVYNRLDISIRVTDMTQMICDFFQYTVDLFFVYKFGRSLYIEYFRQFEIIDVCLETSCYAEMKRRLLKTMTFFLVIWFISSFTDLGAWVITYGWMIPVVHSLSYLYLLIKILATLDLIANIIQVEVRLRIINNFIKNCYNCASACPVGILADCIRNKNWLHGEDGSPDQSLKARSIDSHEIKRLSKCYLMLTEQVMFINKMYGFRILLNTTSLLFDMVKILNLAIRIIVGSQRTLYNSAGYNFLPGVSGFVRFLTCAAILITLVNRCEQAYRQRERILNVIDHLLINKNPDLTLRSAIQDLQSLLQDRPICFNMAGFFTLNFSLLVSIASVVVTYTIILLQSVN

>HarmGR181

MAQTVMNFVVGVSQTHKSIRFVLTFQKVHRFLKNDPHFDNLIIWNWIVVIIAATFYNSAFVYFTNYLGLPMYFIYVSVILSAFDFNIVYAYRLMTCLTHKLEVWNIKVLSSGETNCDIFSKNMFQAYLDILECYDLVEACFQHYFFFVFQILFYIGEVFIHYLDLMSIAGMAIFSTVIWLMKNLAWQIMLSQQCEKFYSTVQSAQDNCMFVLKSNCTESVQRLCKNVRRLHRSRFSKLRVCALFRADAALQLSLMALLTDYIVVVLQFAFL

>HarmGR182

MSLMCTVATALRSSLNTKKALDIGRLQNINVMPFPSNVVDKDVQSILLPLNLLQLLTFCPKYRIKNNVVYPNSLIAISIIVIATSIFVLSFVYRSYYLLSANVLPWFSDFSVYFDVIFYSIGFIMNCFFIIVQSKQNVQFVLIFQSLHSFLKETNLKNLVIWNWVFVILVLLVFHIIFIYLLVILKLPFHFLYYSILLNSLDFHIVYASRLMKLLEHKLILWNIQVLSCQGNIDESYGKRLFQAYIDMLQCYELVKVFFQQFVKVLVHSLFYIKASLDLFSMTVKLGQMHRIAMAALSISSIVIWLVKNLLWQIQLTVQCERFYSVILHSQDTCAIVLNSNGSEAEKRLCKNVRRVTRARFSKLRVCALFYVDASLQLSLMALLTDYIVVLLQFAFLDP

>HarmGR183

MSINLVDKDVQSMLLPLNLMQYIAFCPKYCIKNNFISINSLFSNFISFCGLLIFLSSFLYRNTLIAQSLGHSFTVFMYITAYFDFVYYCCGYVMNFIVGIMQTKNSVNFVLTFQNIHRFLNNETYSRNFKVLNWIIVILTVVGQTTIFAYFNITVGLSHYFIYISFIITVFDFNIIYATRLLGILENKLLLWSNNVLDLREIGEIYDENYCRNMYLAYVDILKCYELHKVCFQEYICFYITETFLHSLICIQVSIEMCKMAASRGNISTIGTAILSTMSVLLWILKDLFWQLLFCRQCEKFYSSMENVPDYCTLILKTSCSESVRRLCKNVRRVHRARFSKLRVCSLFDAGAALQLSLMVLLADYTIVLLQFAFL

>HarmGR184

MEIFVNSRQINSNNIVDSDVQSMLLPLNLLQNVFFCPKYRIKNNYITPTNLMSNLISSIATLVFIIMYAYRNYLIGLFKTSQFSTAWKYSSYFNGFCYSLGFIMNLVIGIIQSQNSVQFVLTFQNVHRFLKNENGFRSFIIWNWVAVCLTLVYYVFFFIYQYTRGTIGKIHACVGFLLSSFDFNVVYATRLLRLLEYQLVLWNNRFFKLRETSDIRDKDIIQKLFNAYANILECYDIIKISFQHYVSFTLQLIIDLLKYAANNDCQKTVIIAIRVSIAVLLWLIKNLMWQMTFSHQCERLYLANERTLDHCAFILTSYCSGMEKRLCKNVLRMSRVRFSKLRVCGLFYAGAALQLSLIALLADYTIVLLQLAFL

>HarmGR186

MWKKSIHAFKILPNNRLEKEVQRIVTPFNVILTAVCSPKFRIRNGYITPSSKKIHILLFFGITACNVWSCYGITKSQNEYTTTSIVSYFFSLTIFFYYIDFILFTSCNVLHSRRQVSLILKIQELYRNIDITKKVKNYIIWTWIWFLATFSVFLVNFLSFLMNLDRIFFIHLSTYFANLQFDLNFIYSVRIMTLLVIYLKEWTKSVVDLNEEEQNKEYFVKKFKTYQNILRAFKIFTLCFKDIVSTSSYI

>HarmGR187

MADSSRRLFELVFRNKLDEDTLMIIKPFNIFLRIFFSSKFKIRNGYITPRDKTYYILPFIFVSLFKVWTVYYVYIYNSSILNNTFRHIYFWHIFISYCIYYSLLVYCNIVNSQNNVVLILRIQEIFRSIHLKNGIRSYVIWNWITFVVLASLECFCTTIYARTMNLLSSLNSFDILLSICYDFNVACSIRLIKSLTLNLVEWSNTDK

>HarmGR188

MWYSHRPSQLLLFTNKLDKDVQRILKPFNIILTIFFSSKFKIRNGYITPCDKKLHIILFICVIFLNAWSVYEMRVYISGKASIIINSQIIFSFLLIICFFTYFILIFSNIAYCQSNILLIYTIQDIHRAMRNSSSFKNYITWNWITILICICFDILIMSSYCMLLSKIHIFSVSTLYLNMASEINVFYCIRVLAFLIMSLEEWIENVLVVKSDDYCEEYCGKLFKVYQDIIKAHKLLQNCFRLLVRSLIFILIKIF

>HarmGR189

MFQSKRSVILLTTNKLDEDILRIVRPFNVVLTAIASSKFKIKNRHITPCVKTFHLLICFSIIALKLWSSFMFVVGRGDFKHKKIVDNFFCLTVFFYCVNYTLLTYNNIMHSCHNISLFMKIQDINRNINISVQSYVVWTWISFLITLILFISDISWFLLKSGIVGTVHVSENILVFQFDINFVYGIRLVTLLVMYLKEWARSIIIMTEERPNEYFVRKLETYVNILEAFKLFTICFKAMVSWFYKLDEMLS

>HarmGR190

MKVTVVHPFVLSPSNKLDKDILRIVKPFNIILTAVCSSKYKIRHGYITPCGLNYYILTYAGIGVFLVWSTYNMISINIDELNQRADLAAYLYCTMFLFYISYGQFIIYNIIHRQDHISLILKIQEIYRSVDISKCVQNVVLWNWLSFFLMFCCMIPNISLLYLSQNFVSYTHITNHISNIMFDFNFIYGIRIIALLVVFLKKWSESILDKEFNLEKKLKAYQNILEAFQLFAICFRAIVSSS

>HarmGR191

MEGKTREKKFSCNYVDKDVRTIFRPLKIMQTCSFNPKYQFKNNFIYPANTISDVIAYFGVITFLTIMIWRITDILFDENLRRYQTLNFLYFASYADSLFYSFGYIMNLILHFLHSKDSVKMILIFQEIHRFIRKSASLKNIVCRNWVALALMGGFQSIVVIYLYIVYMHPPWYIIFYVFYLLSPDYNTVYAICFMKLLLDKAVLWNVNLLLSLQGQRKVLCRQMTRTYGQILDCFDVYKNVFELPVSTYKIFIC

>HarmGR192

MNMNQEFAPVFTVDKDVQTMLMPLNLMQNVFFNKKYRIKNNTILPNNLTSNILSFVASLTCTLIFLYRVYLMSALEDSRFTSILYYSSFFDCLYYCVGYVINFVSGVIFTKKSIEFVFIFQKIHRFISNETDFRLFVIWNWVSVSVAASGYVLICIFFVTNADLSVFNGFPCLFLCIFDFNIVYAMRLIQLLRSKLVLWNLSLETTSI

>HarmGR195

MEIKSSSFFKSFQKCMGPLYFYKVLILLQVLLGRYFSLSKSKLTRFFTKLYCVFMYIHMIYKWNDVVLVSHKFVLPPFIMSEYTGYFVISIILSEDYFFNFCDNLLTNDRVMGFKNIPHVPPNVIGFMLITVISRVAFVLTRHFTVSLPSVHLIYVTVLLISLDLSHIYTCVIFCMIQLRMKVLRCFLENIHIPINIVSGNEVEMSIKNVRKSLYYYNNLLDSMAAIDKHTQCMVSKLYLHQ

>HarmGR196

MFSYLKEYFKLYKMSTVENISRVPSDNICENNRIEEDLQLILRPLNFMQGLFFCAKYSIRGKSITCTTRGYHLLRVICVIVAHGYNAYLFIVNSIAFWNNPIASSFFYSSLCLWVSSFIAYILYMIGDSLNSIVNISMSHLNIVLVLKIQHVLSFLRLRRSDVKGSIICSWACVIIANILSFGWIAYFCATAPEINYIPIITSYASITYEINVIYAFTLLNLTTKMLNVCINEFRTSSCLKASENVKYLHELFHTYCNILEIYMIIEKTFQHMVSI

>HarmGR197

MDNSVYKLLSLRLLFGHYFKLSSSKWICYIAKIFCFSMLIVNFAINCILLLDFTSFDISQISLWMLLWIMLVESSSSILISLYTDETYLLKFSAKIKNYVSSPTPCRATYVMASFIIPLNFSSVLIAYLYEFGVAVNIFYNISYTTCYCSYLTSLYITEMYAKAINNLTSAIVNRLKDINISDEEKRVCIENFLDNYLKLLKIYNATMTVSRINVSVCKSFE

>BmGr1

MNRHDHRFSIYNPKRNEAMWKRELFVNNEGKDIKDFQIKDIYGPEITDKDGALLDKHDSFYLNTKSLLVLFQIMGVMPIMRVPKSAQTTRRTTYNWISKATLWAYLVWGLECIIVVKVGQERLANFQIGSNKRFDEVIYNIIFLSILIPHFLLPIASWRHGPQVAIFKNMWTHYQLKYLKITGKPIVFPNLYILTWGLCIFSWVLSFAVVLSQHYLQDDFELWHSFAYYHIIAMLDGFCSLWYINCNAFGTASRGLAINLHKALEAEHPALKLAQYRHLWVDLSHMMQQLGRAYSNMYGIYCMVIFFTTTISLYGALSEILEHGLSYKEMGLFVIVAYCMTLLFIICNEAYHASRKVGHEFQDRLLNVNLGAIDRSTQREVEMFLVAIAKNPPIMNLDGFTNINRELFTANISFMSTYLIVLMQFKLTLLRQGARKTVTAIVRAIFNTTITDNGAGGSDEDQE

>BmGr2

MIPDHLFEEGINNTFLDYDMRHVQRNRNIQEKTQKDYEQEQRDLLSSQDGDTCEIHDQFYRDHKLLLVLFRALAVMPITRSRPGTITFSWKSTATIYAVCFYIAATAVVLIVGYERIQILQSIKRFDDYIYAILFIVFLVPHFWIPFVGWGVAHQVAIYKTNWGKFQVRYYRVTGENLKFPNLKTLIVIISVGCLLLAVCFLLSLCALLDGFLLKHTSAYYHIITMINMNCALWYINCKAIKIASQSLSECFQRVAAGVQDTLLSIDVLAVDRPTQKEIDHFIQAIEMNPAFVSLKGYAHVNRELLTSVRFTTIIEADLLMIY

>BmGr3

MSFEIKNNFFRTSVPIPNGFPVQTEAKSKNKPIFLDVSPAPTPKVNSPNAIIPMKNNLIDPFINKDIIYENIKPVFMVLRIMGVLPLTRTTSGVTYKQLSNRILPVKLYKKSLLIAIIIPILSTTSVIVTHVTMVHFKTSQIIPYVFLEILTYMLGGYWYLLCEILSLCANVLADDFQQALRHVGPAGKVAKYRALWLRLSKLARNTGVANCYTFTFVNLYLFLIITLSIYGLLSKISEGFGTKDIGLALTALCSVFLLFFICDEAHYASHNVRTNFQKKLLMVELSWMNTDAQTEVNMFLRATEMNPSQISLGGFFDVNRTLFKSLLATMVTYLVVLLQFQISIPDATQPEIPTNIDDHVQNITDTTTEASSPISTLMSAFAKRKND

>BmGr4

MSRIFSMTRYFGVSTCKPSIAFGWTVILLLMLLAIEVGAIWKIVRLLGGWAVHSTDSRGFTARLSGCIFYGNALLSLILSIKFVSSWEQLSERWSRTETDPGLRLPSDSRIKRRTVLVSAFVMTCACVEHMLSMMSATGFDCPPEEYTERYILSSHGFLVQNDEYNLWLAIPIFIMSKLATALWNFQDLIIILISMGFTSRYNRLNTYVHRVVMLERNLKEGAQVSSENYMRFQIWRRIRQAYVRQAALVRLVDDQLGALVLLSNVNNLYFICLQLFLGINSKDRGSFINRLYYFISLGWLMFRACGVVLAAADVYIHSKKALISLYLCPELAYNLEIKRLKYQLKNDEVALTGMGLFSLNRELLLEVAGTIVTYELVLLQFSNED

>BmGr5

MYACYKIIVALSLNRQYNHTVTRVGNRKHIKSTTRGNFRERILRKVQNRISPEPIQEDSKIPLTCQFQLFQTAMKHLLISGQFMGLNPVSRISDHSPTKIRFTVLSWKFVYGVTIGIAQACATVLCFCKLLKDSVNIVALDFVKIVSAYFAFYLSTGCNTFIFLRVASKWPTLIKHVYETQLDSYIDVKVKNKCFAAYIIFFSMSMTEHMLSLLSKFVITMDCLPKGSDLFESYIIRNFPWLFEFDVPYYLPIGVILQFLTLVSTINWSYSDLFIVCMSIYLTSILKQINKKIEMAGNSNHLPIPFWRTLREDYTRATRLVRSFDDTISSVIFLSFASNLFFICLQLYNILSNGVTSKYNLLKEMCPNYPSGPLGGYEQIMYLLFSLSFLLGRSLVVSLVAAKVHSASMVPASALYNIPRNMYCSEIQRFLDQVHGDKVALSGLRFFYVTRSLVLSVAGTIVTYELVLLQFSNED

>BmGr6

MLLRNYKQNLSFWTSAKKSKIHKIQSQETVTFQGSLKLVLFIGQLFSLFPVCGLLSNDANKVKFVPISWKCGYSMLSMIGQLFIIVMCILYVAHFETTLNGTTPIIFYGVTFISMIAFIRASRRWPELIQHISKSEELDPSFDFRLKKKCNITLLLVLVLAILEHIFSIRSAYSASQICYPHTGFYEGFVRYLYPWVFDFLPYSAALGMVTQFLNIQSHFIWNFTDLFVICMSYYLTSRLDLVNKKLLPAQGKYLPEIFWRTTRETYCRATKLVRKVDEIINGILFISFANNLFFVCVQLFNTFDDSVDMVGLCYNYSERRTKPVGREPVIYLLFSLGFLISRSITVSLIASQVNLASTVPAPILYDVPSAVYCVEVQRFLEQVNGDNVALTGLQFFSVTRGLLLSVAGTIVTYELVMVQFNQAPASDSFTEKLVENNISTIETFYNYS

>BmGr7

MVLEAHTQIQYCTAKANYCEFHAGLRHLMRLARWAGFFPVQGLSQTNPDDVRFEFRSLYALYHAITVIGQTVMTFLAFYSFVDSNVSLSVVSNFLFYFTNYVTLVLLWRLSKNWSALISKTLEFEQSVTEIRTTRNLVSRTNTLTYVVLIFAMIEHALSKVFNIRSVMCCLGETSLNHTVINNYFKFKWKFVFDYFSTSTTYSYFVGFIAEFLCMQATFLWSFTDVLIMCFSIYLSSFFEDFNSTVSSFMKKASKTVPWSTLRVQYSQIVLIVKQMDEQLDYFVLISYFTNLFFICFQLYNSLNRIYDANDVCNENMDIIATASVTYLTYYVFSFLFLVTRALLLSIMAANVHSCAQVPQLALYEVPTADYSLDVQRFQLQLRYTTVGLSGVCFNVTRGMILRVIGTIVTYELVLIQLTKKNLDNDTSIRDYYLPKHLI

>BmGr8

MAPRSVRSMVGTSKKDMLKGGFYETVRIPLYIYRLIGILPISGLWHRSSKYNRFSLKSFYTIIYAPTIVMQTFLLLVHIYDLFAFFFGHQRLGRLIYHMNFYTITILIFMGSRKWKNVIKEIETIELTLPRLRNSKKALALTKSFVFAFFVFSLAEVVLILQFTLRLTKQRHVLPGDSGLYLRSYFVYIFPYLYDHFPFSYVMGFIVQIIKVQGIITLNMVNCSVVILSIYLTNRLKHYNRIVFAKGSKTNNTRLKWVELNLLYTRISNLVKIIDKNLNPFVFISFTANLSYICAQLFYILNKLTSSRTVKITSFLEDKRSDWETVLYISISFALVVLKVLLVSITAAEVHTTSREPLRLLYTLPTAEYTIETQRLMTQVYYSNLSLSGLNFFHITRGMLLGMVATLLTYEIVLLQI

>BmGr9

MPPSPDLRADEPKTPCLVGGAHAFILKISSFCGLAPLRFEPRSQEYAVTISKGKCFYSYILVTFLVICTIYGLVAEIGVGVEKSVRMSSRMSQVVSACDILVVAVTAGVGVYGAPARMRTMLSYMENIVAVDRELGRHHSAATERKLCALLLLILLSFTILLVDDFCFYAMQAGKTGRQWEIVTNYAGFYFLWYIVMVLELQFAFTALSLRARLKLFNEALNVTASQVCKPVKKPKNSQLSVYATSVRPVSCKRENVIVETIRVRDKDDAFVMMKTADGVPCLQVPPCEAVGRLSRMRCTLCEVTRHIADGYGLPLVIILMSTLLHLIVTPYFLIMEIIVSTHRLHFLVLQFLWCTTHLIRMLVVVEPCHYTIREGKRTEDILCRLMTLAPHGGVLSSRLEVLSRLLMLQNISYSPLGMCTLDRPLMVTVLGAVTTYLVILIQFQRYDS

>BmGr10

MTMSIKPRLQCMVPPSLALALRVSRLAGIAPLKFVAKQSNIMIRLSTSLCVYSYLLVTALNVCTLIAVMIDFSVPVKLSIRMQTETKRFVWIADVVIMGILSGVGVYTAPIQMRRLIAYLHRIHKINSDLGTYSSSLTDKMLHRLTIGMLLITSVIIVTDFTFVMYLADLNHRQLLIAIMYWCYYCSYFIAHLLEMQFVLIAALALSSLKLVNNGLRTLLHQSGIESLTEIPNSNEQHTANAILPQPPKKSVNNSIDTLAFVVTKRSVRFPTAGWMDQRTIRRLALSYGSICEVVRQIDNNNGIIVLLLLASFLLHLVVTPYYLIISFVTESPHTGFEKVLNPILQTVWCLYHTFGLVMIIEPCHRTHEEMETTRELVSRVMCSADPRDPISIELEMFFRQLVLNKASYAPLKVCTLTRSLVATILGSITTYLIVIVQLEIKNMQ

>BmGr11

MKPFRFFLFVENVICVYRNYSFHKRYARAIILSRVMFEVSLIILTLHSCRNFGAVKYKTEIIFTYLATASSTILILLALYKTNRFTELFLNFKAFYRNRNLDVDHLEKWNRKQKMATVIIVLFCVIKFSTLIYTDLIGEYSTPCRGYFTEYLFYTNLFMCNARYLFEFSTACVVLHLVSEQLDYIAISMDCTMFLYIDISKKNIMSSAKKRKLKYFDIFKQFEKWTDAYMNVKRSANLCDTVFRAQLAIMITTITLYYIILLYGITSFNIERGKFSVVKSLSYLISLFGFLIALLLLSKAGQRIQKSAENLRRKLSKFLLHSLEDPEFHRAATNLLRLVCTHHIKMRCFGFIDIDMTLLPSCLMFVTSYTVIALQFNNVV

>BmGr12

MKNLKLCRTTFYFKIIMCSRFISGLYFKATSKKWISYLYKVICVLYIICITRLFYAKEDTFKPLVFXXQFIGNSIESLRTGEGHVLKCYSTIFSLKLIRNYLPDSNNHIPISSITNFLVIIWKVFDQVYIVLMHYFYTTDIIIHLRILSILTTIGVNLSLMPIIVIFELMWRAVKALRKSLGEHLKGPVLIEGRERLKAQQILRCLNVYKDLNATLKFNSTPMKTMILISTLATFIRLTLFLYQAILGHNEGLHLPRKILAIIYYALPVCLLGVLMELVARECDKLKTLMTKELLVCKDDSYCTVIVDAVSYIELNPLKFSILRAFNVNSTLILGLTNLCTTYLIAVIQFTYSCEDINGLSHSHSH

>BmGr13

MEDSFNRLLSIRNMIIFQNVCGFYHMCTEKLYISRIIKMYCVALAIVLSVFCFQNPDITYLSWDVVWVTFGYTLNVIICLRYNGNYFFQYWNGLHEIDIKMNLTSIDKEKVPISRAVFTVFLILRSTAFAMTIFVFGYLETGILSNTIISIYSINLTEFYRNMSNIPMILMFETFYVRIKILKEQLCSELSTVLGCNNDARQLKLILKYLRNYRSLVRHLMDTTLPFKILILVILVGSFLRSLLIGYAFVYNSDQIILLSLPVMFSTKILSEVVEIKLICTKELLKNKNEGLVLLDLDSKKPTFLTSKACGEQLQDALSFLNNRSYSYTLLQVIEFDCSLAFVFTSFCITHLIVVVQFTHVLD

>BmGr14

MNLHKNIIPIRNNLFANKVTAIALPKTLSVLFKLIHIFFLLDLGVYEYKTFKIKCIVKFLTISGSLTISVVCFSFMVSNLSEHTFVGWYGFFISTYIFVVLFFNLSNRMTFVEFYKTLLRFDANYGIDSNEYKFNFKIIFVNILFIANRMVLSFVYCSYYPQNCIRPRYAQILFMLPWLTLDVLLTTNMFLFYATYCRIAKFPMLIKNSMNIVALRNSYKLIVDSLEKTQTSFDIVFIIALVFSVPEIMMSIYSTLLEVISKHFLEVASILSLNYVAIAQSLLLTLAPSLCAGVLPWKTNNIKIILHEKLFTEKDKASAREIELFIKYIESRPLKLRACNLVPLDFSLTIIVLNICVTYLIVIIQFTHLY

>BmGr15

MISSSDINHKRNKVFAYNVPGIALSKTLTVLFKLLHYVLLLDVGIYEYKTFKNKCIVKFLTIATGVSVSIVYFCLIATVLRKNAFFYWFYVLFISQYMIIVFIFTLSNGMSFTDYYKMLLRFDAKYQINSNNYYFNIKIILVIIISILNRIGMAIIYCSYYTKNCYEMSFSQIIFVLPWLTRDVILIMNVFLFYVTYCRITKFPALLENTKNVGSLRNSYKLIVDSLEKTQKPFDFVFTISLVFNIPEIMLSIYFTLLQVIHSHFLEVAPTLSISYFSITHSVVLILAPSLCAGVLPWKTNTIKIVLHDKLFLEKDKNSARNIKLFIKYIEARPLKLRACNLVPLDFSLPVIVLNLCVTYLIVIVQFSHLS

>BmGr16

MIMNLTTDRISKRNKVFAYNVPEVTLPTTLKVLFKLIQFTLSLDFGVYKYKTFKMKCVAKVLTLAGCLAASAACVSLIISNIFENQLFFGWYTLFVCQYTIVIFMFTFSNGMTFIDYKMMLLRFDAKYQIDSNVYHFNIKIVLVVVISVTSRLFLCAVYCIYSTENCIKPWYNQLLFFPWLSLDIVLIMNMFLFYATYCRLAKFPSLFENPKNVVPLRNSYKLIVDSLEKTKKSFDAVLIAALIFNIPEIMMSIYYTLFQVMNKHFQEVAPVLSLSYFTIILSVLLILAPSLCAGVLPWKTRHMRLILLEKLFAEKDKNSAREIELFIKYIEARPLQLRACNLVPLDFNLPVIVLNLCITYLIVIIQFTHLF

>BmGr17

MGFSLGTTALSMFFFEKPVVFTIIQITMIIVKPAKYKLSDPFRPKDTSKLSESIIMYFKLFHIFLGIDLGGFRYQNRQVKYAVRLISLIQPLAIYGLCIYALLKIIANTEFLWYTISFTEYVAMSVAITLFSNEMTYCNFMINLKFIDTKLKIGDESFRIGVKLISSTILIGVTRCFTTTTYCLLGFCAKPTAAQILFQIPWLTIDLMLLQYMFIFYACYCRLVKILRILKKRNTDIEEMRRIYKTLVDVLDRARAPFDLAYLLGLLFSIPDVLYSIYESIIKVGEINTAKALSMSIIYITNIQSLALMFAPALTAGFLPSLTMKMRIILHDKLLEEQDKKTYRHIVLFIKYIETCPLKLKACQIIPLDFSFPIIILNIVVTYLIVAIQLTHFL

>BmGr18

MRRSTKVISMVNQSDKGEIKTCSRFMKIYFFVIYILTGFNFGFYTGRGLNFLRVIQASVLLLRFIIASNCIYIAFHFRLLEAIWYSLTFSESLAIVVCFMLSRSALSCKNLFEYLYSVDQELKKSVGPSIEVKLALYTVVVSVLRLTVYVFCAIAYYETLHEGFCVELVYNTPCYCSDLYLVIHFTIFHSVYCRLKALRISMNEKFDVYKGTLIYKSLIDNLEEIKKSLDVPFFVILLNAVAIAMINILVTLEISYGQTMKFIRTAPRYLETVLLFSSAFAPVLAADMMASEAQKIKVTLNNILQRDDSLLEDDRRKVKQFAGYVSARPFRLRACRVLSLDCTLPVTVLSICVTYLIVVVQFTHLY

>BmGr19

MRRSTKVISLVNQSDKGEIKTCSRFMKIYFFVIYILTGFNFGFYTGCGLNFLRVIQASVLLLRLSVASYSMYIARYSPLLEVIWCCLTASENLAVVVCFMLSRSALSCKNLFEYLYSVDQELKKSVGPSIEVKLALYTVVVSVLRLIIYVFCATAYYRKLFDGLRLELLYHTPCYSLDLYLVVHFTIFHSVYCRLKALRISLNEKFDVYKGTLIYKSLIDNLEEIKKSLDVPLFVILLNAVAIAMINILVTLHISYGKTGCRIDYKSVAEMACSQLVIRTSRSETANGSSEAKRKKLVVGPLMKLITAAPRYLETVLLFSAAFAPVLAADMMASEAQKIKVTLNNILQRDDSLLEDDRRKVKQFAGYVSARPFRLRACRVLSLDCTLPVTVLSICVTYLIVVVQFMHLY

>BmGr20

MRRSTKVISLVKQSDKGEIKTCSRFMKIYFFVIYILTGFNFGFYTGCGLNFLRVIQASVLLLRSIIASYSIYVAIHFRVLEAIWYCLTFSESLMVVVCFMLSRSALSCKSLFEYLYSVDQELKKSVGPSIEVKLVLYTVVVSVLRLTVYVFCAIAYYESLHEGFSVELIYNTPCYCSDLYLVVHFTIFHSVYCRLKALRISMNEKFDVYKGTLIYKSLIDNLEEIKKSLDVPMKFIRTASRYLETVLLFSSAFAPVLAADMMASEAQKIKVTLNNILQTDDSLRKSYGATVFSFALLVTMIVEATMVGVEDNNIIGPYIDVEREARLTGLYVMAIILTLMFLAKFIFDLVFVYGVVMERAGIVKAYFIMWAVFFFLSVSVFFLNCLDFNTSTIVLEVFYIGLNIYAILLSHSFYKQLNTREDV

>BmGr21

MAQRTNSINLFRSRPPDIRAGVGEPRIFSKFICGTMFTQKSLVNFDLGKTPRGGDQEHSKFFKIYFLAVHSVTVLDFGFDRNAKKLTKILISMFSISVRMGLAAVSFMSLWGRPNALALGWAPGTLLCENILVAVTYSASRSTFKCGDLFADLSTIDELFGSACDYRIESKMLLFTATMTVLRVVIYSTSRLVRADGFDFVDVLEVLNNLETMCMYLFLTVYFFVLFSIYCRFKKLRELMKNDFEIRRANLIYIALKDCTDKIKQSLDVPFLVVLVFTVLVVMVDVFITLEMIISNKYNMAVYVVRYLEITLDFLMLFAPVLLADMMAVQVDGLKITLHDRLCLNNGVGHDDLSDAMWMSFVDMKRYSSLAEFIGYVEARGCRLRACRVVPLDLTLPVTVFNVCVTYLIVMIQFADLY

>BmGr22

MDFGFSLGVYKRMKVLENISLVLRVMVAIMCAAMVMKQDILDSAWADITLTESLLVIVSFKLSKPKLSYRELLENLSIVDETQGAPPAGYKVERKLITYIAGVTALRLTVLCLYCVAHTEQYSIDNFIEFLYNVPCYCLDLYLIVHFIIFHSIYCRLRTLRKALSNNFDVYRAHLIYKTLIDCTEEIKKCLDIPVSRSDRHRHSLHKPMGTFLKSYRTPRVMFQLVVILIATILVVMVNVLVTLRMLFKGEVTQRGSVHVQVVLPENLPLSYFNIVHCISLQSIISAFLLRYIEVILSLALLKLLDELKPDAFKSHWKKIWRVLIVGNDGQFARNQTSTDNIVQAANQWNMTRWIRADRTSDIREVAGSQDHLTETDLEEMLTTRSCVTAYWIFDAGSEHVWYVAAAEERREVRQFAHYVGTRPFRLRACHVLALDSSLPITVVSVCVTYLIVIVQFTHLY

>BmGr23

MAQFQIPSMAGSGLNVAPFSRGRRHEGPKHSNFTKKYFLLVHLVTCLDFGFHRDNDTKTYKWFHAANIGVRLVLSAYVCSVSLSQDLSFASAAWTILNNSKHLLVVAIFTIFKPKSSCAEILKDLLMIDEALKIHRGCDVKGQITVCIVLVTAARLLIAAASSLSLHEAFSASVGAAEVLYSFQSYCLDFYILANFFIFYSVYCRLKNLRRVLQNNFNIYRGNMIYKVLVEHMDDIKKFLDIPFVTSLLVTVIMAMINVLKTLQLIHDGENDVLTIVLRYLEMFLSFSLIFAPVILSDLMSIEADNINVVLHNYIYETDAADIAVAVHPSARPSPVSATEERTAAAAAASVSLAIFSSRLTSFNLCWRARGAGAETFVAVCVVRLGAAVPTPRVSGALAGLHAPRDRAQHLRHLPHRGRAVHASLLISLSLL

>BmGr24

MCINKKIQSIIKSLVSIRTIMLVQSILGFYHKMSNNFFVSFLFLTYTTILISVLSFYSVNDVMAHKFAYTLSMILEYDINTILSLITAGRQYFNFFEEMKKIDFSIGFGELNIEDLPLSRTLFVTIFVTNILLSIMTAALILFFSTPFLIISSGSTYAMAIVFFGLSLNVLPRIIIFELIYKRIKYINLSLKRKLKALALECDHTIARFEIINENLIIYNKLLQSLGNVNVSLKSSILLTTFTCFFRCSLICYYVITMNEDKVYIMQIIELTKQTLFLGVLIILAEYIKNEIENLKMTVSLQLFTCTDTQLYHQVSDXFEIHRITPIQLCGFKNMSVDTNLFLGLINVCSTYLIIITQFLNAYVN

>BmGr25

MFVKCLKYVKKFKPMFSVMFIMNFRLICGLYYRIHSDAFVCFVFKVYCILCSMFLFFTSSDLAAPFSRSIPILATLFEYVANVLDCILTGQSYFFHLRMELMRIDPRLRGLDRPPASSIVFTAILSYKIFILAVYIHGKARTTYLQYEWFSSIGIHLLVLFSNLVHMNRMLIFEMVTFSLEAQKKTLGELLKSSLRRERVERKCEILNRFLKTYKRIIELFNNTMAATKLMTLISVVSCFIRILTYLYQVLTTQLSSSSAGSIFTTRHLTFIVTFVREHVLSVYFLKKVGICQRLQINFKIKCNVKTETLELISQDDEYTEKLEDALDFINSCSSKITILRAMTVDATLPLTFISLCTTYIIVVIQFSHIYD

>BmGr26

MNKTKIYRKKLDKNERLVCSVQPAMFARLIVGLYYDIKVSNRVKWMIKSYCISLSSFICYLIIFRDDNFSLHPKLTSVMEYITYVTFSFLTCDKYLFRYLRFNPRTDGYPIFLYLCKKFEKFFKIIICLFVSFKILGVVLMMQSWPILSTPKYIWGTLALHFLWLASHMGRLVFILVYGILFCRMRTIRIIFENRGFQNTPQNRLTPKRYILMYEAVLNSIESVDFPVKFLIFTFICCFAPKLVVSLFEIMEEMKKGELSLTTFIWFLVELSPSYLFLLLSAIALDLVSEDVQELLSITIDRRLNCKNEKERSEIQEFFQYLRNNPFNYTLWQVVSLNLRTLLVATSFSIANVIAIMQIKNSKI

>BmGr27

MVFKYKIMTKAPKSLPVLKILMLFRLVFGNYFRLSSNRYINFLVKSYCSTFTILLSVMCGKRLKNDSPYMLSLTEYILNKILNYATSEGYIFKYCNSIKTCDKIMGFKKLPIITIDVFIAIIITVITRTAITIYFGFLFPFDKYQVVLYVGCIVFSNDLNSLTIMNVFGLLNNRMNLLRKSLEAMTVPINIIGKNEVAPKVRLVRNAFRYYSNLLDNLDSVNHCVQYSLSVTLLLKFPKAVLLCYDSIKTYFVKIDNNFAMDIVDPTEIILSIVVMSFPAMLCEMITNEVEKIKAILTKHLIQCSDNSLRFELNITLLYICHRPFKYILWRAIPLDTSVPIGIVSLIITYVIVLIQLLHFST

>BmGr28

MAHKIATVGPTNATATVKNNKRKLKISKRVTIFKVVRCLRFILGHYTELTSSKLKAFLIKCCSLLLAVIIIYAPLNYIKMAYVMGLIEYLLFVLLSLFTGDEYFYKFHNSIKSIDVLMGYKRGKIIDSNAIIFLLSVITIMRIVIIYCRSTVLAFRFTIIGVYLAIFSLRISYMLITVIFFAMYHRMKFLRKKFEIITIPVTIIGKQKVASKIRLIRKYLINYHHLLDCLRDINGGLQYFLAIMIACNLPKYIFFAYSAIKIQVLEHITIHSAVQNVEMFEGFLFVVVPAIFAELTTAEVERIIDVINRQLLRCTDEHMELELKVALEFIRRRPFDYVIWRTVPLNASLPIAIISLCITYVVIVIQLTQFHDNF

>BmGr29

MYLRSKKSRFKLFSFERMIKILLMICGHYVQTDSSNVVSSIHRLFSIAITICLCPNFEFNPFYFHVIESVLYSILSQFTQYGFFFRFCSTIKTFDLLSGFKQIPLYTKRVCFFLLITLFMRLFTVLIHFLAYQSKFVTFCAFIIMLSANTGHILMTIMFSTLHTRMKSIQKLFANNPIPVNIVGKNENASHIKRVRKGLICYNNLLDTLKDAEKEIQFTLTVTCLCHVPKIICYVYFVITVIYKSKFSGYNLVPLFDMILACMAVTAPAVFAELTKNTVDKIKKILGSQLLRCSDESLRYELEITLEYVIQRPFSFSIWRAVSLDASLPVAMTSLCITYVIVILQLTQLRP

>BmGr30-1

MYLRSKKSRFKLFSFERMIKILLMICGHYVQTDSSNVVSSIHRIFSIVITICLCPYFQFNPFFFHVIESVLYSILSQFTQYGFFFRYCSTIKTFDLLSGFKQIPLYTKRVCFFLLITLLVRLIIVLIHFSAHQTKLKTFCAFLIILSANTGHILMTIMFSILNTRMTLIQKLFANNPIPVNIVGKNQNASHIKRVRKGLICYNNLLDTLKVAEKEIQFTLTVTYLCHVPKIICYVYFVITVIYKSKFSGYNLVPLFDMILACMAVTAPAVFAELTKNTVDKIKKILGSQLLRCSDESLRYELEITLEYVIQRPFSFSIWRAVSLDASLPVAMTSLCITYVIVILQLTQLRP

>BmGr30-2

MYLRSKKSRFKLFSFERMIKILLMICGHYVQTDSSNVVSSIHRIFSIVITICLCPYFQFNPFFFHVIESVLYSILSQFTQYGFFFRYCSTIKTFDLLSGFKQIPLYTKRVCFFLLITLLVRLIIVLIHFSAHQTKLKTFCAFLIILSANTGHILMTIMFSILNTRMTLIQKLFANNPIPVNIVGKNQNASHIKRVRKGLICYNNLLDTLKVAEKEIQFTLTVTYLCHVPKIICYVYFVITVIYKSKFSGYNLVPLFDMILACMAVTAPAVFAELTKNTVDKIKKILGSQLLRCSDESLRYELEITLEYVIQRPFSFSIWRAVSLDASLPVAMTSLCITYVIVILQLTQLRP

>BmGr30-3

MYLRSKKSRFKLFSFERMIKILLMICGHYVQTDSSNVVSSIHRIFSIVITICLCPYFQFNPFFFHVIESVLYSILSQFTQYGFFFRYCSTIKTFDLLSGFKQIPLYTKRVCFFLLITLLVRLIIVLIHFSAHQTKLKTFCAFLIILSANTGHILMTIMFSILNTRMTLIQKLFANNPIPVNIVGKNQNASHIKRVRKGLICYNNLLDTLKVAEKEIQFTLTVTYLCHVPKIICYVYFVITVIYKSKFSGYNLVPLFDMILACMAVTAPAVFAELTKNTVDKIKKILGSQLLRCSDYS*ICNSTSIFV*HLASCLSRRVSSCRHDKLVHHICNRYSTAHSVKTLIDSSTINSRNELFI*F

>BmGr30-4

MYLRSKKSRFKLFSFERMIKILLMICGHYVQTDSSNVVSSIHRIFSIVITICLCPYFQFNPFFFHVIESVLYSILSQFTQYGFFFRYCSTIKTFDLLSGFKQIPLYTKRVCFFLLITLLVRLIIVLIHFSAHQTKLKTFCAFLIILSANTGHILMTIMFSILNTRMTLIQKLFANNPIPVNIVGKNQNASHIKRVRKGLICYNNLLDTLKVAEKEIQFTLTVTYLCHVPKIICYVYFVITVIYKSKFSGYNLVPLFDMILACMAVTAPAVFAELTKNTVDKIKKILGSQLLRCSDESLRYELEITLEYVIQRPFSFSIWRAVSLDASLPVAMTSLCITYVIVILQLTQLRP

>BmGr30-5

MYLRSKKSRFKLFSFERMIKILLMICGHYVQTDSSNVVSSIHRIFSIVITICLCPYFQFNPFFFHVIESVLYSILSQFTQYGFFFRYCSTIKTFDLLSGFKQIPLYTKRVCFFLLITLLVRLIIVLIHFSAHQTKLKTFCAFLIILSANTGHILMTIMFSILNTRMTLIQKLFANNPIPVNIVGKNQNASHIKRVRKGLICYNNLLDTLKVAEKEIQFTLTVTYLCHVPKIICYVYFVITVIYKSKFSGYNLVPLFDMILACMAVTAPAVFAELTKNTVDKIKKILGSQLLRCSDESLRYELEITLEYVIQRPFSFSIWRAVSLDASLPVAMTSLCITYVIVILQLTQLRP

>BmGr30-6

MYLRSKKSRFKLFSFERMIKILLMICGHYVQTDSSNVVSSIHRIFSIVITICLCPYFQFNPFFFHVIESVLYSILSQFTQYGFFFRYCSTIKTFDLLSGFKQIPLYTKRVCFFLLITLLVRLIIVLIHFSAHQTKLKTFCAFLIILSANTGHILMTIMFSILNTRMTLIQKLFANNPIPVNIVGKNQNASHIKRVRKGLICYNNLLDTLKVAEKEIQFTLTVTYLCHVPKIICYVYFVITVIYKSKFSGYNLVPLFDMILACMAVTAPAVFAELTKNTVDKIKKILGSQLLRCSDESLRYELEITLEYVIQRPFSFSIWRAVSLDASLPVAMTSLCITYVIVILQLTQLRP

>BmGr30-7

MYLRSKKSRFKLFSFERMIKILLMICGHYVQTDSSNVVSSIHRIFSIVITICLCPYFQFNPFFFHVIESVLYSILSQFTQYGFFFRYCSTIKTFDLLSGFKQIPLYTKRVCFFLLITLLVRLIIVLIHFSAHQTKLKTFCAFLIILSANTGHILMTIMFSILNTRMTLIQKLFANNPIPVNIVGKNQNASHIKRVRKGLICYNNLLDTLKVAEKEIQFTLTVTYLCHVPKIICYVYFVITVIYKSKFSGYNLVPLFDMILACMAVTAPAVFAELTKNTVDKIKKILGSQLLRCSDESLRYELEITLEYVIQRPFSFSIWRAVSLDASLPVAMTSLCITYVIVILQLTQLRP

>BmGr30-8

MYLRSKKSRFKLFSFERMIKILLMICGHYVQTDSSNVVSSIHRIFSIVITICLCPYFQFNPFFFHVIESVLYSILSQFTQYGFFFRYCSTIKTFDLLSGFKQIPLYTKRVCFFLLITLLVRLIIVLIHFSAHQTKLKTFCAFLIILSANTGHILMTIMFSILNTRMTLIQKLFANNPIPVNIVGKNQNASHIKRVRKGLICYNNLLDTLKVAEKEIQFTLTVTYLCHVPKIICYVYFVITVIYKSKFSGYNLVPLFDMILACMAVTAPAVFAELTKNTVDKIKKILGSQLLRCSDESLRYELEITLEYVIQRPFSFSIWRAVSLDASLPVAMTSLCITYVIVILQLTQLRP

>BmGr31

MYLRSKKSRFKLFSFERMIKILLMICGHYVQTDSSNVVSSIHRIFSIVITICLCPYFQFNPFFFHVIESVLYSILSQFTQYGFFFRYCSTIKTFDLLSGFKQIPLYTKRVCFFLLITLLVRLIIVLIHFSAHQTKLKTFCAFLIILSANTGHILMTIMFSILNTRMTLIQKLFANNPIPVNIVGKNQNASHIKRVRKGLICYNNLLDTLKVAEKEIQFTLTVTYLCHVPKIICYVYFVITVIYKSKFSGYNLIPMLDMILACMAVTAPALFAELTKNTVDKIKKILGSQLLRCSDESLRYELEITLEYVIQRPFSFSIWRAVSLDASLPVAMTSLCITYVIVILQLTQLRP

>BmGr32

MCYTNFVSRQVSKCINFFSTIRYVIYLRMFCGLYYNCSSSFKIRCIARLYCFIIYCLNLHYNLYIFTSSVSLTNFLHTFITLAEVSIHILFSLYTGESNFMSFCIEMNKLTSGPIDFVATKCVATHFIAFFVIGLHILSSTLICGAEVSCFTFSVVLASMTFLTTLLSRFTTIIMFDLVWIRMRSLRKILVNALESDLSEDEKVKSIESFLKAYKQIIASIRITKLATRNLVTFNFVSLFGKIMTLIYFCINCPGYLNTYLISSWIFGILLAGFVTCAPPVLVEMNVNELDEIKYALADQLVDYTDDKYRTAIYNALDYVEVHSIRYTLWKNFPMDLTMFFGFAGFCATYIIGLLQFTY

>BmGr33

MCYTNFVSRQVSKCIHFFSTIRYIIYLRMFCGLYYNCSSSFKIRCIARLYCFIIYCLNLHYNSYIFTTNVSLTNFFHTFIILAEVSVHILFSLYTGESNFISFCIEMNKLTSDPNEFIATKCVTTHFIAYLVIVSHILSSTLICGARASCFTFSVILTSMTFLTTLLSRFTTIIMFDVVWIRMRSLRKILVNALESDLAENEKAKSIESFLNAYKQIIASTRITKLATRNLVIFNFVSMFGRIMTLIYFCINNPGYLDTYHMSLWIFGILLAGFVTCAPPVLVEMNVNELDEIKYALADQLVDYTDDNYRTAIYNALDYVEVHSIRYTLWKNFPMDLTMFFGFAGFCATYIIGLLQFTY

>BmGr34

MCYTNFVSRQVSKCIHFFSTIRYVIYLRMFCGLYYDCSSSFKIRCIARLYCFIIYCLNLHYNLYIFTSGVSLTNFFYSFITFAEVSIHILLPLYTGESSFMSFCIEMNKLTSGPNEFIATKCVATHFIALLVIVSHILSSTLMCGARASCFTFSVILASMMFLTTLLSRFTTIIMFDVVWIRMRSLRKILVNALESDLAEDEKAKSIENFLNAYKKVIASIRITKLATRNLVTFNFVFMFGKIMTLIYFCINNPGYLNTYLISSWIFGILLAGFVTCAPPVLVEMNVNELDEIKYALADQLVDYTDDNYRTAIYNALDYVEVHSIRYTLWKNFPMDLTMFFGFAGFCATYIIGLLQFTY

>BmGr35

MYSSLKLKDYIVNLESVMCSDQSITISFKNVFTVFFDYISSLDFMMVCRLCFGYYYEFNCSNLCKIMFKCFSISVCIFCVSMHLVQLISPPYLNHCVIMLESTVSIITSLVTEDKYFFEFCLDMKDINSMMNQPRNIKSFKIIYVIISGAICHVIRHISICREKALSFCFSTEYLTASFTIISGYWNYLNITMMFDLLYQRLVAVKQMLTNGLNICDTDEYKIKSVQKFIDVYKALTVSLSKTSNLIKHTVSLGIFCALWRIIFFVYYCISMDFQVESAQFITWVSSMCLSVFLVYIPALIVELCSNEVDAIKWILASELLEYRDKRLRTSLRDALDYIDVCPIDFEIWHCFPMNLSLCLGFIDISSSYIISILQFKY

>BmGr36

MTVPYDKIKSALSKFVQLLFSINVVLFVRFLFGFYMKIGSRKYFHIATKIWIVTLTIFRVYFQCRNFMNYPSYALILHDFTCTVELVILCIISSLGGEQHFYTYCSEMAELIDNRKNKRASYFTTSTLLIGFIILIVPTSISCKKVSNALSMLFLNIFNYVACFMHHLTIIYVFELLWREIRKCRISLERLEIMSVDDKIMKIENFLDSYKRSLDSLNKANGVMIPTMALAYFAIIAKIVFFTYNILSLRGFNILVHGDSWLLSTSIAIIFICAPALLVELAANEVNKIQNLLAVELLKMKDDKF

>BmGr37

MSAKINYQDKLCSIKSIMYLGLFCGLYFRSSTSRMMLLMTKVYCIVLLFLGISFHLNMLSSDLPTETTLHSSIIMLEFFIHIITSVSTGQAKFLHFCTEMMKINGHNSKGSLDFQLVITNIALIIIIITQTTSSLLYCIIRSKCLSHSYVFTVITSLCVLFSSFTMIIKYELIWNTVRSLKNTLVSNLDSFDLSEQEKVNSVYNFLSTYRDIKANVDLTIKGTRTTMIFNLFFLFVKILFLVYYCIINAENLDCLHASARVSAIVLSVAVTCIAPILVEINVYEFGRIKFALADQLLEYTDNNFRIALHDALDYIEVHSIRCSIWKNYPMDLNLCIGFISVCATYTISILQFSY

>BmGr38

MKPKYVTNIKLVLFLRFLCGYYYEMEIPRRLKTVAKAYCIFFLFFYLVLHHLYCSFSNHTAKWSLYLEYSIYVFMSLYSKKMYLMDYYTSSRIIDFEPHSRIYKKLNIYLAILIPFLVVLKIVNMVTFCLSKSFNCWSWVSLLHNLLWNFTVLGRIPPVFVFALLFCRTRIIRRTLVSITVGPGSTVLKSLFKCTRYWLTVLKRPNTHLNSCYFFQLTVFLLCSTPKLILETFLMLNKIKESGPIVEKLAVYAIETFYTHLFFVVSSILFDLINVDLQRIKILIVEKRMKTKNTKHRIEVEKLFQFVKSQTIECTLWRVLSLNVRNILSFVSFAVTTIIAVLQIKNNNIY

>BmGr39

MNVSPQYSVLKIFKPLFKVQTLFGSVRVKVGDNGITKTTKLQKFYSIFNILFATTGHFYTSFVYSVCVPCVGNSVAETSMALIQIYAGHLMNSFIVFSNTFLHYEKNVQMFKSLCSIDELMKIVRLEHRDLKLFIAIILLLSSTVIMNVYFLIYMVIILPISEKWVPFANIGIMNEDLEAITFVSVLYMLYDRVKYINQTALDPVNIAKLIKESDGQNDEETVSRILKAFKEISKAYKIVEKTFRVFFMFLVIHMFFFQMLTVEILIVLSDNLSWGTFMSKNLIGVKFIARIMLISVCVSYLERELQKTKGLCNLAVRNCENDIVRCHLKNIYRIIDTEIEPMTVFGLFYINNVPLDLISLTATYTVVLLQFAFL

>BmGr40

MLPICYIKLVYNISSIMFLRLIFGLYFELNVSNRMRWLQRMYCVFISIVIMYFCYFKTYRILMTVKVPFILEYGMHVILSLYLRENFEYTRYDPIIDSAADTKNFYKKFDIFIKIFLFVAILLKM*SMLVFCVLWSGLCYTGFIGICGMNFIWIATFMGRMVLPLIFGILLCRIRMFRLTLQKQGFDNLPYNRFSPRRYIMMYDSIVRGLEKTDFPAKNIMFVFTICIYSKILTGLFDLISVLKREGPKLMNVMLFTLEFLPSYVLLMIYSVTLDMVSTEMKEILKIVTEKRVFCKDKSHANIQELCQYIKNNQLKYTIWRLVSLNMQSLLRATSFCIVSTIAILQIKDWNG

>BmGr41

MSENSLEEYIHMSFSPIYKYQKFLGSNRISLKAKNKITVANNWEKLYAFLWMLAASYSIHHFISFFYSYYYERSNIIFLACSLGISMHYLTYILTITYDKFLTREADIDLFIDIQKIDRLLKLDRCTVLFKKFRLINIFLLILVTVPFISGFLIHVFDYIDKPYKTFFLGLGVTITYVDVLVTAFFITKLTLRLAYINDRIAMYNKINIPHKKYSGIRRSILWIFGWRIFKIMPKIKKNGTREKKSTFIKYPSIIFNILKCYRSITEIYSLPVFLITATVSIWTFLVIGSLVAGSRSEIKIFPVVAMITVGLWNFYYIIQLTSLAFVNDLFLMEVKNTKQLCISVLLYTCDDSINKAANTILKNIECVPPIFSVYGIFVFDKSIILFLFGIITSNVMTVIQFSY

>BmGr42

MNKTKKIERLSRDILDEDFIDVFKSIFIFQRIFGLLSVNITYKYITETSKLYKLFVMSLWTVNVLCLVDYILNYRTSFDVATDSMLKLVMSVNVTTNALIVWRNNFKLNTLKSQIYVKLQNLDRDLKTKDAVTMNKKLSALSIALMICGFIWCTIWLFVYNAIAMNTFCVPLTIILSANVGNWLEMVLLFIIFYFVNVRAEYVNKLLRRRLNQTECPDRVFLIQNAKPSDTVSREFICGMQSLLEIIGNIKDIYQFPIFLSTCQVMLCILVIVQNLIISVKEQMGGRAHSPPGFKWLLEPIDIYNTSTMVDSMLCMLPALLLMLTIFFSLCVIAEALTSKLDITKKLCAMGMHSFTDDISRRSSKQIVLLLEAKRSMSVFNIYTLGTRLPIHLLGVTASYTIVLLQFAVL

>BmGr43

MKSPEYLSKDILDEDFVRVFRFPFLVQMALGSCRVHLKARFITIPTLGQKLYTVMSIIICSLLYFNITKLYISLYYQHSIVYYLFLAVAGLDQLSFFANLIHVRFLNGETNTGFCIMMQRIDRKMKIDHNNIFNKTVIRANILTITLIILLYMSLVISTIILKKYSLVTLFGLVHGQLILLVEMAYCSNLIIFFFIRVRFVNAIIKNHVHPENQNQPPKLVRYFVTNRIMRYLAAQTHDFIVNDTDVYLKQIFEGFSMFIDIYRFQASMPLLHKANCNEPANLRILLGWDTKKFFHTEFGSLINSPCDFAFFFSQEKIAGSPPARQVLASKNLANYYVIVHSVIGFITALYISGRCEFFFREIRETKRLAVAVLLKYQEGTILTIAYFPKLS

>BmGr44

MFPVVKIIMMIRLCFGLYYKVKCRFCSVVLKMYCVIYYIYCISAIVNMLLKIWTTPVGRARW*LGGSRVAICLFPFEHLALFLGVSCSYKISGMPVWTPLQIVVLSMTSGTRGASRCTTLVVLDVLRLRMNFLKRLLEKTMQSEISNQQKYQFLEQFAEHYRYLLEALDSTRPHSAIVVTTMLMTSFVKVLIIIYFVITNSIMTIDNSRLTYMMMTSFEVAAVSVAPALLEMANNEKDQIIMILADQLLKYTDSKLRRSVYDVVEYITVAPPTVTIWPEFTVDLGLYMDFIGLTVCYCIMILQFEY

>BmGr45

MKSPEYLSKDILDEDFVRVFSFPFLVQMALGSCRVHLKARFITVPTLGQKLYTVMCIIICSLMYFNMTKLYLPLYYEHSIVYYIFVTVTGLDQLSFFANLIHLRFLNGETNTAFYIMMQRIDRNMKIDHNNIFNKTVTLANILTITLIILHYVGLVISTIILKEYSLLSLFGLLYGQLMLMVEMALCSNLIIFFFMRVRFVNAIIKNHVHPENQNQPPKLVRYFITNRITRYLAAQTHDFIVNDTDVYLKQIFEGFSMFIDIYRFQVCPLCIKLVVLTLLNFEFCLVAIQRNVLGPNHIGNYYIIVNSVMGFFTALYVSGRCELFFREIRETKRLSVAVLLQYQEGPLREKATRMLKIIEESTPQFSIYDMWQMDGYTFVKICSLVTNLIVTLLQFAYL

>BmGr46

MITMKSPEYLSKDILDEDFVRVFRFPFLVQMALGSCRVHLKARFITIPTLGQKLYTVMSIIICSLLYFNITKLYLPLYYQHSIVYYLFLAVTGLDQLSFFANLIHVRFLNGETNTAFCIMMQRIDRNMKIDHNNILNKTVIRANIFTITFIILIYVVLVISTIMLNEYSLVTLFGLLYGQLIFMVEMAHCSNLILFFFTRVRFVNAIIKNHVHPENQNQPPKLVRYFVTNRITRYLAAQTHDFIVNDTDVYLKQIFEGFSMFTDIYRFQVCLFCIKIVVLSLLTFELCFVAVQRNLLETKNLTNYYIMTYSVIGFFTALYVSGRCELFFREIRETKRLAVAVLLQYQEGPLREKATRMLKIIEESTPQFSVYDMWNMDGYIFIRICSLVTNLIVTLLQFAYL

>BmGr47

MIDFKTRNVCDINTIMKIRFLFGFYCDFPFNKRFQNILKFYCISVLVVLILGSWACSTGFRSDKKIVIYCEYIAYFLISLSTKDRYIFDYYKQQPLIDGSTTSKVLYKKLERLLKYFVTITIVLKMLNIFVFCGWNLTKCINELDGVLFINLLWIGLLLARLSLPVIYGLLYFRLRVLRMTLESKGFSNSPQNRFTPKKYITIYEKIMKDLLKMDYPLKYVFIIFLIGSVPKLLQNSWQFLNSLKNYGPEISKILEFTLECLHSYIVIILPIVVALDLSEDEIKKMKIITLNKRLACLNERQKMEIQQLFLLLKNNSLRYNLWRVVPVNLKSVLIFLSFGVTNAIAIMQAKNLN

>BmGr48

MTVFMLKAQVDQDTILEKVVKPLNNTMFNNQEWSFQQDSAPGCSLGTSSQYLTYILTITYDKFLTREADIDLFINLQKIDRLLKLDRCTVLFKKIRLIYIFLLILVTVPFISGFLIHVFDYIDQPYKTFFLGLGLTIIYVDVLVTAFFIANLTLRLAYINDRIAMYYKRSLPLRKDSGIRRSRSWICGSWIFQIMPKIKNNGTRMKNYTFIKYQSLIFNILKCYRLITEIYSLPVFLITATVSIWTFLVIGSIVAGSRSEIKLFSIVAMITVGLWILFFIIQLTSLAFVNDLFLMEVKNTKQLCIRVLSYTRDDSINKAVNTILKDIEYAPPIFSVYGIFVFDKSIILFLIGIITGNIMTVIQFSY

>BmGr49

MAGIRTISFKVKPLELPDVSENNFADDGLKIVQRFKFFIYIQVITGINRLYLLKCNKFVMLFSYLYAIFLISFVASVYWTKEPMKNSHLVIRLFSFIEYILLICISVFLKKKKMMKFFENLSMFDQILKIDKNVNSTFCMKRVFFWVTGSIVYNLIEFYALEFYDNTSKGLMTIICTYTIALTHDCEQIFFFTLQRVVYLRLLVVKRHIQEHFKVDEDSNRKKPNKYEMLSKNVQLNLTALHEVYGLLHNCAEKLNKIMSIPVLLMLFTSGLTTTILLKILVRVIQLADPSNPGPAAIGVCVYLIVHCIKYTLLVVIPCYYSSITATQVSLIRITLHDAINTIPLGKCKLQRRKVKAFYLMTKEYSFVYTLAGVIKLNMSLPLSYISLCTTYLVIIIQFSKFLD

>BmGr50

MAGIRTISSKVKPLELPDVSENNFADDGLKIVQPFKFFIYIQAITGINRLYLLKCNKFVLMFSYLYAIFLISFVALVYWTTEPKKNSHLVIRLFTFFEYTLLACISVFLKKKKMIKFFENLSLLDKMLKINKNVNSTCCMKQVFFWVTGSIVYNLIEFYAMEFYDNTNKGLKTIICTYAIALAHDCEQIFFFTLQRVVYLRLLVVKRHIQEYFKVDEDSSRKKPNKYEMLSNNVQLNLTALHEVYALLHNCAEKLNTVMSIPVLLMLFTSGLSTTILLKFFVRVIQLTDPSNPGSAIGVCMYLIVRCIKYTLLVVISCYYSSITATQVSLIRITIHDAINTVPLGKLQRRKVKAFYLMTKEYSFVYALAGVIKLNMSLPLSYISLCTTYLVIIIQFSKFLD

>BmGr51

MAMGIRTILSKVKPLELPDVSENNFADDGLKIVQRFKFFIYIQVLTGINRLYLLKCNKFVMLFSYLYAIFLISFVASVYWTKEPMKNSHLVIRLFSFIEYILLICISVFLKKKKMMKFFENLSMFDQILKIDKNVNSTFCMKRVFFWVTGSIVYNLIEFYALEFYDNTSKGLMTIICTYTIALTHDCEQIFFFTLQRVVYLRLLVVKRHIQEHFKVDEDSNRKKPNKYEMLSKNVQLNLTALHEVYGLLHNCAEKLNKIMSIPVLLMLFTSGLTTTILLRILVRVIQLADPSNPGSAIGLCVYLIVRCIKYTLLVVISCYYSSITATQVSLIRITINDAINTIAFGKLQRRKVKAFYLMTKEYSFVYTLAGVIKLNMSLPLSYISLCTTYLVIIIQFSKFFD

>BmGr52

MAGIRTISSKVKPLELPDVSENNFADDGLKIVQRFKFFIYIQVITGINRLYLLKCNKFVMLFSYLYAMFLISFVVLVYWTTEAMKNSNLVIRNFTCLEYILLICIAMFLKKKKMIKFFENLSCLDKMLKIDKNVNSTCCMKRVSFWVAGSIVYNLIEFYAIEFYDNTNKGLVTIICTYTFALAHDCEQIFFFTLQRVVYLRLLVVKRHIQEYFKVDEDSSRKKPNKYEMLSNNVQLNLTALHEVYALLHNCAEKLNTVMSIPVLLILFTSGLSTTILLKILVRVIQFTDPSNPGQQSECACI**CIASNTRC*L*SRAITQASLQLKFLLFVSRFMMPSTLFHWVSKLQRRKVKAFYLMTKEYSFVYTLAGVIKLNMSLPLSYISLCTTYLVIIIQFSKFLD

>BmGr53

MAHIKDENQSKQQQKEHETLNKNKLKKVVYTLKPALMLENWFGLSDFLLVNEDELVLLMQTEKFGVILSIFFIVMFAVFVDFPDTETESIMELMDEVPSMVVLSQYFIASITTSSCLSAIAIRIFETFADLDSMLLITTTQDFYNKSRYQTNKYLIILGVSHIISSTLDLLTDDEIVWCKFFVLPIYFLQKLEVLTFCKLIVMIQCRLQIINKYLTNFIEEQEKNKALVFTLAESNPKKTDKFNWIGCPSPNNMKIRDLATMYDVIGTICSLINDLFNIQIFMTLVSTFTYIVIAIWSTLYFYRAPNFTFGTLTTIIIWCITIILSVVVMSFVCERLVSVRNNTKILVNKVIMNYDLPKTMRVQAKAFMELIESWPLKIMVYDMFSVDISLMLKFISVATTYLIVIIQLSHFV

>BmGr54

MTHAALPRSEAYFLMTLSRSTIVSKRAYGPPDGEWLPSPMDFSNARGRAKPLPTVCLRVCVQNNLSFYRPILIILQLCGYDFDYYNINLVLNVLTKAYCASLTCVVVYATIACCSSIQLSHIWSLIEYGTSVVIIACFRSQTKLFLKQLTTLDVYLRISNRRFVLEKCKIFTITSVIFLLRIVYTSIYCSTHHCFNVLIYFLLSQFALVCLDVNRIWRCIVFDAIRYRLKTLRLRMEENPDCNYYLYVKNNKSIRKNKISFCLFLYRTIADLVDLVSPELNVSRSTTNICGLKTHIIRGAPKTADTSCTRYSSLPEKNQQCISILFLSVACSLPKIVSNAYHLLLIIEDREPLETGGYVLMHTLQVSLLLFTPFIIVECYTMEVEKIKLYLVHRLIDENDTTMRDNIRLFLEYMSVRTFRYRIFRIVPVNATLPLELVNLCVNYVIVLINFTHLYG

>BmGr55

MERINLLKSFAFLENVMCIYRNFMFYNQRARFIIIGRIVAELVFYIFSAYNGFLLVYTDWFSQNFSVFFIEIISKSSFYVITFFTMVNGILKSREYKTFIFSINKIHDYILNDTDYLKRLKCTNIFCTATIIILFVVTLIRTAIDGSNYGQLSGINARSVIWMLTTILLECQYQTECVVYFGFILFIHAIMKYLNIRVTNTIIKIARSDMAVKRIPKYIIGRTELKDETDTGVDVNNVVDLEEVRYWVFIYRQLGLTTELLQKCFGMQTAFIFVTAVLNQIITVFRVIAVFIYGSLANRGAEHSIIANFLFTLLYRLPGLLMIIVGGQMVQNQTDMLRRSMARLNNIISNNPHRETFSALSDFHRMIVKNPVKIYVLSVLPVGAYMLPLFMTLLINHIIILLQFNHVA

>BmGr56

MKKIRLLRSIVFLENLLCIYRNFLFFNKKARAIILIHITIELVLYVLSIVNNSFIIYSYFHSDNRSMLIVFTTICCFYVVTFVSIVMGILRSEEFKDLVTSLELINKFFTNNKTYLKSLGRSNTMIIAITTILYCVTCIGIAVDKITLNDFYEFTSSDVIWTVSSTLLELRYQTECVVYFGIEYLFLIFTKHLNLLVKEAIKKVSLDNNGTVKDVPISSDAVTKNEVKRWATIYRQLMMSSKLLQACFSLQIICVFVSAVINFITTAFRMVKVSVLGSIATDMNEIIIVNLIFTLLYQNIGLVLIIVTGQRVWNQILLLNVLLARLYNGILIQPCRDTLRTLKNLQRMVVKNPVQIKMLSVLPVGSYMLPMFMTLSVSYIIVMLQFGHVV

>BmGr57

MEEIKAIKLVTFIENCICVYRNYAMCTKRNKKIISLRIIVEIIIVFFVNINNILLLHKYYNGSGLLYIIYLFLVVYYINYMFCIFYGALQGKAYRQLIFCFNKINAIAKRDKSYKKSLARLKNMCIVISIALLIISALSVFVDRSNSWNIYEVSLRDSLLILSKIHMDFFYHFEYVVYFTHIKIFHLTLRYLNSRVKMAQFEMKMTRRDVHDEGERNIRILLTKELTTEWAVLYKCLVFGTKTMKSLFGLQMLIAMVMSFVNFTLSLYGIILICSIEQSQTASQHNLLLILTYYTATMLLIFIVAQSVYNEVEMLKRNLARMYNILAVDSDETQQKLVKDFLRMVYKNKVEIKMLSIFPVGMPMLTFFLSLSASYVVVMVQFSNVF

>BmGr58

MSSRRVLYRAEVLLSNNVDAHVQDMLKPLNFFQFILFFPKYTIRDGYITPNSLIRNIWSATGAFVFISICVFRILTMNKIAVYDTFTTMLLISKYFDVALYCIGFIVNTYVNIAYSNVNVLLYLKLQTIKTFIPRNNEIMKNVKWYSVILIIVLFCGTLAMFSFFHLSFSYFNIFDLTTDLAVFSFDLNLVYACSVLNFLAQSLDELNKEIWRLGNAKVTVCKDGSKPDWNGINLTYINVLDAYNYFKEAFRLLIFFHTFKTLTHMFIYIQSIIELCKKFYPGDDYDAITVGAVVGVWFFRNITLQCLVGVSCQNFYSATSNTESICAVQVGSIVSDEHKLFLKAVRRLNNVVFYKWSMYGMFIVDATLPRRLIELIATYTVVFLQFAFK

>BmGr59

MPYKKDSNRCEVLLYNNVDTDLQDMLRPLNFIQTIYLSPKYTIKDGYITPNSLFCNILSAAGAIVFFSICVYRILTASKIGTFEGFSTTLLITKYFDAILFSLGFVANAYVSIRLSHLNVLLYLKLQAIKTFVPCKKIMQKVKYYSIVLIIGLIIVRLIMYIHFHWSLGYLSYLDLITDLGVISFDLNLVYASSIVKFLGYNLEELNKEILRLDEIKATMDEEGSKPDWNGIRRTYLKFSEAYNYFKDAFRILILFHTLNTFAHVFIYVQSVIELCKAPADNYMGGRAHSPPGVKWLLEPLDIYNSAAFSVLIAVIVWLLRNIILQSLIGISCQSFYSATSNTQSICSILVRSVLSGEYNQWFLLKKYDVETHFFIAYVVGRAHSPSGVKWLLEPIDIYNVNSTTHLEI

>BmGr60

MLTPRSDLCNEKLSPSFPSGKTTAADKDDTEARCQVDSSLERLLLPFNLVQHVSFIPMYSIRRGLVSPDGPLAYLYSLLGFCLFTSVSVYRNAIMHGTRLSSLHLFTLYSDLVSFVINYSLSLICNVVNSKSNVEFVCRLQRLQTVLRRNQREQEQFARSNWAHLAVVTALYLAVVGLLNVVVLKQSLPDTLYLLLLFCIDVNVLYATRMLALLRCYLQLWTRKINEKAFNPVHHNMFTAYLDILQEYEVYTTLFKKIITYYVLETFLHGLLYVQVAIQICKSIRRSGRFSEQLMMIVSIFTWTIKNMIIMTLHNVECEKFYLAVEQAVAACQTQRASTTRCREEKRLYKNVCRVSRAAFSRERGWGLLAAGAALTLRFMDLATTYVTVLLQFAFVSRT

>BmGr61

MSIRFEKDLLHNYVEIELQYFLRPFNVMQSLFFQSKYRIVDNFILPNTLFKNIMSFVVSVLCALSFIYTIISVWQNTHATSFHALVTSVYLSYNIYGILIGSVLIIWLSDRNIEFVLKIQDLIKILEFNKCFLIEYAFINSIIMAAIFILNFLLYGYFVVHLQKFALGLTFSAIVCILNQDLDIIYVIIFANILKKCASRWTVEARQKNNFNDQGKWVKLFNAFLNLTESYQLYQKIFEFYELLRRVGIVFLGLQLTVCRVCSNDIKSIQCTVMLHAFQLICVWIVKKFITLSILSFEMEIFYEKLREIETVCIILVSSDNPSERELKIWKNIIRVSSCSVRKTTACGLCEVGAALPQWLLQATTAYTIVLLQFHITTFSRATNDIYDLD

>BmGr62

MNDLFLSKIVKWTKTTKYKLDDDFQSLFRVFNIAQAMNLCPKFLIYDKYITNNAWFIHILAISSFIVLVCLDSFFANFRLVLSEAMGPPFYGFSFYFISILYENIGVIIQITMNGYLTKNNVLIITKLQDTFKDFRTTDYITKSNRWTNWFIFFIYMNFIANYSYFNFYVNTFSFHKFCFAFIKMCFDLNIVYTIFIFKMIGDSLTMFKDTAFCSKNMKLYEVSNRVYWNKMLRLYSNILDVFELSKRTLNFFIFYFVSNILLRILSHVQLAILMNSINWLQHVAYSNIVMVLLTLAKEGIILIVLIAKCEKIYCVIGDVQTACQLALGNAACPEKRRFCKNVRRSSSAAFSKIYICNILAVDAKLAVSLMSVTTTYTIVMLQAILIK

>BmGr63

MQIGNAVIHLKSTKLTTMNTISPTTKLLKIFALNSNIEEIDLKCSTKLRITMTAFVLCSLIFYSLYYKFIYVFDYVNISIKITDCVQMVYDFCQYIVDLYFVTNYGRNISSEYFQQYKIIDKILEVVCYEIIKHRIVKLLWVFMCIWFSSSCFDFIAWFLNYGWITPLVYSVAYIFLLIKILTTLDLSAHIMNVEIRLKMIADLIHHYYMSCEDNFQAEETLCHKNWLNSKERAKYYELQFRIHALKQLSCNNNEIKLLSRCYLMLTEQVEIINRMYGFRILLNSLSLLIDMVRFTNISVRIMIGSQNLAYNCGYFPAVSSIFRLLTCGAVIINLVSHCERVYYQRTRICNVIDHMIVNKNLSRESTEALQEFRNLVQNHPIEFNMANFFQLNYSLLVSIASVVVTYTIILLQSVN

>BmGr64

MKISLRKIVSIRNMTLIQNMFGFYHKFTDNRAIGVLLKIFCGFYSLFLSFLCINCTPRFTNDFLTYDIFFFVIEYLTSVLVCLLYDGQYFLNYLYDLKLIDREAGIEESLEKLPISQPLFSLIFITRVIYLLSCLLMFDGIKDSLFLPAQSSVFGANFTEFARTIGYFPRVIMFEMFYKRVNYLKSQLRNDLAHANLYPIGFVCSKVIMKYINFYKLLLRNLQQNSLQFKILMSMSSLYIIIKALASAYAFIYREDGVHVFIFIEFATGVFLFFVMSSIIISIFNEIEDIRQIVLAQLRYCKQGANTKRVQDALTILNIRCFKYALCRIYTVDFTFILRILDVSVTYVIVLVQFTHILD

>BmGr65

MKISLRKIVSIRNMTLMQNMFGFYHKFTDNRSIGVLLKIFCGFYSLFLSFLCINCIPRFTNDFLTYNIFFYVIEYLTSVLVCLLYDGQYFLNYLYDLKLIDREAGIEESLEKLPISQPLFSLIFITRVIYLLSCLLMFDGIKDSLFLPAQSSVFGANFTEFARTIGYFPRVIMFEMFYKRVNYLKSQLRNDLAHANLYPIGFVCSKVIMKYINFYKLLLRNLQQNSLQFKILMSMSALYIIIKALATAYAFIYCEDGIHVFIFIEFAIGVFLFFVMSSIIISIFNEIEDIRQIVLAQLRYCKQGANTKRVQDALTILNIRSFKYALCRIYTVDFTFILRILDVSVTYVIVLVQFTHVLD

>BmGr66

MSPPLVHINTFVQPQAKYTVDKVSKFFIICSFLLGVNRLPIISSKHVYTIPSIIYTFVLMCVLNFFGFDSVSLSIMSLNLVLHILCSFLGMFFWKRMRLYYSELCKFDICIGCRPITAQGSSKLVIQTCIINVLIALVFIVPNSLQILIKPVIYLLPMHAFVSFEVHYYGHLLNLLIPRLHLINYYMESSLTTTSDKRESSVLKHVILFKYYNKESNCQMKKFMDLYYIIVESYRYLIDAIKWQLLFIIIVSFISVLGFCYHFSLHFLRGKNIADCLVTDLGLALVVMIPLFVPCVFGDKVHTEVKRLRELLASRLYENQMDKSSRSIARALLAFTETRDLSFSLLRMLNIDISLPFKFVGLLVTYLIILLQFEKVINP

>BmGr67

MRERKKKFNKLLNTRNYNNIVEALLPSDSIRKISGVSVVYLAVNSENRIVTKFSFIGTIFFLFWYILYFYCTYKAHSEDQTILRTIYNTKLKRYGDDFERIASIIYVTYSMWKVPFRMSGNQVFIQRIVDIDSAIENMGEAVDYNKNAKTALVISIAQLGDFLVRMFCIWLSLENLSVIVPTEKLYQVVYTDALSFVITSHYCFSLIVLRGRYKYINKVLSEIKTRSAWEYKVFVRNKVAPDLEKVQRLQDRIVCEKIKACARIYSMLYKATEAINRMYGTALVLTMLLYLVFIILYMFYFMEATASGLLYDIKKYVDFLICVFWQMSHALSIIYANVYFSESITREVCKF

>BmGr68

MKRKLKKFFPNKEYNNIVEATHLWKLIRKLTGLSVLTLESKEGNRIETRFSSLGFVFFLLWFTIYFYCTYKAHNEDQTILRNIYSTKLQRYGDDFERITSIIYVLYSMWKLPFQISGNRLLLQEIVDIDKAIESVGVTIDYKKNATFALFIYIGQIATYLFRLFCVWGCLGNLNSPVPVEKLYQDIFTDALSLLLTSQYCFSLVILRDRCRYINKILCGIENRESSRLRLFVYSSMPGAEKDITCRKIKDCSKIYGMIYKAVESTNITYGFALVLTMLLYLIFIILYMFYFMEATAAGLFLDTKKYIDFLICVLSELLHAMLIIFLNIYFSEETVKETRTTSFVIHGIINSDFNTQAKTEAIHFSTQLLHQIPKFTASGL381VELNYSLLYEVGGGILVVTP401MGSGNNLATDGPRVCSPI

>BmGr69

MRFGLKAGAAVVTILRPYNLCLKNIFKPFYVMLSLLGLFPYSIRFLGGKQFLIKPKSIYTNAVCALSLMLSMTLFLIFHIDHIIYKSTEDNSLTEGFMTQVNYIIEMLNLEIFCVVYYFSSFLNRNKFVKVLNTVAVWSDRISISGIKTLSFLRLKIHFSIGILMFLLISQVCVNFTRVDSLWKKVLVMFTFNIPQMIQFTAILFYYILVNMVITLLVIIQENISISTRDTKTSSFIRVEHRMPLSLKQLELIYIKAFELKRDINKAFEAPILLTTMQCFHSIVSESHIIYHGAVMEPHMVLHSIMNCSVWILYQLFKLYILASTGHLLQEKIQHFSNLIHFHGKGLTVYGLFPLDGTLMFKVVASAAMYLIILVQFDKRN
