# Supplementary material for: Antennal transcriptome analysis of the chemosensory gene families in Carposina sasakii (Lepidoptera: Carposinidae)
Source: BMC Genomics. 2018 Jul 20;19:544. doi: 10.1186/s12864-018-4900-x (PMC6053724; doi:10.1186/s12864-018-4900-x)
Supplement: Supplementary file 4 — Figures S1–S3. (DOCX 1489 kb) [file 12864_2018_4900_MOESM4_ESM.docx]

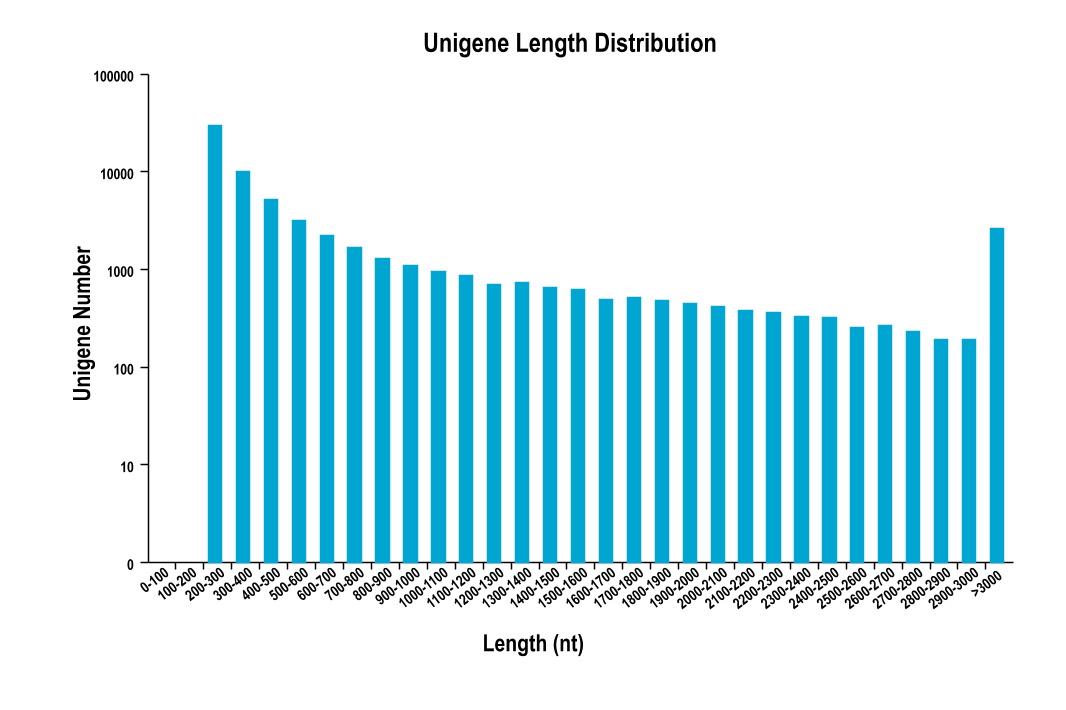


Figure. S1 The length distribution of the assembled unigenes in the *C. sasakii* antennal transcriptome


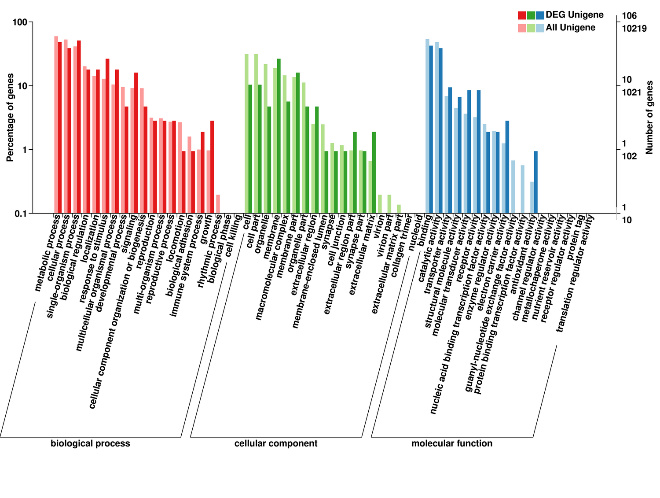


Figure. S2 Gene Ontology (GO) classifications of the differential unigenes and all of the unigenes in *C. sasakii*

According to the biological processes, the functional annotations were classified into three categories (molecular function, cellular component, and biological process). The light colors and the dark colors represent all unigenes and differentially expressed unigenes, respectively.


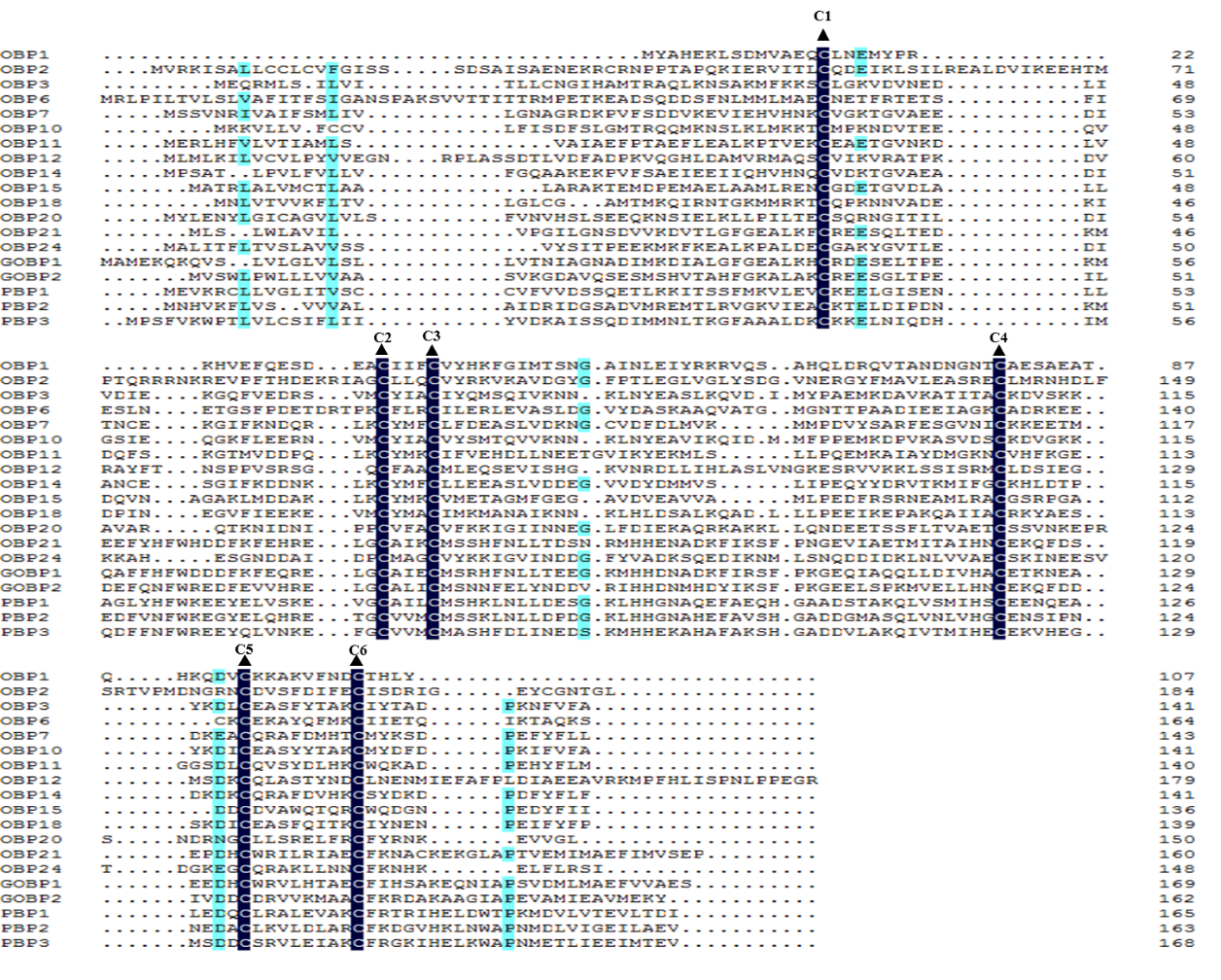


Figure S3. Alignment of candidate *C.sasakii* OBPs. The highly conserved cysteine residues are marked by dark triangle above.
